# Synthesis molecular docking and DFT studies on novel indazole derivatives†

**DOI:** 10.1039/d4ra02151g

**Published:** 2024-04-23

**Authors:** Bandaru Gopi, Vijayaparthasarathi Vijayakumar

**Affiliations:** a Department of Chemistry, School of Advanced Sciences, Vellore Institute of Technology Vellore – 632014 India chinnabandaru389149@gmail.com bandaru.gopi2022@vit.student.ac.in kvpsvijayakumar@gmail.com vvijayakumar@vit.ac.in

## Abstract

The amide bond is an important functional group used in various fields of chemistry, including organic synthesis, drug discovery, polymers, and biology. Although normal amides are planar, and the amide has an N–C(O) bond, herein, the 26 indazole derivatives were reported *via* amide cross-coupling (8a–8z). Using IR, ^1^H NMR, ^13^C NMR, and mass spectrometry, all of the produced compounds were analysed. A DFT computational study was also conducted using GAUSSIAN 09-Gaussian View 6.1, which revealed that 8a, 8c, and 8s had the most substantial HOMO–LUMO energy gap. The effectiveness of indazole moieties with renal cancer-related protein (PDB: 6FEW) was assessed by docking the derivatives using Autodock 4. The analysis showed that derivatives 8v, 8w, and 8y had the highest binding energies.

## Abstract 2

3-Carboxamide indazoles have been developed using the amide coupling process. Density function theory (DFT) computations, and the assessment of binding energy using Auto Dock, illustrate the pharmaceutical effectiveness.

## Graphical abstract

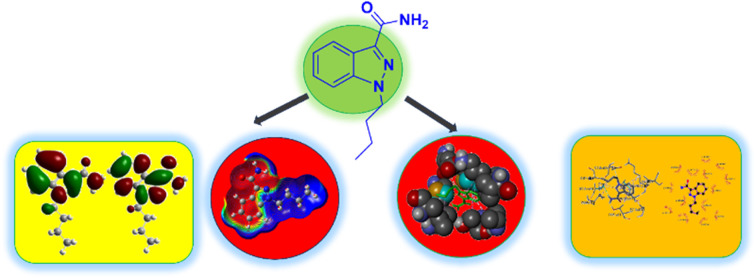

## Introduction

**Fig. 1:**
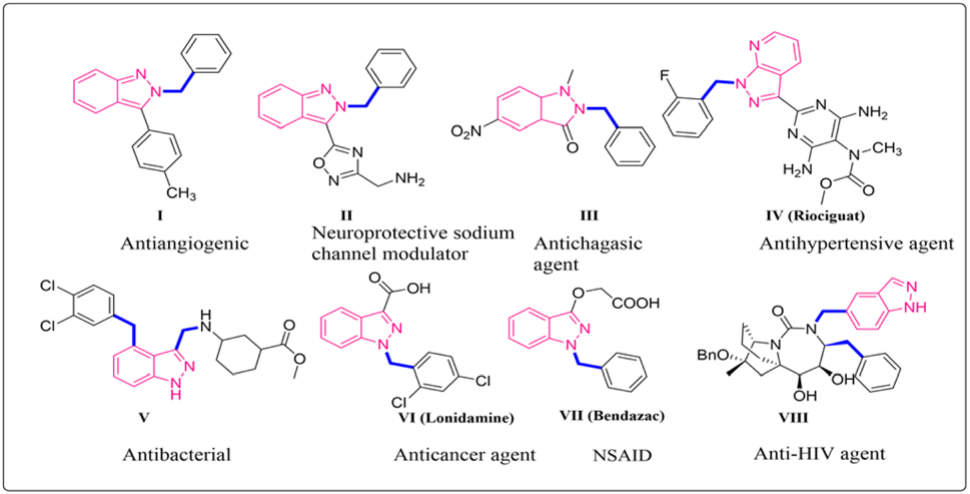
Some representative examples of pharmaceutically important benzylated indazoles I–VIII.

Indazole is a significant heterocycle in the field of medicinal chemistry. It has been used in the development of numerous commercially successful medications and potential drugs with an indazole core.^1^ Its pharmacological and biological characteristics make it a valuable compound in drug discovery. Fused heterocycles that contain two or more pharmacophores have proven to have antitumor, antibacterial, anti-inflammatory, antiplatelet, antidepressant, and anti-cancer properties and can act as HIV protease inhibitors.^2–4^ Indazole is the main structural component in pharmaceutical compounds like bendazac and MK-4827 (ref. 5) which are important molecules in the pharmaceutical industry. Its potential therapeutic effects can be used to treat several illnesses making these moieties good anticancer agents (I, pazopanib, MK-4827, lonidamine VI), neuroprotective sodium channel modulators (II), antichagasic agents (III), antihypertensive agents (recoguat IV), antibacterial (V), non-steroid anti-inflammatory drugs (bendazac VII) (Fig. 1).^6^ The unique structure of indazole is due to its planarity and the presence of two nitrogen atoms, which permits the alteration of its sites to produce a wide variety of biological and medicinal variations.^7^ As a result, it can act as an anticancer agent. In 2020, the International Agency for Research on Cancer reported 19.3 million new cancer cases globally, with colorectal, lung, and female breast cancer accounting for 10% of all new cancer cases. Renal cell carcinoma, which affects primarily males and accounts for 5% of all cancer diagnoses globally, is the most widespread and dangerous cancer of the urological system. Renal cancer mortality and incidence are predicted to increase in the upcoming years, with over 300 000 fatalities predicted by 2040.^8^ Therefore, the synthesised indazole derivatives can be widely studied, particularly against kidney cancer which helps in expanding the chemical library for renal cancer therapeutic screening.^9,10^ Many synthetic methods have been reported in synthesizing indazole moieties due to their biological significance. In our work, we are focussing on the synthesis of *N*-alkylated indazole derivatives through amide cross-coupling of indazole carboxylic acids with various aromatic amines. The successful application of advanced tools and techniques in the field of chemistry can lead to novel conclusions. GAUSSIAN 09-Gaussian View 6.1, and Auto Dock4 have been utilized to study the density functional theory and molecular docking respectively.^11–13^ Density functional theoretical (DFT) calculation with B3LYP/6-311+ level was used to study the physicochemical properties and electrostatic potential of the novel indazole derivatives. The *in silico* studies of the amide indazole derivatives were performed to find the binding energy of the prepared moieties with the renal cancer receptor (PDB: 6FEW), thus helping in finding out the best ligand among all indazole derivatives. Based on this, the synthesis of novel derivatives of indazole molecules is being explored.

## Results and discussion

**Scheme 1:**
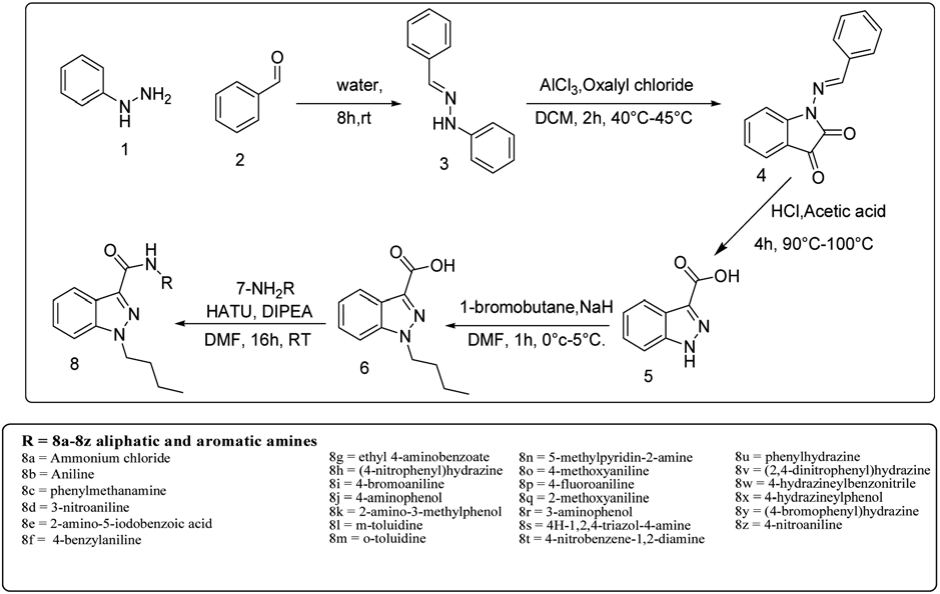
Synthesis of Indazole derivatives from 8a–8z.

**Fig. 2:**
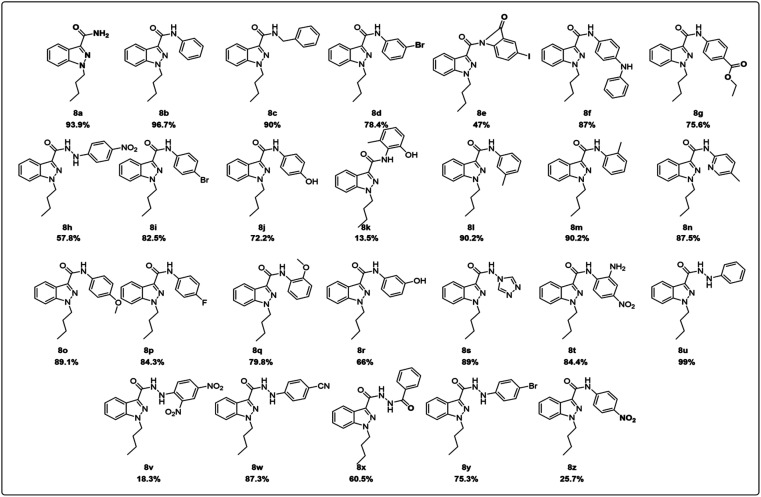
Indazole derivatives from 8a–8z.

A solution of phenylhydrazine 1 in water was treated with benzaldehyde 2 (1 eq.) at room temperature. The addition was gradual, and the mixture was stirred for 8 hours to yield benzylidene-1-phenylhydrazine 3. This compound was then subjected to a reaction with oxalyl chloride in the presence of AlCl_3_ (1.5 eq.) for two hours at 40 °C to 45 °C, affording 1-(benzylidine amino)indolin-2,3-dione 4. This compound was further converted to indazole-3-carboxylic acid 5, which underwent an alkylation reaction with bromobutane using NaH as a base to produce 1-butyl-1*H*-indazole-3-carboxylic acid 6. A simple synthetic approach was employed to obtain a series of 3-carboxamide indazole derivatives 8a–8z. The reaction was performed at room temperature using HATU (2 equiv.) as an amide coupling reagent and DIPEA (3 equiv.) as a base in DMF (10 vol) solvent (8a and 8b, Scheme 1, Fig. 2), All derivatives, except for 8e, 8k, 8v and 8z, have yields between 55 and 80%. The progress of the reaction and the purity of the target molecules were determined using TLC. All the derivatives were purified by column chromatography (ethyl acetate/hexane 1 : 3) and characterized by IR, ^1^H and ^13^C NMR, and HRMS spectral data. Further details on the synthesis and characterization of the derivatives are available in the ESI.†

## DFT study

Based on Koopman's relation, the LUMO–HOMO energy gap (Δ*E* = *E*_LUMO_ − *E*_HOMO_) and other frontier orbital energy characteristics, including the chemical potential of the molecules under test can be calculated. The molecules with the highest HOMO values can act as electron donors and the molecules with the lowest LUMO can be the electron acceptors.^14–18^ The HOMO and LUMO energies and global reactivity parameters have been calculated for all the molecules 8a–8z using GAUSSIAN 09 for DFT calculation 6-31+G (d,p) basis set and the obtained data has been given as Table 1 in the ESI.† Out of all the compounds evaluated, compounds 8a, 8c, and 8s had the biggest energy gap (Table 1).

**Table 1:** DFT calculated values of 8a–8z

S. No.	Compound	HOMO	LUMO	Δ*E* = *E*_LUMO_ − *E*_HOMO_
1	8a	−6.0453	**−0.86238**	**5.18292**
2	8b	−5.51394	−1.07163	4.44231
3	8c	−5.88654	**−0.81999**	**5.06655**
4	8d	−6.12522	−2.65032	3.4749
5	8e	−5.7888	−1.17666	4.61214
6	8f	−4.44879	−0.98955	3.45924
7	8g	−5.89761	−1.40211	4.4955
8	8h	−5.65434	−2.31201	3.34233
9	8i	−5.62275	−1.21878	4.40397
10	8j	−5.14566	−1.04112	4.10454
11	8k	−5.23692	−0.92853	4.30839
12	8l	−5.44563	−1.05111	4.39452
13	8m	−5.41485	−1.07244	4.34241
14	8n	−5.67216	−1.00143	4.67073
15	8o	−5.11137	−1.0287	4.08267
16	8p	−5.5039	−1.15128	4.35262
17	8q	−5.21937	**−0.92529**	4.29408
18	8r	−5.47317	−1.14426	4.32891
19	8s	−6.34878	−1.4283	**4.92048**
20	8t	−5.27715	−2.60523	2.67192
21	8u	**−6.8823**	−2.45457	4.42773
22	8v	−6.36687	−3.59964	2.76723
23	8w	−5.08923	−1.32435	3.76488
24	8x	**−6.67332**	−2.42568	4.24764
25	8y	−4.24305	−1.00143	3.24162
26	8z	**−6.4638**	−2.77776	3.68604

**Fig. 3:**
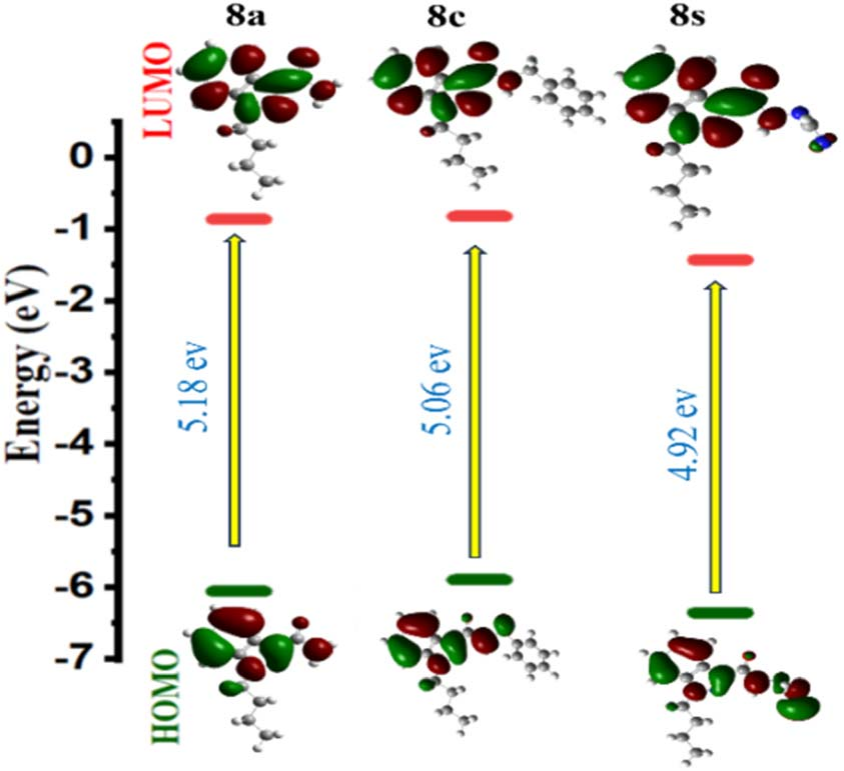
FMO diagrams of indazole derivatives (8a, 8c and 8s).

The energy gap was computed using the Δ*E* = (*E*_LUMO_ − *E*_HOMO_)^19^ formula, and Fig. 3 displays the FMO representation. It is discovered that compounds 8a, 8c, and 8q are good electron acceptors whereas compounds 8u, 8x, and 8z are good electron donors. The indazole molecule's HOMO and LUMO distributions span practically the entire molecule (see the ESI†). These descriptors provide insight into molecular behaviour, facilitating our understanding of chemical reactivity. The HOMO–LUMO energy gap makes it simple to calculate a molecule's chemical hardness, which is a good indicator of its reactivity. A higher degree of molecular softness is indicated by a smaller gap value, whereas a bigger HOMO–LUMO energy gap is indicative of higher molecular hardness. Fig. 3 displays the electron distribution of 8a, 8c and 8s under HOMO–LUMO. The red and green colours represent the positive (nucleophile) and negative (electrophilic) lobes respectively. The molecular electrostatic potential (MEP) is an essential tool that assists in predicting the reactivity sites of a molecule for nucleophilic and electrophilic attacks. Understanding hydrogen bonding and the relationship between a molecule's electrical characteristics, such as dipole moment, electronegativity, atomic charges, and so on, is also crucial for comprehending electrophilic and nucleophilic reactions. To compute the MEP surface analysis of a compound, Density Functional Theory (DFT) calculations are employed using the optimized structure and the B3LYP/6-31G(d,p) basis set. The MEP surface analysis provides extensive insights into a compound's properties and behaviour that are critical for scientific research and development.

**Fig. 4:**
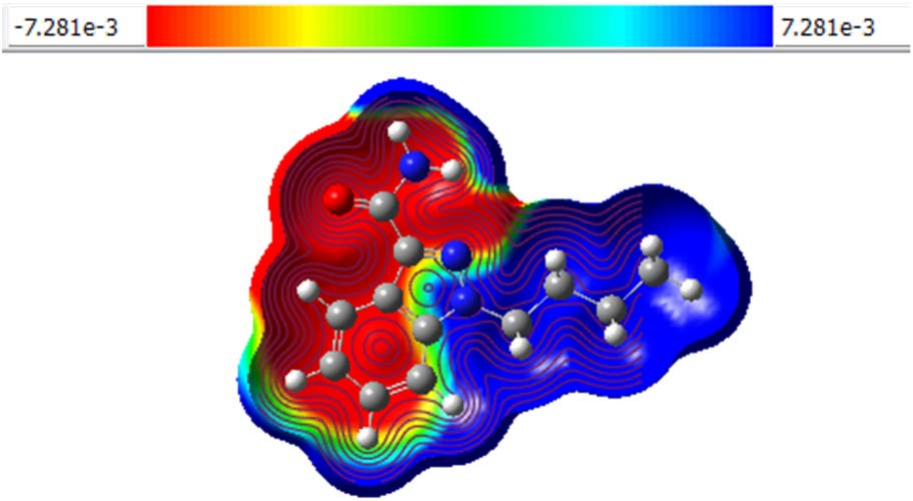
The molecular electrostatic potential surface of 8a.

MEP analysis clarifies the electron distribution pattern of compound 8a and its electron density (positive site: +7.281e, negative site: −7.281e), which are displayed in Fig. 4, which represents the compounds' mapped electrostatic potential surface. Electrostatic potential levels and zones of positive, negative, and neutral electrostatic potential are represented by different colours. Electrophilic sites are shown in red colour, whereas nucleophilic sites are represented in blue colour. An area with a neutral electrostatic potential is shown by the colour green. The oxygen and nitrogen atoms have electrophilic sites, while the hydrogen atoms have nucleophilic sites. Therefore, nucleophilic and electrophilic molecules are drawn to places with higher negative electronegative potential and positive electrostatic potential.^20–22^

## Molecular docking

The literature has identified ENTRECTINIB, PAZOPANIB, and AXITINIB as the three most commonly available medications with an indazole moiety that exhibit potent anti-renal cancer activity. To further augment the efficacy of these medications, we synthesized novel indazole derivatives and performed comprehensive molecular docking studies to evaluate their effectiveness against renal cancer. Through rigorous experimentation, we identified an enhanced PDB 6FEW^23^ that is directly linked to renal cancer and utilized the Auto Dock 4.2 software to perform molecular docking studies.

**Fig. 5:**
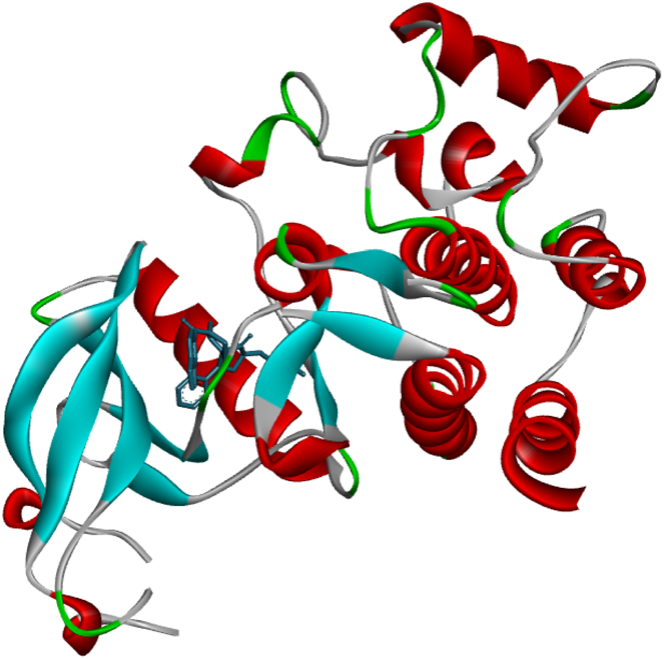
The crystal structure of DDR1, 2-[8-(1*H*-indazole-5-carbonyl)-4-oxo-1-phenyl-1,3,8-triazaspiro [4.5]decan-3-yl]-*N*-methyl acetamide (6FEW). 3D graphics were generated using Discovery Studio Visualizer 2021.

**Fig. 6:**
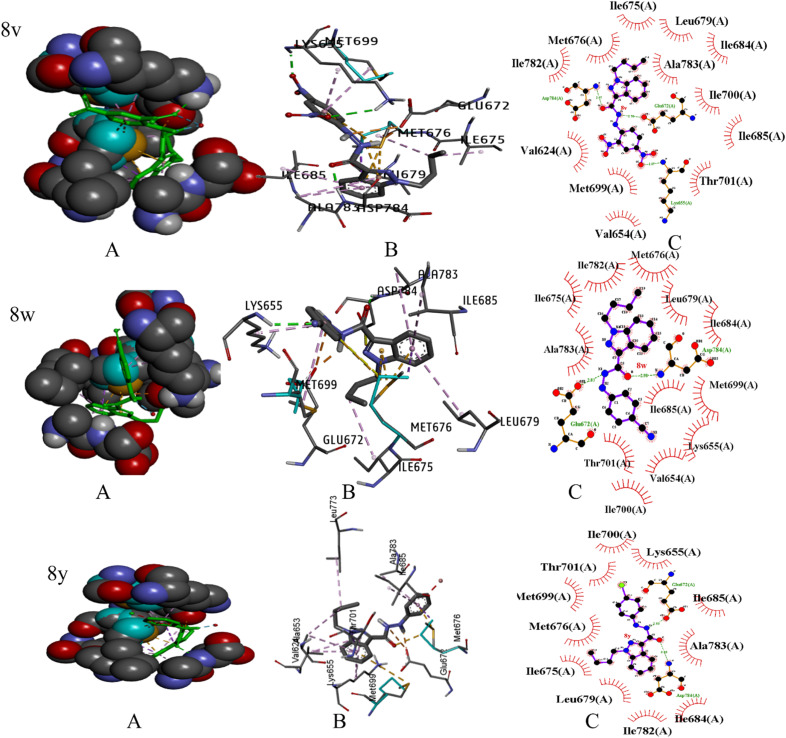
The binding pattern of indazole derivatives with PBD-6FEW. (A) Represents 3D surface representation, (B) represents the active ligand catalytic centre of the protein target, and (C) 2D-schematic LigPlot interactions shown for the docked pose of indazole derivatives shown by the spokes.

The crystal structure of DDR1, specifically 2-[8-(1*H*-indazole-5-carbonyl)-4-oxo-1-phenyl-1,3,8-triazaspiro[4.5]decan-3-yl], demonstrated exceptional efficacy against renal cancer (Fig. 5). The binding energy values (in kcal mol^−1^) of the ligand–receptor complex were utilized to rank the docking outcomes.^24^ The 2D structures of all 26 derivatives were prepared using the ChemOffice tool “Chem Draw 16.0,” which was used to show the proper 2D orientation of the chemical structures of the chosen ligands. ChemBio3D was then used to lower the energy of each molecule to improve geometry estimation and ligand–receptor affinity.^25^ To generate the protein, we employed Discovery Studio Visualizer for a better receptor file, and the auto-prepared file was used in an auto docking station to prepare targeted indazole molecules. The Discovery Studio 2021 application LigPlot v.2.2.8 was utilized to meticulously analyze the docking results, and the outcomes are presented in this report. The 2D structures vividly illustrate the interactions between the ligand and amino acid residues, while the 3D structures reveal the precise location of the ligand in the receptor's active site.^26^ We selected the molecules with the least amount of energy for a docking simulation in AutoDock Vina. The docking simulation grid box was given its configuration and the dimensions of *x*, *y*, and *z* is 40. The macromolecule's target area was placed inside the grid in such a way that it covered the whole structure. It was determined that the ligand and protein could be docked most effectively by utilizing the docking technique made accessible by Auto Dock Vina. During the docking approach, a maximum of ten conformations of each ligand were investigated. The use of a flexible sidechain to simulate the covalent ligand produced good results. All ten docking runs produced comparable conformations, all of which were quite similar to the crystallographic conformation. The 3D and 2D ligand–receptor interactions were investigated using the Discovery studio visualizer. We completed docking studies for 26 molecules and among the 26 molecules three compounds showed more active and binding energy those are 8v, 8w, and 8y (Table 2). Three compounds interact with amino acid residues involved in water hydrogen bonding, and covalent hydrogen bonding, Alkyl 2D interactions graphics using LigPlot (v.2.2.8) visualizer (Fig. 6).

**Table 2:** Docking results in terms of binding energy (kcal mol^−1^)

Compound	Δ*G*_binding energy_ (kcal mol^−1^)	*K*_i_ (micromolar) [temperature = 298.15 K]	H bond energy (kcal mol^−1^)
8v	−11.77	0.0235	−13.47
8w	−11.64	0.0294	−13.33
8y	−11.52	0.0361	−13.21

The compounds: 8v, 8w, and 8y ligand are interactions with ASP784, LYS655, MET699, GLU672, MET676, ILE685, LEU679, ALA783, ILE675, PHE762. *K*_i_ values (micromolar) calculated under room temperature (298 K) by utilizing auto dock software are 0.0235, 0.0295, and 0.0361, Our research group was finding the H bonding energy of all compounds (see in ESI†). We evaluated different approaches to docking covalently linked ligands: a grid-based technique and a flexible sidechain approach. Fig. 6 shows the docking results. Compounds 8v, 8w, and 8y interacted with amino acids through covalent bonds made up of hydrogen and alkyl, π–alkyl interaction with ASP784, LYS655, and ILE675, LEU679, ALA783, ILE685, MET699, HIS764. Water hydrogen bonds with HOH1193. These two derivatives formed pi–sulfur and pi–sigma interactions with GLU672 and MET676. Three substances are actively creating a salt bridge and actively interacting with GLU672.

## Conclusion

In conclusion, a range of 3-carboxamide indazole derivatives was successfully and efficiently synthesized using amide coupling. The comprehensive characterization of the target compounds and the high yields attained validated the methodologies used. Using DFT analysis, we determined the geometrical optimization of all the derivatives and discovered that 8a, 8c, and 8s had high energy gaps. We then used Auto Dock 4.0 to perform auto docking to determine the effectiveness of the indazole molecule in renal cancer and discovered that 8v, 8w, and 8y had the highest binding energy.

## Experimental

All the reactions were carried out in round bottom flasks. All the solvents and chemical materials were purchased from commercial sources. The 1-butyl-1*H*-indazole-3-carboxamide was prepared according to the reported protocols. ^1^H and ^13^C NMR spectra were recorded on Bruker Avance400 spectrometer and are referred to the residual solvent signal CDCl_3_: (7.26) for ^1^H and (77.16) for ^13^C NMR: dimethyl sulfoxide-d_6_ (2.50) for ^1^H and (39.50) for ^13^C NMR: chemical shift (*δ*) is given in ppm and coupling constant (*J*) were measured in Hz. The following abbreviations are used: s-singlet, d-doublet, dd-doublet of doublet, t-triplet, td-triplet of doublet, dt-doublet of triplet, q-quartet, qd-quartet of doublet, qn-quintet, br-broad, m-multiplet. HRMS ESI-MS was recorded using Xeo G2 XS OT of (water) and values are given *m*/*z*. Column chromatography was carried out using silica gel (100–200 mesh) packed in a glass column. Analytical TLC was carried out on Macherey-Nagel 60 F245 aluminium-backed silica gel plates.

### General procedure for the synthesis 8a–8z

To a stirred solution of 1-butyl-1*H*-indazole-3-carboxylic acid (250 mg, 1.146 mmol) was dissolved in DMF (10 mL), HATU (2 equivalents) and DIPEA (3 equivalents) were added to the reaction mixture, then commercial amines (2 equivalents) were added. The reaction mixture was stirred at room temperature for 8–16 h. After completion of the reaction, the resultant reaction mixture was poured into water, the solution was extracted with water and ethyl acetate (4 × 20 mL). The organic layer was dried with anhydrous sodium sulphate and the solvent was removed under reduced pressure to afford crude product. The crude was purified by silica gel chromatography to obtain pure products 8a–8z.

### Characterization of indazole carboxamide derivatives from 8a–8z

#### 1-Butyl-1*H*-indazole-3-carboxamide (8a)

Appearance: off-white solid (melting point: 86 °C, yield = 93.9%, 140 mg). ^1^H NMR [400 MHz, DMSO-d_6_]: *δ* 8.11 (d, *J* = 8.00 Hz, 1H), 7.69 (d, *J* = 8.80 Hz, 1H), 7.56 (s, 1H), 7.37 (t, *J* = 8.00 Hz, 1H), 7.28 (s, 1H), 7.19 (t, *J* = 7.60 Hz, 1H), 4.40 (t, *J* = 6.80 Hz, 2H), 1.79 (q, *J* = 6.80 Hz, 2H), 1.15–1.17 (m, 2H), 0.82 (t, *J* = 7.60 Hz, 3H). ^13^C NMR [100 MHz, DMSO-d_6_]: d ppm 141.04, 137.5, 126.84, 122.68, 122.64, 122.41, 110.71, 48.80, 31.88, 19.87, 13.96. IR streching: (N–H) = 3306 cm^−1^, (C=O) = 1664 cm^−1^. HRMS (ESI) *m*/*z*: [M + H] calculated C_12_H_15_N_3_O 217.1215 + found 240.111 [M + Na].

#### 1-Butyl-*N*-phenyl-1*H*-indazole-3-carboxamide (8b)

Appearance: pale brown solid (melting point: 88 °C, yield = 96.7%, 195 mg). ^1^H NMR [400 MHz, DMSO-d_6_]: *δ* 10.19 (s, 1H), 8.24 (d, *J* = 8.00 Hz, 1H), 7.89 (d, *J* = 8.00 Hz, 2H), 7.82 (d, *J* = 8.80 Hz, 1H), 7.49 (t, *J* = 7.20 Hz, 1H), 7.38–7.36 (m, 3H), 7.10 (q, *J* = 6.80 Hz, 1H), 4.56 (t, *J* = 6.80 Hz, 2H), 1.91 (q, *J* = 6.80 Hz, 2H), 1.34–1.32 (m, 2H), 0.92 (t, *J* = 7.20 Hz, 3H). ^13^C NMR [100 MHz, DMSO-d_6_]: d ppm 161.04, 141.17, 139.21, 137.46, 129.01, 127.17, 123.92, 123.05, 122.25, 120.78, 110.96, 49.08, 32.0, 19.94, 14.02. IR streching: (N–H) = 3306 cm^−1^, (C=O) = 1664 cm^−1^. HRMS (ESI) *m*/*z*: [M + H] calculated C_18_H_19_N_3_O 293.1528 + found 294.1596 [M + H].

#### *N*-Benzyl-1-butyl-1*H*-indazole-3-carboxamide (8c)

Appearance: pale yellow solid (melting point: 96 °C, yield = 90%, 190 mg). ^1^H NMR [400 MHz, DMSO-d_6_]: *δ* 8.81 (t, *J* = 6.40 Hz, 1H), 8.11 (d, *J* = 8.40 Hz, 1H), 7.70 (d, *J* = 8.80 Hz, 1H), 7.38 (t, *J* = 0.80 Hz, 1H), 7.19–7.21 (m, 4H), 7.15–7.16 (m, 2H), 4.40–4.41 (m, 4H), 1.79 (q, *J* = 7.20 Hz, 2H), 1.16–1.16 (m, 2H), 0.82 (t, *J* = 7.60 Hz, 3H). ^13^C NMR [100 MHz, DMSO-d_6_]: d ppm 162.4, 141, 140.42, 137.36, 128.69, 127.89, 127.15, 126.97, 122.74, 122.63, 122.27, 110.8, 48.88, 42.39, 31.93, 19.91, 14. IR streching: (N–H) = 3381 cm^−1^, (C=O) = 1639 cm^−1^. HRMS (ESI) *m*/*z*: [M + H] calculated C_19_H_21_N_3_O 307.17 + found 308.1760 [M + H].

#### *N*-(3-Bromophenyl)-1-butyl-1*H*-indazole-3-carboxamide (8d)

Appearance: brown solid (melting point: 99 °C, yield = 78.4%, 200 mg). ^1^H NMR [400 MHz, CDCl_3_]: *δ* 8.78 (s, 1H), 8.32 (d, *J* = 8.40 Hz, 1H), 7.95 (d, *J* = 1.60 Hz, 1H), 7.60–7.59 (m, 1H), 7.37–7.36 (m, 2H), 7.16 (t, *J* = 7.20 Hz, 2H), 4.34 (t, *J* = 7.20 Hz, 2H), 1.87 (q, *J* = 7.60 Hz, 2H), 1.34–1.32 (m, 2H), 0.89 (t, *J* = 7.20 Hz, 3H). ^13^C NMR [100 MHz, CDCl_3_]: 160.51, 141.11, 139.38, 136.73, 126.99, 126.83, 123.03, 122.94, 122.73, 122.44, 118.03, 109.48, 49.38, 31.38, 20.07, 13.66. IR streching: (N–H) = 3272 cm^−1^, (C=O) = 1655 cm^−1^. HRMS (ESI) *m*/*z*: [M + H] calculated C_18_H_18_BrN_3_O 371.2660 + found 372.0707 [M + H].

#### 2-(1-Butyl-1*H*-indazole-3-carboxamido)-5-iodobenzoic acid (8e)

Appearance: yellow solid (melting point: 101 °C, yield = 47%, 150 mg). ^1^H NMR [400 MHz, DMSO-d_6_]: *δ* 8.46 (d, *J* = 8.00 Hz, 1H), 8.38 (s, 1H), 8.24 (d, *J* = 8.40 Hz, 1H), 7.88 (d, *J* = 8.40 Hz, 1H), 1887.17 (q, *J* = 8.40 Hz, 2H), 7.41 (t, *J* = 7.60 Hz, 1H), 4.58 (t, *J* = 6.40 Hz, 2H), 1.90 (t, *J* = 6.80 Hz, 2H), 1.29 (q, *J* = 6.80 Hz, 2H), 0.91 (t, *J* = 7.20 Hz, 3H). ^13^C NMR [100 MHz, DMSO-d_6_]: d ppm 157.97, 153.64, 146.45, 145.49, 141.21, 136.41, 134.02, 129.31, 127.51, 123.75, 122.91, 122.50, 119.65, 111.33, 93.54, 49.40, 31.93, 19.88, 13.98. IR streching: (O–H) = 2947 cm^−1^, (N–H) = 2860 cm^−1^, (C=O) = 1740 cm^−1^. HRMS (ESI) *m*/*z*: [M + H] calculated C_19_H_16_IN_3_O_2_ 445.0287 + found 446.0365 [M + H].

#### 1-Butyl-*N*-(4-(phenylamino)phenyl)-1*H*-indazole-3-carboxamide (8f)

Appearance: grey solid (melting point: 113 °C, yield = 87%, 230 mg). ^1^H NMR [400 MHz, DMSO-d_6_]: *δ* 10.06 (s, 1H), 8.26 (d, *J* = 8.00 Hz, 1H), 8.08 (s, 1H), 7.78 (t, *J* = 7.20 Hz, 3H), 7.48 (t, *J* = 7.20 Hz, 1H), 7.31 (t, *J* = 7.60 Hz, 1H), 7.22 (t, *J* = 7.20 Hz, 2H), 7.08 (q, *J* = 8.40 Hz, 4H), 6.79 (t, *J* = 7.20 Hz, 1H), 4.53 (t, *J* = 6.80 Hz, 2H), 1.90 (t, *J* = 6.80 Hz, 2H), 1.30 (q, *J* = 7.20 Hz, 2H), 0.90 (t, *J* = 7.20 Hz, 3H). ^13^C NMR [100 MHz, DMSO-d_6_]: d ppm 160.64, 144.51, 141.16, 139.64, 137.71, 131.98, 129.62, 127.12, 122.91, 122.88, 122.33, 122.15, 119.55, 118.03, 116.43, 110.89, 49.02, 31.98, 19.93, 14. IR streching: (N–H) = 3342 cm^−1^, (C=O) = 1635 cm^−1^. HRMS (ESI) *m*/*z*: [M + H] calculated C_24_H_24_N_4_O 384.4830 + found 384.1970 [M + H].

#### Ethyl 4-(1-butyl-1*H*-indazole-3-carboxamido) benzoate (8g)

Appearance: off white solid (melting point: 120 °C, yield = 75.6%, 190 mg). ^1^H NMR [400 MHz, DMSO-d_6_]: *δ* 10.58 (s, 1H), 8.24 (d, *J* = 8.00 Hz, 1H), 8.08 (d, *J* = 8.80 Hz, 2H), 7.96 (d, *J* = 8.40 Hz, 2H), 7.84 (d, *J* = 8.40 Hz, 1H), 7.51 (t, *J* = 8.00 Hz, 1H), 7.34 (t, *J* = 7.20 Hz, 2H), 4.57 (t, *J* = 6.80 Hz, 2H), 4.31 (q, *J* = 6.80 Hz, 2H), 1.91 (q, *J* = 7.20 Hz, 2H), 1.26–1.27 (m, 2H), 0.00 (t, *J* = 7.20 Hz, 3H). ^13^C NMR [100 MHz, DMSO-d_6_]: d ppm 165.86, 161.38, 143.72, 141.22, 137.08, 130.47, 127.30, 124.86, 123.32, 122.96, 122.12, 120.06, 111.12, 60.90, 49.16, 31.97, 19.92, 14.70, 14.01. IR streching: (N–H) = 3383 cm^−1^, (C=O) = 1712 cm^−1^. HRMS (ESI) *m*/*z*: [M + H] calculated C_21_H_23_N_3_O_3_ 365.4330 + found 366.1817 [M + H].

#### 1-Butyl-*N*-(4-nitrophenyl)-1*H*-indazole-3-carbohydrazide (8h)

Appearance: pale brown solid (melting point: 92 °C, yield = 57.8%, 140.5 mg). ^1^H NMR [400 MHz, DMSO-d_6_]: *δ* 10.60 (s, 1H), 9.22 (s, 1H), 8.11 (q, *J* = 9.20 Hz, 3H), 7.83 (d, *J* = 8.40 Hz, 1H), 7.49 (t, *J* = 7.60 Hz, 1H), 7.30 (t, *J* = 7.20 Hz, 1H), 6.83 (d, *J* = 9.20 Hz, 1H), 4.54 (t, *J* = 6.80 Hz, 2H), 1.91 (q, *J* = 7.20 Hz, 2H), 1.27–1.29 (m, 2H), 0.92 (t, *J* = 7.20 Hz, 3H). ^13^C NMR [100 MHz, DMSO-d_6_]: d ppm 162.31, 155, 67, 140.87, 138.40, 135.72, 127.21, 126.41, 123.15, 122.96, 121.89, 111.11, 111.02, 49.08, 31.91, 19.92, 14.02. IR streching: (N–H) = 3355 cm^−1^, (C=O) = 1658 cm^−1^, (NO_2_) = 1595 cm^−1^. HRMS (ESI) *m*/*z*: [M + H] calculated C_18_H_19_N_5_O_3_ 353.3820 + found 354.1569 [M + H].

#### *N*-(4-Bromophenyl)-1-butyl-1*H*-indazole-3-carboxamide (8i)

Appearance: brown solid (melting point: 99 °C, yield = 82.5%, 210.6 mg). ^1^H NMR [400 MHz, DMSO-d_6_]: *δ* 10.41 (s, 1H), 8.23 (d, *J* = 8.40 Hz, 1H), 7.90 (d, *J* = 8.80 Hz, 2H), 7.83 (d, *J* = 8.40 Hz, 1H), 7.55–7.52 (m, 3H), 7.32 (t, *J* = 7.60 Hz, 1H), 4.55 (t, *J* = 7.20 Hz, 2H), 1.90 (q, *J* = 7.20 Hz, 2H), 1.34–1.32 (m, 2H), 0.91 (t, *J* = 7.20 Hz, 3H). ^13^C NMR [100 MHz, DMSO-d_6_]: d ppm 161.16, 141.18, 138.68, 137.23, 131.82, 127.24, 123.18, 122.74, 122.18, 111.04, 55.37, 31.99, 19.92, 14.01. IR streching: (N–H) = 3272 cm^−1^, (C=O) = 1655 cm^−1^. HRMS (ESI) *m*/*z*: [M + H] calculated C_18_H_18_BrN_3_O 371.2660 + found 372.0707 [M + H].

#### 1-Butyl-*N*-(4-hydroxyphenyl)-1*H*-indazole-3-carboxamide (8j)

Appearance: off white solid (melting point: 101 °C, yield = 72.2%, 153 mg). ^1^H NMR [400 MHz, DMSO-d_6_]: *δ* 9.97 (s, 1H), 9.24 (s, 1H), 8.22 (d, *J* = 8.00 Hz, 1H), 7.80 (d, *J* = 8.40 Hz, 1H), 7.64 (dd, *J* = 1.60, 8.60 Hz, 2H), 7.48 (t, *J* = 7.60 Hz, 1H), 7.30 (t, *J* = 8.00 Hz, 1H), 6.75 (d, *J* = 8.40 Hz, 2H), 4.53 (t, *J* = 6.80 Hz, 2H), 1.90 (q, *J* = 6.80 Hz, 2H), 1.28 (q, *J* = 7.60 Hz, 2H), 0.91 (t, *J* = 7.20 Hz, 3H). ^13^C NMR [100 MHz, DMSO-d_6_]: d ppm 160.58, 15.06, 141.13, 137.71, 130.75, 127.09, 122.86, 122.61, 122.31, 115.42, 110.88, 48.98, 31.98, 19.92, 14.01. IR streching: (N–H) = 3323 cm^−1^, (C=O) = 1651 cm^−1^. HRMS (ESI) *m*/*z*: [M + H] calculated C_18_H_19_N_3_O_2_ 309.3690 + found 310.1553 [M + H].

#### 1-Butyl-*N*-(2-hydroxy-6methylphenyl)-1*H*-indazole-3-carboxamide (8k)

Appearance: pale brown solid (melting point: 89 °C, yield = 13.5%, 30 mg). ^1^H NMR [400 MHz, DMSO-d_6_]: *δ* 9.34 (s, 1H), 9.22 (s, 1H), 8.18 (d, *J* = 8.00 Hz, 1H), 7.81 (d, *J* = 8.40 Hz, 1H), 7.48 (t, *J* = 8.00 Hz, 1H), 7.29 (t, *J* = 7.20 Hz, 1H), 7.02 (t, *J* = 8.00 Hz, 1H), 6.75 (q, *J* = 7.20 Hz, 2H), 4.54 (t, *J* = 7.20 Hz, 2H), 2.20 (s, 3H), 1.91 (q, *J* = 7.20 Hz, 2H), 1.28–1.30 (m, 2H), 0.94 (t, *J* = 5.60 Hz, 3H). ^13^C NMR [100 MHz, DMSO-d_6_]: d ppm 161.17, 153.47 141.08, 137.35, 137.17, 127.37, 127.05, 124.00, 122.87, 122.79, 122.28, 121.00, 113.95, 110.88, 48.98, 31.42, 22.53, 14.42, 14.01. IR streching: (N–H) = 3355 cm^−1^, (OH) = 3104 cm^−1^, (C=O) = 1643 cm^−1^. HRMS (ESI) *m*/*z*: [M + H] calculated C_19_H_21_N_3_O_2_ 323.3960 + found 324.1711 [M + H].

#### 1-Butyl-*N*-(*m*-tolyl)-1*H*-indazole-3-carboxamide (8l)

Appearance: off white solid (melting point: 130 °C, yield = 90.2%, 190 mg). ^1^H NMR [400 MHz, DMSO-d_6_]: *δ* 10.07 (s, 1H), 8.23 (d, *J* = 8.00 Hz, 1H), 7.81 (d, *J* = 8.40 Hz, 1H), 7.76 (s, 1H), 7.67 (d, *J* = 8.00 Hz, 1H), 7.49 (t, *J* = 8.00 Hz, 1H), 7.32 (t, *J* = 7.60 Hz, 1H), 7.23 (t, *J* = 8.00 Hz, 1H), 6.92 (d, *J* = 7.60 Hz, 1H), 4.54 (t, *J* = 7.20 Hz, 2H), 2.33 (s, 3H), 1.91 (q, *J* = 7.20 Hz, 2H), 1.30 (q, *J* = 7.60 Hz, 2H), 0.91 (t, *J* = 7.20 Hz, 3H). ^13^C NMR [100 MHz, DMSO-d_6_]: d ppm 160.97, 141.18, 139.11, 138.19, 137.47, 128.87, 127.16, 124.64, 123.04, 122.22, 121.26, 117.91, 110.95, 49.05, 31.96, 21.70, 19.93, 14. IR streching: (N–H) = 3316 cm^−1^, (C=O) = 1659 cm^−1^. HRMS (ESI) *m*/*z*: [M + H] calculated C_19_H_21_N_3_O 307.3970 + found 308.1765 [M + H].

#### 1-Butyl-*N*-(*o*-tolyl)-1*H*-indazole-3-carboxamide (8m)

Appearance: off white solid (melting point: 130 °C, yield = 90.2%, 190.5 mg). ^1^H NMR [400 MHz, DMSO-d_6_]: *δ* 9.61 (s, 1H), 8.24–8.22 (m, 3H), 7.83 (d, *J* = 8.40 Hz, 1H), 7.69 (dd, *J* = 2.40, −8.40 Hz, 1H), 7.50 (td, *J* = 0.80, −7.00 Hz, 1H), 7.34 (td, *J* = 0.40, −11.20 Hz, 1H), 4.53 (t, *J* = 6.80 Hz, 2H), 2.28 (s, 3H), 1.89 (q, *J* = 7.20 Hz, 2H), 1.28 (q, *J* = 7.60 Hz, 2H), 0.89 (t, *J* = 7.60 Hz, 3H). ^13^C NMR [100 MHz, DMSO-d_6_]: d ppm 160.42, 149.27, 148.46, 141.42, 139.25, 136.37, 129.24, 127.37, 123.49, 122.55, 121.91, 113.34, 111.20, 49.31, 31.77, 19.86, 17.76, 13.93. IR streching: (N–H) = 3391 cm^−1^, (C=O) = 1679 cm^−1^. HRMS (ESI) *m*/*z*: [M + H] calculated C_19_H_21_N_3_O 307.3970 + found 308.1721 [M + H].

#### 1-Butyl-*N*-(5-methylpyridin-2-yl)-1*H*-indazole-3-carboxamide (8n)

Appearance: off white solid (melting point: 142 °C, yield = 87.5%, 185.5 mg). ^1^H NMR [400 MHz, DMSO-d_6_]: *δ* 9.65 (s, 1H), 8.23 (d, *J* = 8.00 Hz, 1H), 7.81 (d, *J* = 8.40 Hz, 1H), 7.69 (d, *J* = 7.60 Hz, 1H), 7.49 (t, *J* = 7.20 Hz, 1H), 7.33–7.31 (m, 3H), 7.13 (t, *J* = 7.60 Hz, 1H), 4.53 (t, *J* = 6.80 Hz, 2H), 2.31 (s, 3H), 1.90 (q, *J* = 7.20 Hz, 2H), 1.35–1.34 (m, 2H), 0.91 (t, *J* = 7.20 Hz, 3H). ^13^C NMR [100 MHz, DMSO-d_6_]: d ppm 160.80, 141.21, 137.26, 136.54, 132.05, 127.16, 125.62, 125.13, 123.05, 122.76, 122.19, 110.96, 48.98, 31.88, 19.9, 18.16, 13.95. IR streching: (N–H) = 3387 cm^−1^, (C=O) = 1675 cm^−1^. HRMS (ESI) *m*/*z*: [M + H] calculated C_19_H_20_N_4_O 308.3850 + found 309.1719 [M + H].

#### 1-Butyl-*N*-(5-methylpyridin-2-yl)-1*H*-indazole-3-carboxamide (8o)

Appearance: off white solid (melting point: 145 °C, yield = 89.1%, 198 mg). ^1^H NMR [400 MHz, DMSO-d_6_]: *δ* 10.11 (s, 1H), 8.24 (d, *J* = 8.00 Hz, 1H), 7.80 (d, *J* = 8.80 Hz, 3H), 7.48 (t, *J* = 7.60 Hz, 1H), 7.30 (t, *J* = 7.60 Hz, 1H), 6.94 (d, *J* = 8.80 Hz, 2H), 4.53 (t, *J* = 7.20 Hz, 2H), 3.76 (s, 3H), 1.90 (q, *J* = 7.20 Hz, 2H), 1.34–1.30 (m, 2H), 0.90 (t, *J* = 7.60 Hz, 3H). ^13^C NMR [100 MHz, DMSO-d_6_]: d ppm 160.73, 155.9, 141.14, 137.61, 132.31, 127.12, 122.93, 122.86, 122.37, 122.30, 114.17, 110.91, 55.63, 49.02, 31.99, 19.93, 14. IR streching: (N–H) = 3279 cm^−1^, (C=O) = 1651 cm^−1^. HRMS (ESI) *m*/*z*: [M + H] calculated C_19_H_21_N_3_O_2_ 323.3960 + found 324.1711 [M + H].

#### 1-Butyl-*N*-(4-fluorophenyl)-1*H*-indazole-3-carboxamide (8p)

Appearance: pale brown solid (melting point: 94 °C, yield = 84.3%, 180.5 mg). ^1^H NMR [400 MHz, DMSO-d_6_]: *δ* 10.34 (s, 1H), 8.25 (d, *J* = 8.00 Hz, 1H), 7.96–7.95 (m, 2H), 7.79 (d, *J* = 8.40 Hz, 1H), 7.48 (t, *J* = 7.20 Hz, 1H), 7.31 (t, *J* = 7.60 Hz, 1H), 7.20 (t, *J* = 9.20 Hz, 2H), 4.53 (t, *J* = 7.20 Hz, 2H), 1.89 (q, *J* = 7.20 Hz, 2H), 1.33–1.31 (m, 2H), 0.89 (t, *J* = 7.60 Hz, 3H). ^13^C NMR [100 MHz, DMSO-d_6_]: d ppm 161.04, 159.9, 141.16, 137.36, 127.16, 123.05, 122.93, 122.7, 122.62, 122.24, 115.65, 115.43, 110.93, 49.07, 31.98, 19.91, 13.96. IR streching: (N–H) = 3307 cm^−1^, (C=O) = 1663 cm^−1^. HRMS (ESI) *m*/*z*: [M + H] calculated C_18_H_18_FN_3_O 311.3604 + found 312.1508 [M + H].

#### 1-Butyl-*N*-(2-methoxyphenyl)-1*H*-indazole-3-carboxamide (8q)

Appearance: off white solid (melting point: 142 °C, yield = 79.8%, 177.5 mg). ^1^H NMR [400 MHz, DMSO-d_6_]: *δ* 9.44 (s, 1H), 8.40 (dd, *J* = 7.60, Hz, 1H), 8.26 (d, *J* = 8.00 Hz, 1H), 7.83 (d, *J* = 8.80 Hz, 1H), 7.52–7.52 (m, 1H), 7.34 (t, *J* = 7.60 Hz, 1H), 7.15–7.14 (m, 2H), 4.54 (t, *J* = 6.80 Hz, 2H), 3.94 (s, 3H), 1.88 (q, *J* = 7.20 Hz, 2H), 1.34–1.32 (m, 2H), 0.91 (t, *J* = 7.20 Hz, 3H). ^13^C NMR [100 MHz, DMSO-d_6_]: d ppm 160.06, 148.68, 141.39, 136.9, 127.55, 127.37, 124.35, 123.41, 122.47, 122.07, 121.13, 119.71, 111.41, 111.17, 56.53, 48.99, 31.8, 19.84, 13.88. IR streching: (N–H) = 3307 cm^−1^, (C=O) = 1663 cm^−1^. HRMS (ESI) *m*/*z*: [M + H] calculated C_19_H_21_N_3_O_2_ 323.3960 + found 324.1713 [M + H].

#### 1-Butyl-*N*-(3-hydroxyphenyl)-1*H*-indazole-3-carboxamide (8r)

Appearance: pale brown solid (melting point: 101 °C, yield = 66%, 140 mg). ^1^H NMR [400 MHz, DMSO-d_6_]: *δ* 10.05 (s, 1H), 9.43 (s, 1H), 8.23 (d, *J* = 8.40 Hz, 1H), 7.80 (d, *J* = 8.40 Hz, 1H), 7.50–7.50 (m, 2H), 7.31 (t, *J* = 7.60 Hz, 1H), 7.26 (d, *J* = 8.40 Hz, 1H), 7.13 (t, *J* = 8.40 Hz, 1H), 6.52 (dd, *J* = 1.60, 8.00 Hz, 1H), 4.53 (t, *J* = 6.80 Hz, 2H), 1.89 (q, *J* = 2.80 Hz, 2H), 1.33–1.31 (m, 2H), 0.90 (t, *J* = 7.20 Hz, 3H). ^13^C NMR [100 MHz, DMSO-d_6_]: d ppm 160.95, 157.99, 141.16, 140.2, 137.53, 129.67, 127.18, 123.04, 122.86, 122.23, 111.56, 111.12, 110.95, 107.88, 49.04, 31.96, 19.91, 13.99. IR streching: (N–H) = 3347 cm^−1^, (OH) = 3200 cm^−1^, (C=O) = 1703 cm^−1^. HRMS (ESI) *m*/*z*: [M + H] calculated C_18_H_19_N_3_O_2_ 309.3690 + found 310.1554 [M + H].

#### 1-Butyl-*N*-(4H-1,2,4-triazol-4yl)-1*H*-indazole-3-carboxamide (8s)

Appearance: off white solid (melting point: 98 °C, yield = 89%, 174 mg). ^1^H NMR [400 MHz, DMSO-d_6_]: *δ* 12.12 (s, 1H), 8.80 (s, 2H), 8.14 (d, *J* = 8.00 Hz, 1H), 7.87 (d, *J* = 8.40 Hz, 1H), 7.52 (t, *J* = 7.20 Hz, 1H), 7.35 (t, *J* = 7.60 Hz, 1H), 4.57 (t, *J* = 7.20 Hz, 2H), 1.91 (q, *J* = 6.80 Hz, 2H), 1.32–1.31 (m, 2H), 0.91 (t, *J* = 7.20 Hz, 3H). ^13^C NMR [100 MHz, DMSO-d_6_]: d ppm 161.53, 144.49, 140.98, 134.46, 127.48, 123.73, 123.01, 121.58, 111.27, 49.26, 31.89, 19.87, 13.99. IR streching: (N–H) = 3112 cm^−1^, (C=O) = 1695 cm^−1^. HRMS (ESI) *m*/*z*: [M + H] calculated C_14_H_16_N_6_O 284.3230 + found 285.1460 [M + H].

#### *N*-(2-Amino-4-nitrophenyl)-1butyl-1*H*-indazole-3-carbohydrazide (8t)

Appearance: brown solid (melting point: 115 °C, yield = 84.4%, 205 mg). ^1^H NMR [400 MHz, DMSO-d_6_]: *δ* 9.75 (s, 1H), 8.25 (s, 1H), 8.20 (d, *J* = 8.40 Hz, 1H), 7.93 (dd, *J* = 2.80, 9.00 Hz, 1H), 7.83 (d, *J* = 8.80 Hz, 1H), 7.49 (t, *J* = 8.00 Hz, 1H), 7.32 (t, *J* = 7.20 Hz, 1H), 6.85 (t, *J* = 8.80 Hz, 1H), 6.56 (s, 2H), 4.55 (t, *J* = 7.20 Hz, 2H), 1.92 (q, *J* = 7.20 Hz, 2H), 1.28–1.30 (m, 2H), 0.92 (t, *J* = 7.60 Hz, 3H). ^13^C NMR [100 MHz, DMSO-d_6_]: d ppm 161.70, 150.93, 141.11, 137.06, 136.05, 127.15, 123.77, 123.56, 123.07, 122.94, 121.19, 121.73, 114.58, 110.95, 49.07, 32.00, 19.95, 14.03. IR streching: (N–H) = 3459 cm^−1^, (N–H) = 3327 cm^−1^, (C=O) = 1669 cm^−1^, (NO_2_) = 1623 cm^−1^. HRMS (ESI) *m*/*z*: [M + H] calculated C_18_H_19_N_5_O_3_ 353.3820 + found 354.1563 [M + H].

#### 1-Butyl-*N*-phenyl-1*H*-indazole-3-carbohydrazide (8u)

Appearance: off white solid (melting point: 100 °C, yield = 99%, 140 mg). ^1^H NMR [400 MHz, DMSO-d_6_]: *δ* 10.14 (d, *J* = 2.40 Hz, 1H), 8.05 (d, *J* = 8.00 Hz, 1H), 7.82 (d, *J* = 2.80 Hz, 1H), 7.73 (d, *J* = 8.40 Hz, 1H), 7.40 (dt, *J* = 0.80, 10.80 Hz, 1H), 7.21 (t, *J* = 7.60 Hz, 1H), 7.08 (t, *J* = 8.00 Hz, 2H), 6.73 (d, *J* = 7.60 Hz, 2H), 6.64 (t, *J* = 7.20 Hz, 1H), 4.46 (t, *J* = 9.20 Hz, 2H), 1.83 (q, *J* = 7.20 Hz, 2H), 1.28–1.26 (m, 2H), 0.84 (t, *J* = 7.20 Hz, 3H). ^13^C NMR [100 MHz, DMSO-d_6_]: d ppm 162.52, 150.06, 140.81, 136.30, 129.14, 127.07, 122.88, 122.86, 121.97, 118.88, 112.71, 110.89, 48.95, 31.90, 19.92, 14.02. IR streching: (N–H) = 3233 cm^−1^, (C=O) = 1645 cm^−1^. HRMS (ESI) *m*/*z*: [M + H] calculated C_18_H_20_N_4_O 308.3850 + found 331.1558 [M + Na].

#### 1-Butyl-*N*-(2,4-dinitrophenyl)-1*H*-indazole-3-carbohydrazide (8v)

Appearance: pale brown solid (melting point: 133 °C, yield = 18.3%, 50 mg). ^1^H NMR [400 MHz, DMSO-d_6_]: *δ* 11.03 (s, 1H), 10.30 (s, 1H), 8.91 (s, 1H), 8.34 (d, *J* = 8.40 Hz, 1H), 8.14 (d, *J* = 7.20 Hz, 1H), 7.86 (d, *J* = 7.60 Hz, 1H), 7.51 (s, 1H), 7.32 (s, 2H), 4.56 (s, 2H), 1.92 (s, 2H), 1.32 (d, *J* = 6.00 Hz, 2H), 0.926 (s, 3H). ^13^C NMR [100 MHz, DMSO-d_6_]: d ppm 161.89, 149.34, 140.91, 137.14, 135.37, 130.64, 127.31, 123.64, 123.36, 123.02, 121.82, 116.22, 111.10, 49.14, 31.93, 19.91, 14.02. IR streching: (N–H) = 3363 cm^−1^, (N–H) = 3331 cm^−1^, (C=O) = 1691 cm^−1^. HRMS (ESI) *m*/*z*: [M + H] calculated C_18_H_18_N_6_O_5_ 398.3790 + found 399.1414 [M + H].

#### 1-Butyl-*N*-(4-cyanophenyl)-1*H*-indazole-3-carbohydrazide (8w)

Appearance: brown solid (melting point: 148 °C, yield = 87.3%, 200 mg). ^1^H NMR [400 MHz, DMSO-d_6_]: *δ* 10.46 (s, 1H), 8.79 (s, 1H), 8.12 (d, *J* = 8.00 Hz, 1H), 7.82 (d, *J* = 8.40 Hz, 1H), 7.56 (d, *J* = 8.40 Hz, 2H), 7.48 (t, *J* = 7.60 Hz, 1H), 7.29 (t, *J* = 7.60 Hz, 1H), 6.83 (d, *J* = 8.40 Hz, 2H), 4.53 (t, *J* = 7.20 Hz, 2H), 1.90 (q, *J* = 7.20 Hz, 2H), 1.26–1.28 (m, 2H), 0.00 (t, *J* = 7.60 Hz, 3H). ^13^C NMR [100 MHz, DMSO-d_6_]: d ppm 162.39, 153.62, 140.84, 135.87, 133.85, 127.16, 123.07, 122.93, 121.9, 120.58, 112.19, 99.15, 49.04, 31.90, 19.92, 14.01. IR streching: (N–H) = 3343 cm^−1^, (N–H) = 3235 cm^−1^, (CN) = 2220 cm^−1^, (C=O) = 1679 cm^−1^. HRMS (ESI) *m*/*z*: [M + H] calculated C_19_H_19_N_5_O 333.3950 + found 334.1668 [M + H].

#### 1-Butyl-*N*-(4-hydroxyphenyl)-1*H*-indazole-3-carbohydrazide (8x)

Appearance: pale brown solid (melting point: 155 °C, yield = 60.5%, 135 mg). ^1^H NMR [400 MHz, DMSO-d_6_]: *δ* 10.19 (d, *J* = 9.60 Hz, 2H), 10.09 (s, 1H), 8.14 (d, *J* = 8.40 Hz, 3H), 7.82 (d, *J* = 8.40 Hz, 1H), 7.47 (t, *J* = 0.40 Hz, 1H), 7.30 (t, *J* = 7.20 Hz, 1H), 6.86 (d, *J* = 8.40 Hz, 2H), 4.52 (t, *J* = 7.20 Hz, 2H), 1.90 (q, *J* = 6.80 Hz, 2H), 1.26–1.26 (m, 2H), 0.91 (t, *J* = 7.20 Hz, 3H). ^13^C NMR [100 MHz, DMSO-d_6_]: d ppm 165.93, 161.90, 161.07, 140.84, 136.07, 129.98, 1247.09, 123.78, 122.99, 122.93, 121.96, 115.44, 110.93, 48.96, 31.89, 19.90, 14.00. IR streching: (N–H) = 3359 cm^−1^, (OH) = 3204 cm^−1^, (C=O) = 1679 cm^−1^. HRMS (ESI) *m*/*z*: [M + H] calculated C_19_H_20_N_4_O_3_ 352.3940 + found 353.1595 [M + H].

#### *N*-(4-Bromophenyl)-1butyl-1*H*-indazole-3-carbohydrazide (8y)

Appearance: brown solid (melting point: 153 °C, yield = 75.3%, 200 mg). ^1^H NMR [400 MHz, DMSO-d_6_]: 10.28 (s, 1H), 8.12 (s, 2H), 7.81 (d, *J* = 8.40 Hz, 1H), 7.47 (t, *J* = 8.00 Hz, 1H), 7.29 (t, *J* = 11.60 Hz, 3H), 6.74 (d, *J* = 8.40 Hz, 2H), 4.52 (t, *J* = 6.80 Hz, 2H), 1.90 (q, *J* = 6.80 Hz, 2H), 1.24–1.26 (m, 2H), 0.91 (t, *J* = 7.20 Hz, 3H). ^13^C NMR [100 MHz, DMSO-d_6_]: d ppm 162.48, 149.45, 140.81, 136.12, 131.77, 127.10, 122.95, 121.93, 114.69, 110.91, 109.60, 48.98, 31.89, 19.91, 14.01. IR streching: (N–H) = 3343, 3231 cm^−1^, (C=O) = 1658 cm^−1^ HRMS (ESI) *m*/*z*: [M + H] calculated C_18_H_19_BrN_4_O_2_ 386.2810 + found 409.0665 [M + Na].

#### 1-Butyl-*N*-(4-nitrophenyl)-1*H*-indazole-3-carboxamide (8z)

Appearance: yellow solid (melting point: 110 °C, yield = 25.7%, 40 mg). ^1^H NMR [400 MHz, DMSO-d_6_]: *δ* 10.88 (s, 1H), 8.26 (q, *J* = 9.20 Hz, 5H), 7.86 (d, *J* = 8.40 Hz, 1H), 7.52 (t, *J* = 7.60 Hz, 1H), 7.36 (t, *J* = 7.20 Hz, 1H), 4.58 (t, *J* = 6.80 Hz, 2H), 1.92 (q, *J* = 7.60 Hz, 2H), 1.27–1.28 (m, 2H), 0.92 (t, *J* = 7.20 Hz, 3H), (t, *J* = Hz, 3H). ^13^C NMR [100 MHz, DMSO-d_6_]: d ppm 161.60, 145.67, 142.80, 141.25, 136.80, 127.39, 125.17, 123.04, 122.06, 120.39, 111.21, 49.25, 31.99, 19.92, 14.01. IR streching: (N–H) = 3383 cm^−1^, (C=O) = 1712 cm^−1^. HRMS (ESI) *m*/*z*: [M + H] calculated C_18_H_18_N_4_O_3_ 338.14 + found 361.1274 [M + Na].

## Conflicts of interest

There are no conflicts to declare.

## Supplementary Material

RA-014-D4RA02151G-s001

RA-014-D4RA02151G-s002
