# Supplementary material for: Synthesis molecular docking and DFT studies on novel indazole derivatives
Source: RSC Adv. 2024 Apr 23;14(19):13218–26. doi: 10.1039/d4ra02151g (PMC11037238; doi:10.1039/d4ra02151g)
Supplement: RA-014-D4RA02151G-s002 [file RA-014-D4RA02151G-s002.pdf]

Electronic Supplementary Material (ESI) for RSC Advances.
This journal is © The Royal Society of Chemistry 2024

Supplementary Data

Synthesis molecular docking and DFT studies on novel indazole derivatives

Bandaru Gopi[a], Vijayaparthasarathi Vijayakumar*

Department of Chemistry, School of Advanced Science, Vellore Institute of Technology, Vellore- 632014, India

Table of contents

1. General methods……………………………………………………….1-1
2. Scheme and Experimental procedure…………………………………..1-4
3. ^{1}H and ^{13}C NMR, Dept-135, COSY, HSQC, IR and HRMS spectra of the compounds……………………4-90
4. DFT studies indazole derivatives with HOMO and LUMO values and Docking results from (8a-8z) 3-carboxamide indazole derivatives…………………………………………..…………90-116

1. General methods

All the reactions were carried out in round bottom flasks. All the solvents and chemical materials were purchased from commercial sources. The 1-butyl-1H-indazole-3-carboxamide was prepared according to the reported protocols. ^{1}H and ^{13}C NMR spectra were recorded on Bruker Avance400 spectrometer and are referred to the residual solvent signal $CDCl_3$: (7.26) for ^{1}H and (77.16) for ^{13}C NMR: dimethyl sulfoxide-d_6 (2.50) for ^{1}H and (39.50) for ^{13}C NMR: chemical shift (δ) 3is given in ppm and coupling constant (J) were measured in Hz. The following abbreviations are used: s- singlet, d-doublet, dd-doublet of doublet, t-triplet, td-triplet of doublet, dt- doublet of triplet, q-quartet, qd- quartet of doublet, qn-quintet, br-broad, m-multiplet. HRMS ESI-MS was recorded using Xeo G2 XS OT of (water) and values are given m/z. Colum chromatography was carried out using silica gel (100-200 mesh) packed in a glass column. Analytical TLC was carried out on Macherey-Nagel 60 F245 aluminium-backed silica gel plates.

2. Scheme and Experimental procedure for indazole derivative

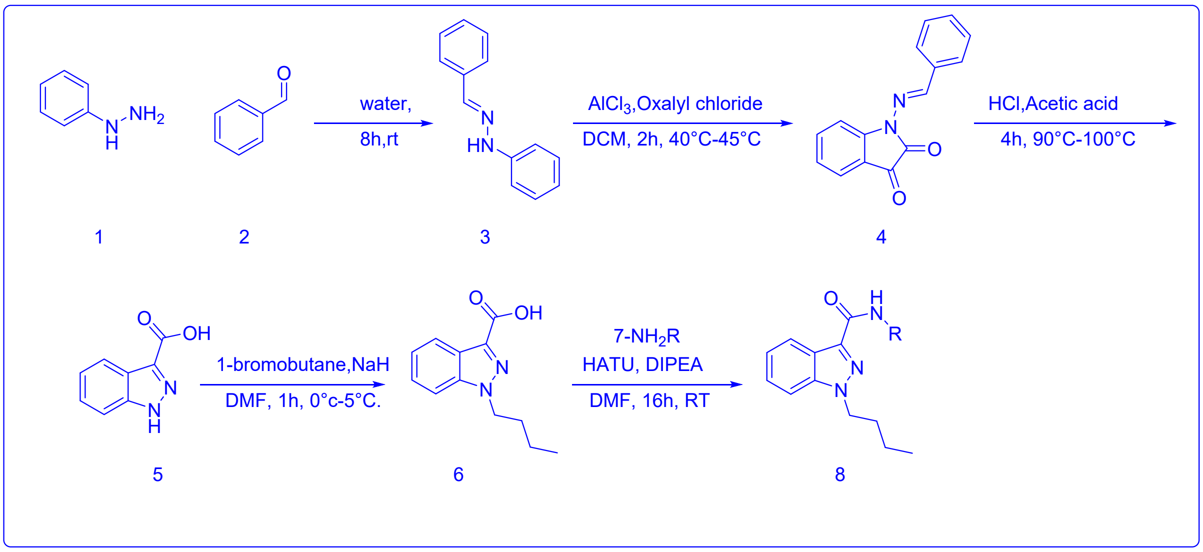

Table-1

2.1. Preparation of 2-benzylidene-1-phenylhydrazine (3):

To a stirred solution of phenyl hydrazine (50 gm) in water (500mL, 10 vol), add benzaldehyde (equivalent) very slowly at room temperature and stir for 8h. TLC showed the completion of starting material. Filter off the solid washed with water (150 mL) and chilled isopropyl alcohol (50 mL), and dry the solid under the oven at 70°C to 80°C for 8h to get 2-benzylidene-1-phenylhydrazine as a white solid

2.2. Preparation of 1H-indazole-3-carboxalic acid (5):

To a stirred solution of 2-benzylidene-1-phenylhydrazine (70 gm) in DCM (500 mL), oxalyl chloride (1.05 equivalent) was added at room temperature and stirred at 40°C to 45°C for 2h. TLC showed the completion of 2-benzylidene-1-phenylhydrazine, add aluminium trichloride (1.5 equivalent) at 40°C to 45°C and stir for 2h. TLC showed the completion of the intermediate (oxalyl chloride intermediate). Poured the reaction mixture into cooled water (350 mL), wash the combined organic layer and extract the aqueous layer with DCM (2x200 mL), wash the combined organic layer with 10% HCl and brine solution. Dry the organic layer over sodium sulphate and concentrated under reduced pressure. To the obtained crude add acetic acid (300 mL) and conc. HCl (100 mL), heated at 90°C to 100°C for 4h. Cool the reaction mixture to room temperature and stir for 2h. Filter off the solid material and washed with water. Dissolve the obtained solid into 5N sodium hydroxide solution, filter off the solid, wash with water, and discard the solid material. Take the aqueous layer into round bottom flask, and acidify the layer with conc. HCl up to pH 2, stir for 30 min and filter off the solid, wash the solid with water and dry the solid material under oven at 80°C for 4-5h to get 1H-indazole-3-carboxylic off white solid.

2.3. Preparation of 1-butyl-1H-indazole-3-carboxylic acid:

To a stirred suspension of sodium hydride (1.2 equivalent) in DMF (250 mL), add 1H-indazole-carboxylic acid (50 gm) dissolved in DMF (150 mL) at 5°C and stir for 1h. To this reaction, mass adds 1-Bromobutane (1.05 equivalent) at 10°C and stir at room temperature for 8h. TLC showed the completion of starting material and the formation of the non-polar spot. Quenched the reaction mass into ice water, wash the aqueous layer with ethyl acetate, and acidify the organic layer using con. HCl up to pH reaches 1, extract the layer with ethyl acetate (2 x 200 mL), wash the organic layer with brined solution (2 x 100 mL), dry over sodium sulphate and concentrate. The obtained crude was stirred in n-hexane at 15°C for 1h. Filter off the solid compound, wash with chilled hexane, after that washed with sodium bicarbonate and organic layer was acidify with conc. HCl up to PH 2, concerted organic layer under reduced pressure to obtained solid was dried in oven at 50°C-60°C to get 1-butyl-1H-indazole-3-carboxylic acid as off white solid.

2.4. General Procedure for the synthesis 8a-8z:

To a stirred solution of 1-butyl-1H-indazole-3-carboxylic acid (250 mg, 1.146mmol) was dissolved in DMF (10 mL), HATU (2 equivalents) and DIPEA (3 equivalents) were added to the reaction mixture, then commercial amines (2 equivalents) were added. The reaction mixture was stirred at room temperature for 8-16h. After completion of the reaction, the resultant reaction mixture was poured into water, the solution was extracted with water and ethyl acetate (4 x 20 mL). The organic layer was dried with anhydrous sodium sulphate and the solvent was removed under reduced pressure to afford crude product. The crude was purified by silica gel chromatography to obtain pure products **8a-8z**.

2.5. Plausible mechanism:

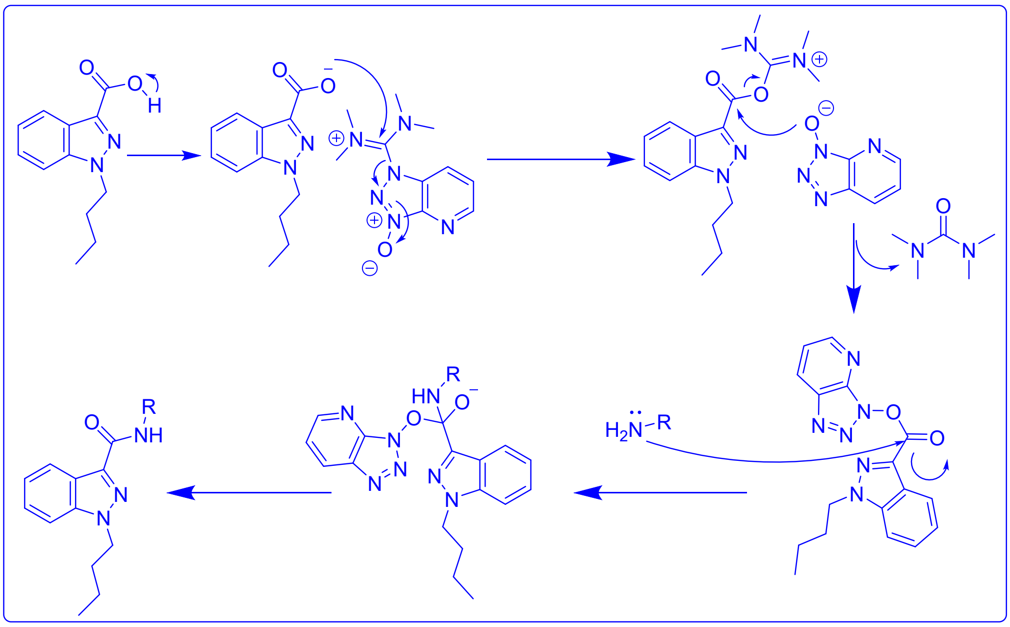

Table-2

R = 8a-8z aliphatic and aromatic amines

8a = Ammonium chloride
8b = Aniline
8c = phenylmethanamine
8d = 3-bromoaniline
8e = 2-amino-5-iodobenzoic acid
8f = 4-benzylaniline
8g = ethyl 4-aminobenzoate
8h = (4-nitrophenyl)hydrazine
8i = 4-bromoaniline
8j = 4-aminophenol
8k = 2-amino-3-methylphenol
8l = m-toluidine
8m = o-toluidine
8n = 5-methylpyridin-2-amine
8o = 4-methoxyaniline
8p = 4-fluoroaniline
8q = 2-methoxyaniline
8r = 3-aminophenol
8s = 4H-1,2,4-triazol-4-amine
8t = 4-nitrobenzene-1,2-diamine
8u = phenylhydrazine
8v = (2,4-dinitrophenyl)hydrazine
8w = 4-hydrazineylbenzonitrile
8x = 4-hydrazineylphenol
8y = (4-bromophenyl)hydrazine
8z = 4-nitroaniline

Table-3

2.6. Acid- Amide cross-coupling derivatives of indazole amides (8a-8z)

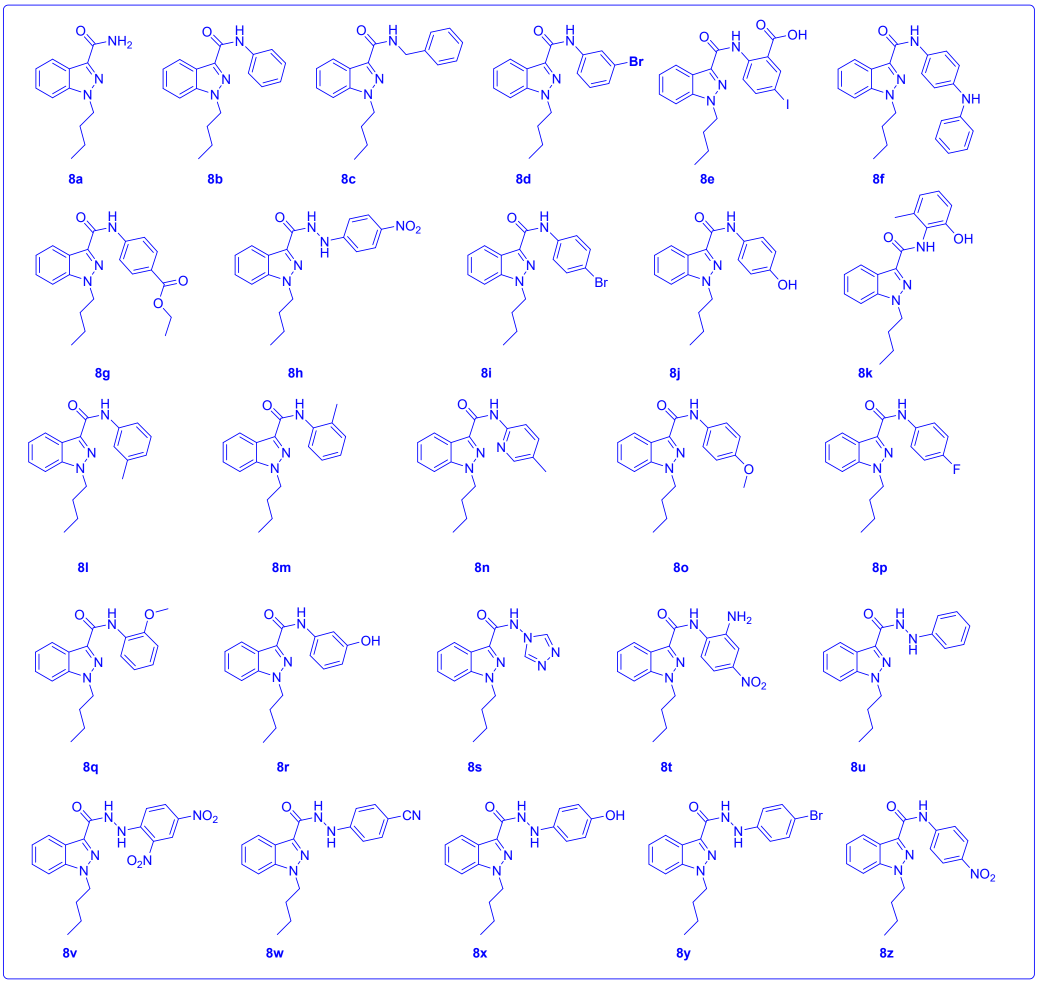

Table-4

3. ^{1}H and ^{13}C NMR, Dept-135, COSY, HSQC, IR and HRMS spectra of the compounds

^{1}H-NMR [400MHz, DMSO-d_6] spectrum of 1-benzylidene-2-phenyl hydrazine(3).

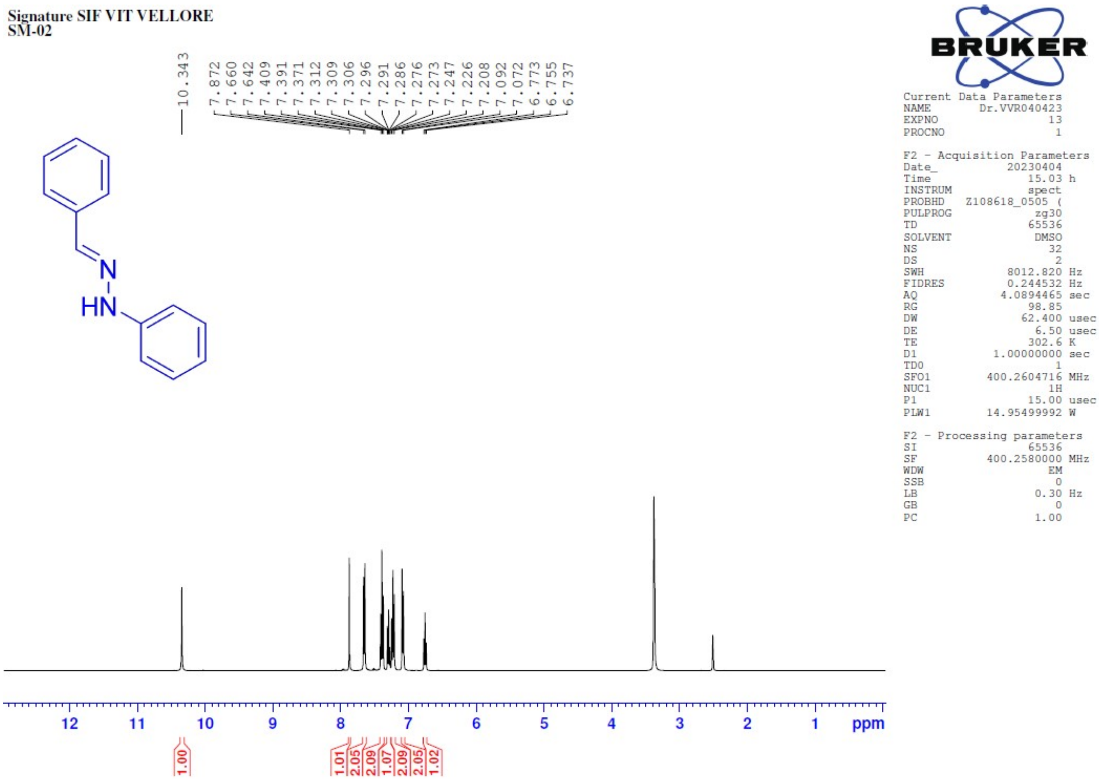

^{1}H-NMR [400MHz, DMSO-d_6] spectrum of 1H-indazole-3-carboxalic acid (5).

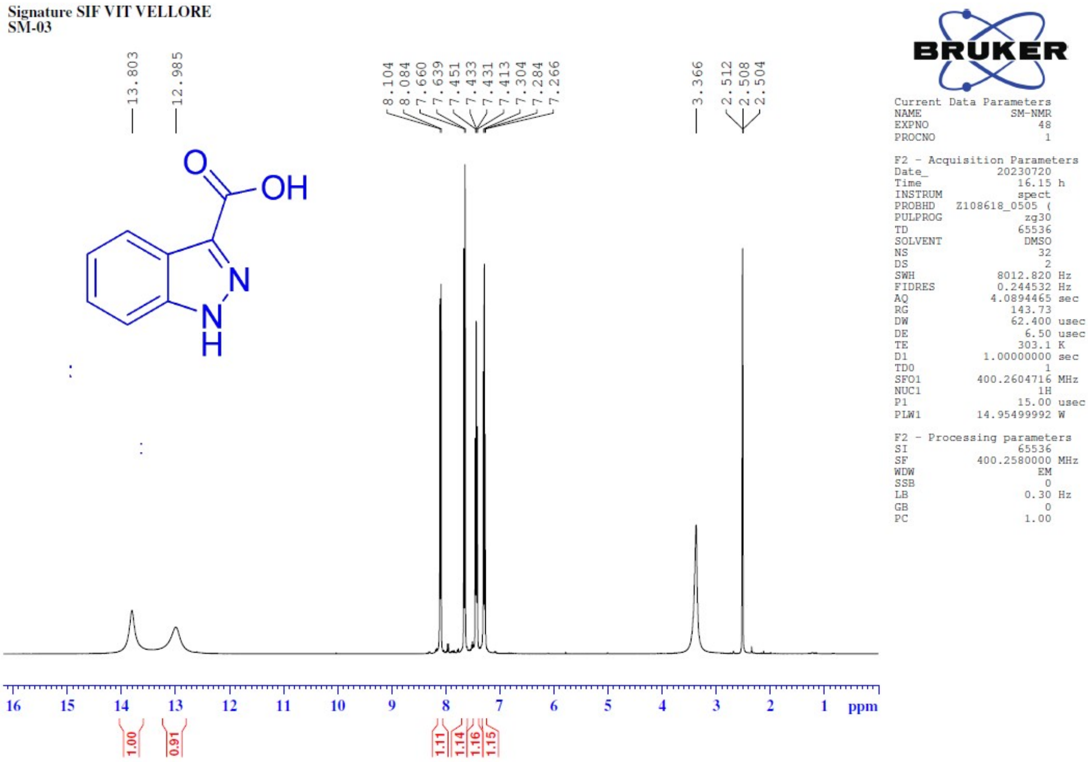

^{1}H-NMR [400MHz, DMSO-d_6] spectrum of 1-butyl-1H-indazole-3-carboxylic acid (6).

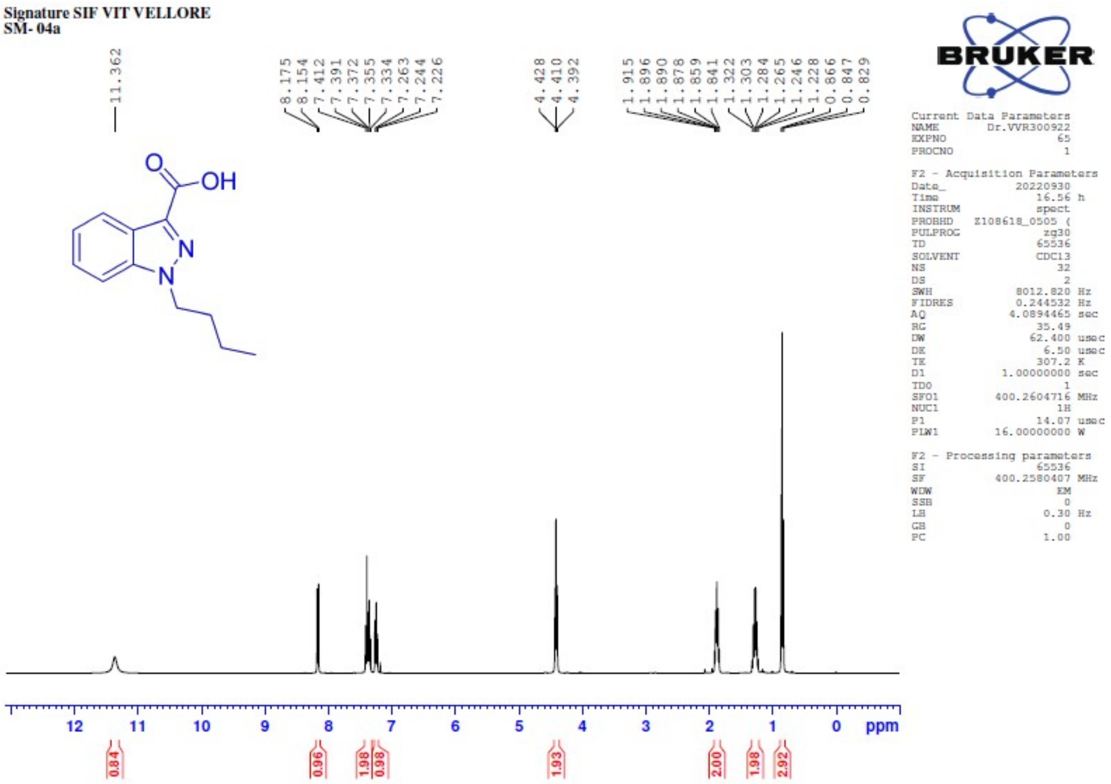

^{1}H-NMR [400MHz, DMSO-d$_6$] spectrum of 1-butyl-1H-indazole-3-carboxamide (8a).

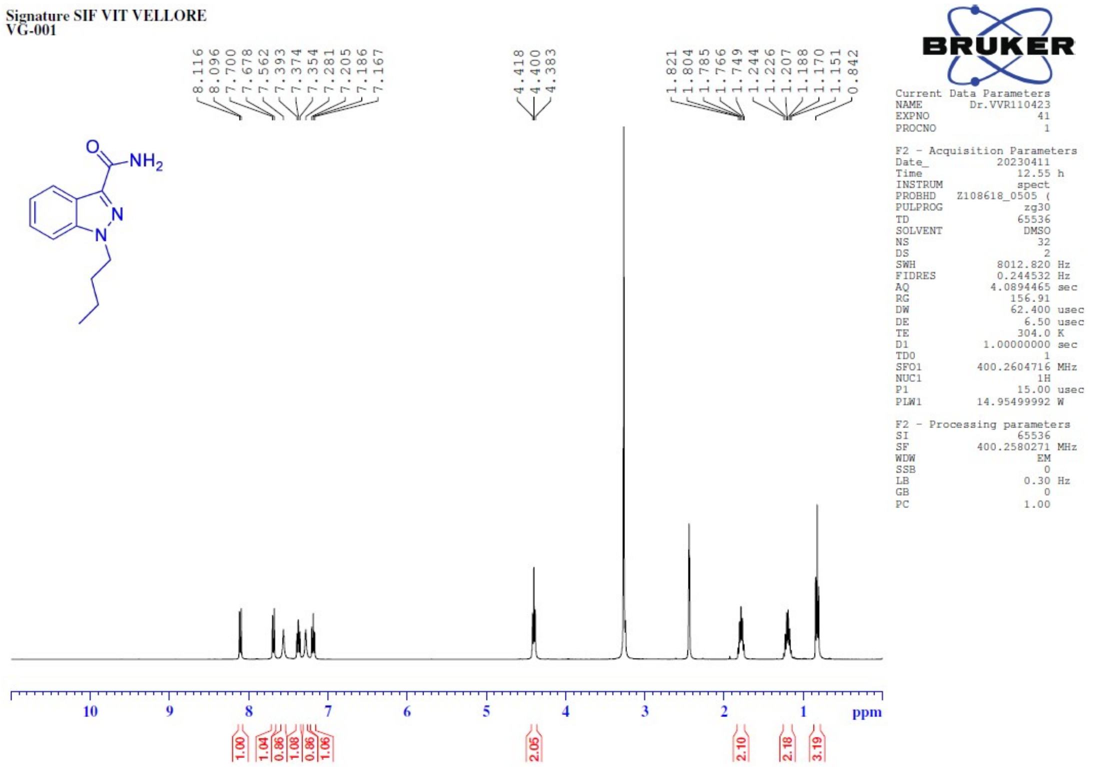

^{13}C-NMR [100MHz, DMSO-d_6] spectrum of 1-butyl-1H-indazole-3-carboxamide (8a).

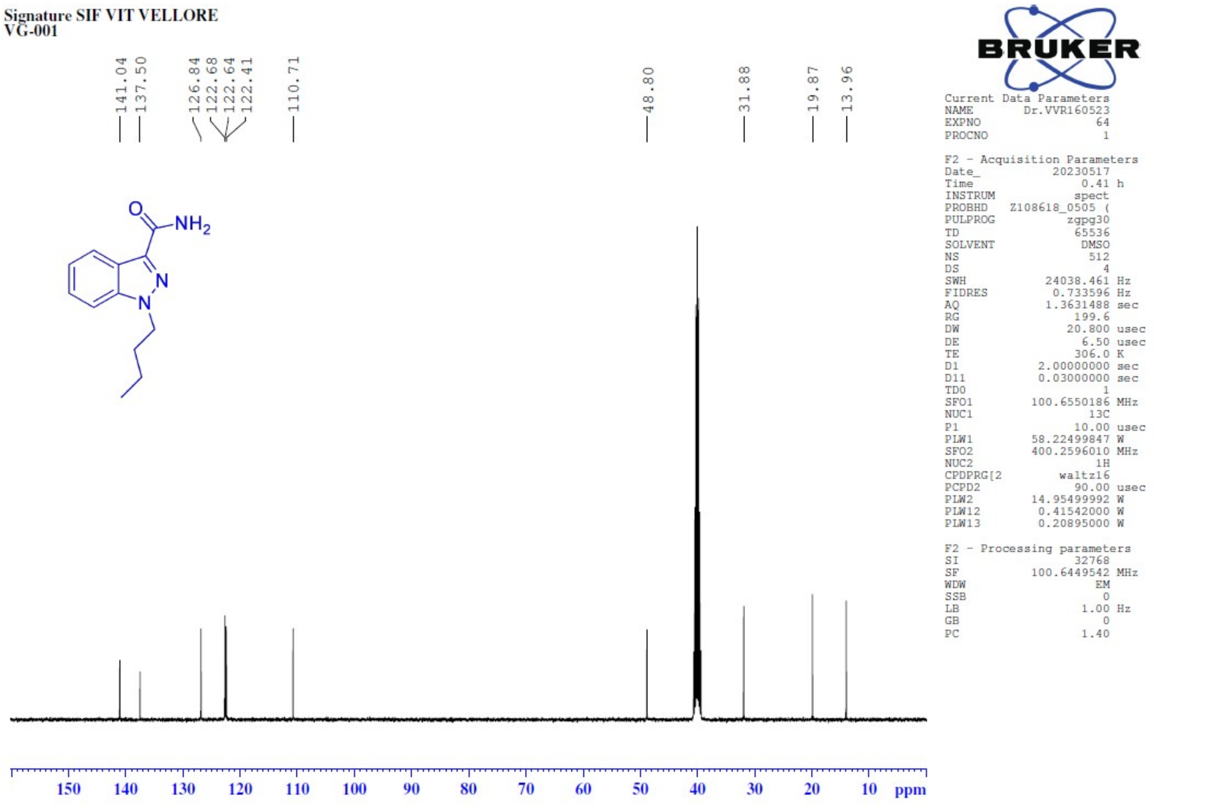

135-DEPT-NMR [100MHz, DMSO-d_6] spectrum of 1-butyl-1H-indazole-3-carboxamide (8a).

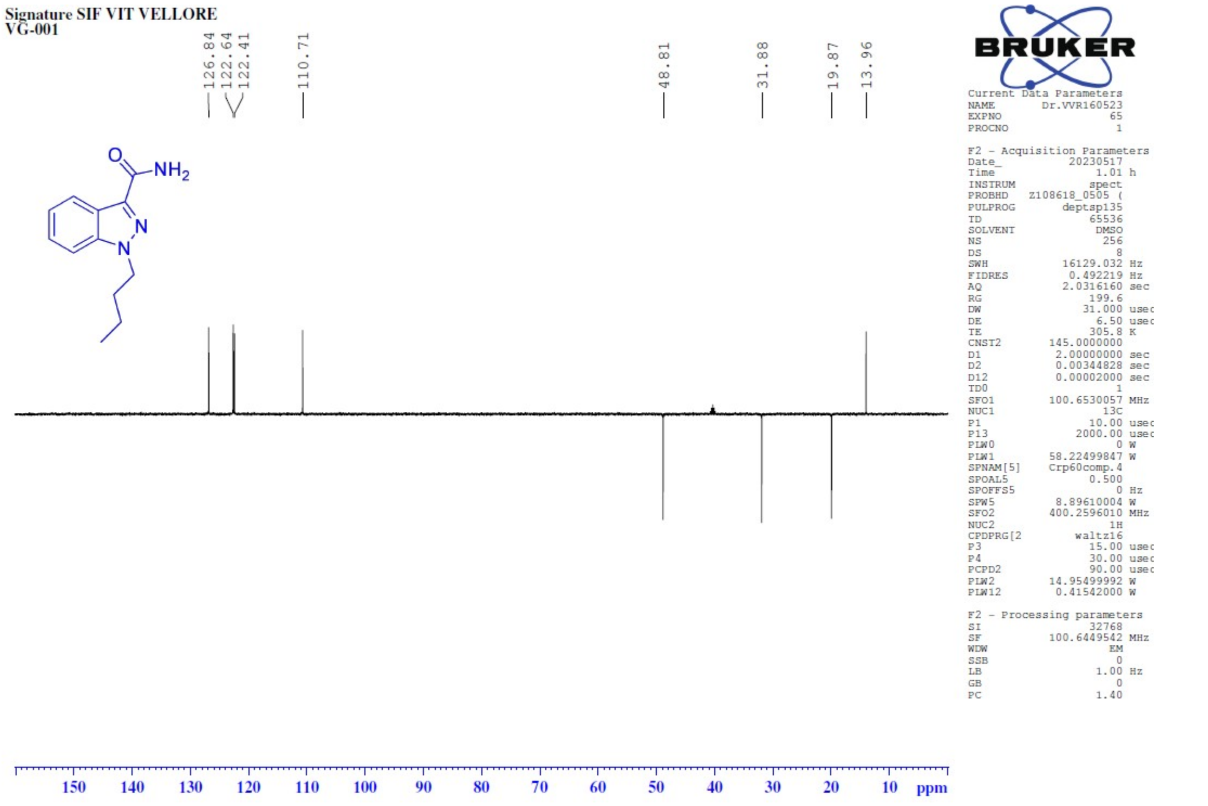

COSY-NMR [400MHz, DMSO-d_6] spectrum of 1-butyl-1H-indazole-3-carboxamide (8a).

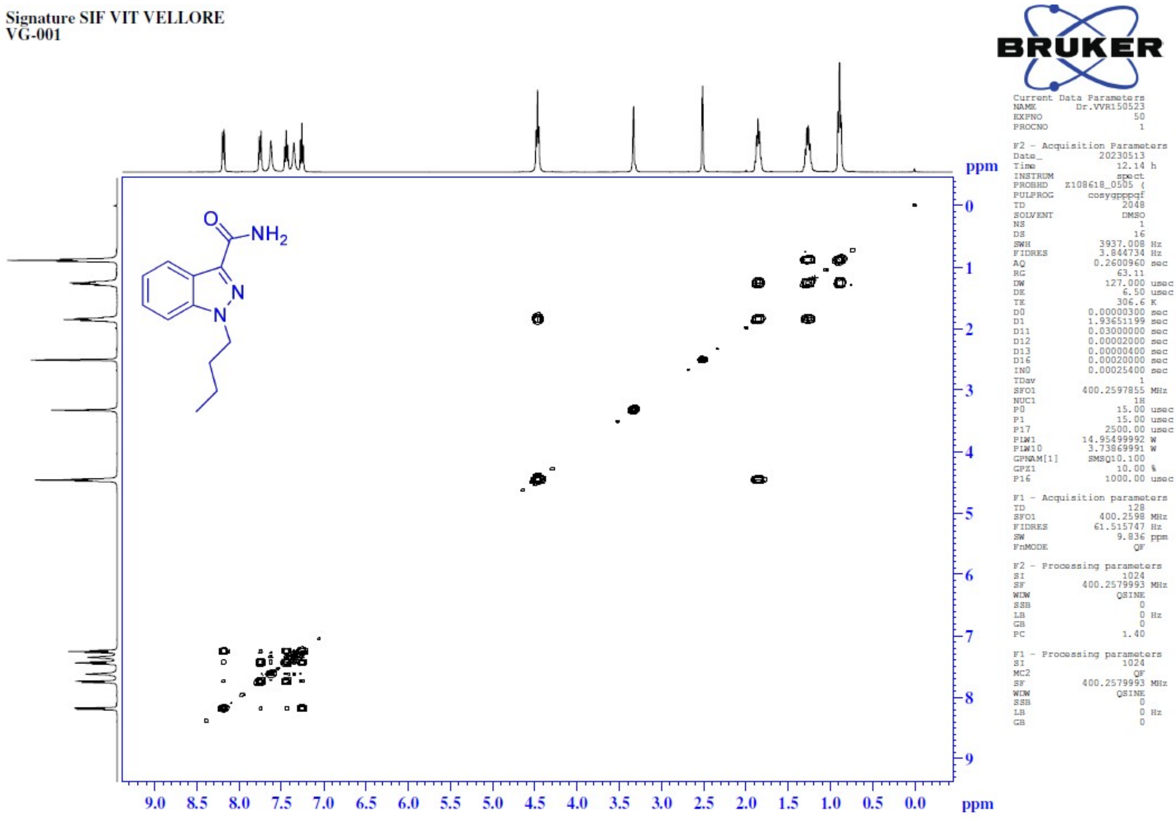

HSQC-NMR [400MHz, DMSO-d_6] spectrum of 1-butyl-1H-indazole-3-carboxamide (8a).

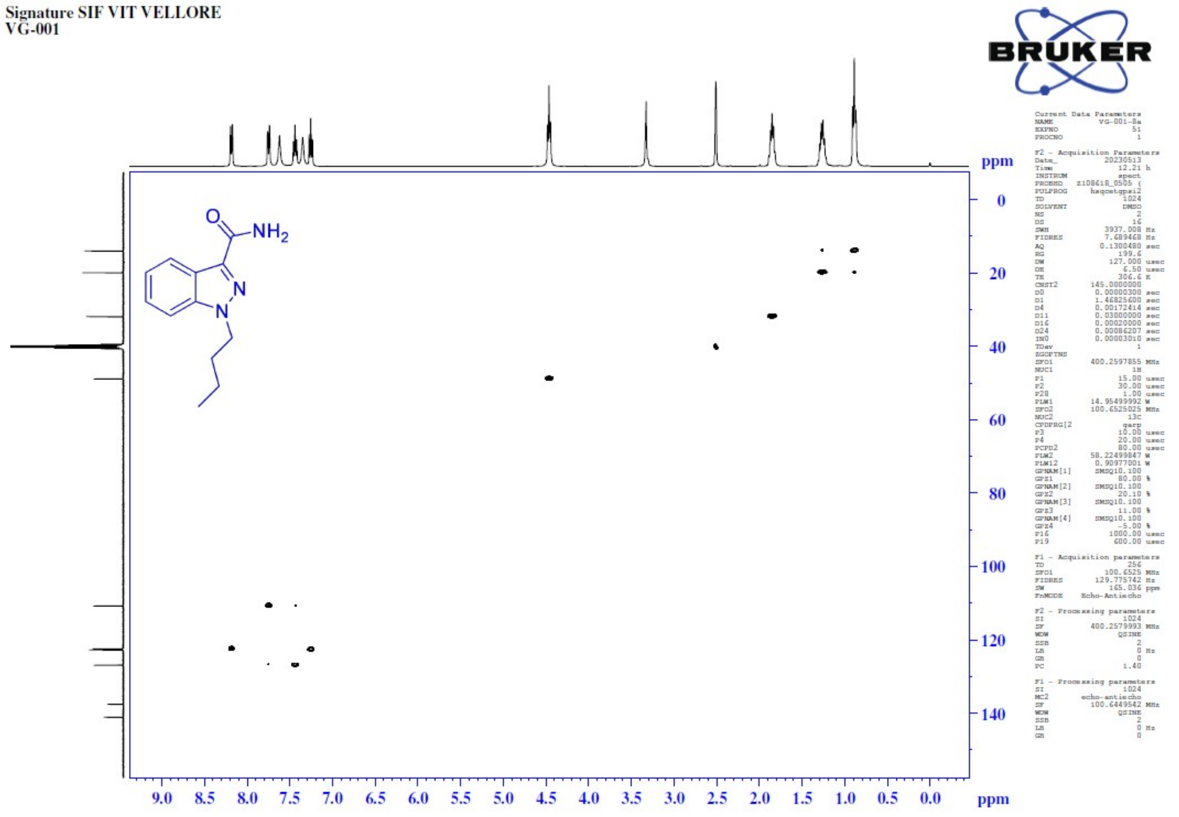

FT-IR spectrum of 1-butyl-1H-indazole-3-carboxamide (8a).

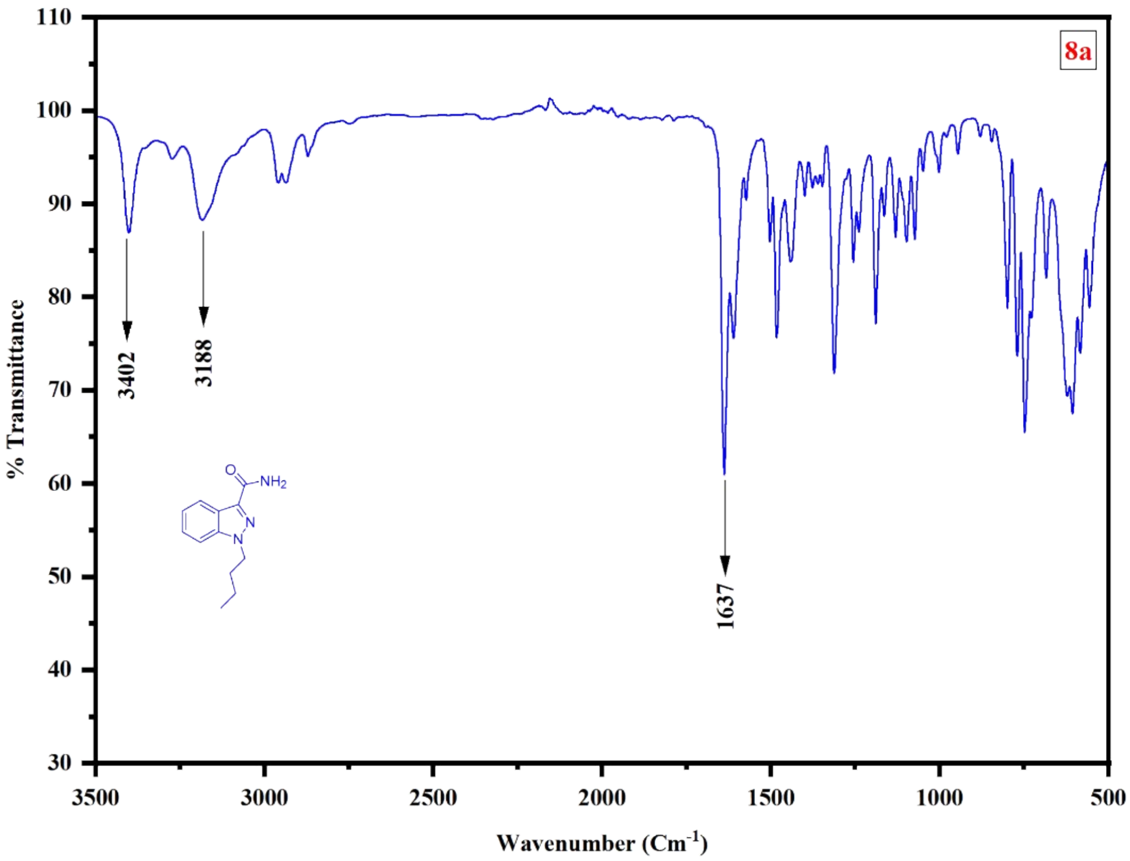

HRMS of 1-butyl-1H-indazole-3-carboxamide (8a)

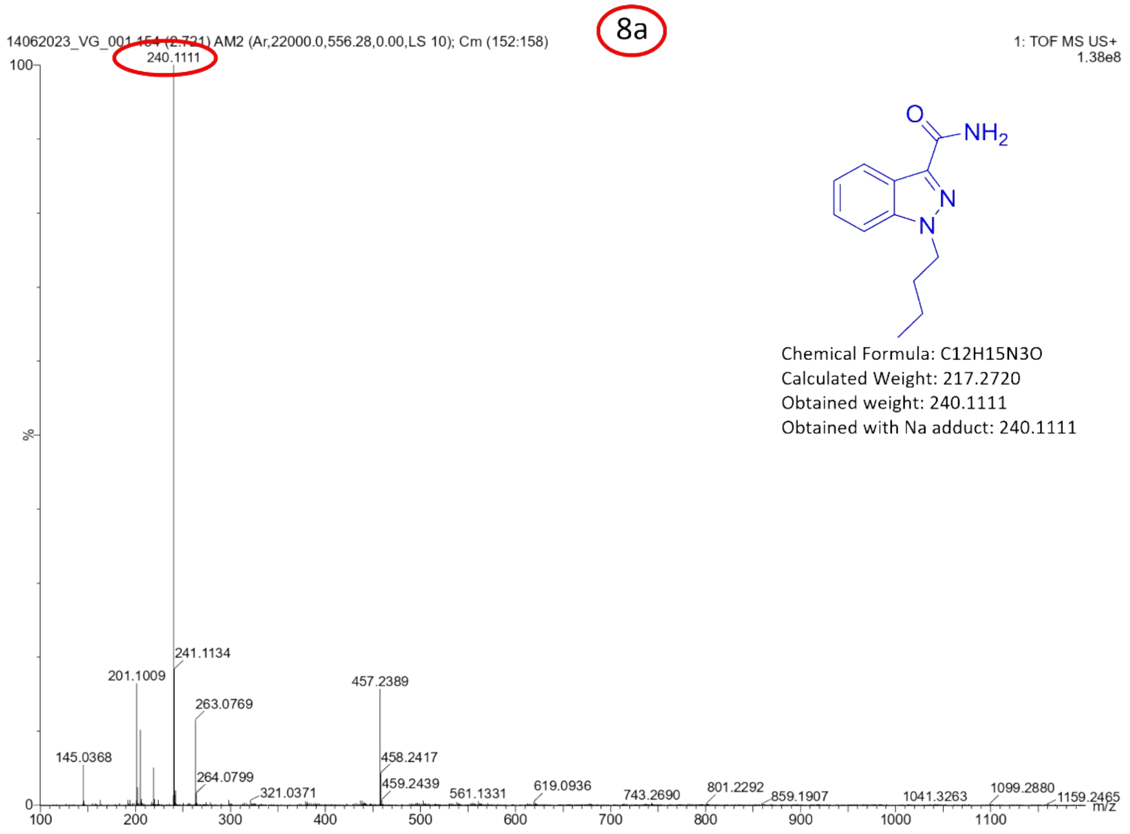

^{1}H-NMR [400MHz, DMSO-d_6] spectrum of 1-butyl-N-phenyl-1H-indazole-3-carboxamide (8b).

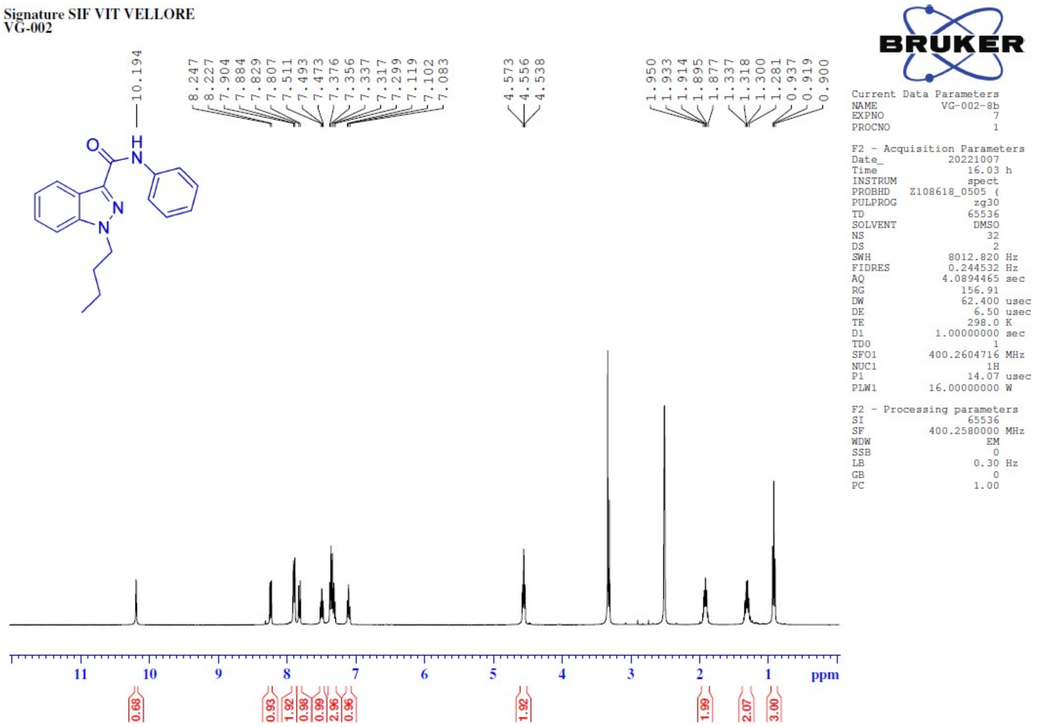

^{13}C-NMR [100MHz, DMSO-d_6] spectrum of 1-butyl-N-phenyl-1H-indazole-3-carboxamide (8b).

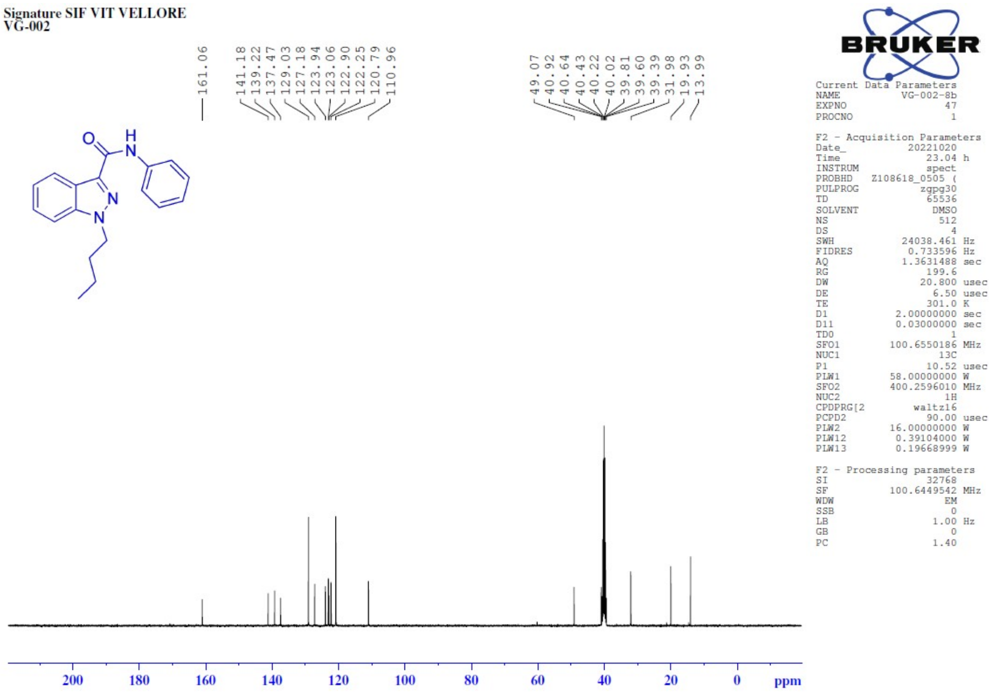

135-DEPT-NMR [100MHz, DMSO-d_6] spectrum of 1-butyl-N-phenyl-1H-indazole-3-carboxamide (8b).

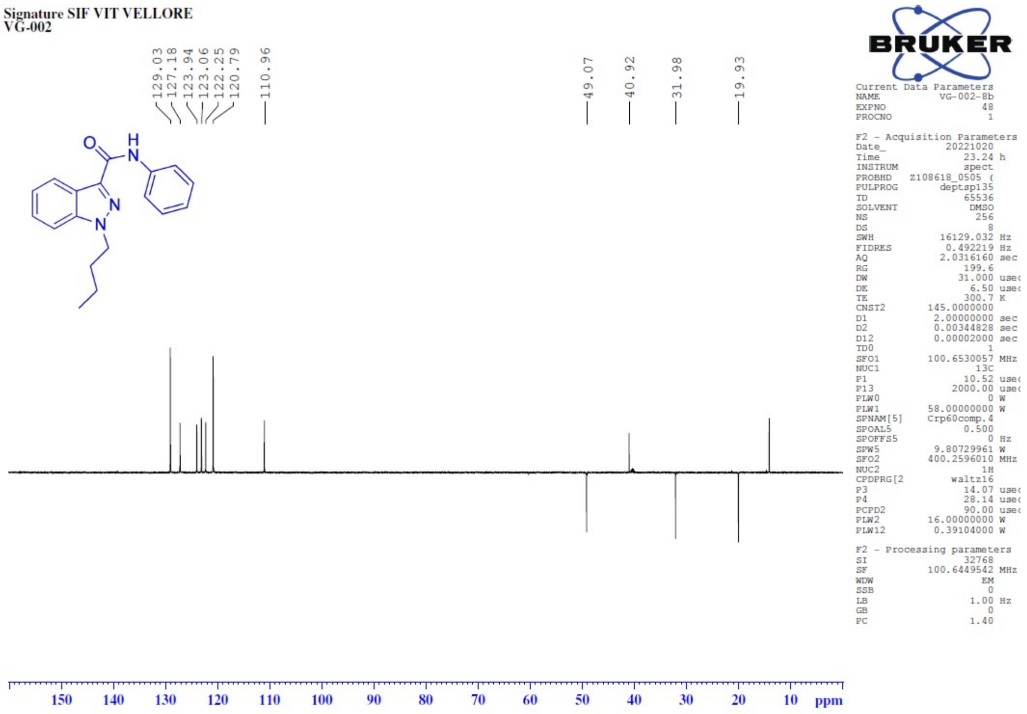

COSY-NMR [400MHz, DMSO-d_6] spectrum of 1-butyl-N-phenyl-1H-indazole-3-carboxamide (8b).

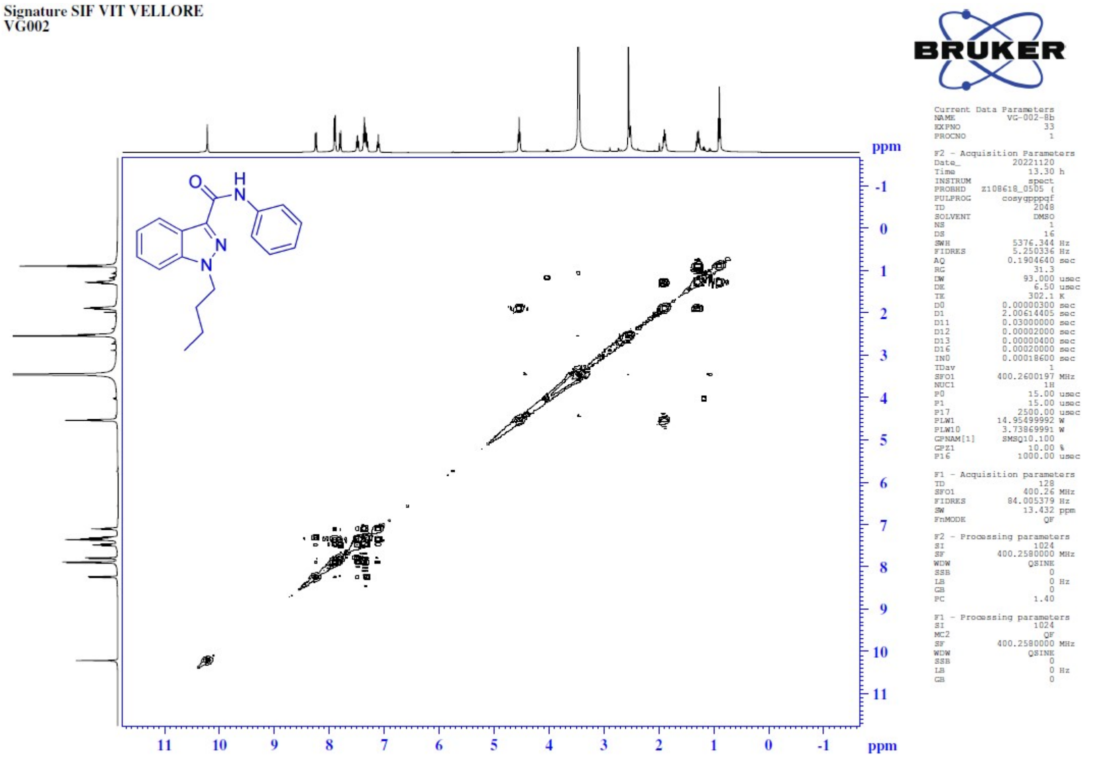

HSQC-NMR [400MHz, DMSO-d_6] spectrum of 1-butyl-N-phenyl-1H-indazole-3-carboxamide (8b).

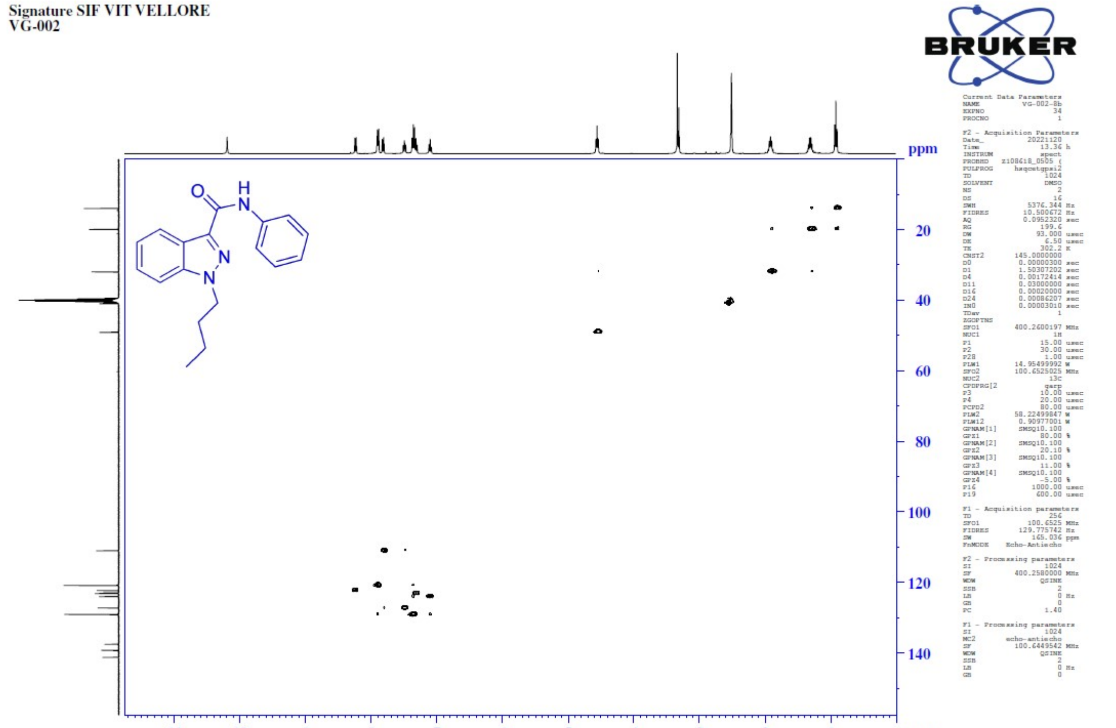

FT-IR spectrum of 1-butyl-N-phenyl-1H-indazole-3-carboxamide (8b).

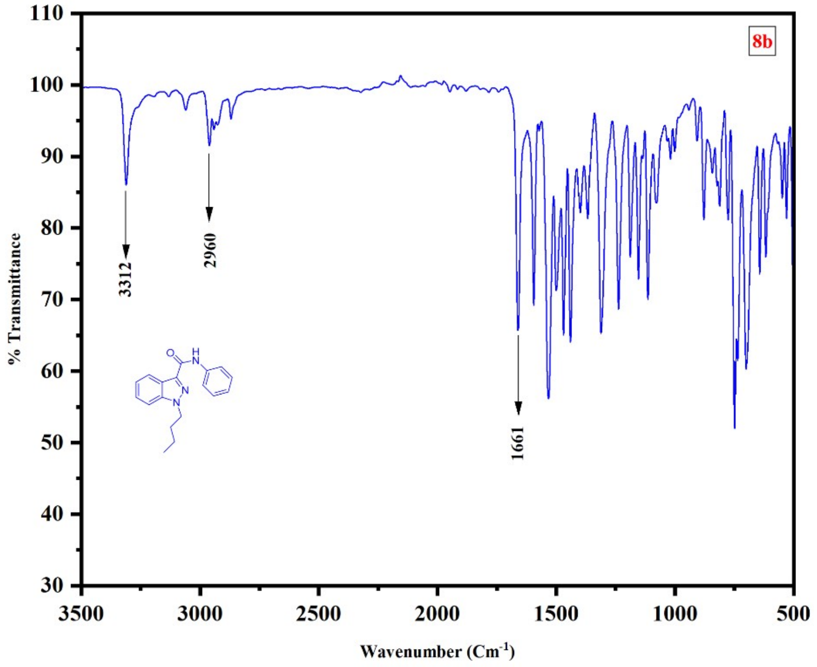

HRMS of 1-butyl-N-phenyl-1H-indazole-3-carboxamide (8b).

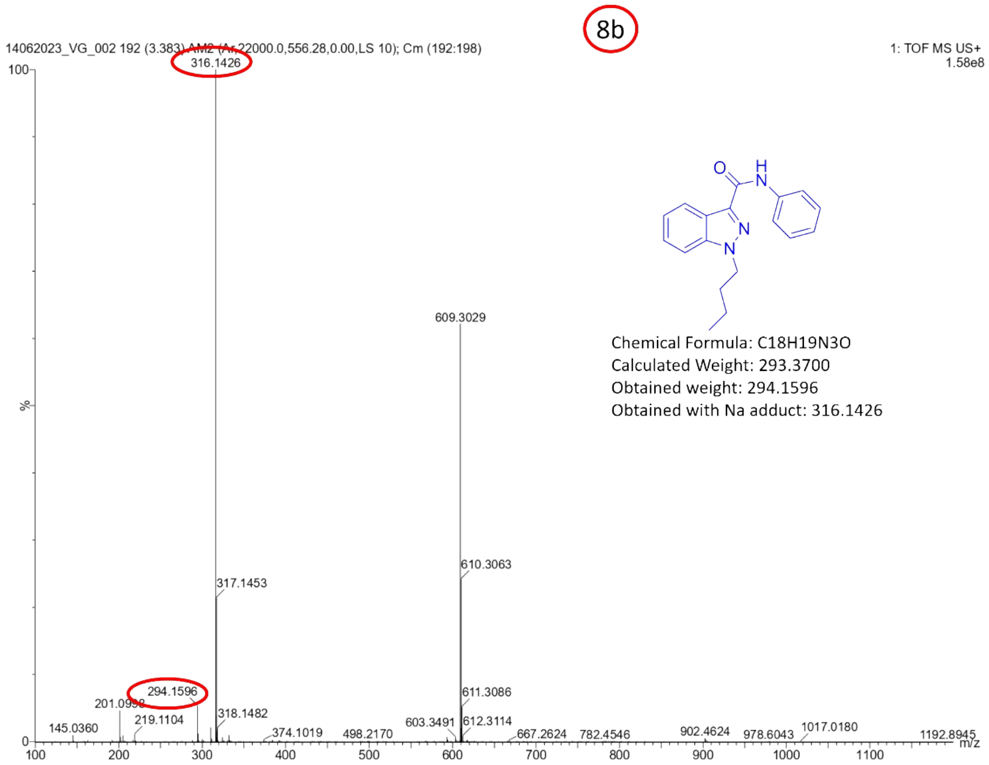

^{1}H-NMR [400MHz, DMSO-d_6] spectrum of N-benzyl-1-butyl-1H-indazole-3-carboxamide (8c).

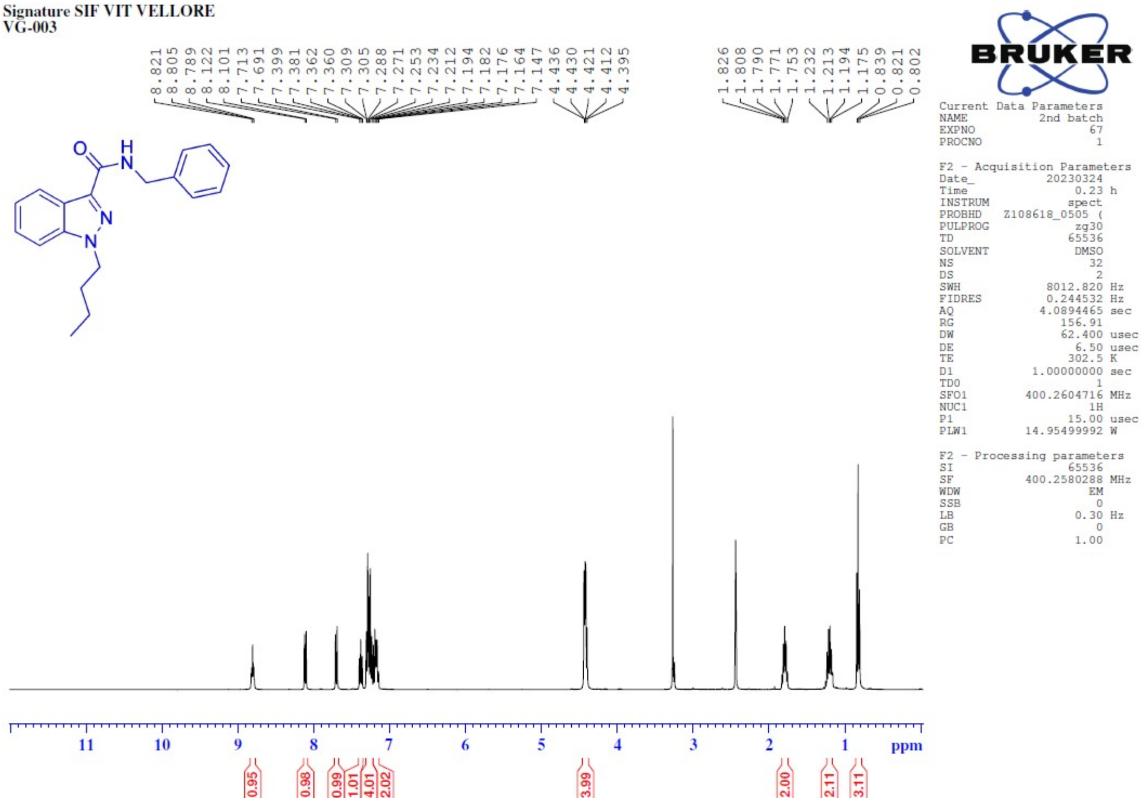

^{13}C-NMR [100MHz, DMSO-d_6] spectrum of N-benzyl-1-butyl-1H-indazole-3-carboxamide (8c).

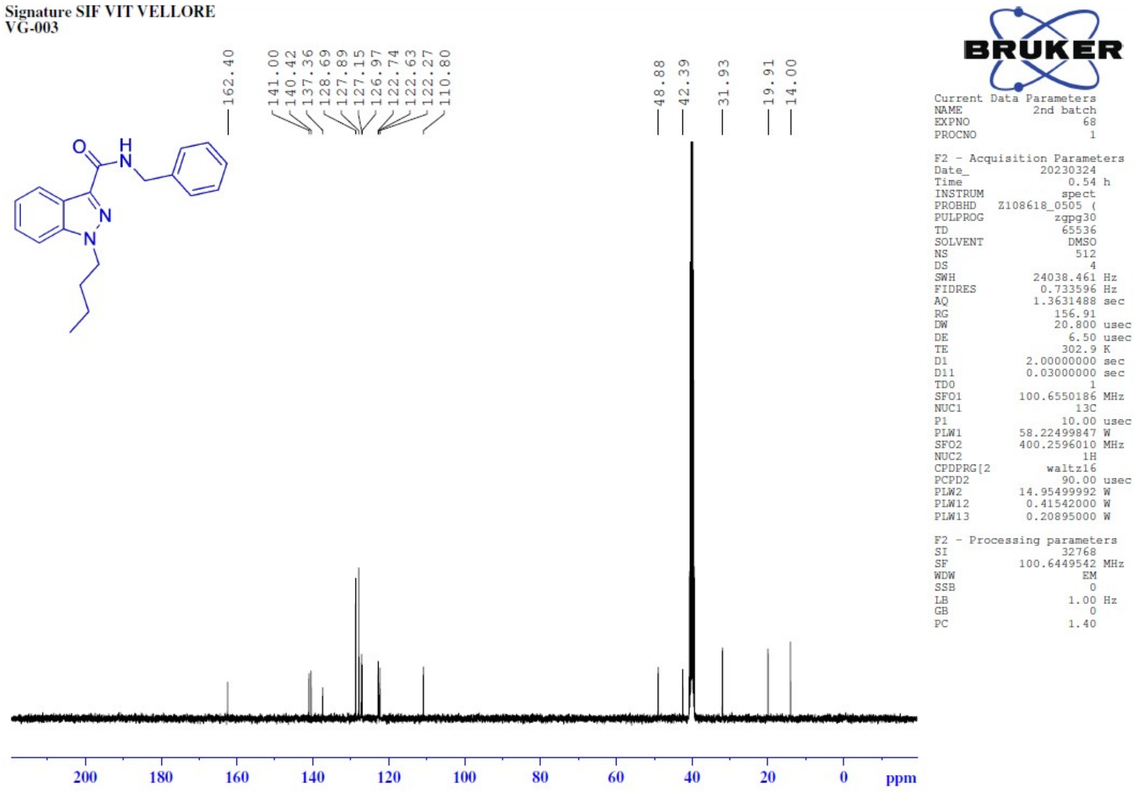

135-DEPT-NMR [100MHz, DMSO-d_6] spectrum of N-benzyl-1-butyl-1H-indazole-3-carboxamide (8c).

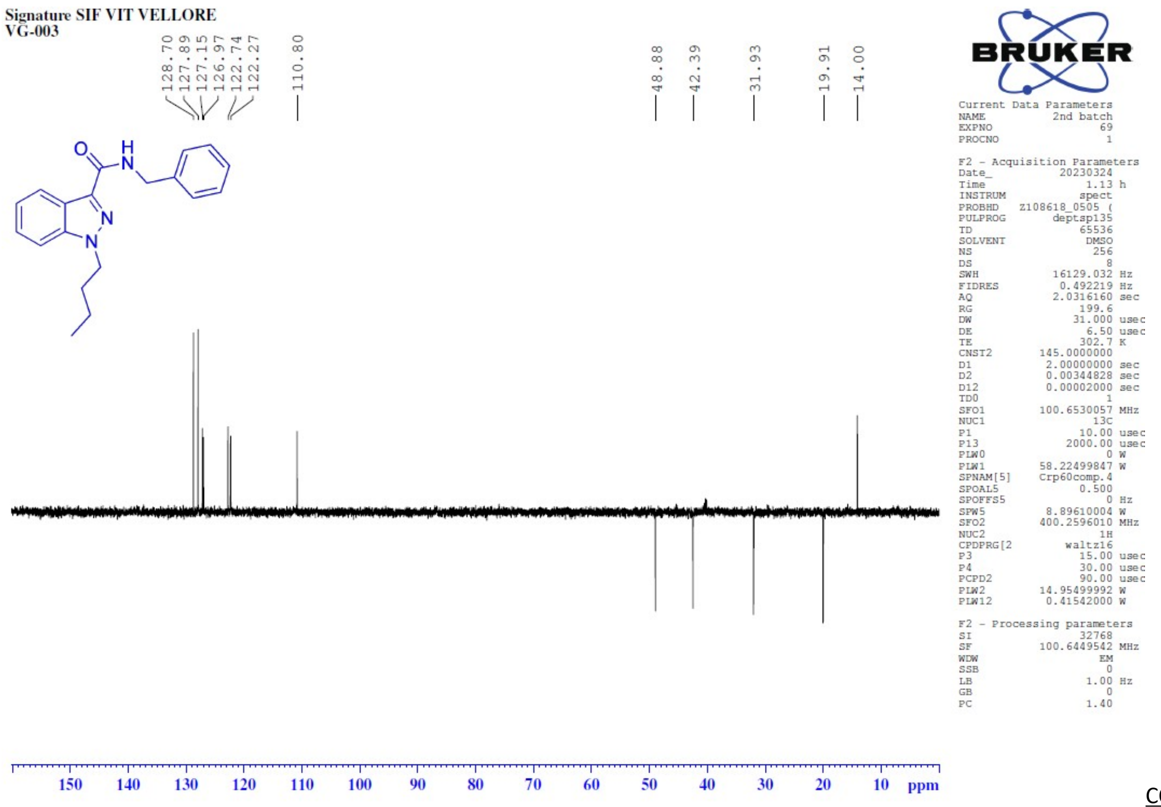

COSY-NMR [400MHz, DMSO-d$_6$] spectrum of N-benzyl-1-butyl-1H-indazole-3-carboxamide (8c).

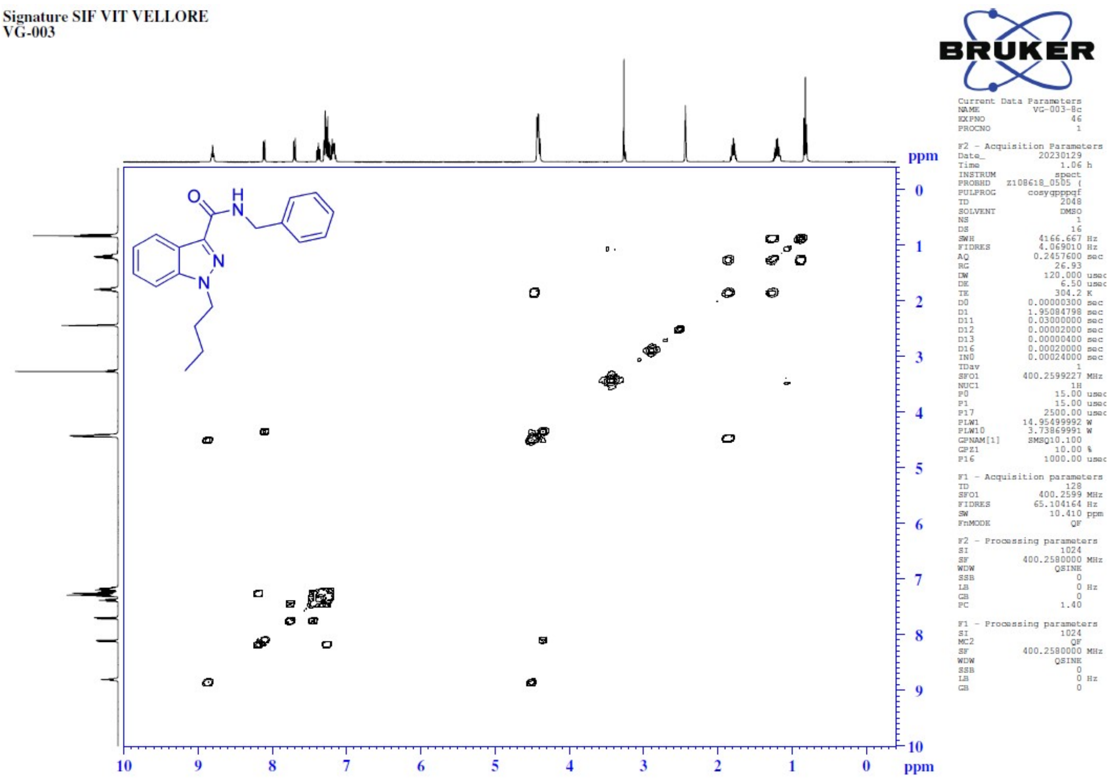

HSQC-NMR [400MHz, DMSO-d$_6$] spectrum of N-benzyl-1-butyl-1H-indazole-3-carboxamide (8c).

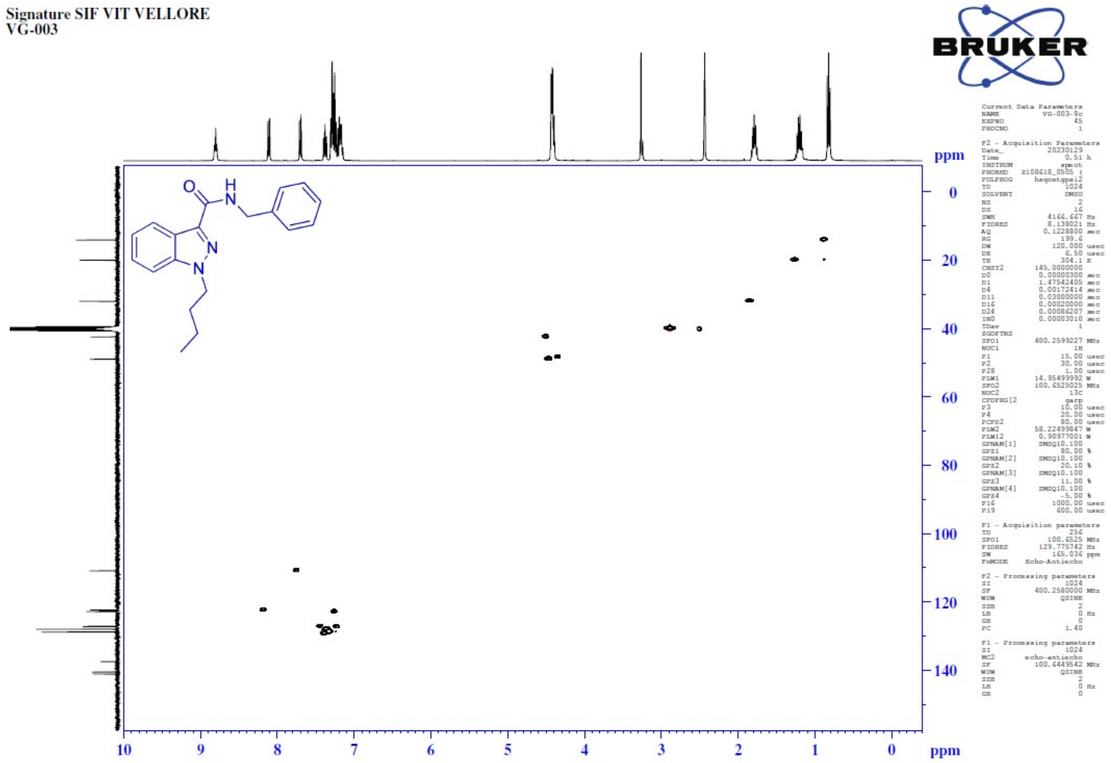

FT-IR spectrum of N-benzyl-1-butyl-1H-indazole-3-carboxamide (8c).

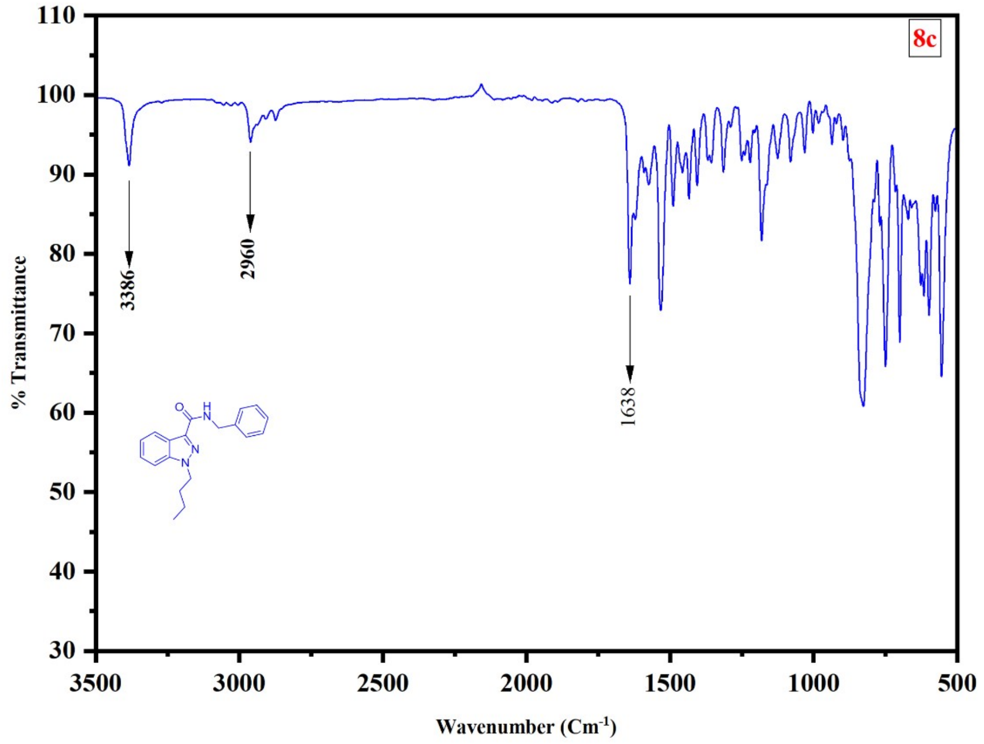

HRMS of N-benzyl-1-butyl-1H-indazole-3-carboxamide (8c).

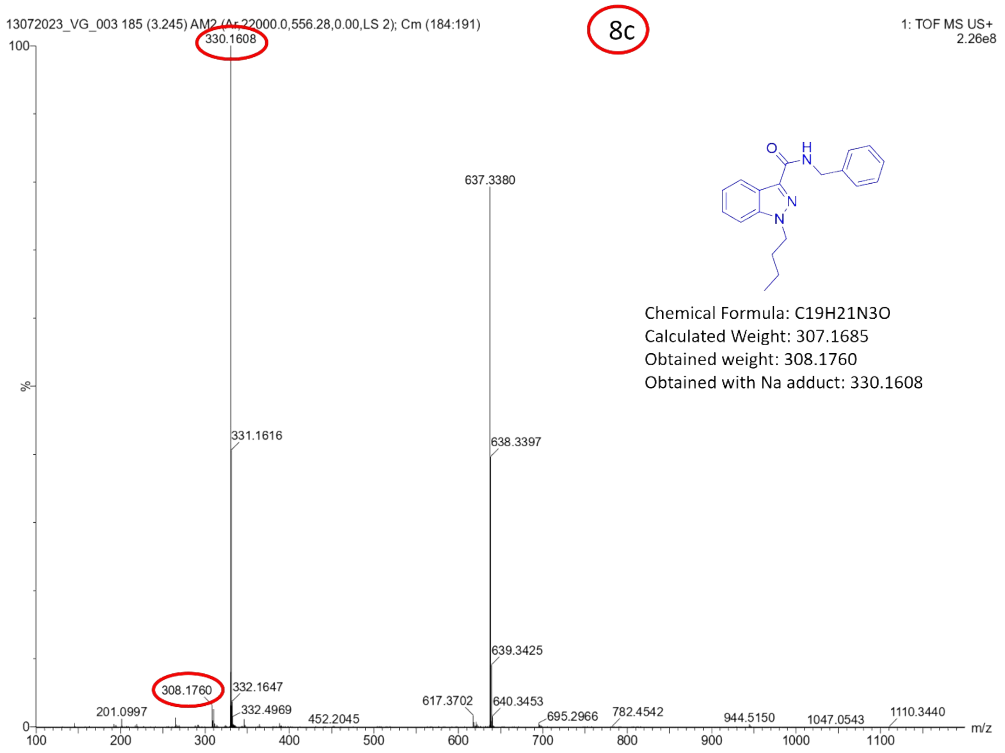

^{1}H-NMR [400MHz, DMSO-d_6] spectrum of 1-butyl-N-(6-methylpyridine-2yl)-1H-indazole-3-carboxamide (8d).

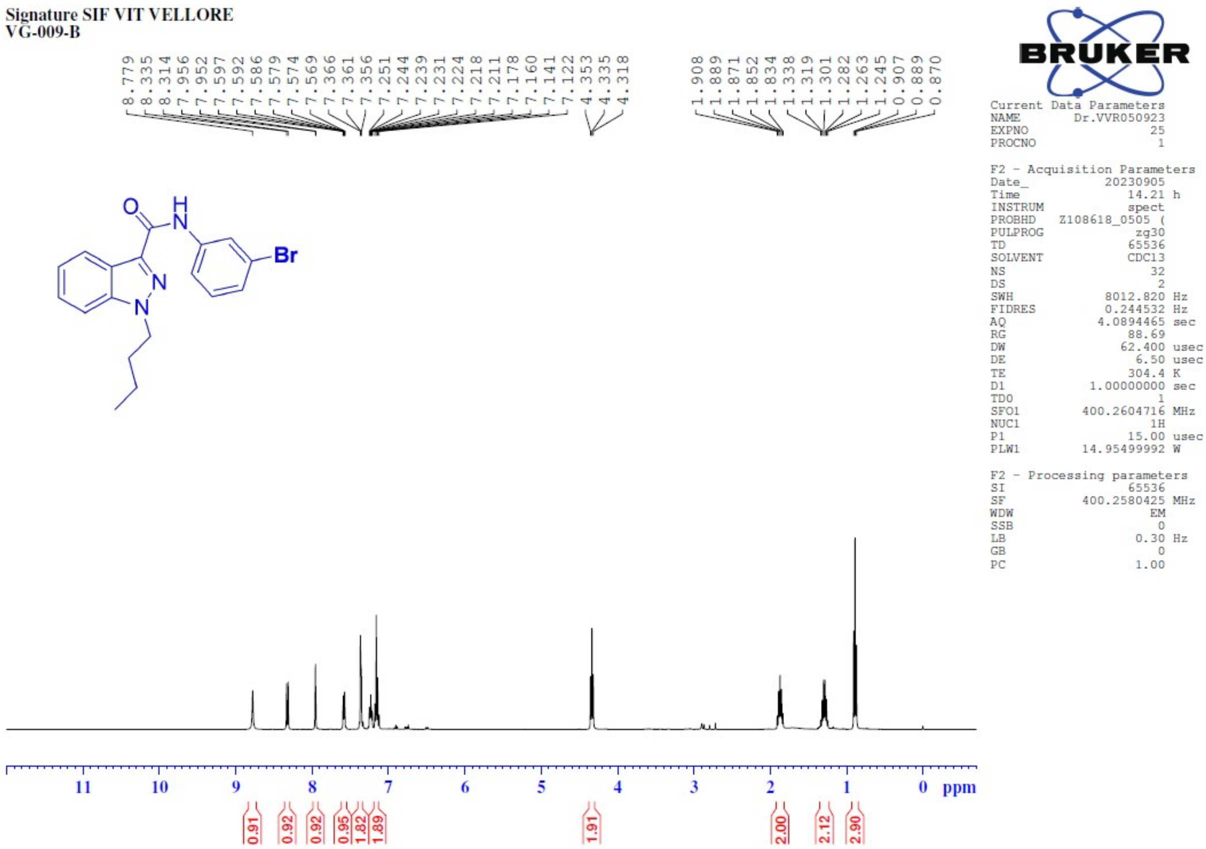

^{13}C-NMR [100MHz, DMSO-d_6] spectrum of 1-butyl-N-(6-methylpyridine-2yl)-1H-indazole-3-carboxamide (8d).

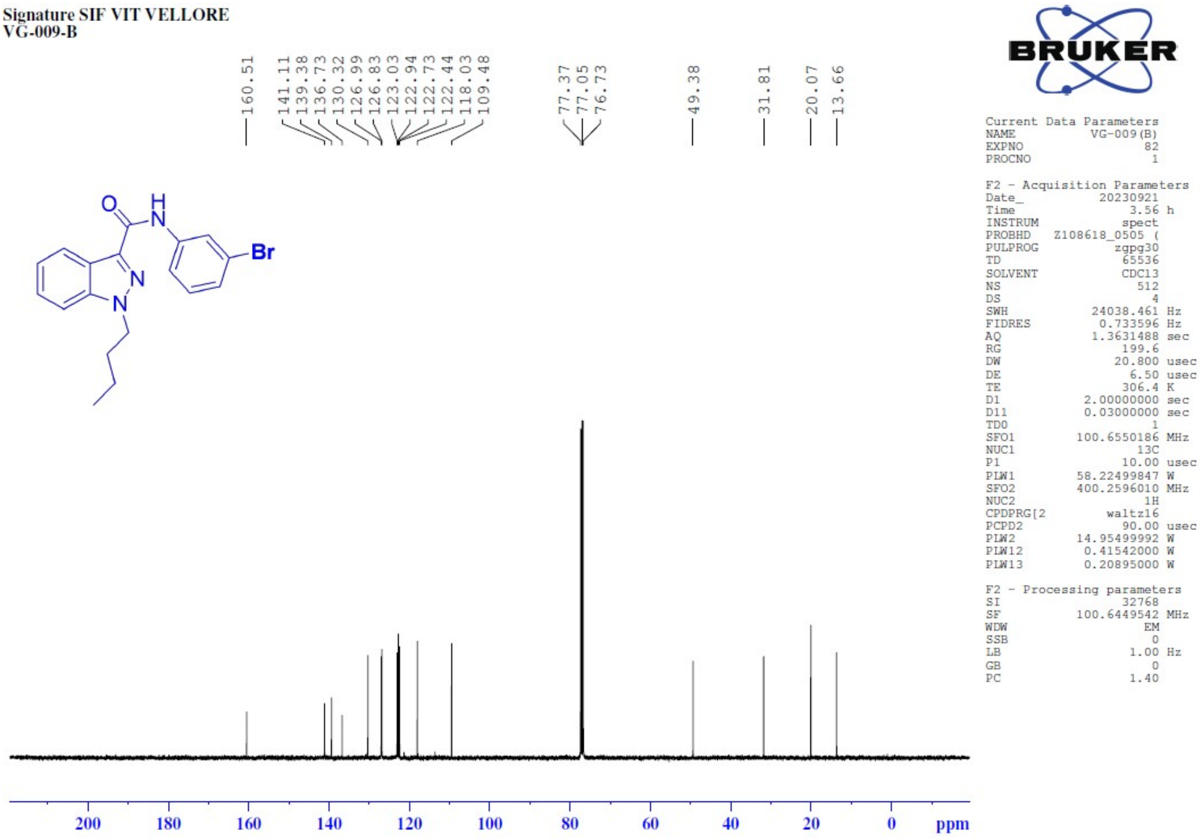

135-DEPT-NMR [100MHz, DMSO-d_6] spectrum of 1-butyl-N-(6-methylpyridine-2yl)-1H-indazole-3-carboxamide (8d).

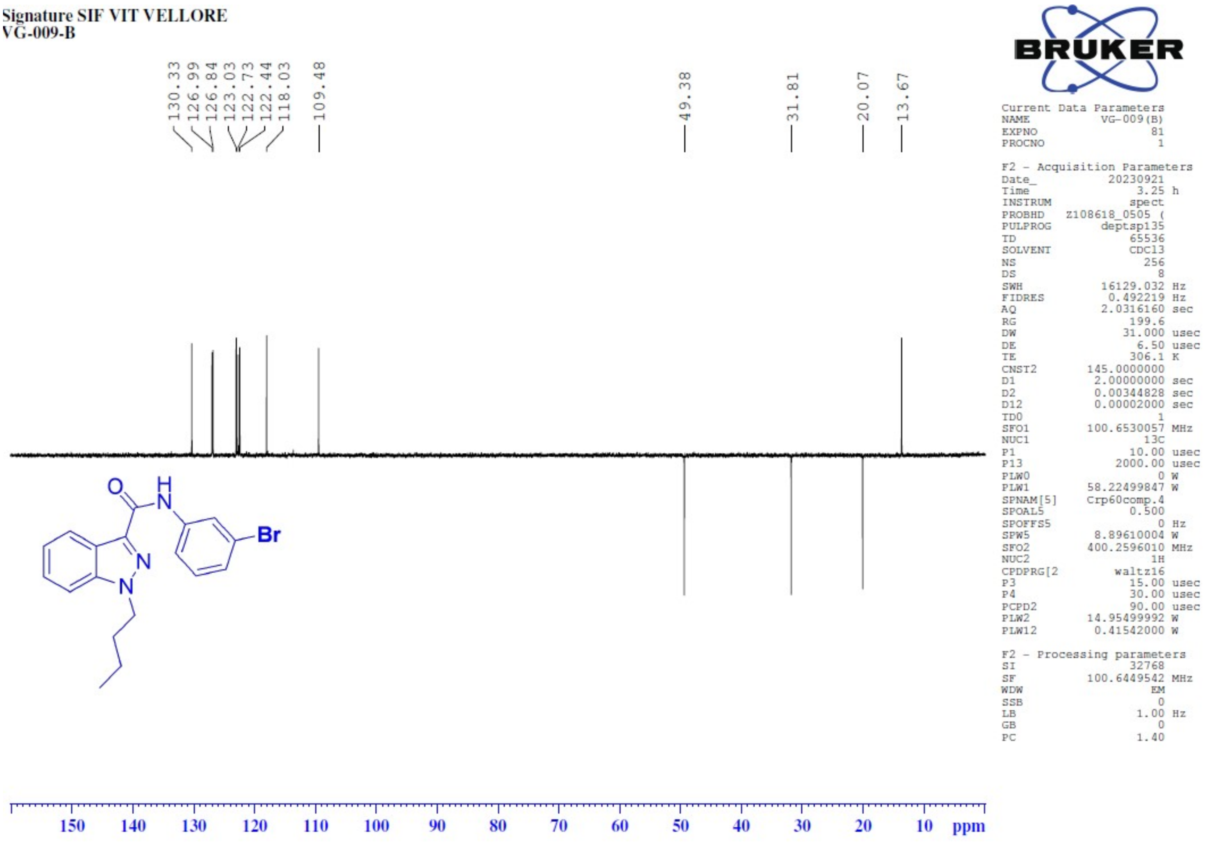

COSY-NMR [400MHz, DMSO-d_6] spectrum of 1-butyl-N-(6-methylpyridine-2yl)-1H-indazole-3-carboxamide (8d).

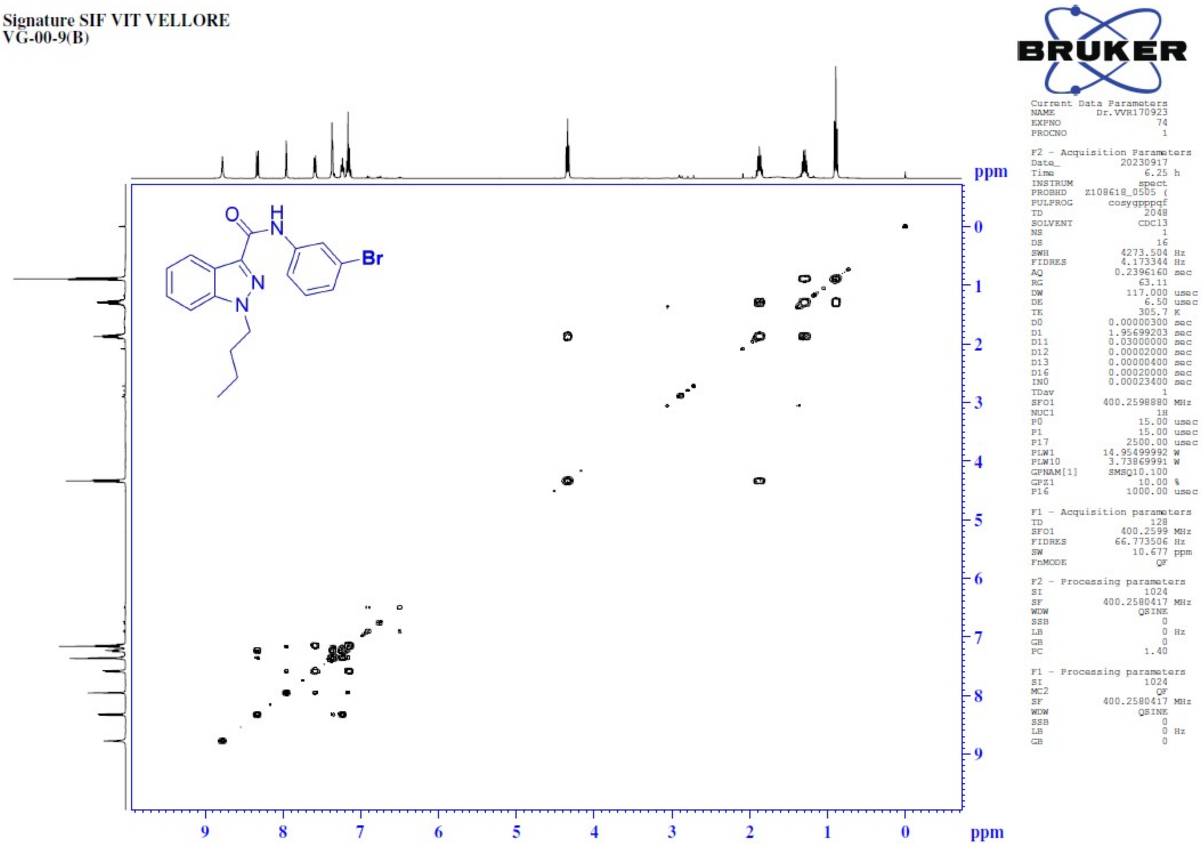

HSQC-NMR [400MHz, DMSO-d_6] spectrum of 1-butyl-N-(6-methylpyridine-2yl)-1H-indazole-3-carboxamide (8d).

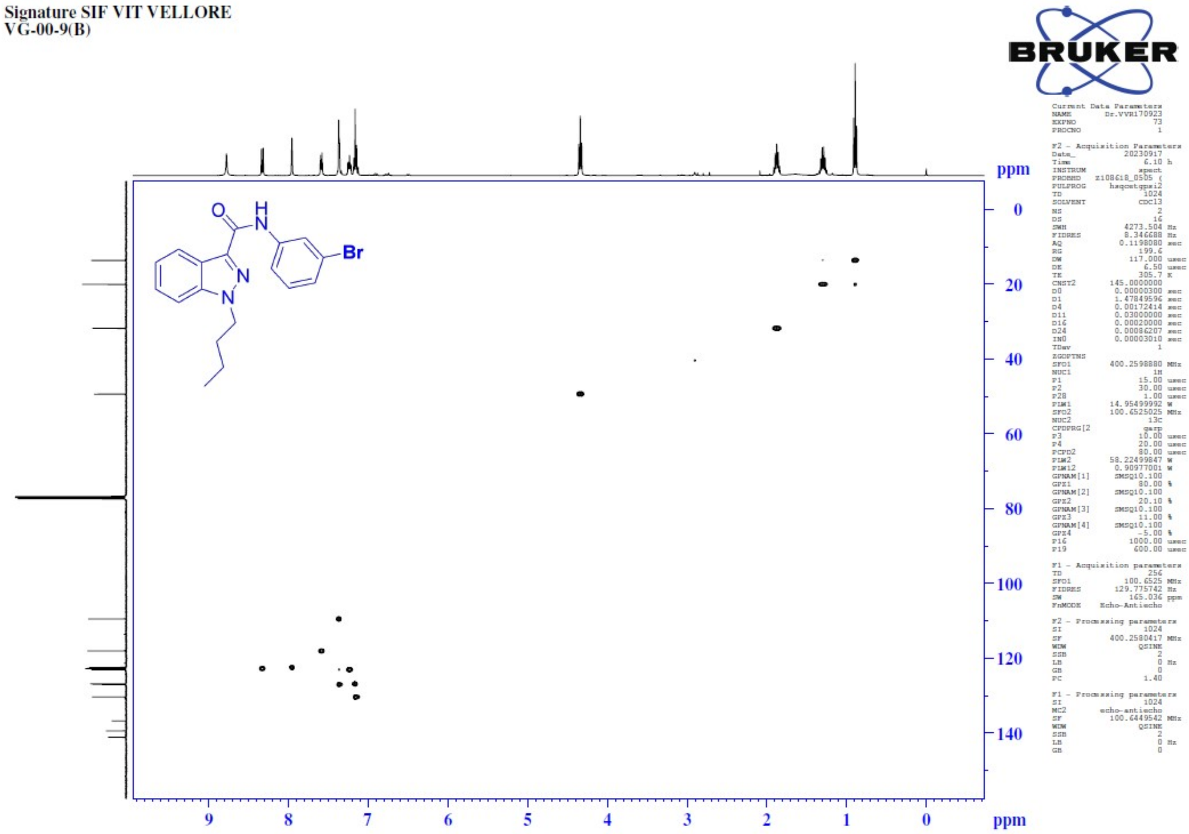

FT-IR spectrum of 1-butyl-N-(6-methylpyridine-2yl)-1H-indazole-3-carboxamide (8d).

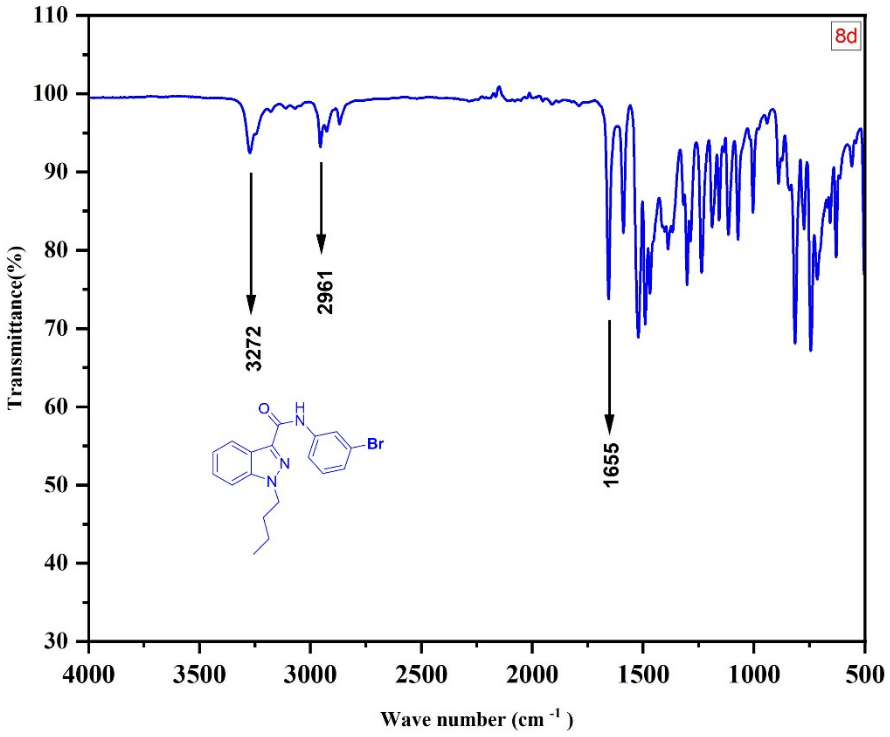

HRMS of 1-butyl-N-(6-methylpyridine-2yl)-1H-indazole-3-carboxamide (8d).

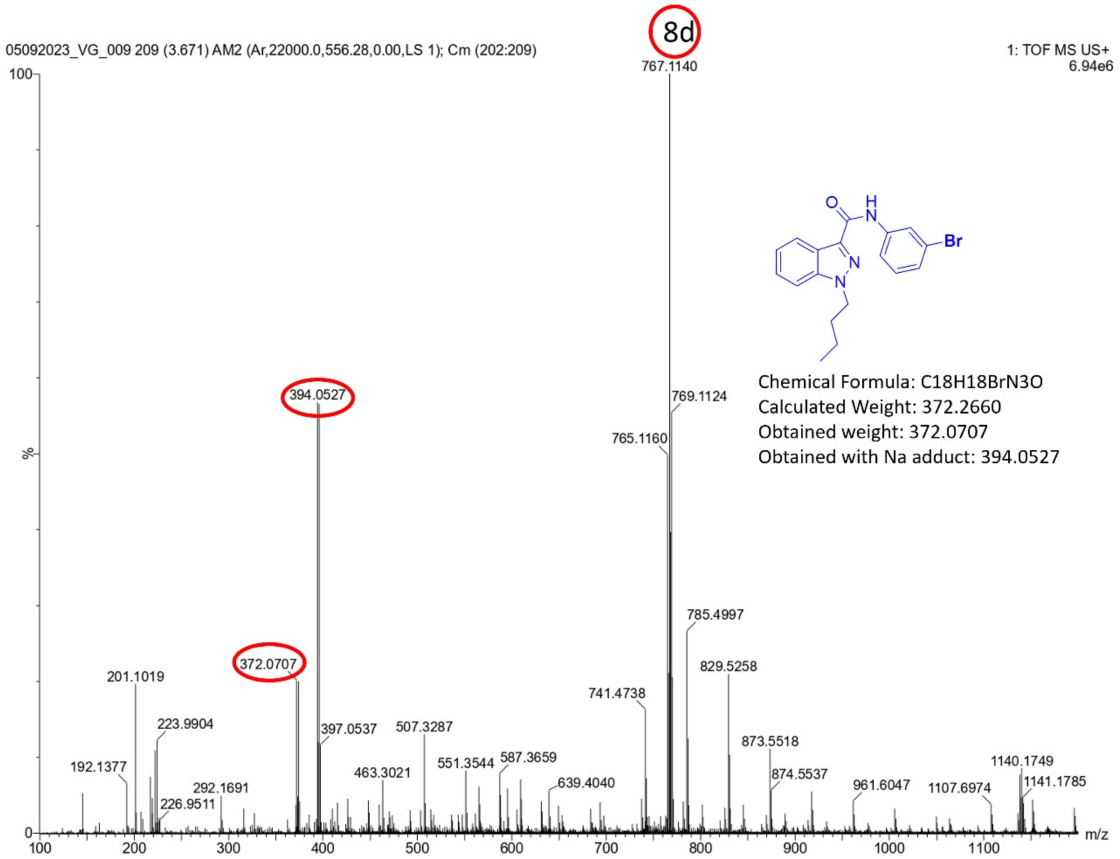

^{1}H-NMR [400MHz, DMSO-d_6] spectrum of 2-(1-butyl-1H-indazole -3-carboxamido)-5-iodobenzoic acid (8e).

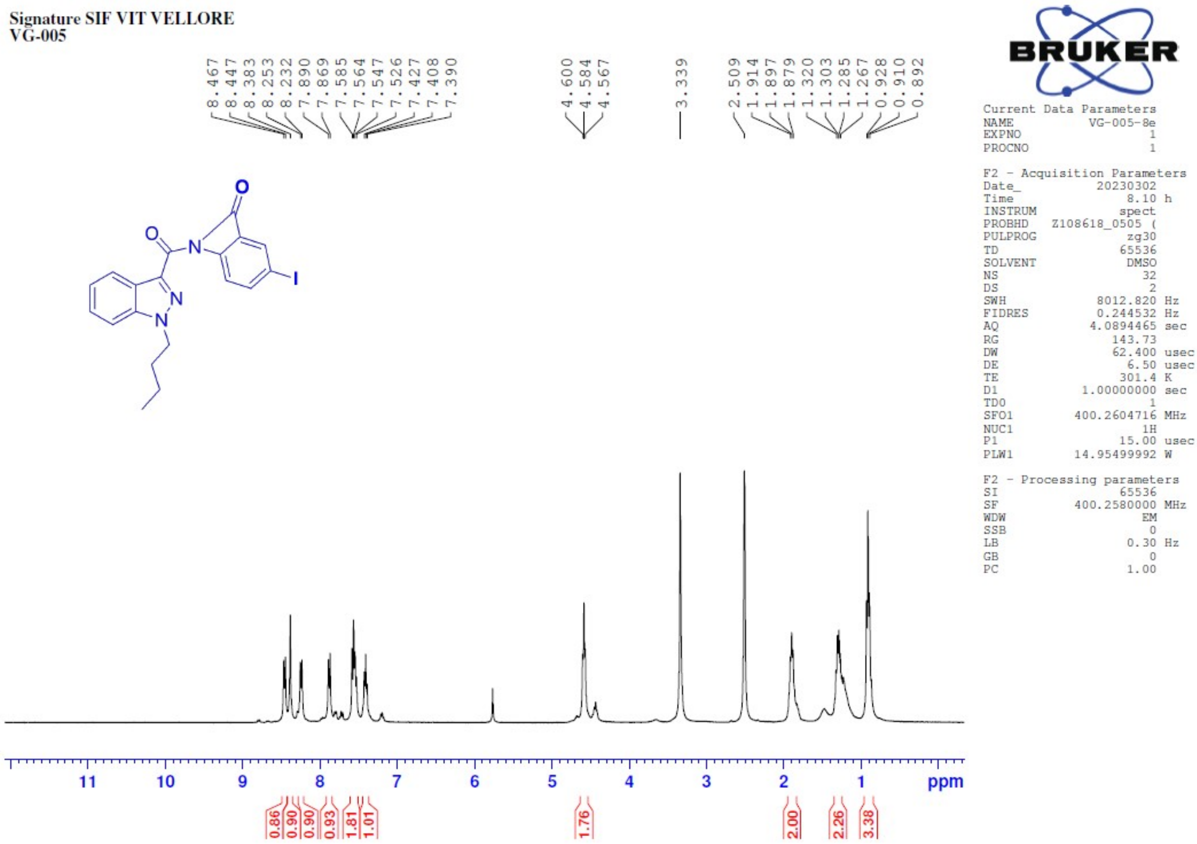

^{13}C-NMR [100MHz, DMSO-d$_6$] spectrum of 2-(1-butyl-1H-indazole -3-carboxamido)-5-iodobenzoic acid (8e).

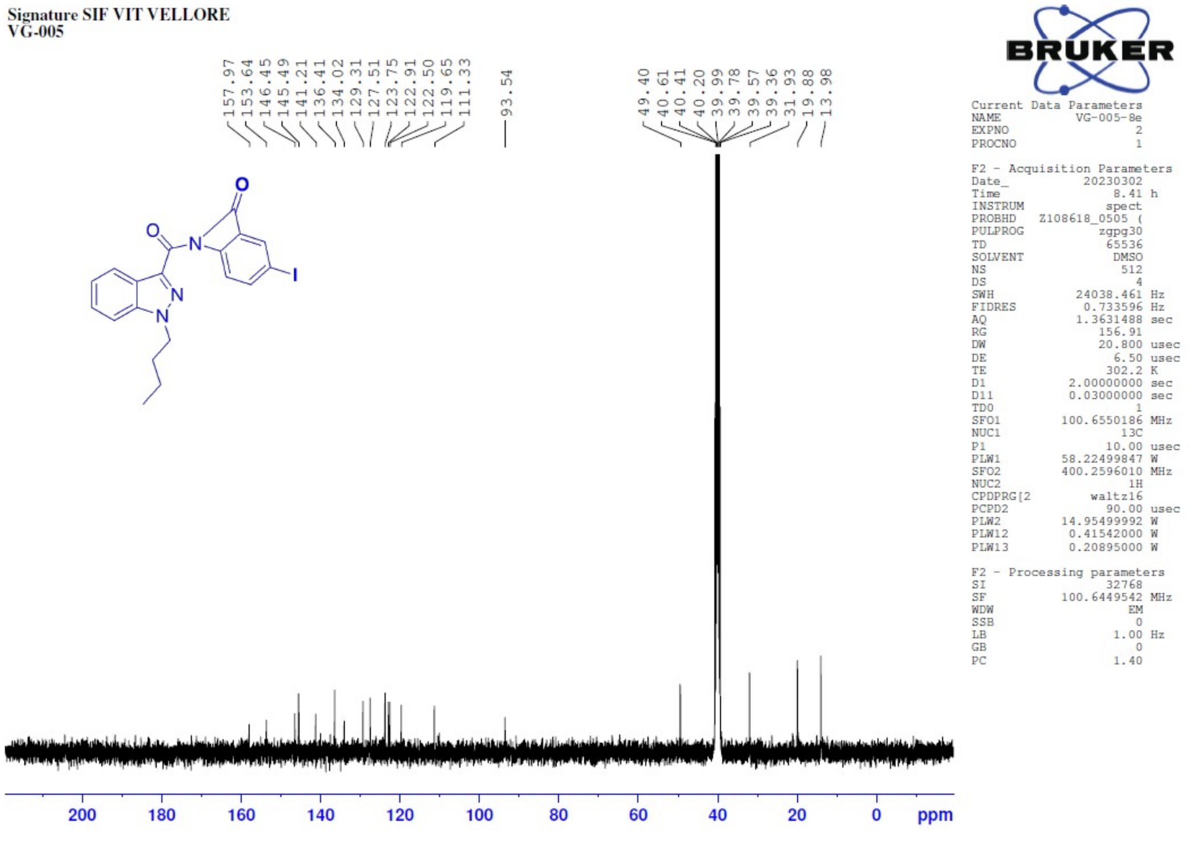

135-DEPT-NMR [100MHz, DMSO-d$_6$] spectrum of 2-(1-butyl-1H-indazole -3-carboxamido)-5-iodobenzoic acid (8e).

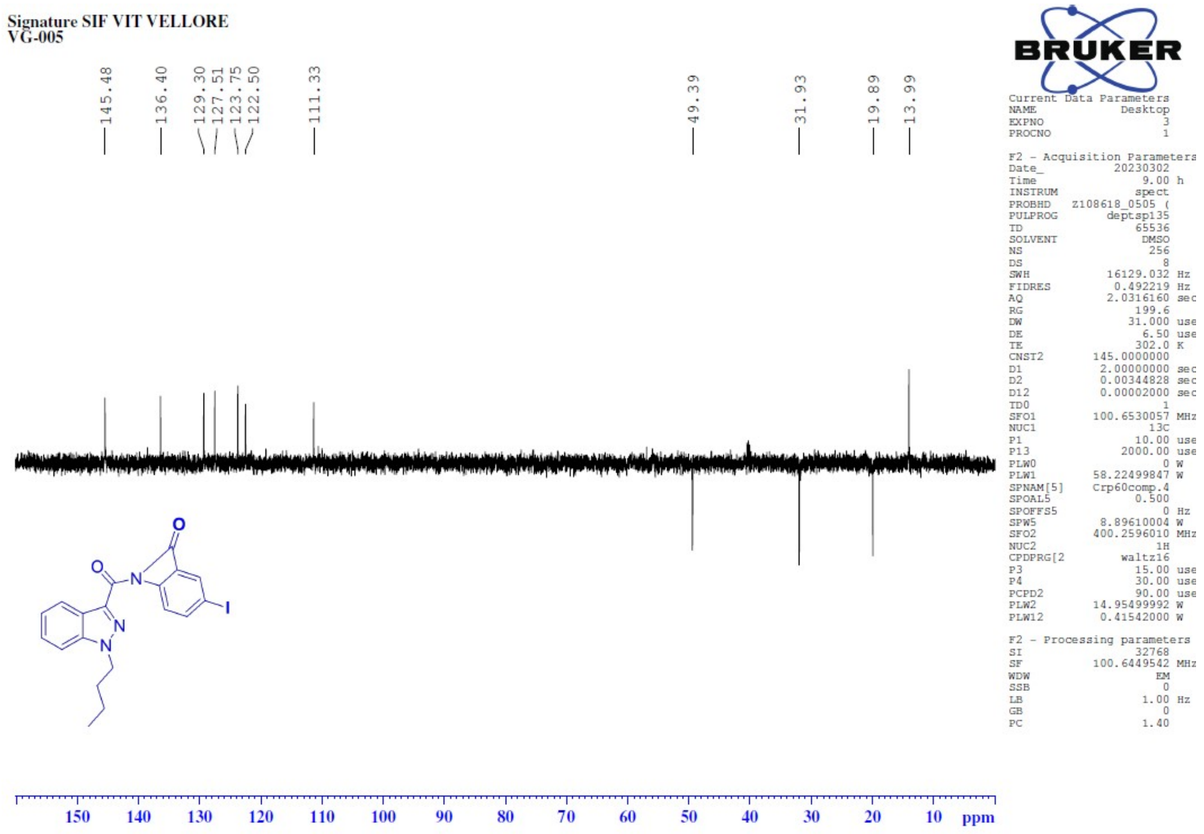

COSY-NMR [400MHz, DMSO-d$_6$] spectrum of 2-(1-butyl-1H-indazole -3-carboxamido)-5-iodobenzoic acid (8e).

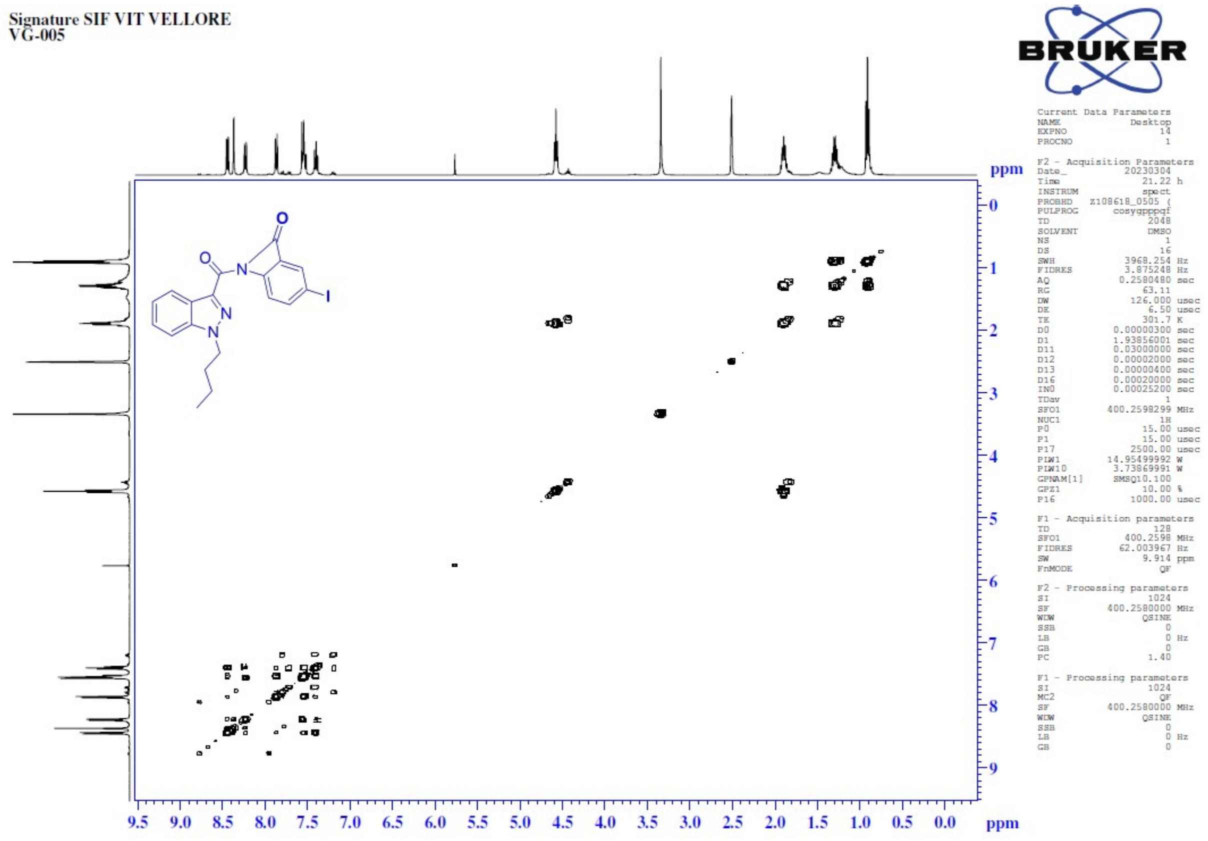

HSQC-NMR [400MHz, DMSO-d$_6$] spectrum of 2-(1-butyl-1H-indazole -3-carboxamido)-5-iodobenzoic acid (8e).

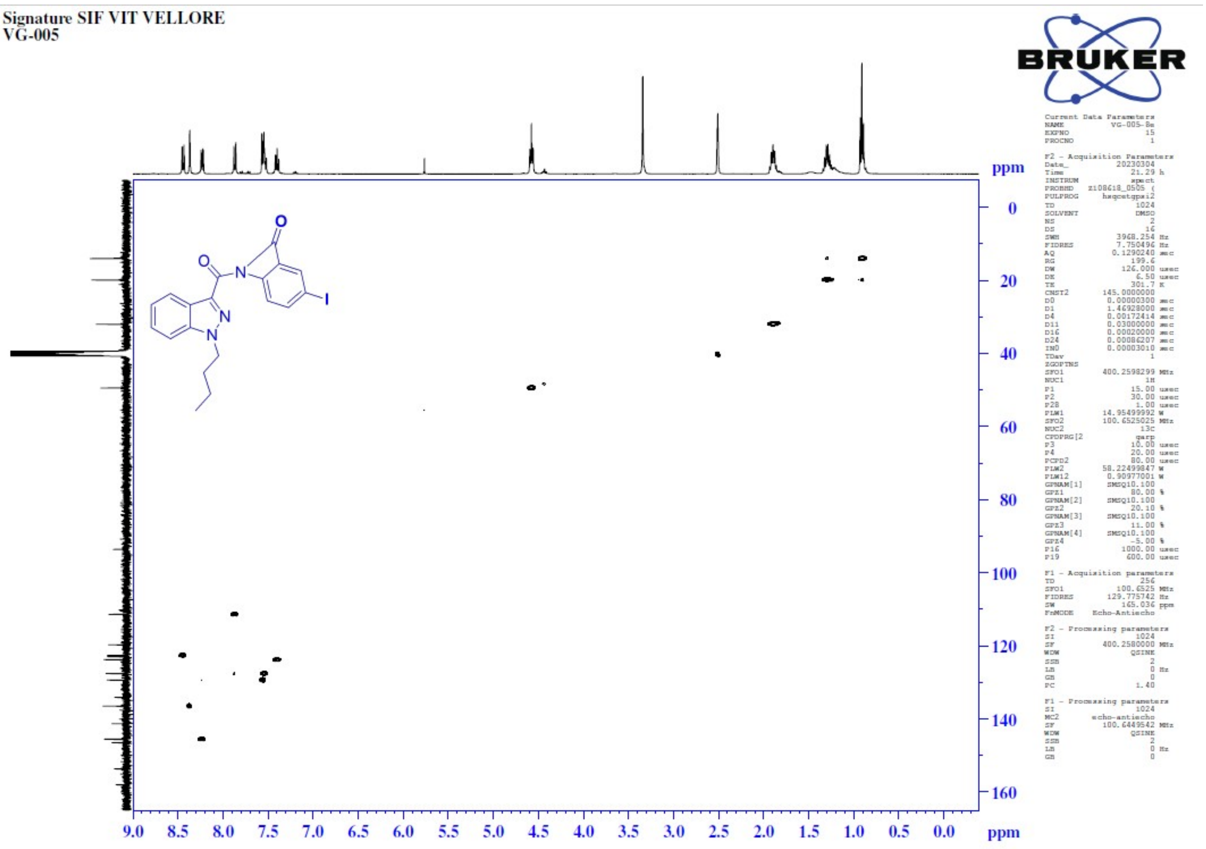

FT-IR spectrum of 2-(1-butyl-1H-indazole -3-carboxamido)-5-iodobenzoic acid (8e).

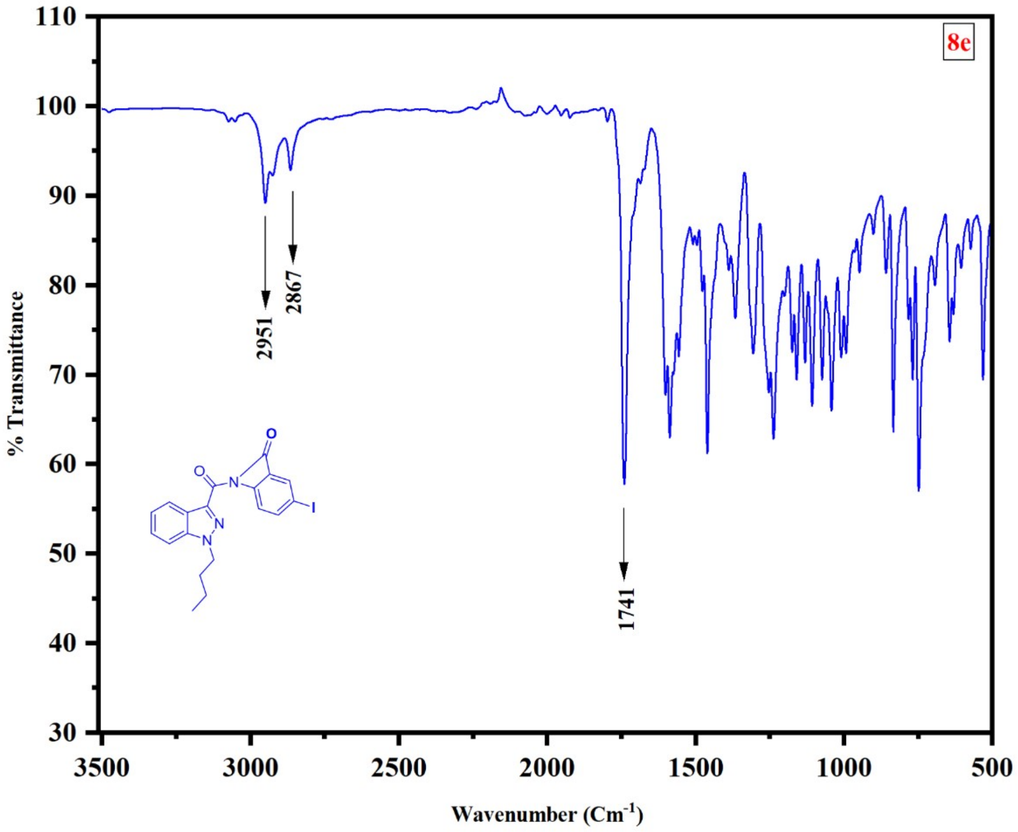

HRMS of 2-(1-butyl-1H-indazole -3-carboxamido)-5-iodobenzoic acid (8e).

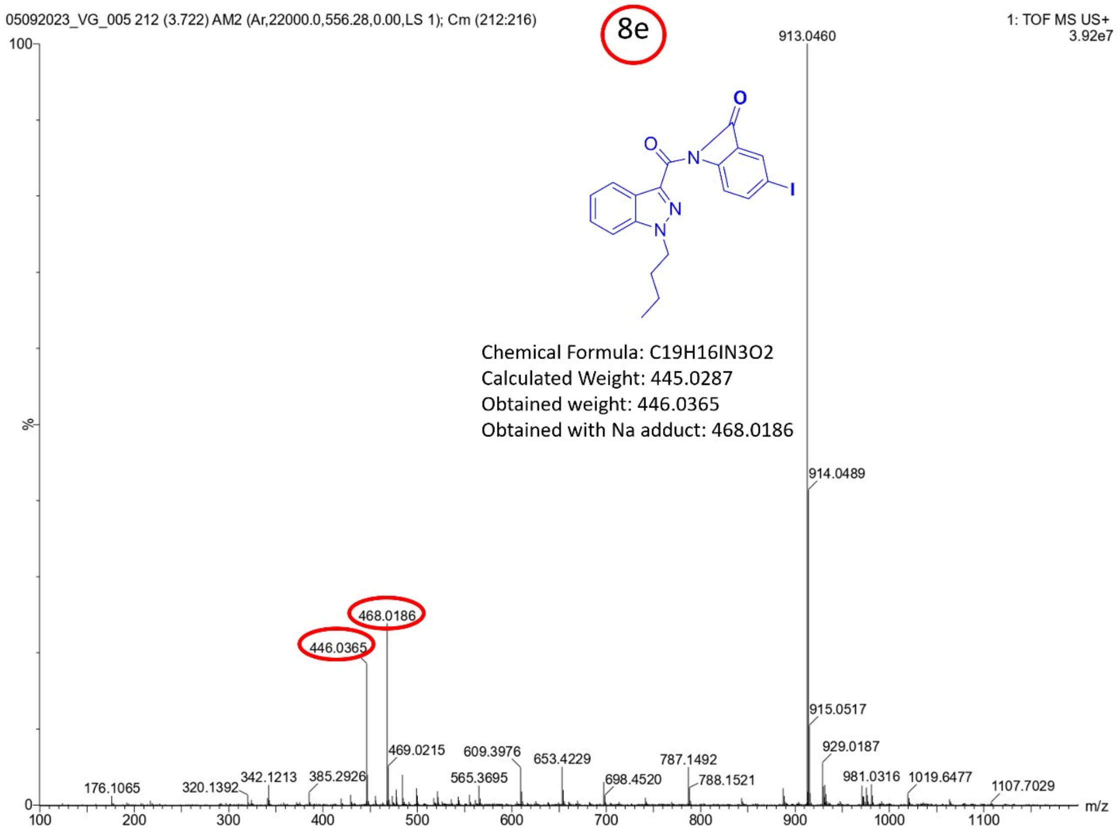

^{1}H-NMR [400MHz, DMSO-d$_6$] spectrum of 1-butyl-N-(4-(phenylamino) phenyl)-1H-indazole-3-carboxamide (8f).

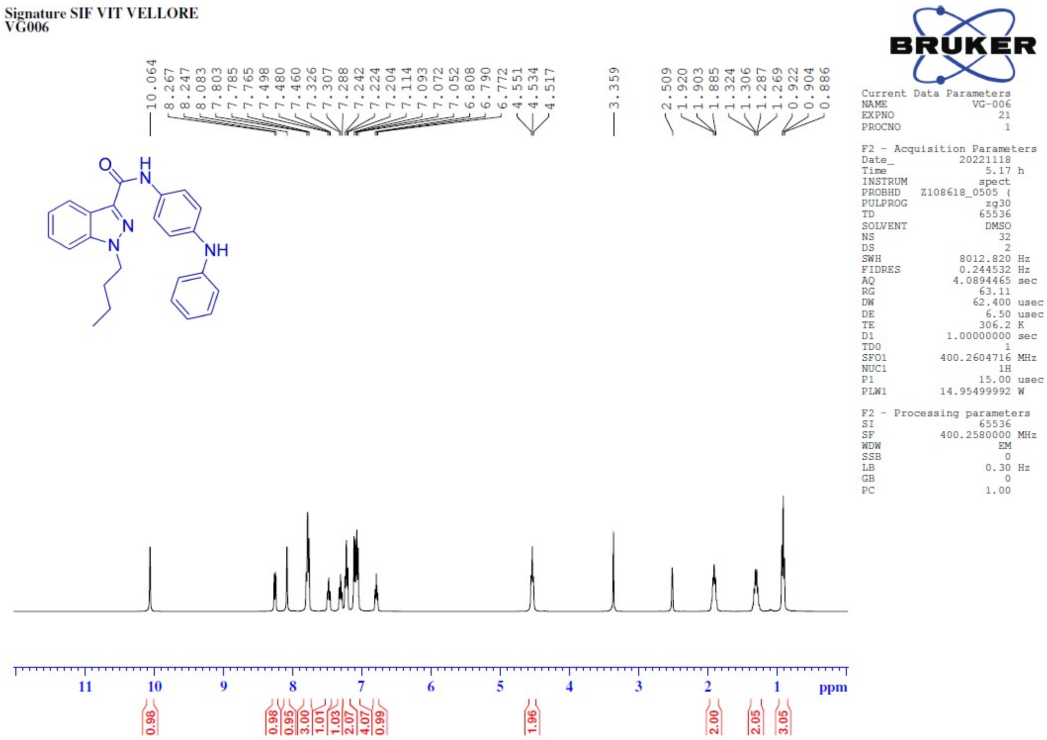

^{13}C-NMR [100MHz, DMSO-d_6] spectrum of 1-butyl-N-(4-(phenylamino) phenyl)-1H-indazole-3-carboxamide (8f).

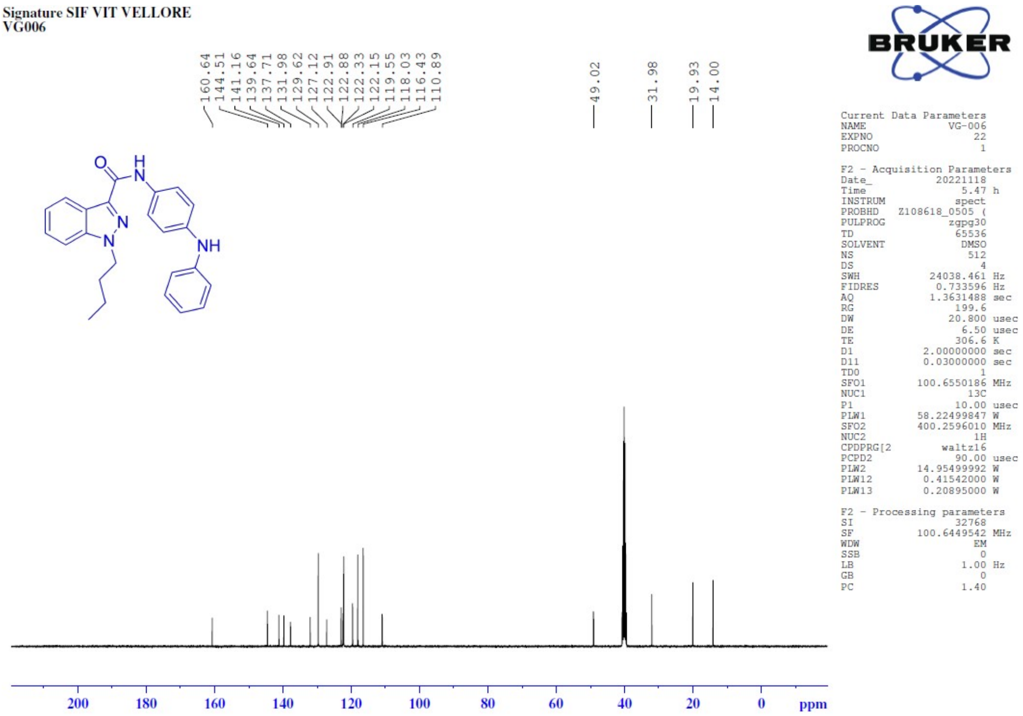

135-DEPT-NMR [100MHz, DMSO-d_6] spectrum of 1-butyl-N-(4-(phenylamino)phenyl)-1H-indazole-3-carboxamide (8f).

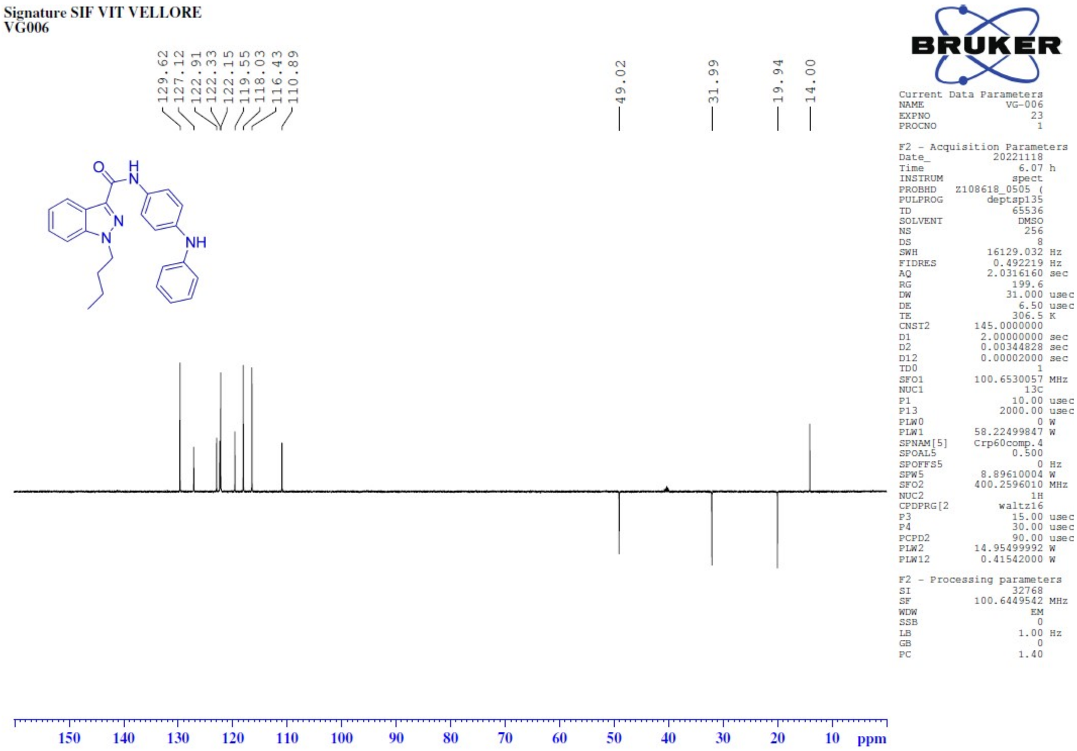

COSY-NMR [400MHz, DMSO-d_6] spectrum of 1-butyl-N-(4-(phenylamino)phenyl)-1H-indazole-3-carboxamide (8f).

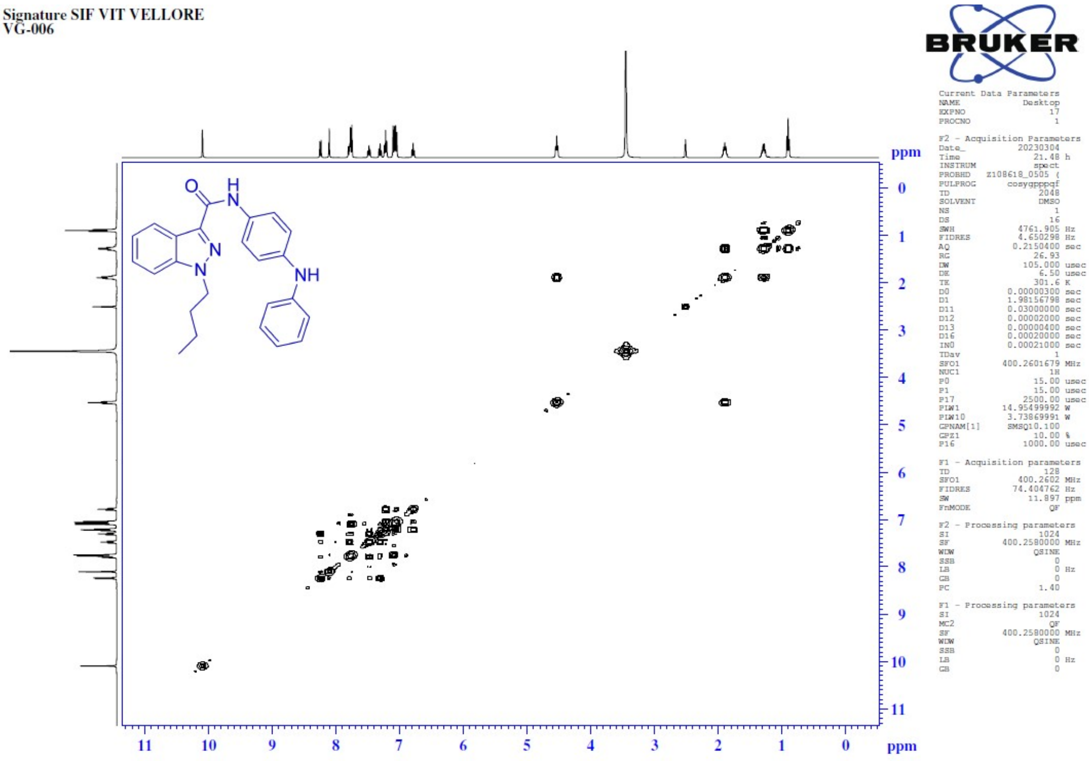

HSQC-NMR [400MHz, DMSO-d_6] spectrum of 1-butyl-N-(4-(phenylamino)phenyl)-1H-indazole-3-carboxamide (8f).

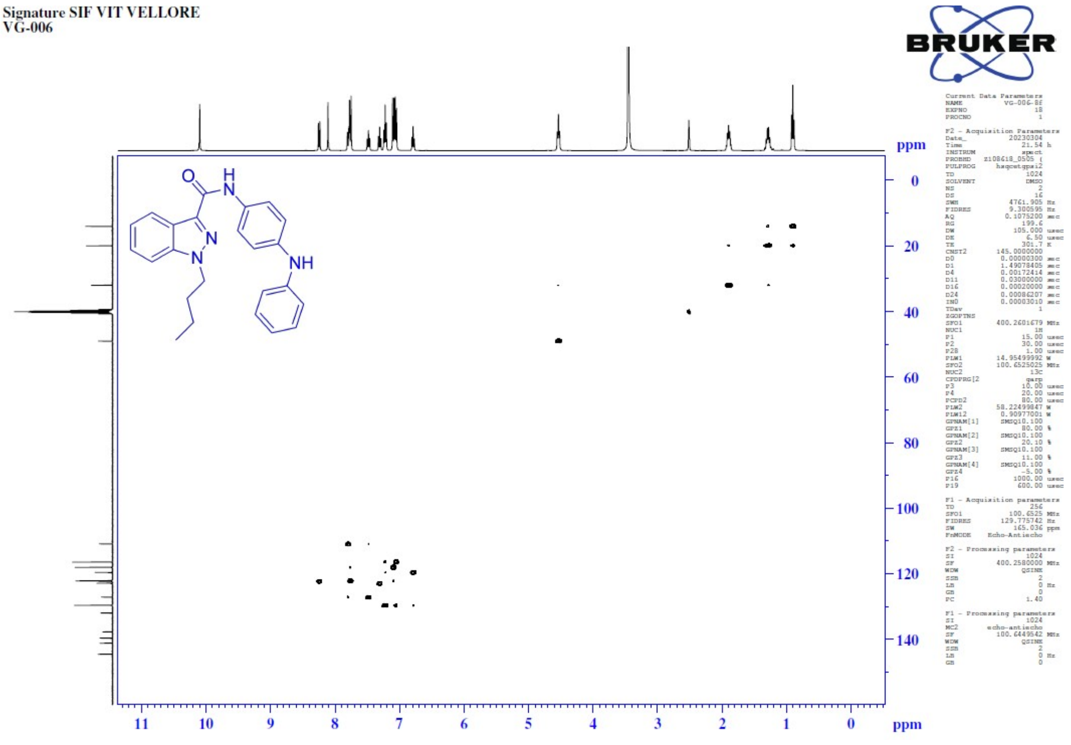

FT-IR spectrum of 1-butyl-N-(4-(phenylamino)phenyl)-1H-indazole-3-carboxamide (8f).

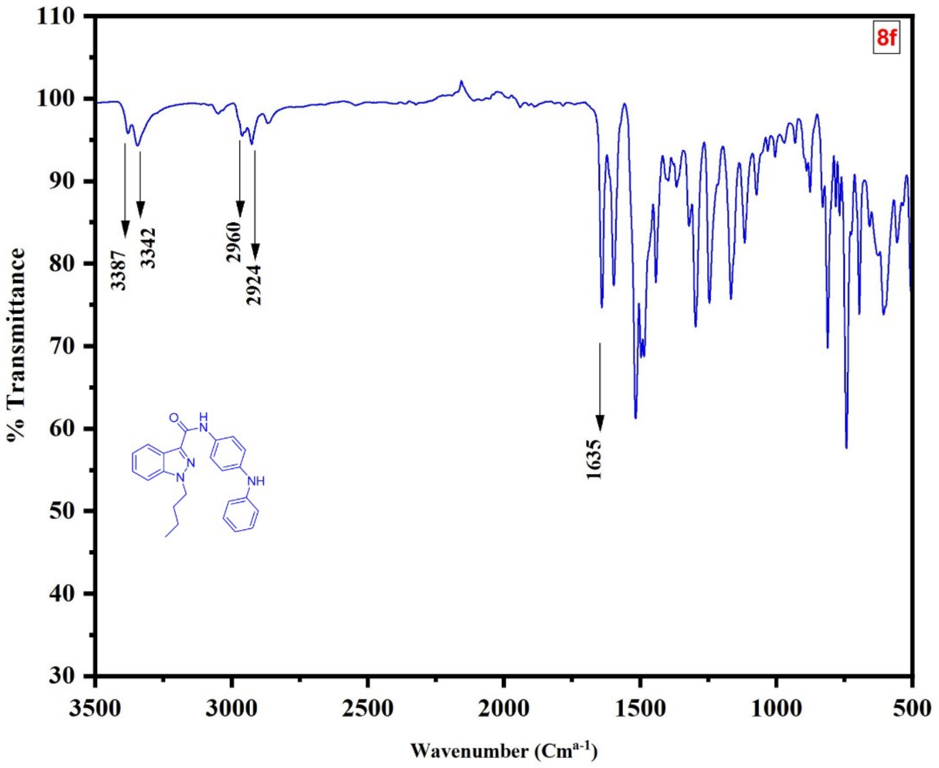

HRMS of 1-butyl-N-(4-(phenylamino)phenyl)-1H-indazole-3-carboxamide (8f).

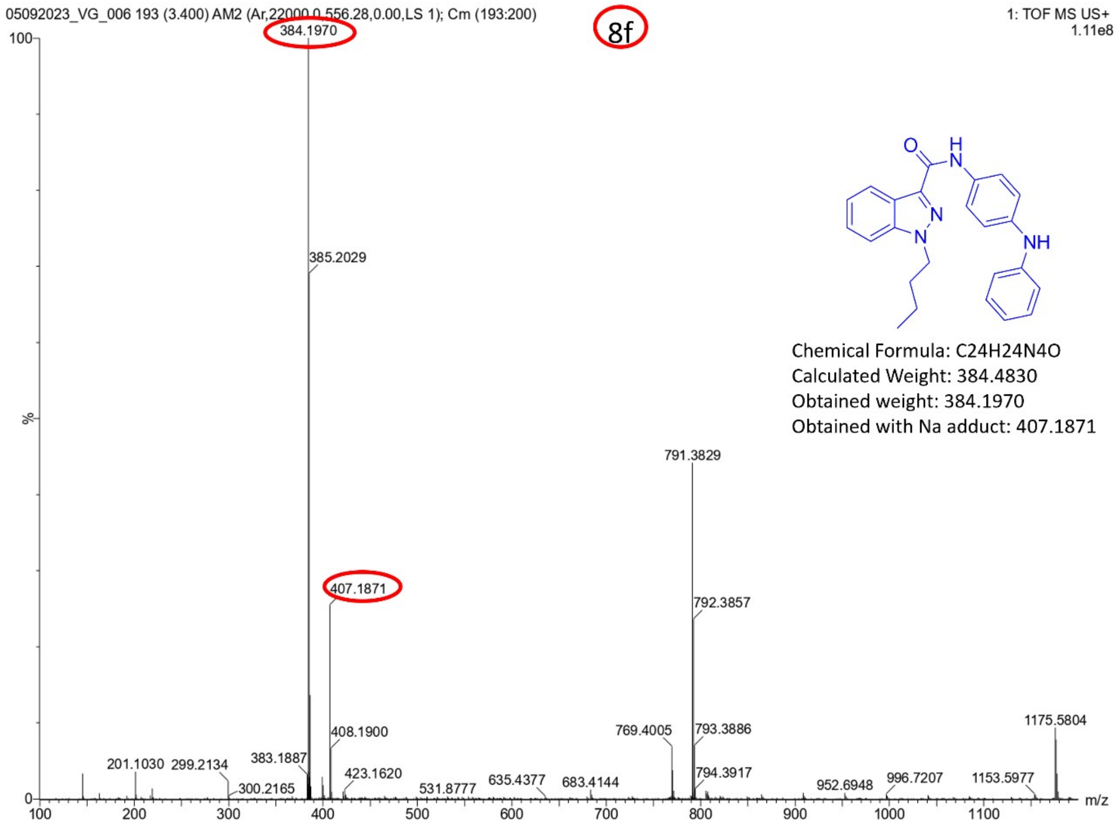

^{1}H-NMR [400MHz, DMSO-d_6] spectrum of 1-butyl-N-(4-nitophenyl)-1H-indazole-3-carboxamide (8g).

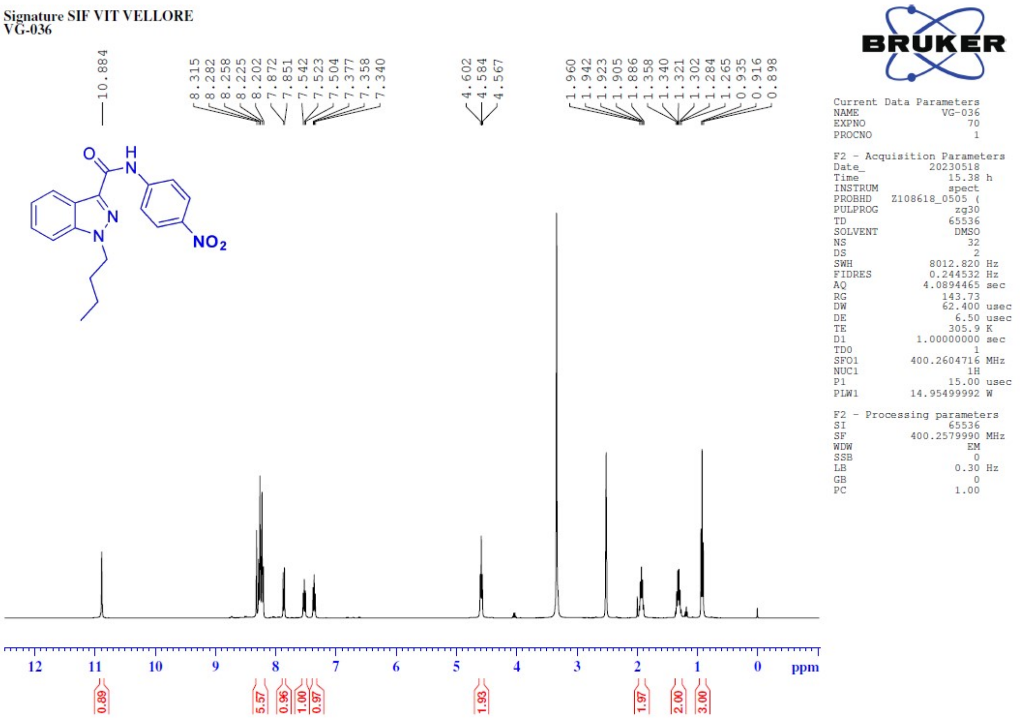

^{13}C-NMR [100MHz, DMSO-d_6] spectrum of 1-butyl-N-(4-nitophenyl)-1H-indazole-3-carboxamide (8g).

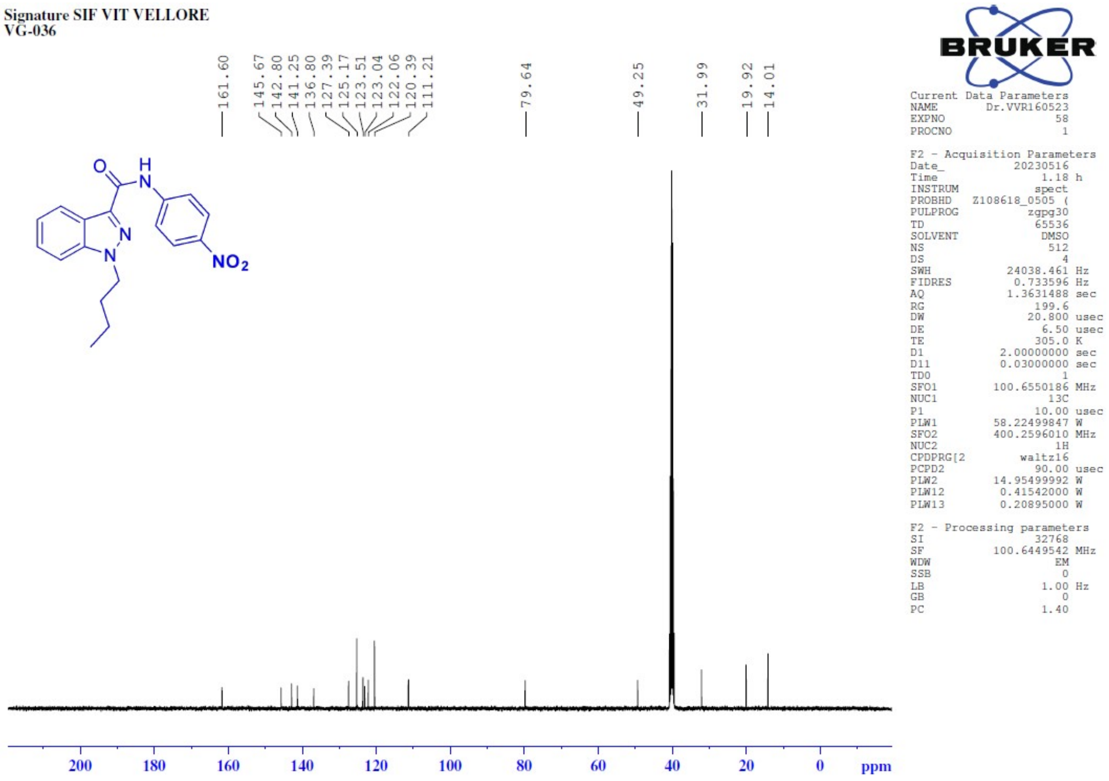

135-DEPT-NMR [400MHz, DMSO-d_6] spectrum of 1-butyl-N-(4-nitophenyl)-1H-indazole-3-carboxamide (8g).

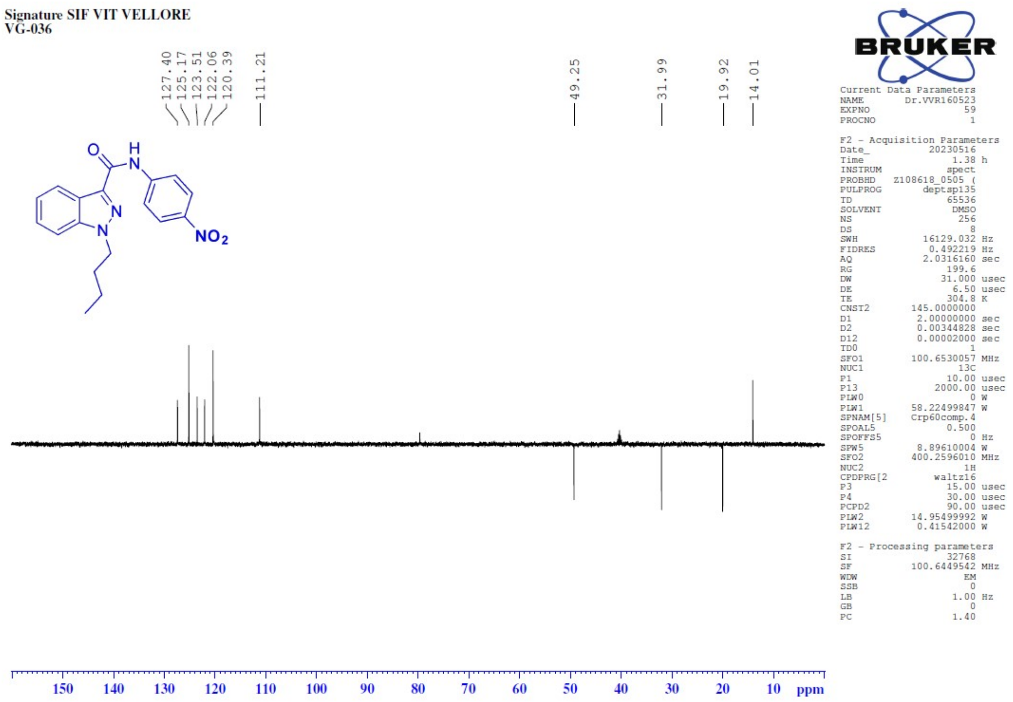

FT-IR spectrum of 1-butyl-N-(4-nitophenyl)-1H-indazole-3-carboxamide (8g).

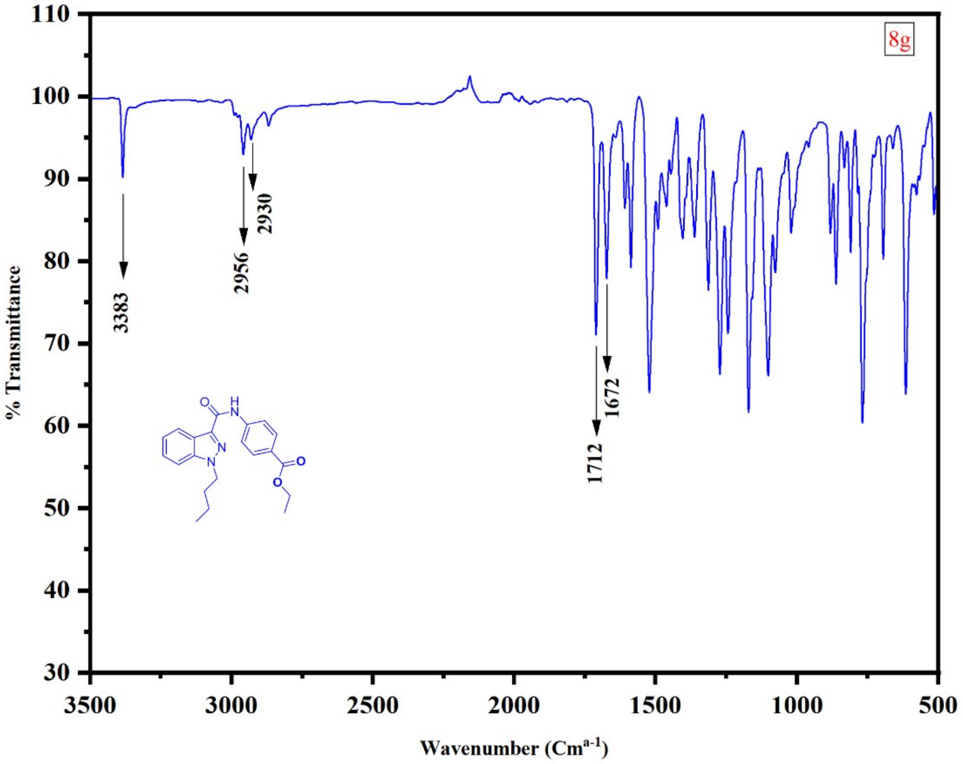

HRMS of 1-butyl-N-(4-nitophenyl)-1H-indazole-3-carboxamide (8g).

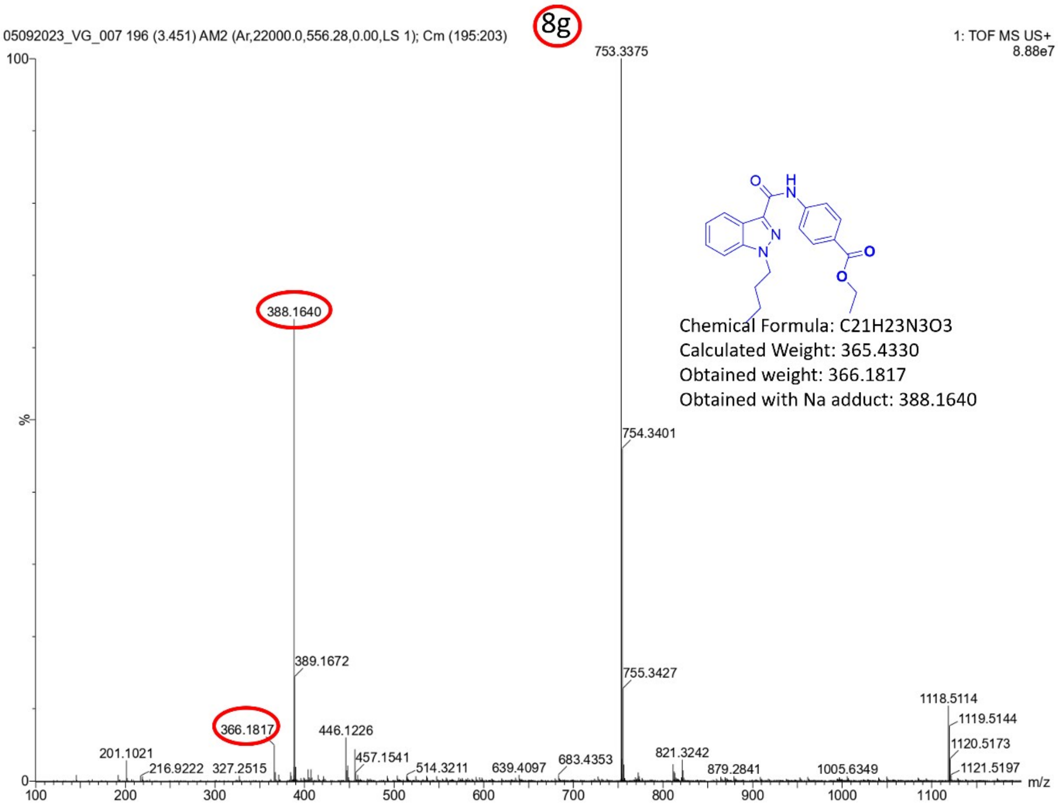

^{1}H-NMR [400MHz, DMSO-d_6] spectrum of 1-butyl-N-(4-nitrophenyl)-1H-indazole-3-carbohydrazide (8h).

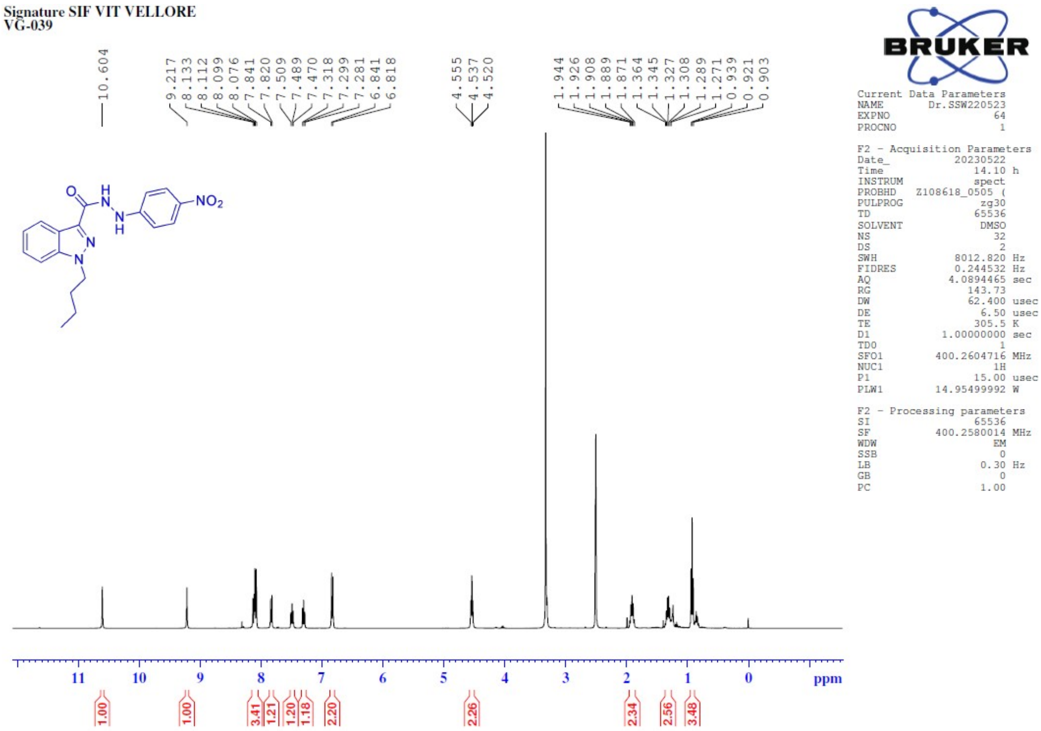

^{13}C-NMR [100MHz, DMSO-d_6] spectrum of 1-butyl-N-(4-nitro phenyl)-1H-indazole-3-carbohydrazide (8h).

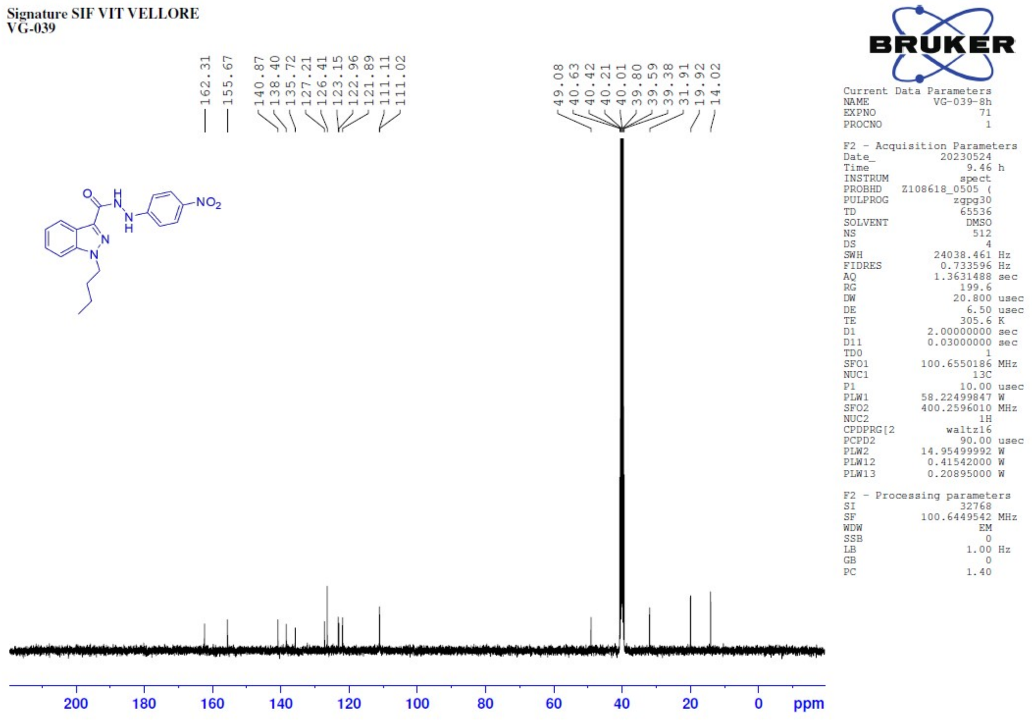

135-DEPT-NMR [100MHz, DMSO-d_6] spectrum of 1-butyl-N-(4-nitrophenyl)-1H-indazole-3-carbohydrazide (8h).

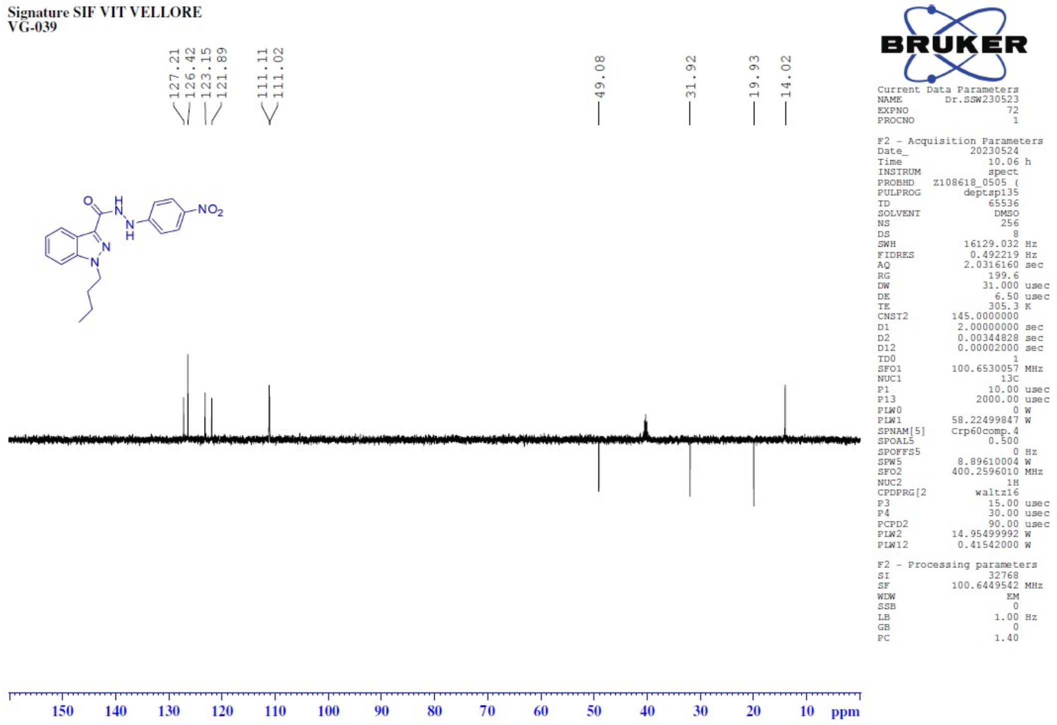

FT-IR spectrum of 1-butyl-N-(4-nitrophenyl)-1H-indazole-3-carbohydrazide (8h).

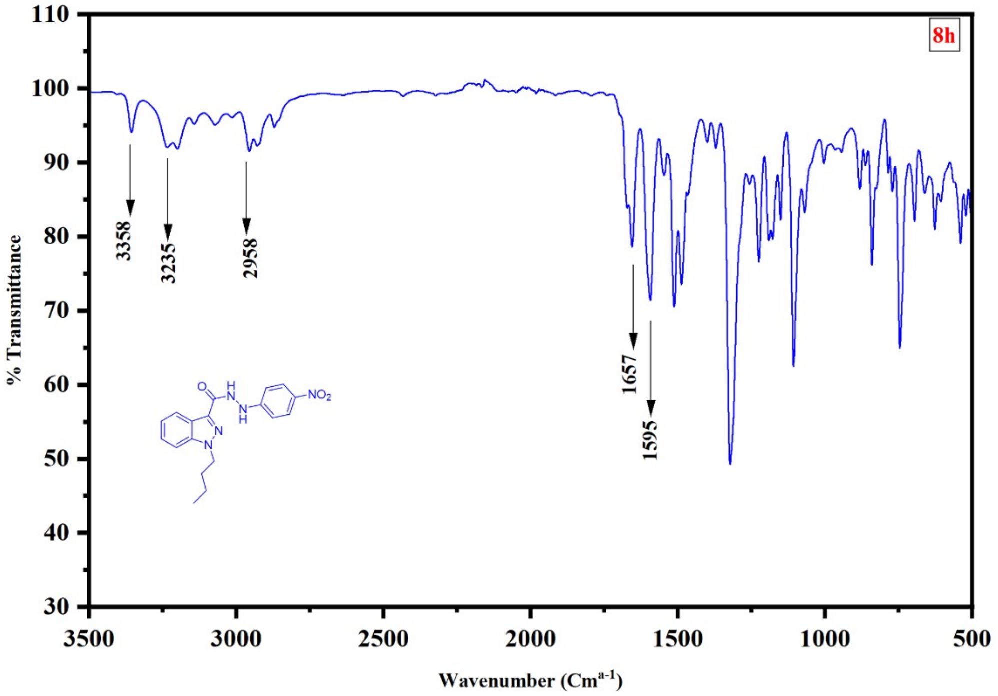

HRMS of 1-butyl-N-(4-nitrophenyl)-1H-indazole-3-carbohydrazide (8h).

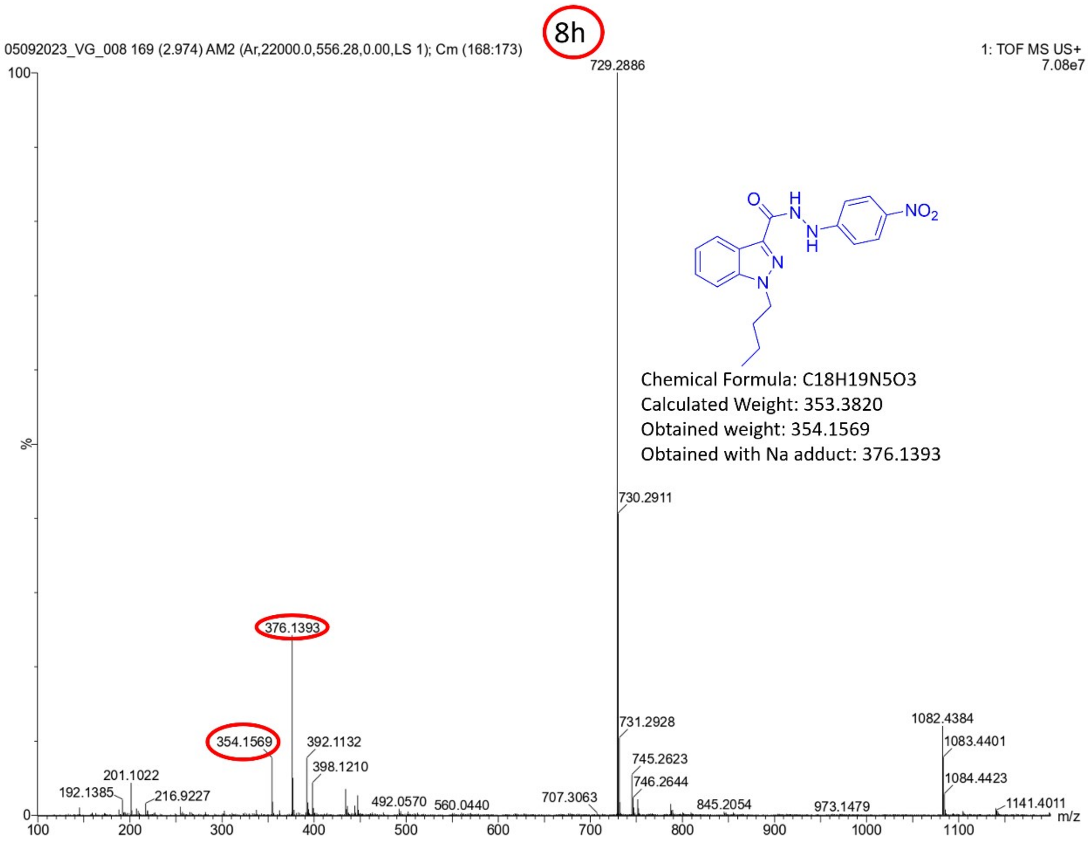

^{1}H-NMR [400MHz, DMSO-d_6] spectrum of N-(4-Bromophenyl)-1-butyl-1H-indazole-3-carboxamide (8i).

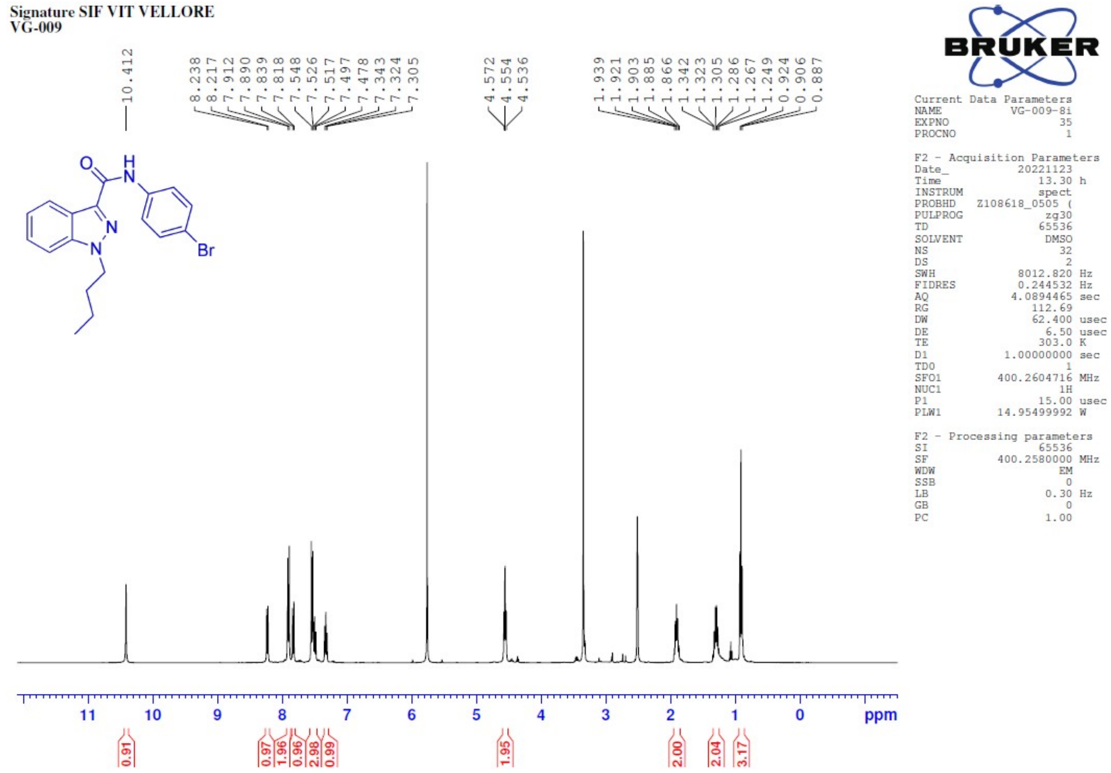

^{13}C-NMR [100MHz, DMSO-d$_6$] spectrum of N-(4-Bromophenyl)-1-butyl-1H-indazole-3-carboxamide (8i).

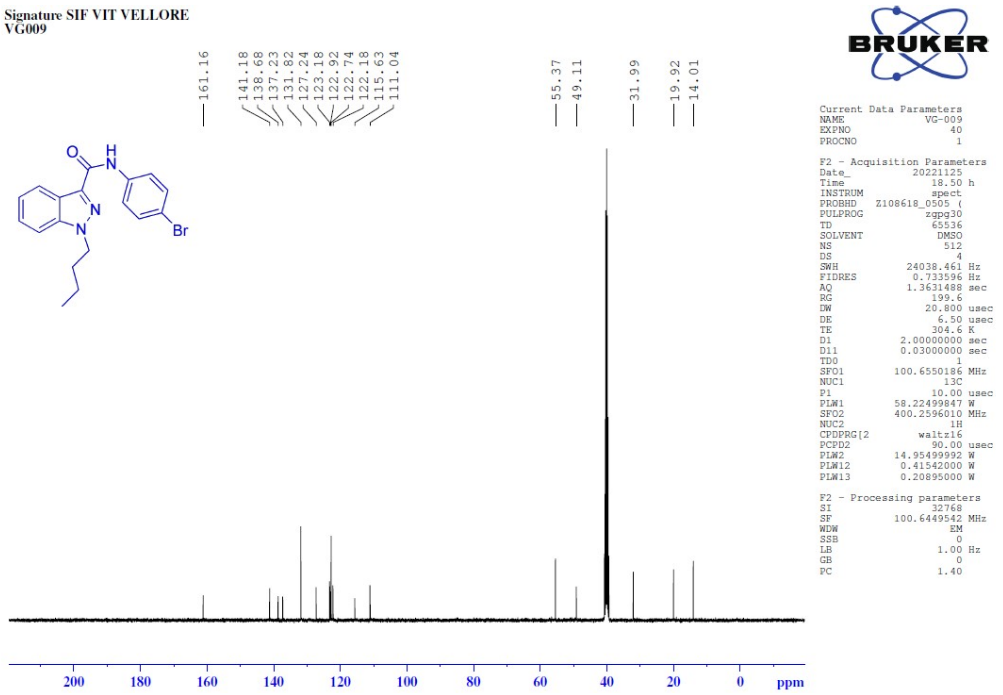

135-DEPT-NMR [100MHz, DMSO-d$_6$] spectrum of N-(4-Bromophenyl)-1-butyl-1H-indazole-3-carboxamide (8i).

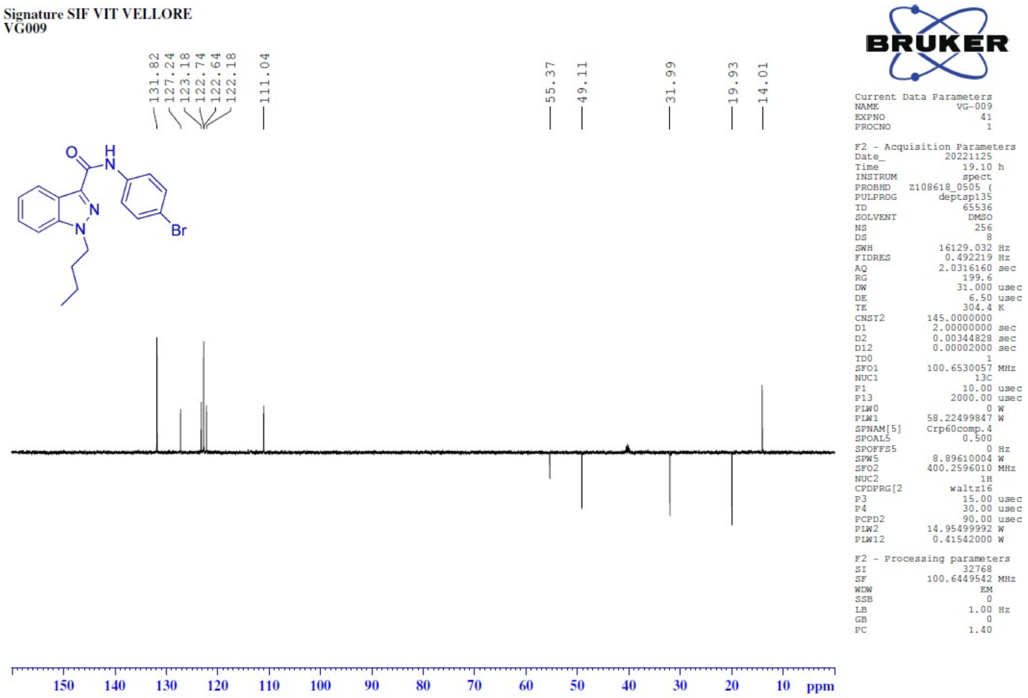

COSY-NMR [400MHz, DMSO-d$_6$] spectrum of N-(4-Bromophenyl)-1-butyl-1H-indazole-3-carboxamide (8i).

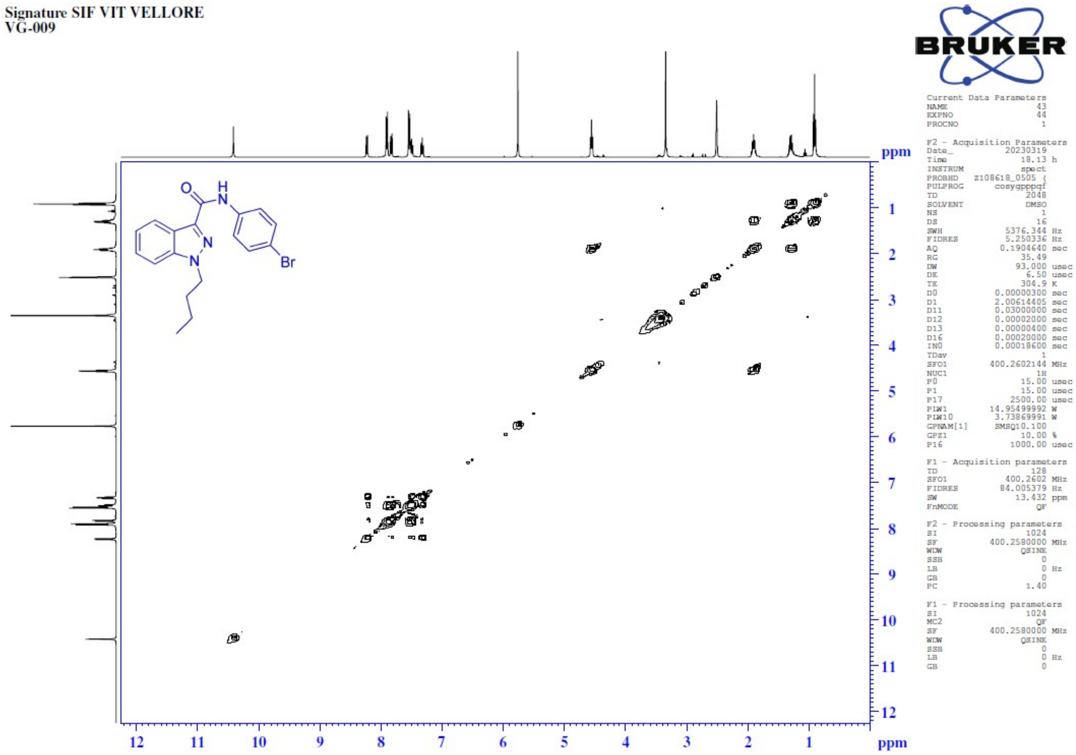

HSQC-NMR [400MHz, DMSO-d$_6$] spectrum of N-(4-Bromophenyl)-1-butyl-1H-indazole-3-carboxamide (8i).

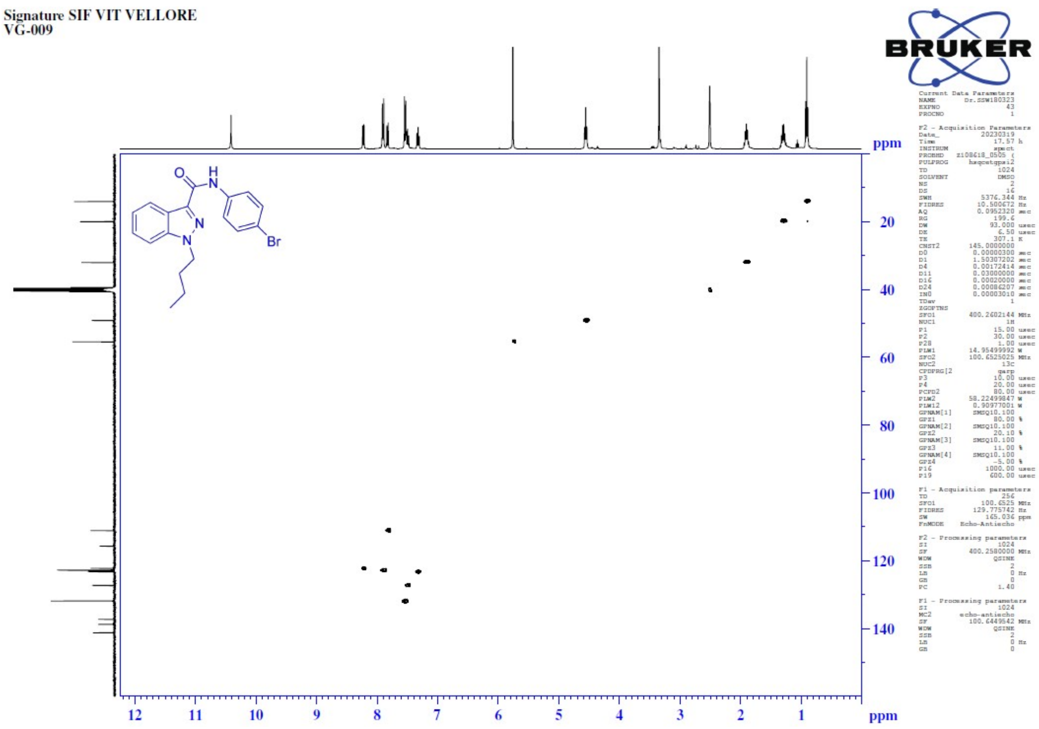

FT-IR spectrum of N-(4-Bromophenyl)-1-butyl-1H-indazole-3-carboxamide (8i).

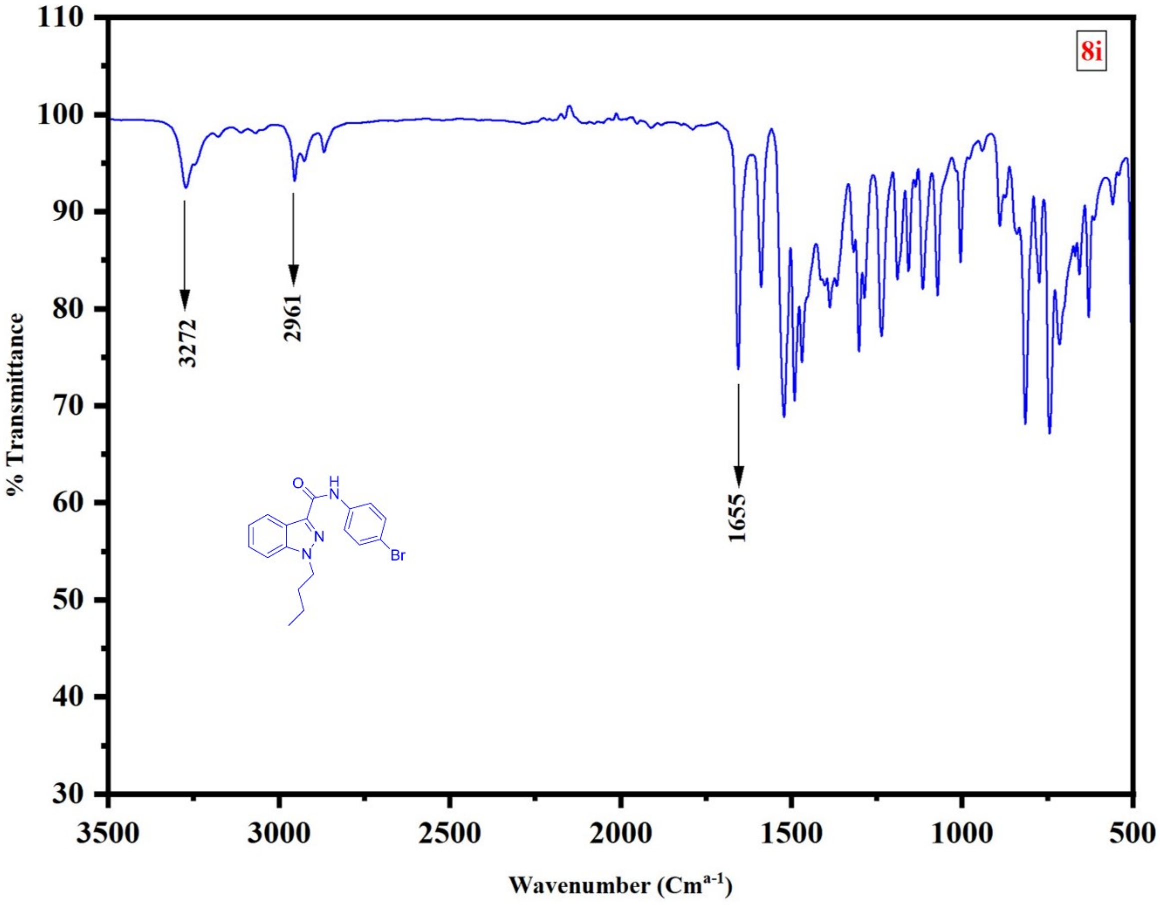

HRMS of N-(4-Bromophenyl)-1-butyl-1H-indazole-3-carboxamide (8i).

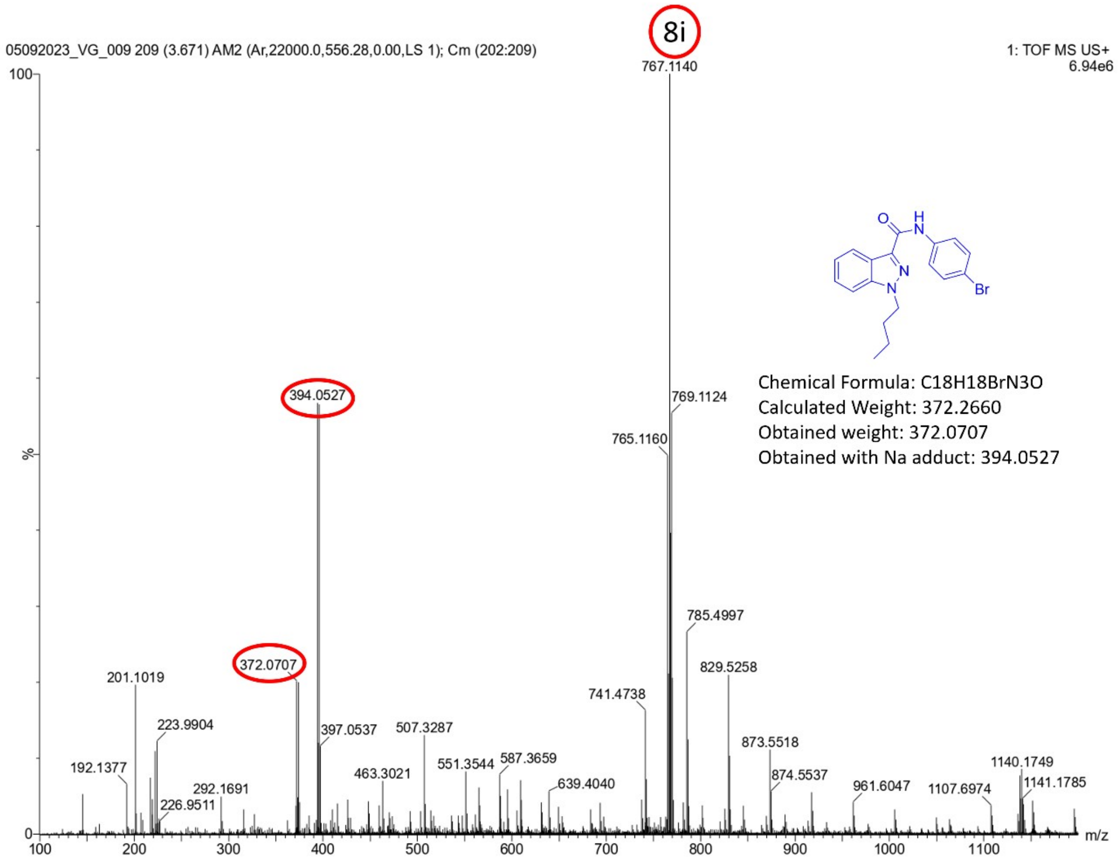

^{1}H-NMR [400MHz, DMSO-d_6] spectrum of 1-butyl-N-(4-hydroxypheny)-1H-indazole-3-carboxamide (8j).

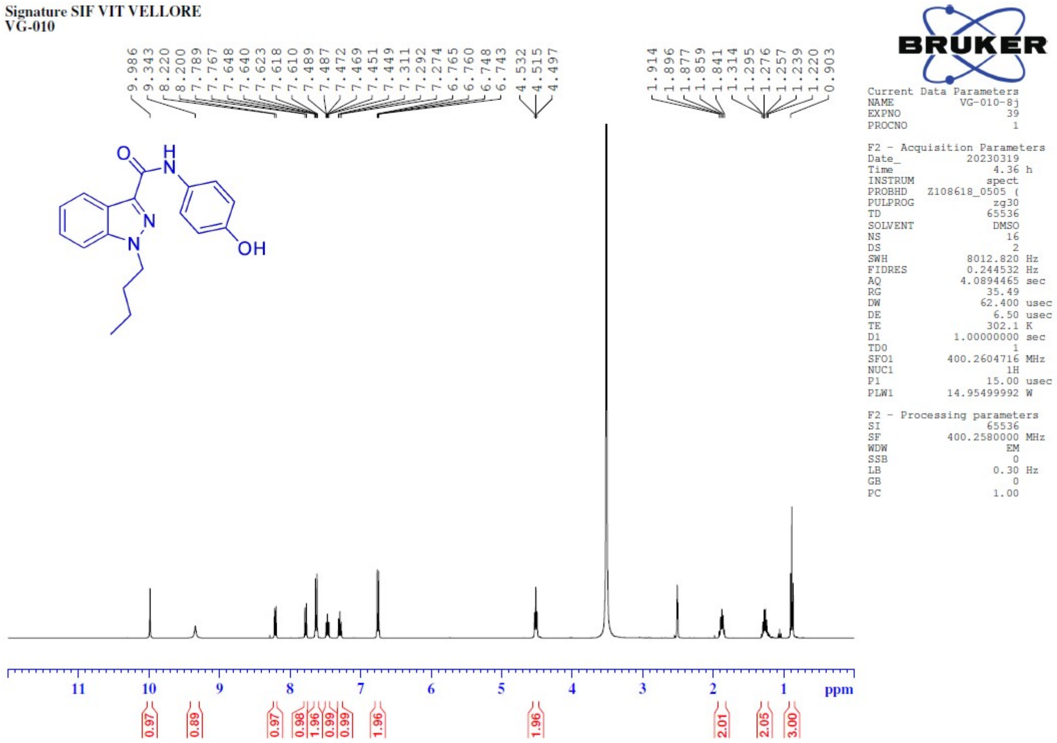

^{13}C-NMR [100MHz, DMSO-d_6] spectrum of 1-butyl-N-(4-hydroxypheny)-1H-indazole-3-carboxamide (8j).

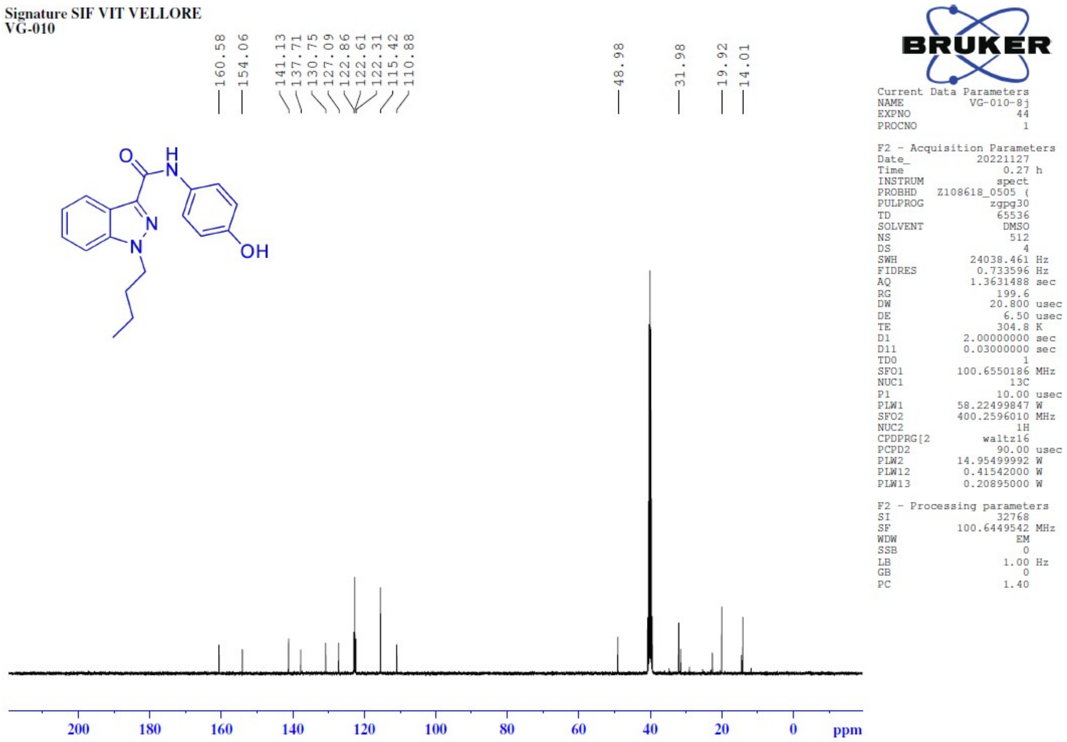

135-DEPT-NMR [100MHz, DMSO-d_6] spectrum of 1-butyl-N-(4-hydroxypheny)-1H-indazole-3-carboxamide (8j).

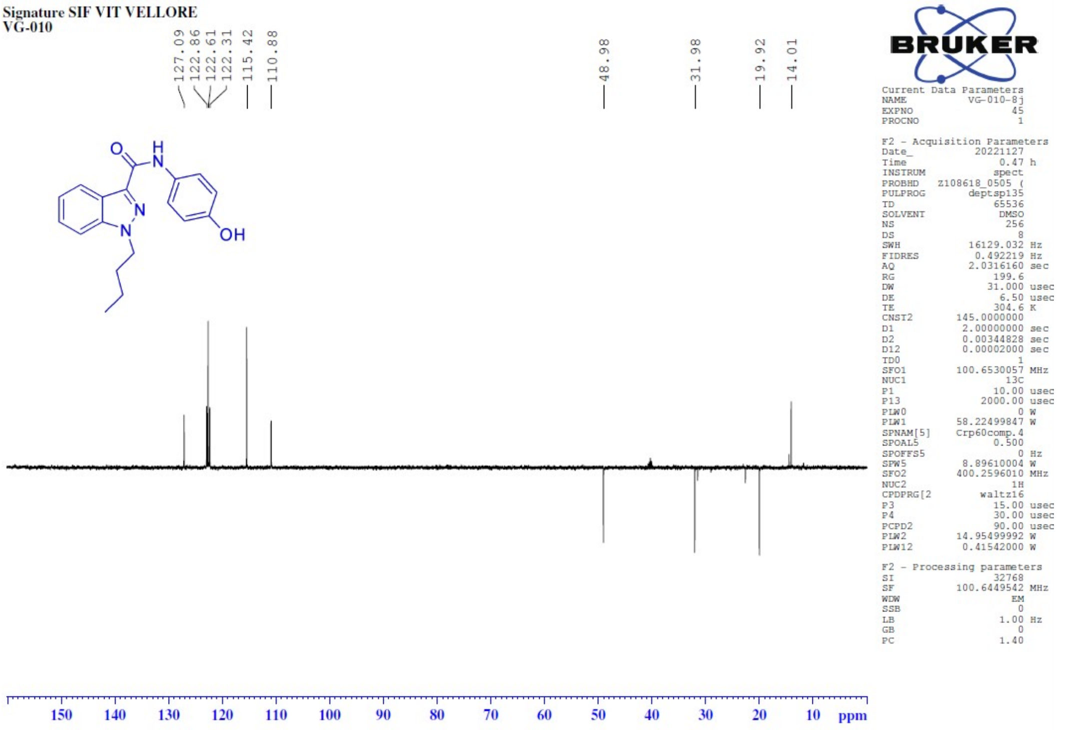

COSY-NMR [400MHz, DMSO-d_6] spectrum of 1-butyl-N-(4-hydroxypheny)-1H-indazole-3-carboxamide (8j).

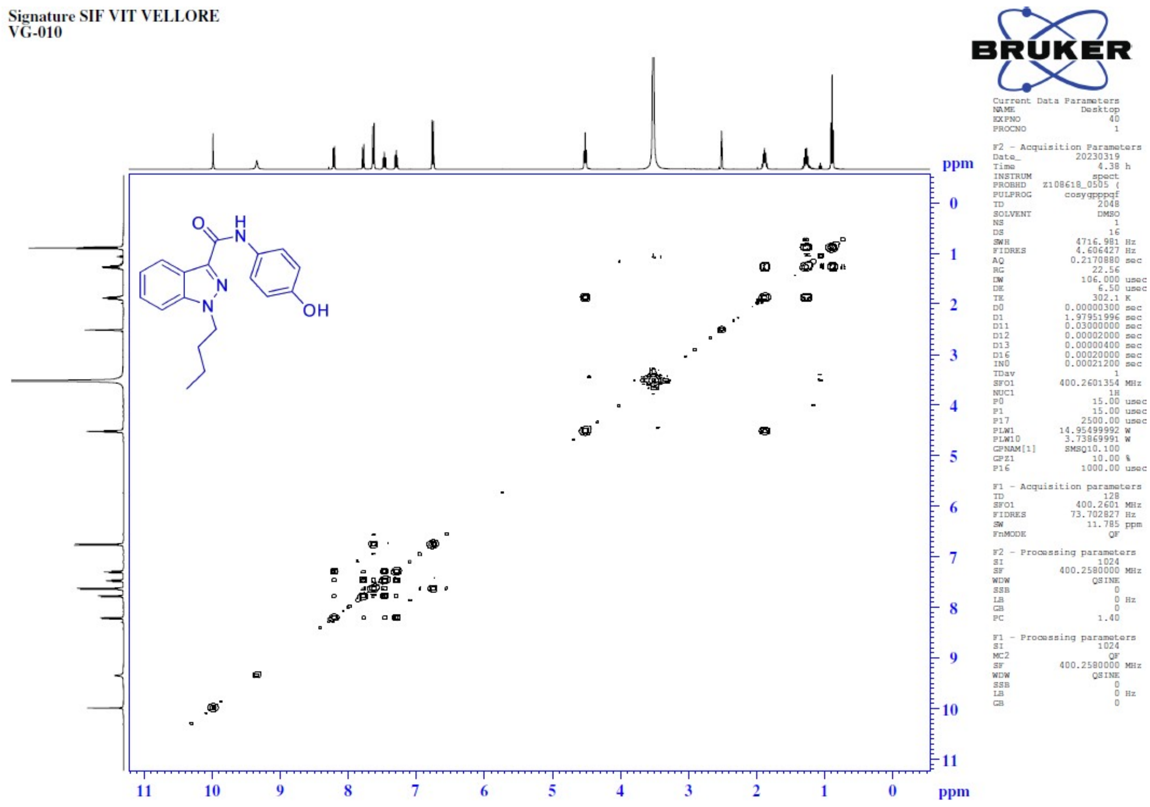

HSQC-NMR [400MHz, DMSO-d_6] spectrum of 1-butyl-N-(4-hydroxypheny)-1H-indazole-3-carboxamide (8j).

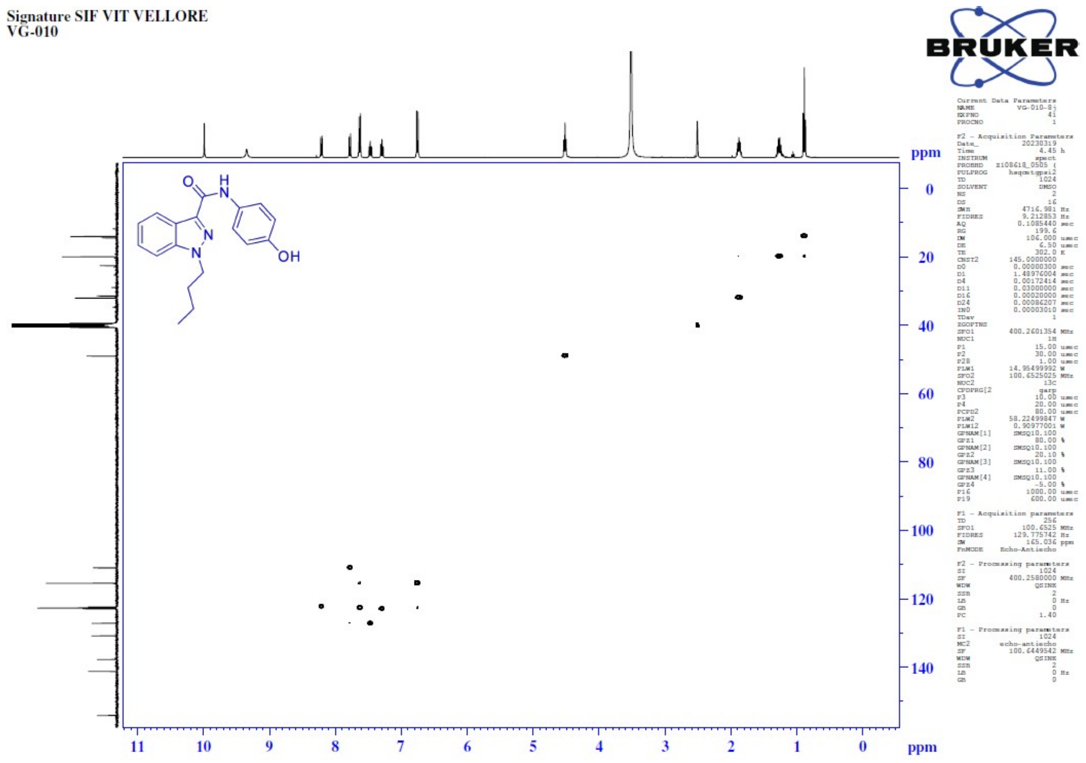

FT-IR spectrum of 1-butyl-N-(4-hydroxypheny)-1H-indazole-3-carboxamide (8j).

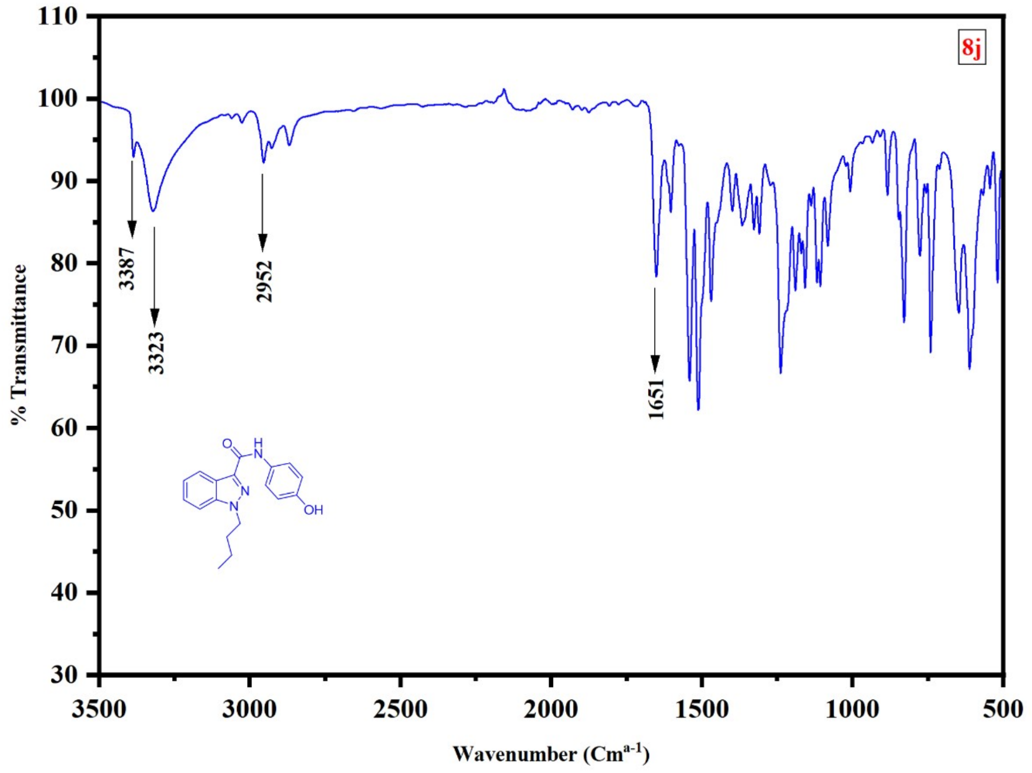

HRMS of 1-butyl-N-(4-hydroxypheny)-1H-indazole-3-carboxamide (8j).

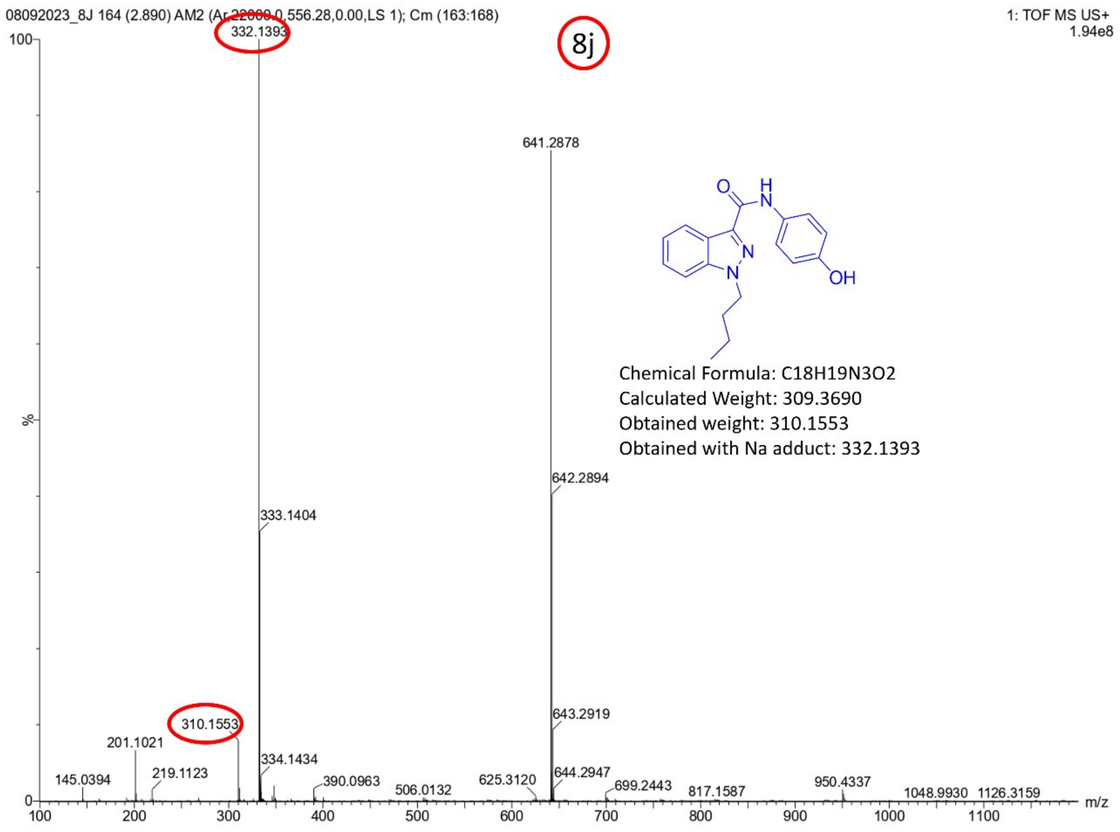

^{1}H-NMR [400MHz, DMSO-d_6] spectrum of 1-butyl-N-(2-hydroxy-6-methylpheny)-1H-indazole-3-carboxamide (8k).

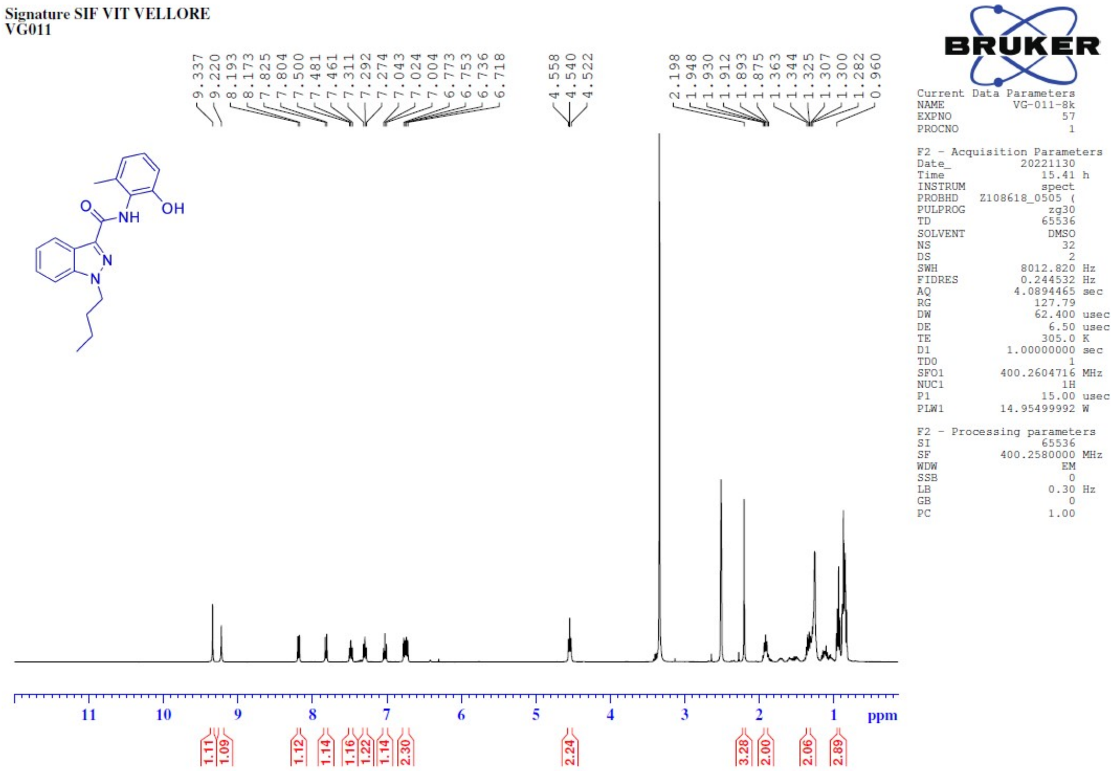

^{13}C-NMR [100MHz, DMSO-d_6] spectrum of 1-butyl-N-(2-hydroxy-6-methylpheny)-1H-indazole-3-carboxamide (8k).

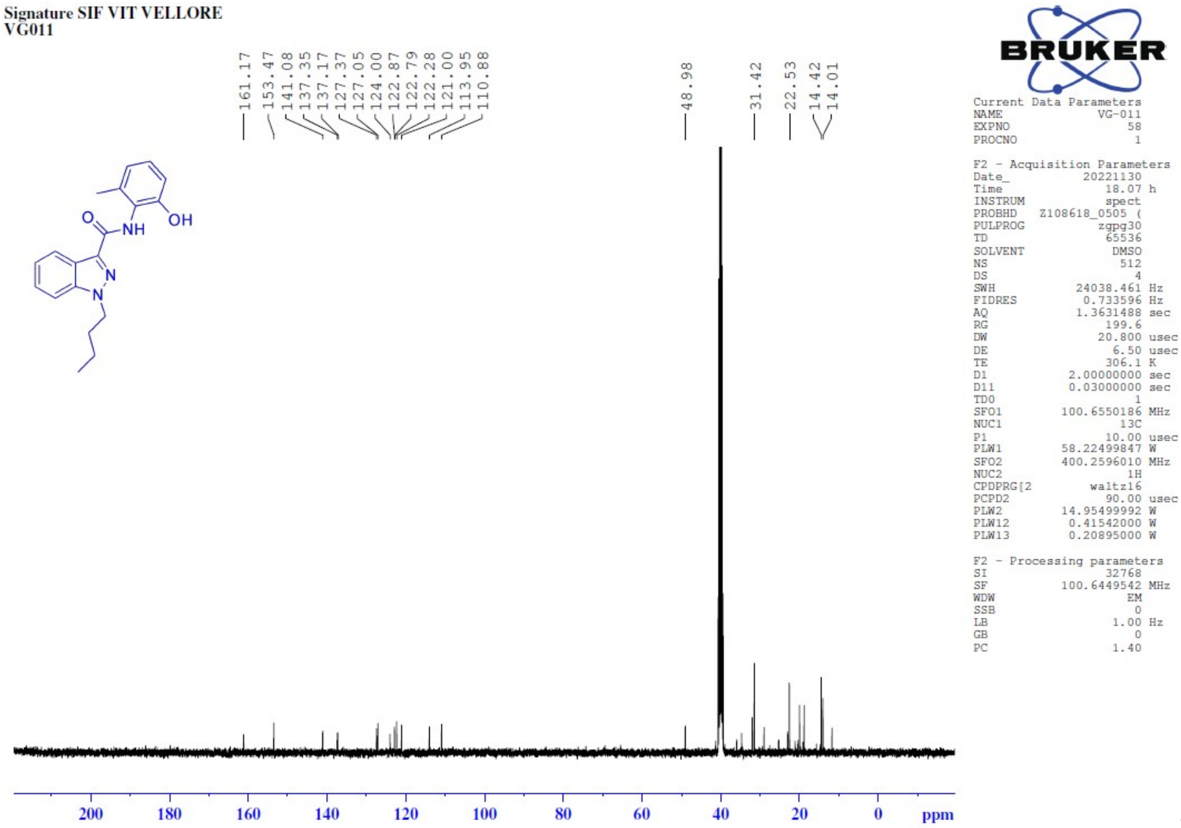

13

5-DEPT-NMR [100MHz, DMSO-d_6] spectrum of 1-butyl-N-(2-hydroxy-6-methylpheny)-1H-indazole-3-carboxamide (8k).

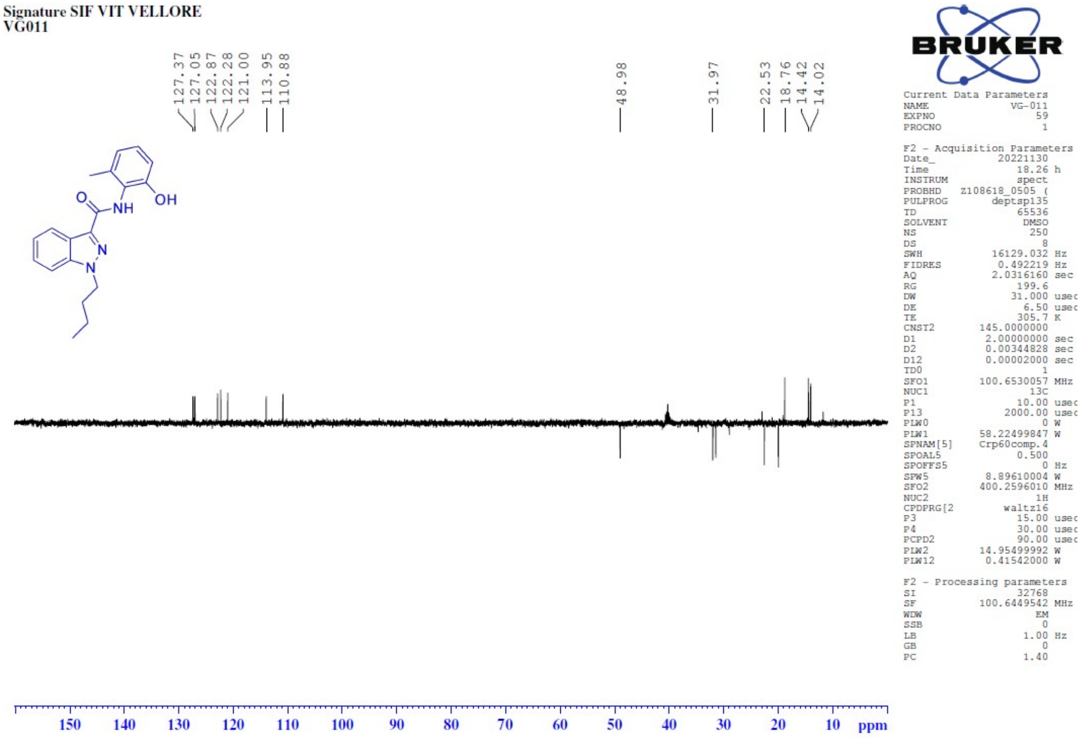

FT-IR spectrum of 1-butyl-N-(2-hydroxy-6-methylpheny)-1H-indazole-3-carboxamide (8k).

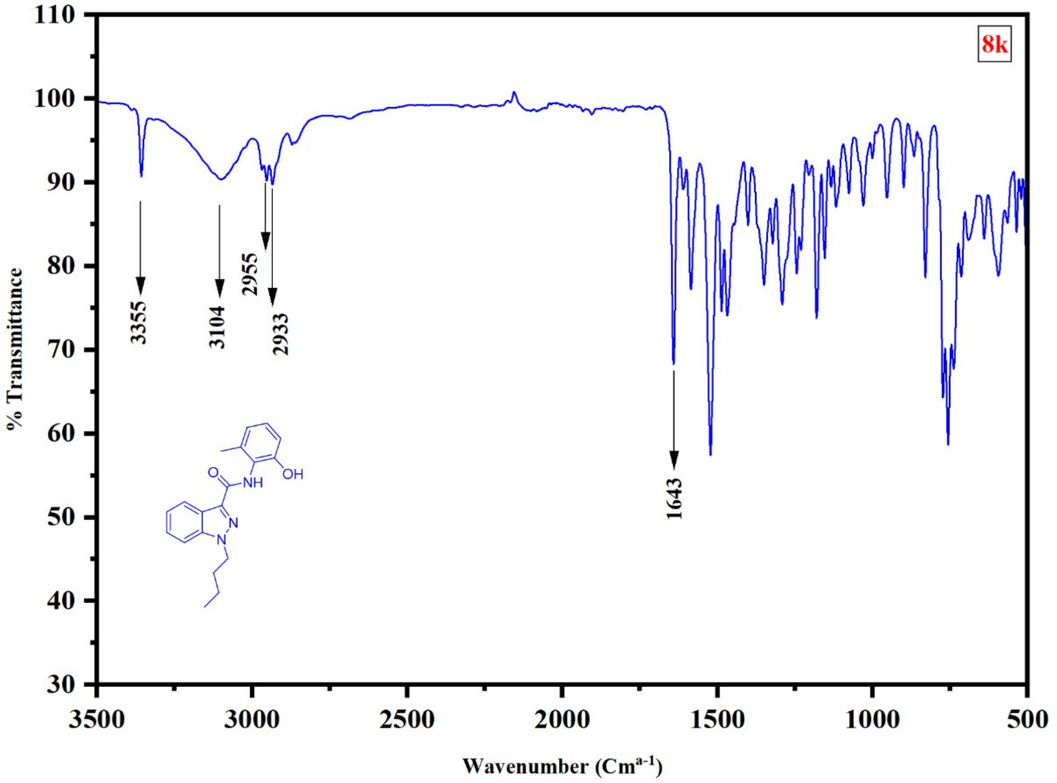

HRMS of 1-butyl-N-(2-hydroxy-6-methylpheny)-1H-indazole-3-carboxamide (8k).

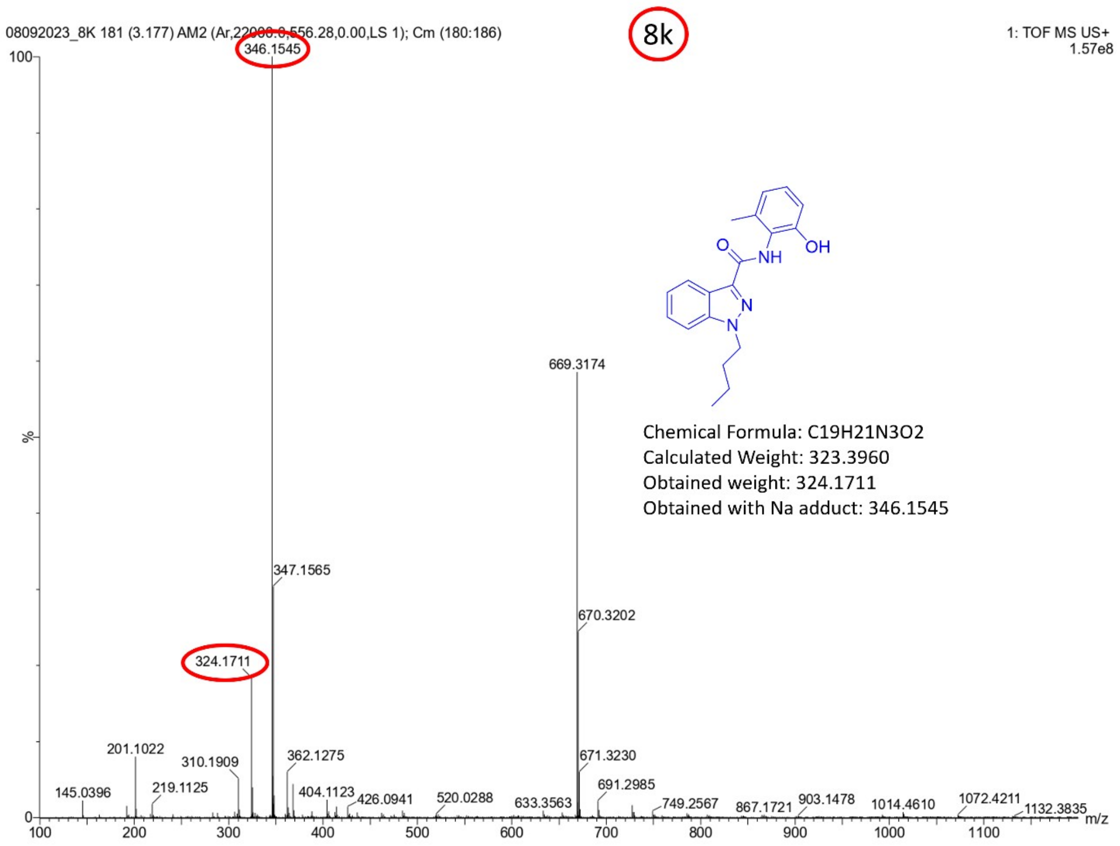
08092023_8K 181 (3.177) AM2 (Ar,22000.0,556.28,0.00,LS 1); Cm (180:186)
8k
1: TOF MS US+
1.57e8
100
%
0
346.1545
669.3174
347.1565
670.3202
324.1711
201.1022
310.1909
362.1275
671.3230
145.0396
219.1125
404.1123
426.0941
520.0288
633.3563
691.2985
749.2567
867.1721
903.1478
1014.4610
1072.4211
1132.3835
m/z
100
200
300
400
500
600
700
800
900
1000
1100
O
NH
OH
N
N
Chemical Formula: C19H21N3O2
Calculated Weight: 323.3960
Obtained weight: 324.1711
Obtained with Na adduct: 346.1545

^{1}H-NMR [400MHz, DMSO-d$_6$] spectrum of 1-butyl-N-(m-tolyl)-1H-indazole-3-carboxamide (8l).

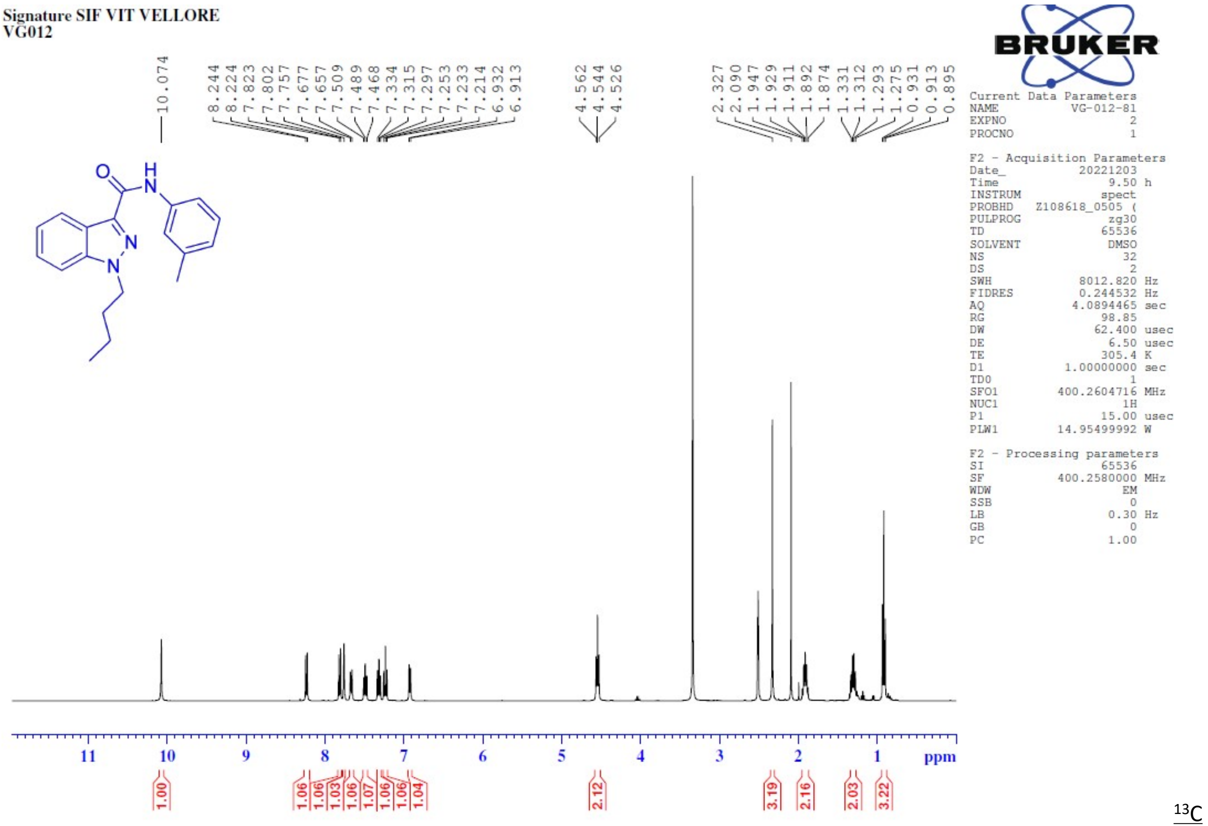

^{13}C-NMR [100MHz, DMSO-d$_6$] spectrum of 1-butyl-N-(m-tolyl)-1H-indazole-3-carboxamide (8l).

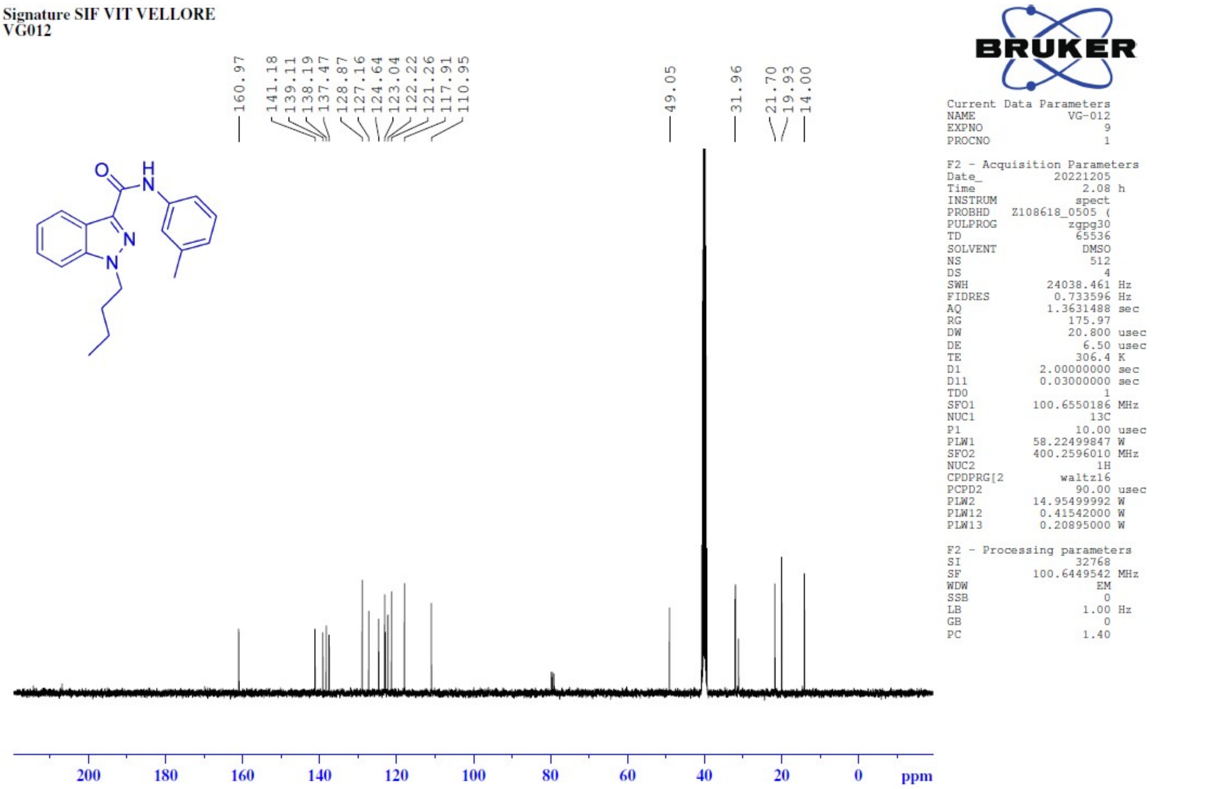

135-DEPT-NMR [100MHz, DMSO-d$_6$] spectrum of 1-butyl-N-(m-tolyl)-1H-indazole-3-carboxamide (8l).

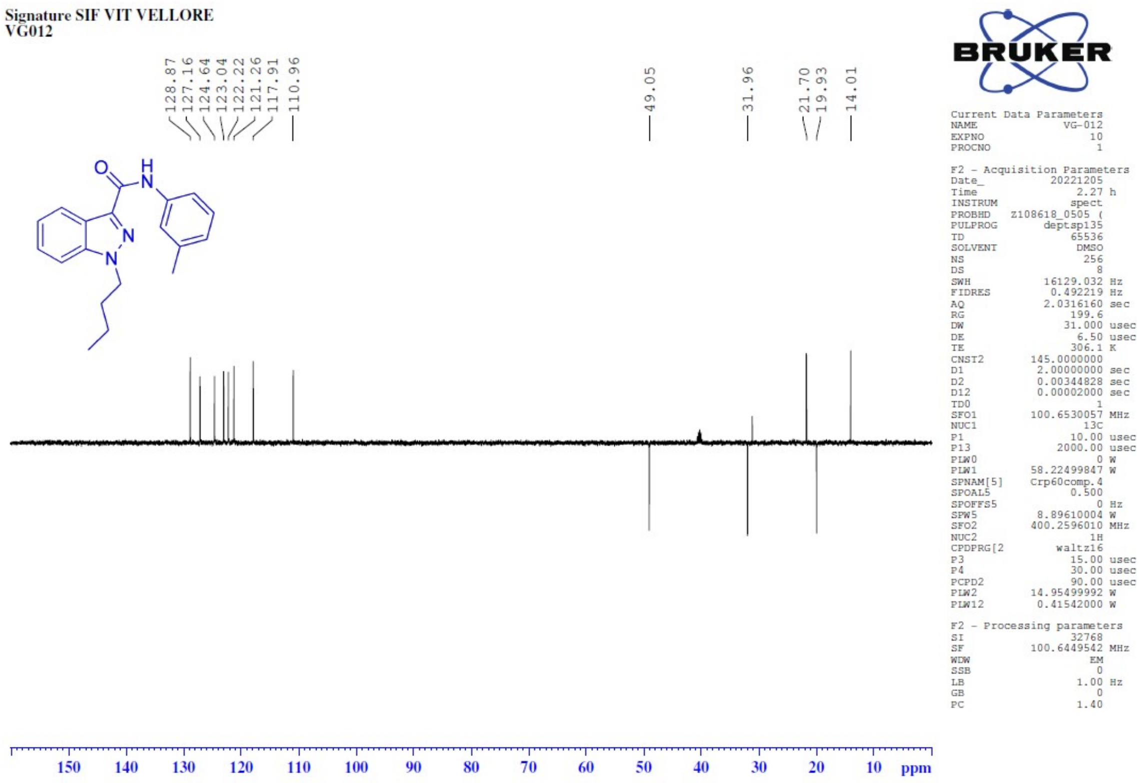

COSY-NMR [400MHz, DMSO-d$_6$] spectrum of 1-butyl-N-(m-tolyl)-1H-indazole-3-carboxamide (8l).

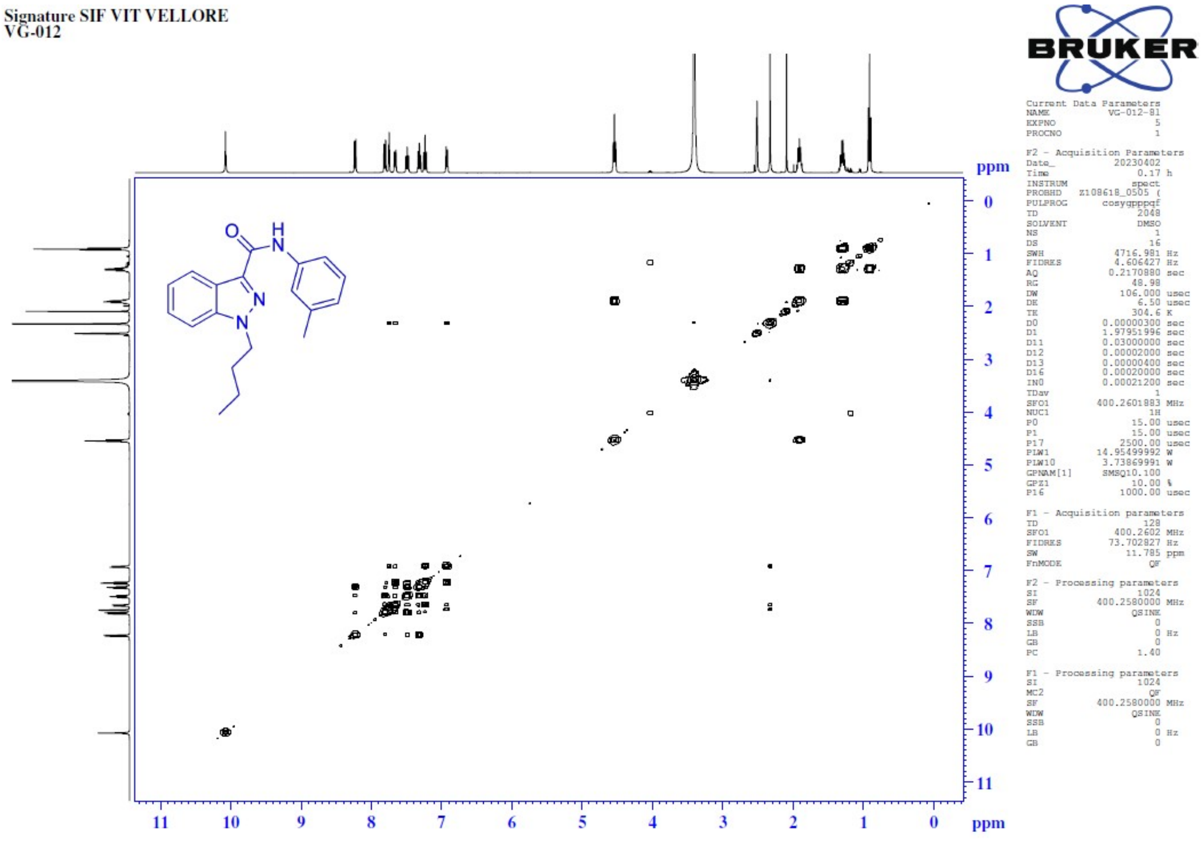

HSQC-NMR [400MHz, DMSO-d$_6$] spectrum of 1-butyl-N-(m-tolyl)-1H-indazole-3-carboxamide (8l).

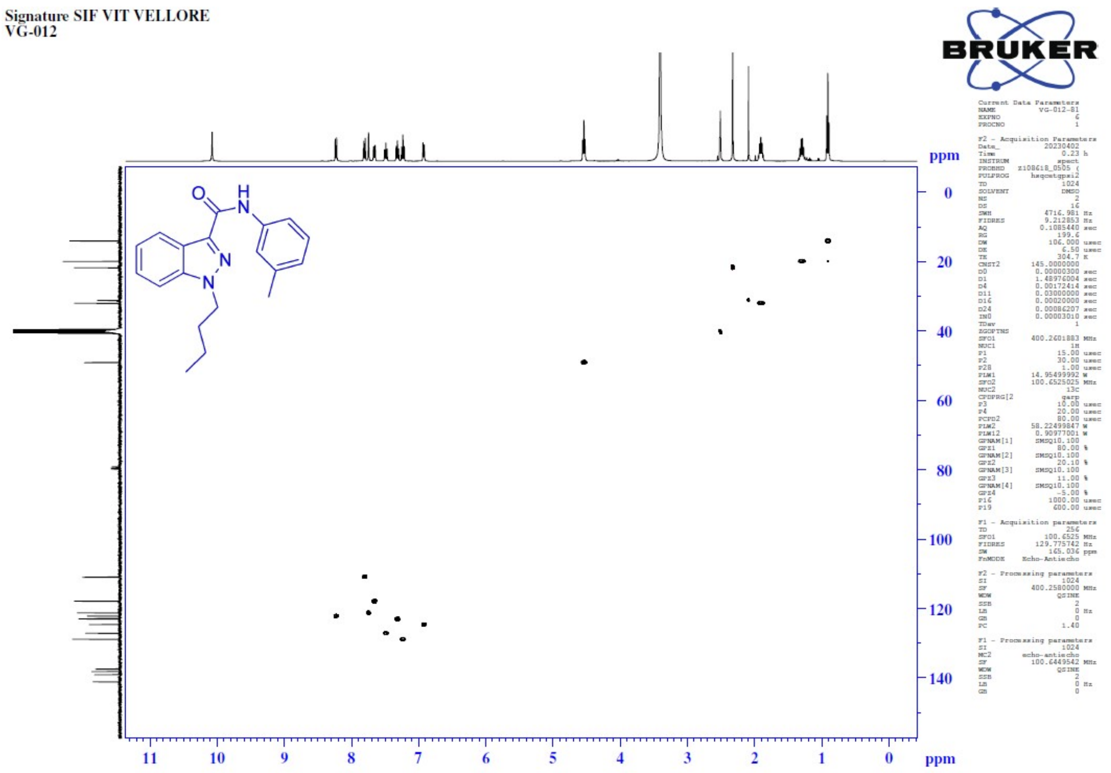

FT-IR spectrum of 1-butyl-N-(m-tolyl)-1H-indazole-3-carboxamide (8l).

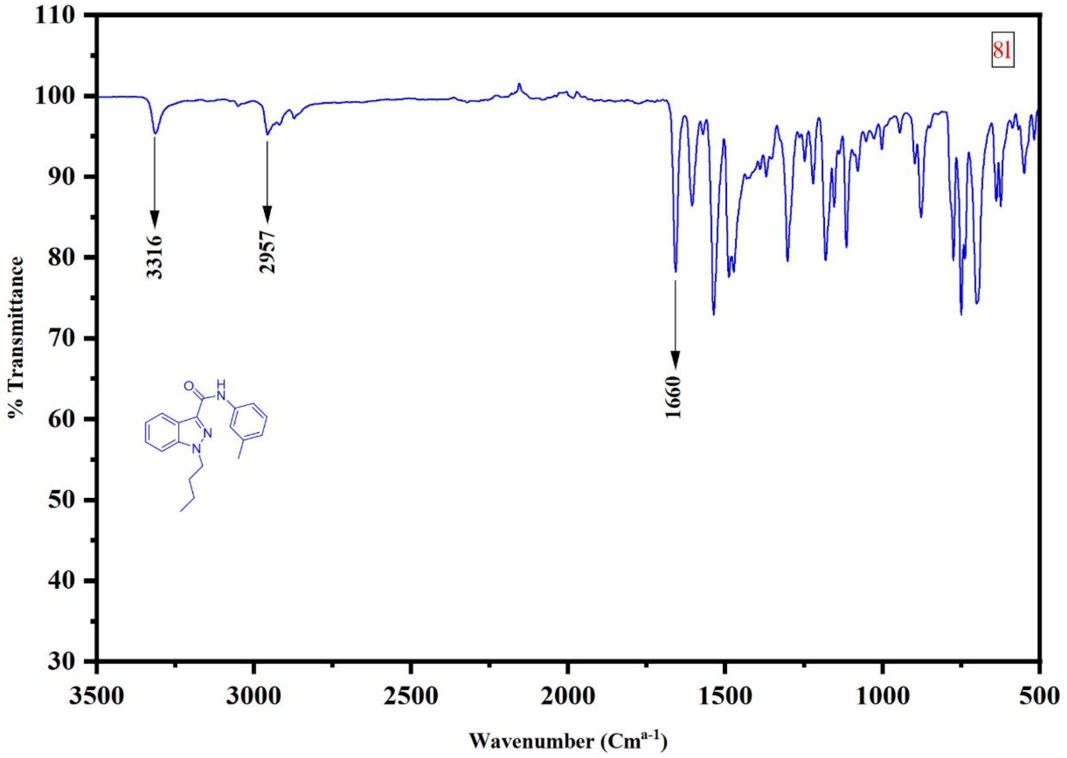

HRMS of 1-butyl-N-(m-tolyl)-1H-indazole-3-carboxamide (8l).

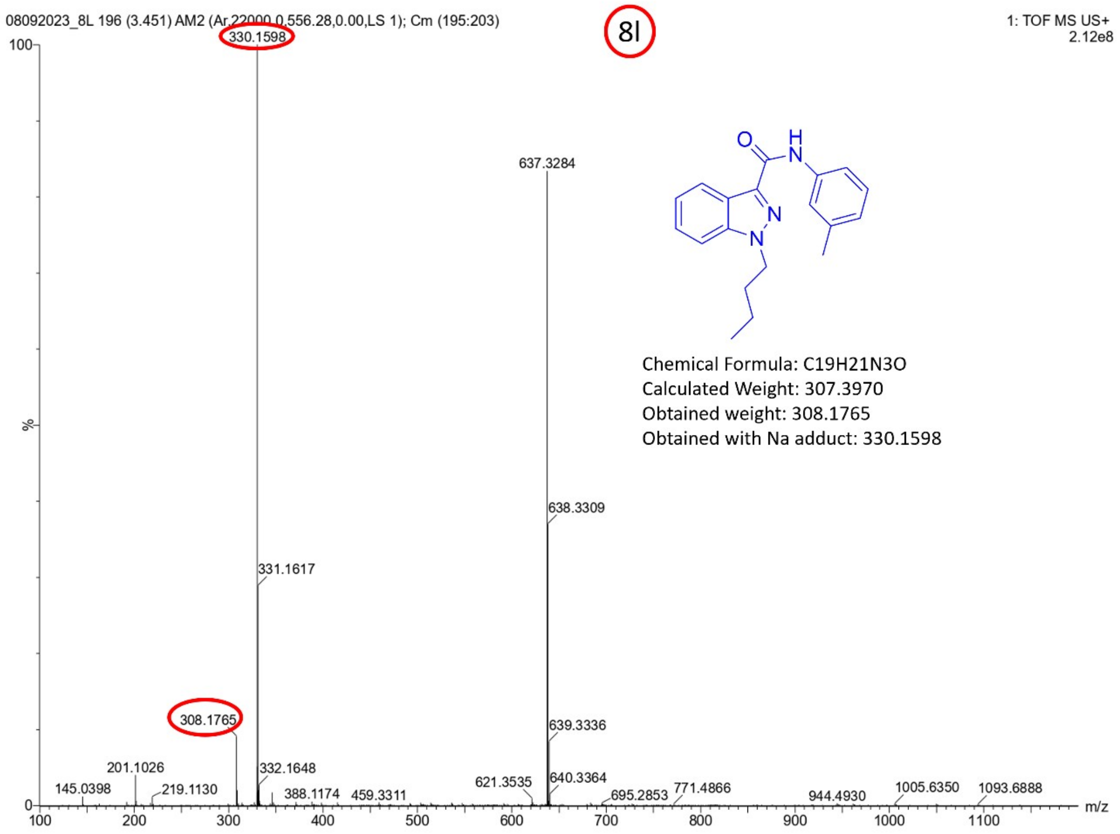

^{1}H-NMR [400MHz, DMSO-d_6] spectrum of 1-butyl-N-(-tolyl)-1H-indazole-3-carboxamide (8m).

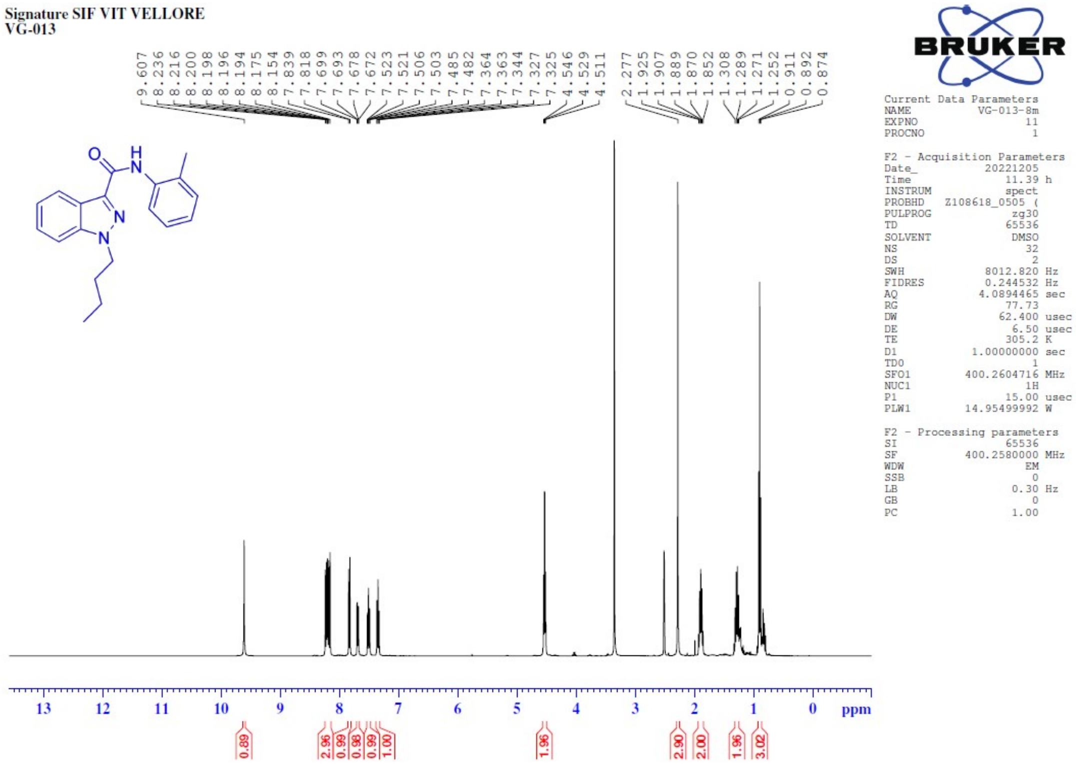

^{13}C-NMR [100MHz, DMSO-d_6] spectrum of 1-butyl-N-(o-tolyl)-1H-indazole-3-carboxamide (8m).

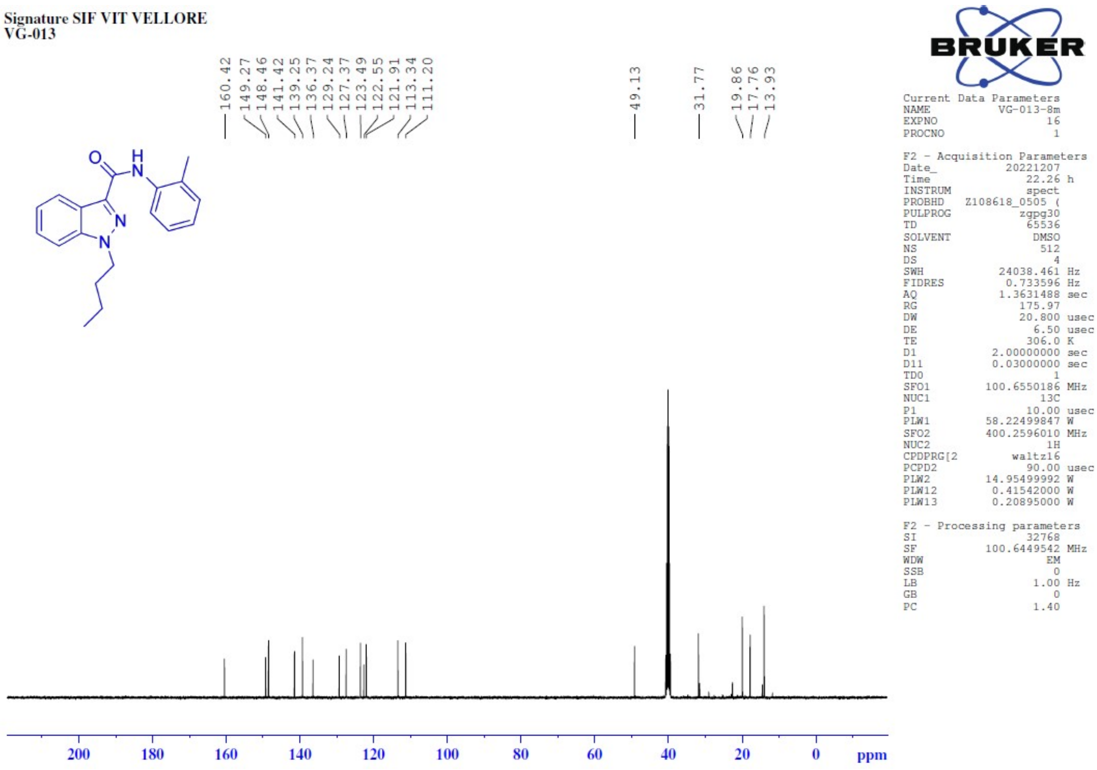

135-DEPT-NMR [100MHz, DMSO-d_6] spectrum of 1-butyl-N-(o-tolyl)-1H-indazole-3-carboxamide (8m).

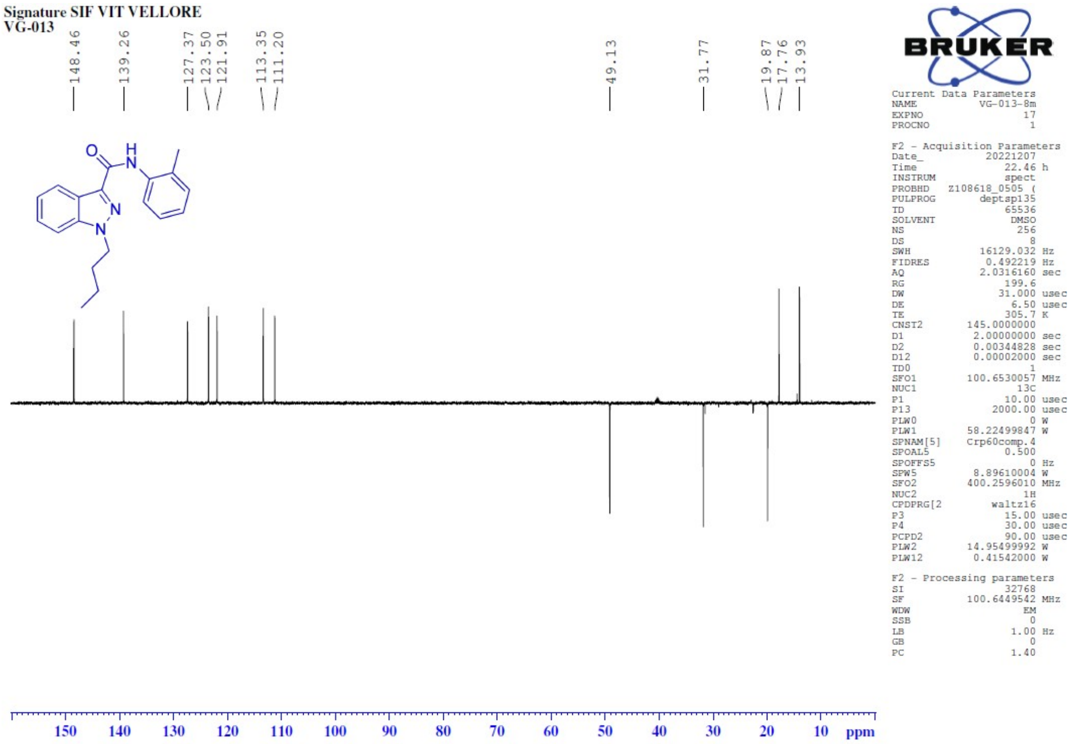

COSY-NMR [400MHz, DMSO-d_6] spectrum of 1-butyl-N-(o-tolyl)-1H-indazole-3-carboxamide (8m).

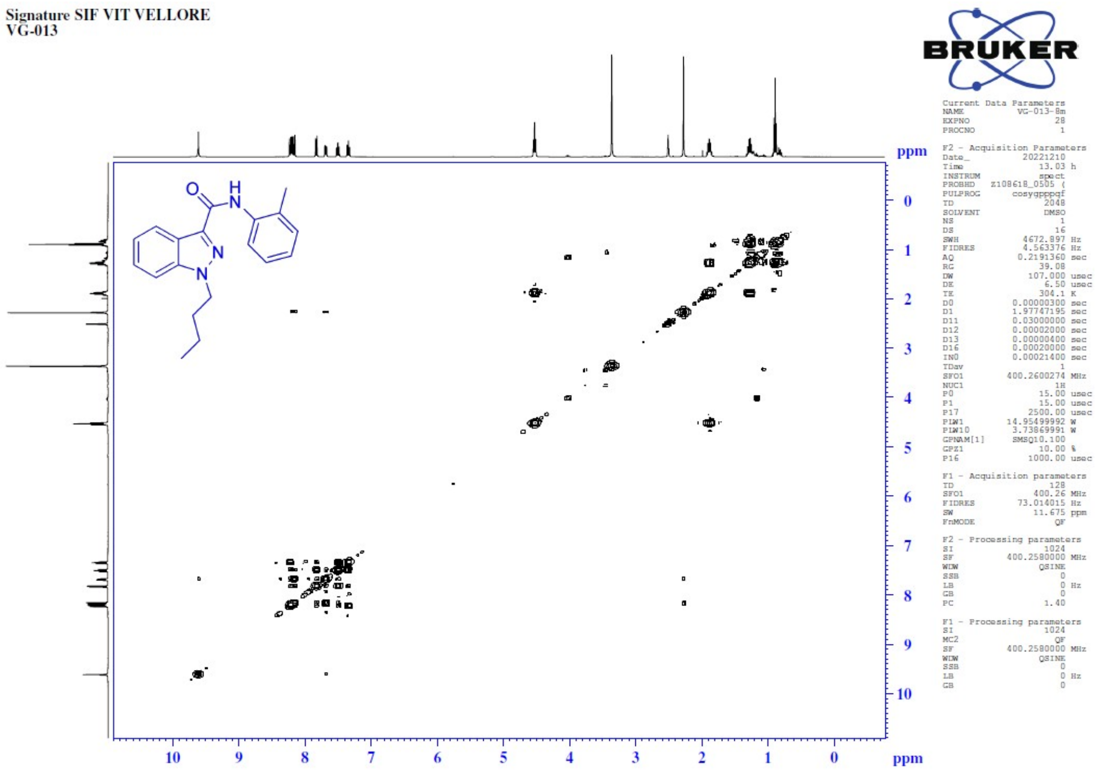

HSQC-NMR [400MHz, DMSO-d_6] spectrum of 1-butyl-N-(o-tolyl)-1H-indazole-3-carboxamide (8m).

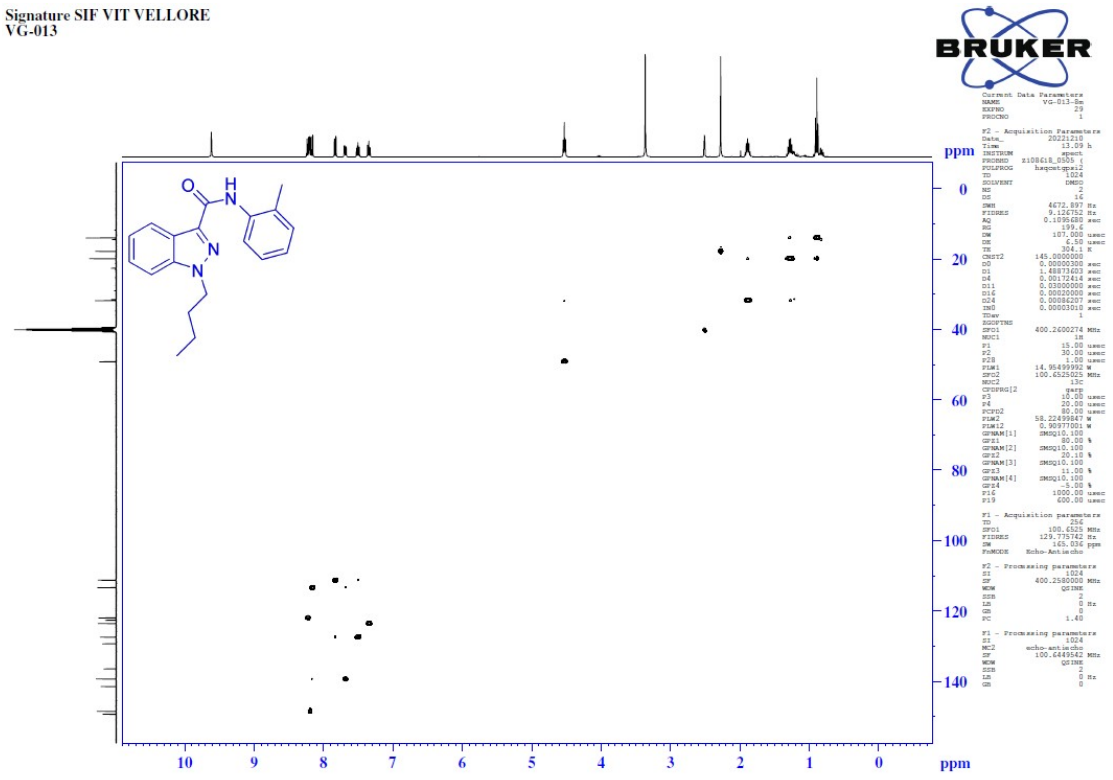

FT-IR spectrum of 1-butyl-N-(o-tolyl)-1H-indazole-3-carboxamide (8m).

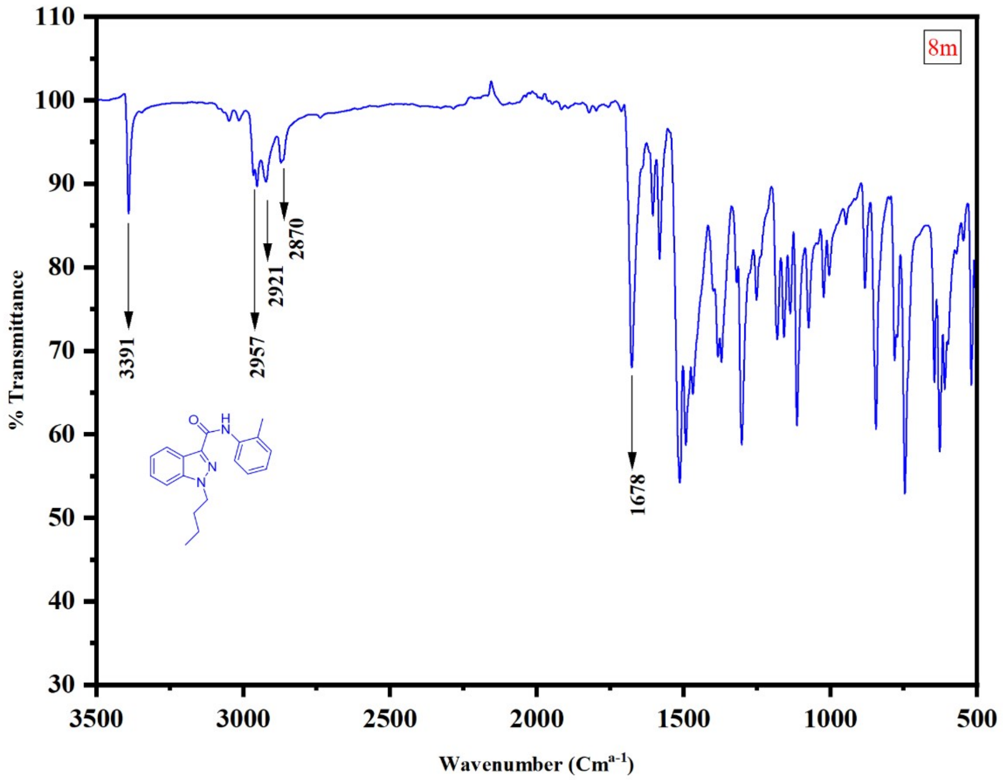

HRMS of 1-butyl-N-(o-tolyl)-1H-indazole-3-carboxamide (8m).

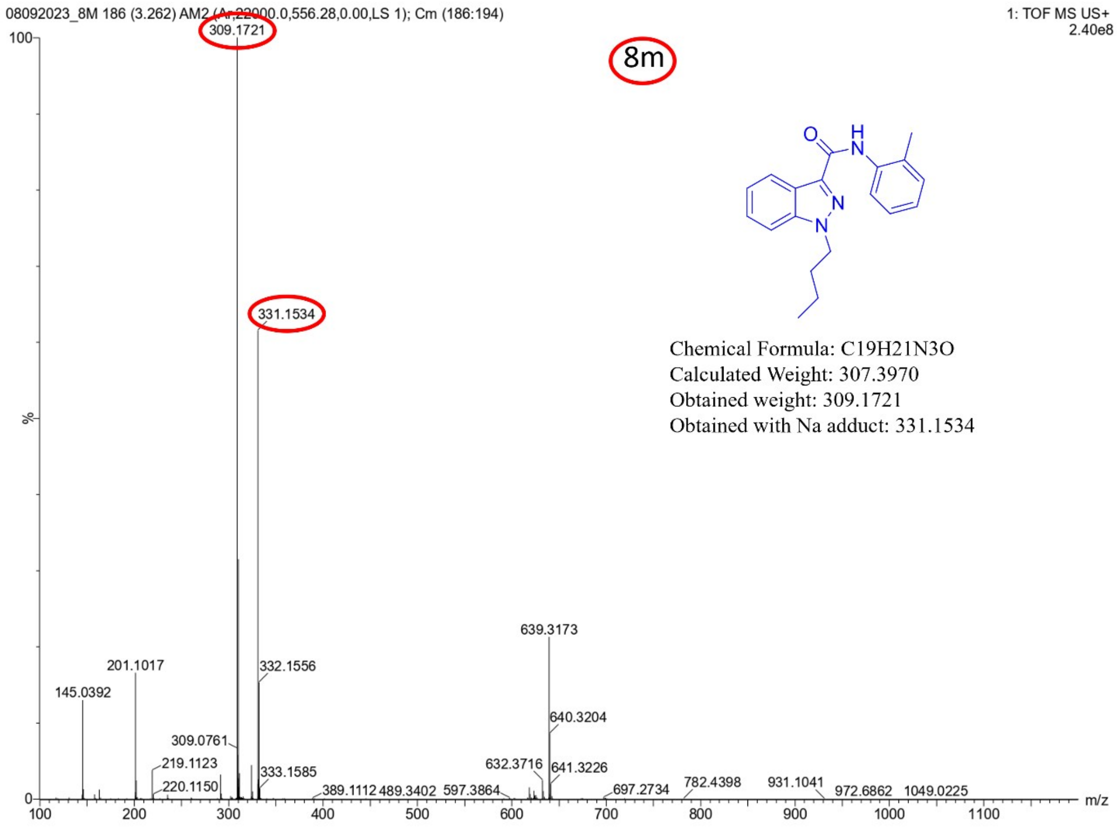

^{1}H-NMR [400MHz, DMSO-d$_6$] spectrum of 1-butyl-N-(5-methylpyridin-2-yl)-1H-indazole-3-carboxamide (8n).

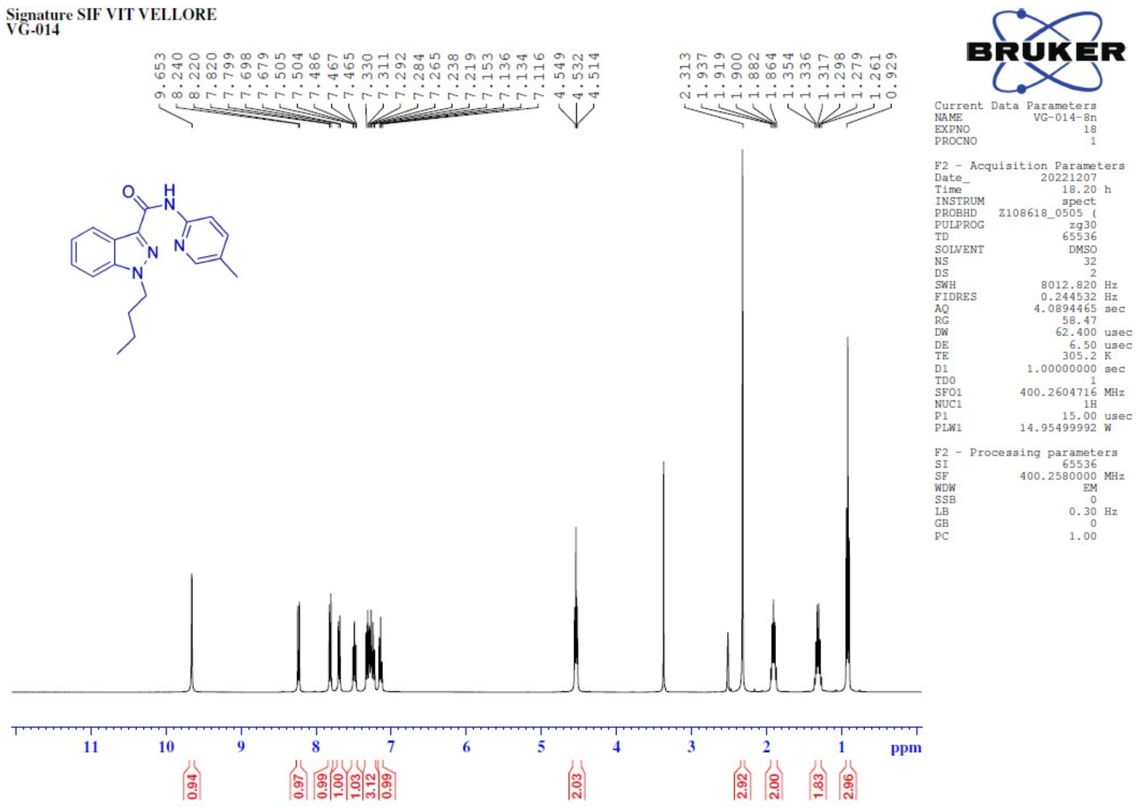

^{13}C-NMR [100MHz, DMSO-d$_6$] spectrum of 1-butyl-N-(5-methylpyridin-2-yl)-1H-indazole-3-carboxamide (8n).

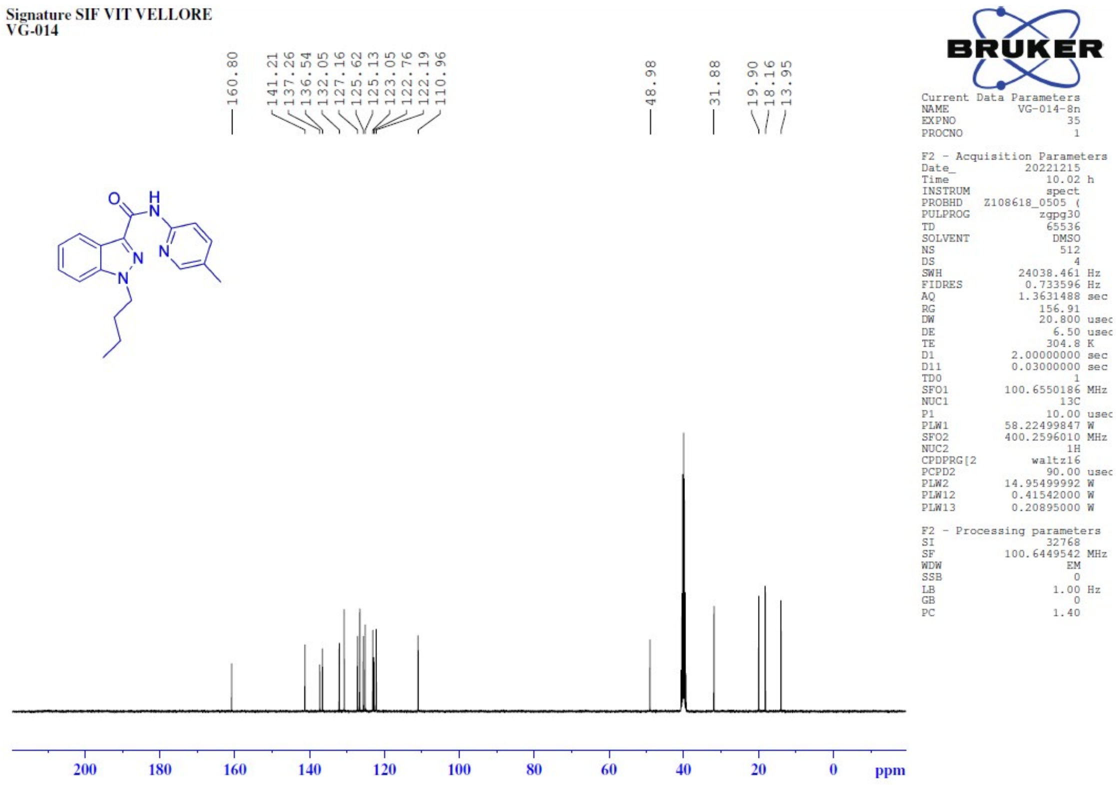

135-DEPT-NMR [100MHz, DMSO-d_6] spectrum of 1-butyl-N-(5-methylpyridin-2-yl)-1H-indazole-3-carboxamide (8n).

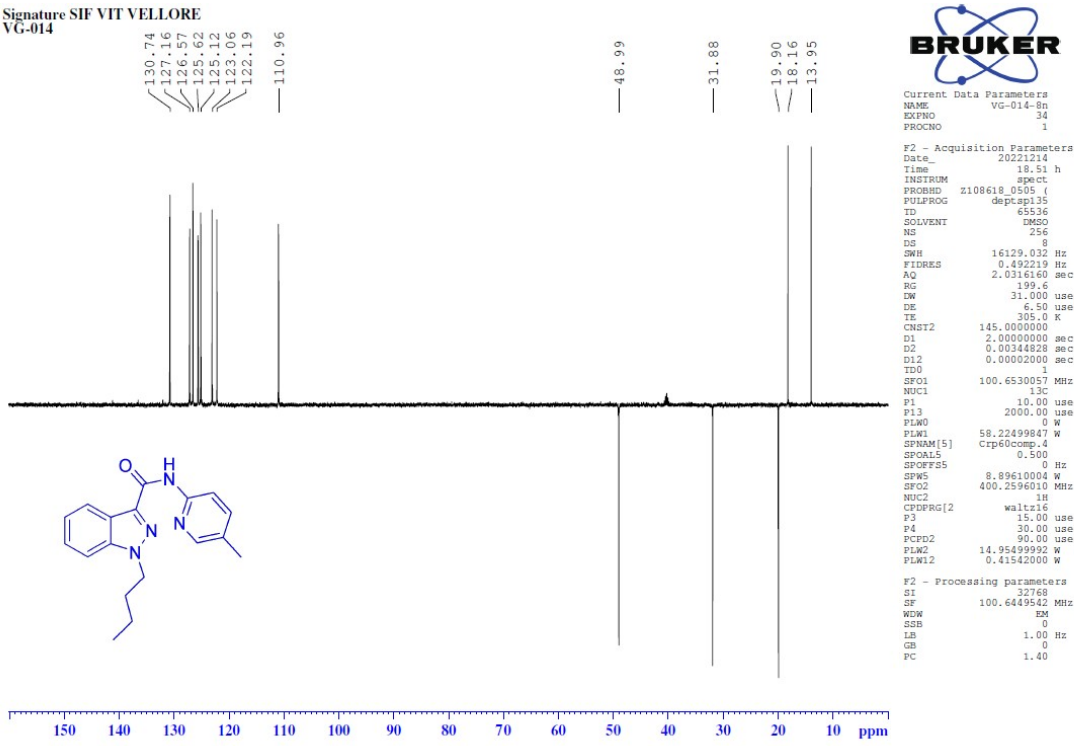

COSY-NMR [400MHz, DMSO-d_6] spectrum of 1-butyl-N-(5-methylpyridin-2-yl)-1H-indazole-3-carboxamide (8n).

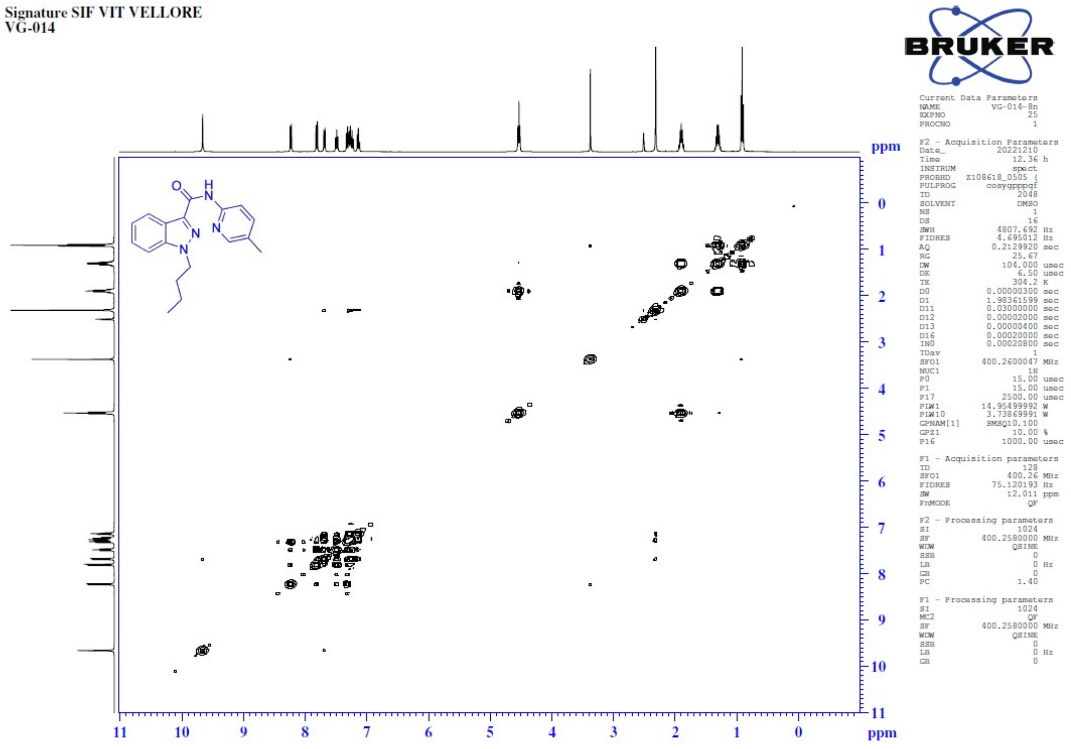

HSQC-NMR [400MHz, DMSO-d_6] spectrum of 1-butyl-N-(5-methylpyridin-2-yl)-1H-indazole-3-carboxamide (8n).

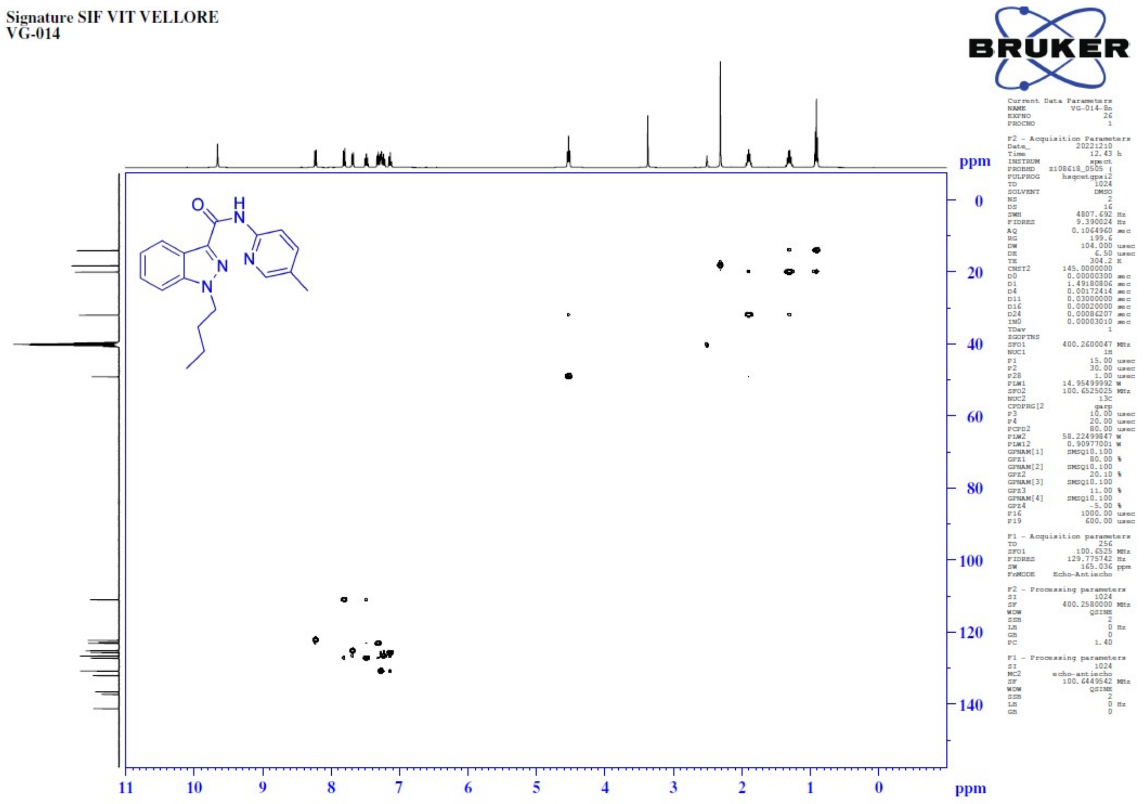

<u>FT-IR spectrum of 1-butyl-N-(5-methylpyridin-2-yl)-1H-indazole-3-carboxamide (8n).</u>

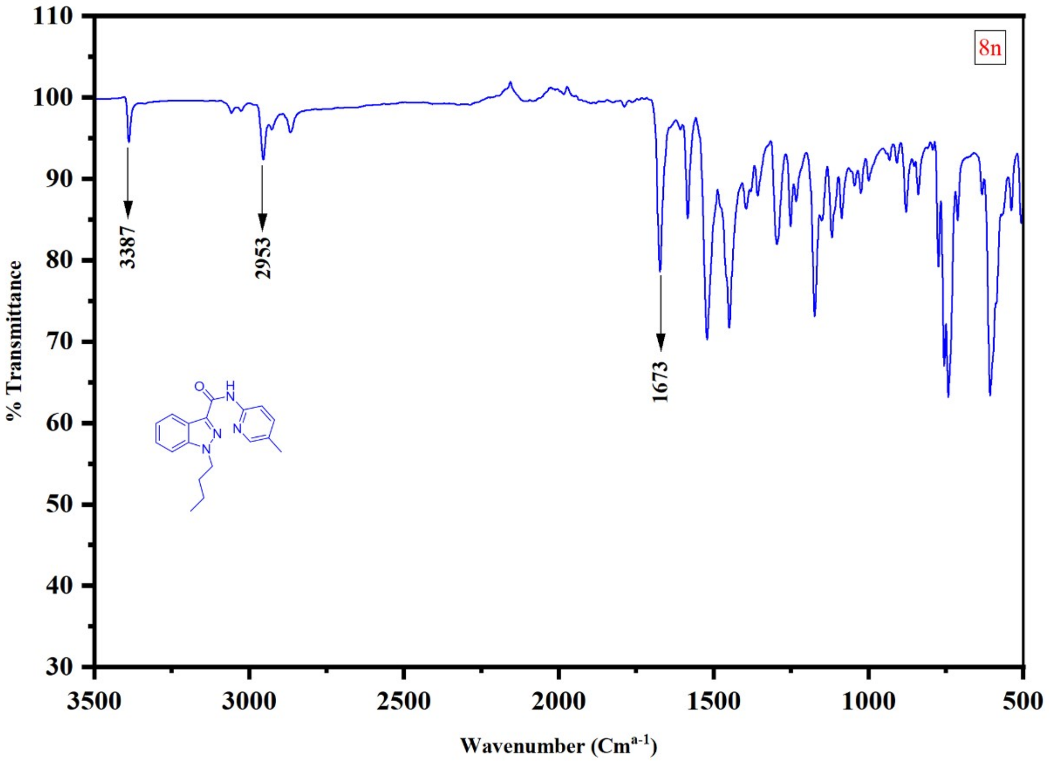

<u>HRMS of 1-butyl-N-(5-methylpyridin-2-yl)-1H-indazole-3-carboxamide (8n).</u>

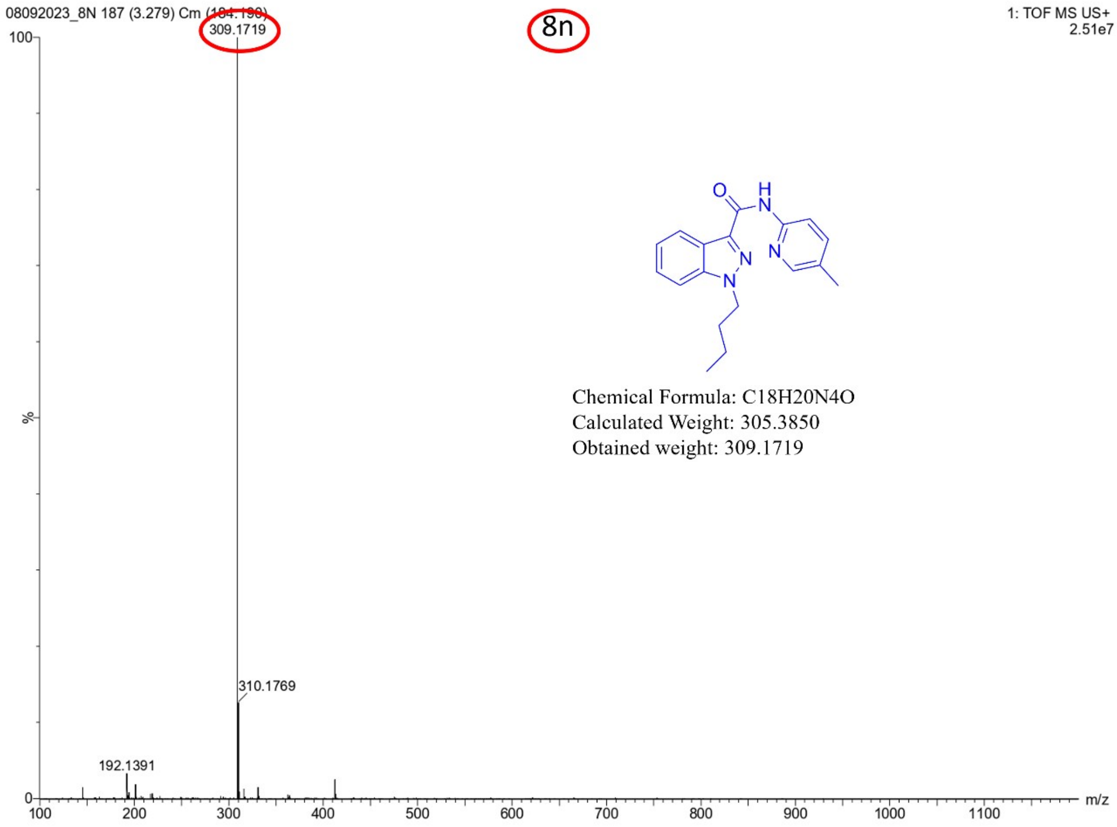

^{1}H-NMR [400MHz, DMSO-d_6] spectrum of 1-butyl-N-(4-methoxyphenyl)-1H-indazole-3-carboxamide (8o).

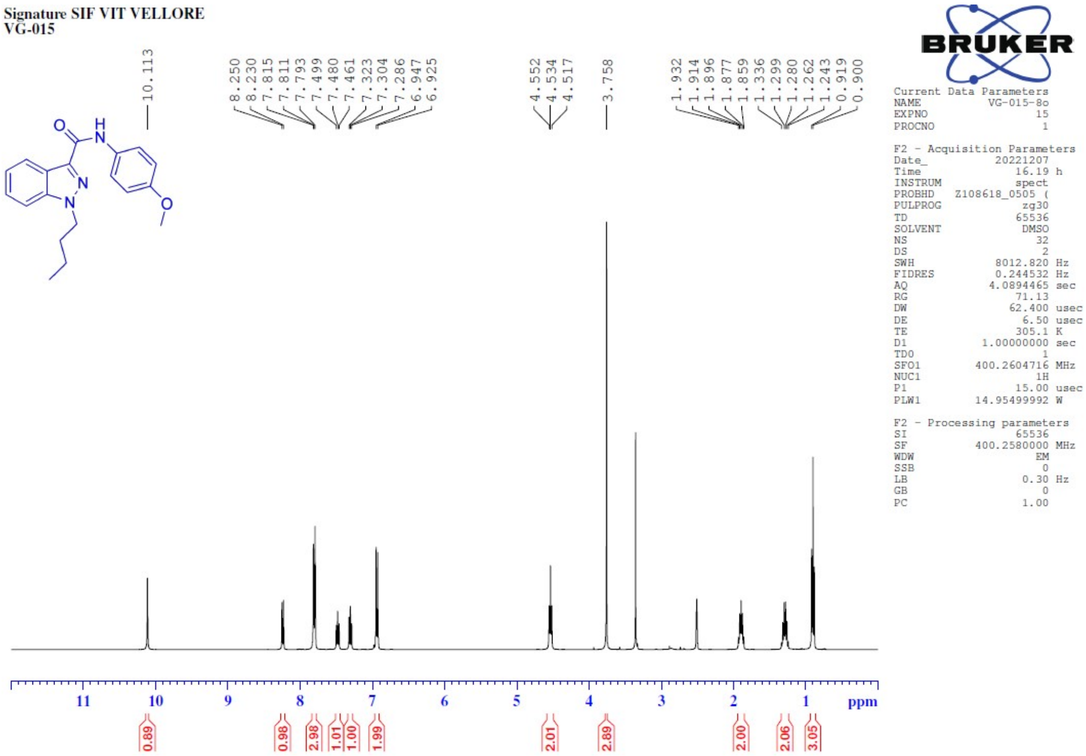

^{13}C-NMR [400MHz, DMSO-d_6] spectrum of 1-butyl-N-(4-methoxyphenyl)-1H-indazole-3-carboxamide (8o).

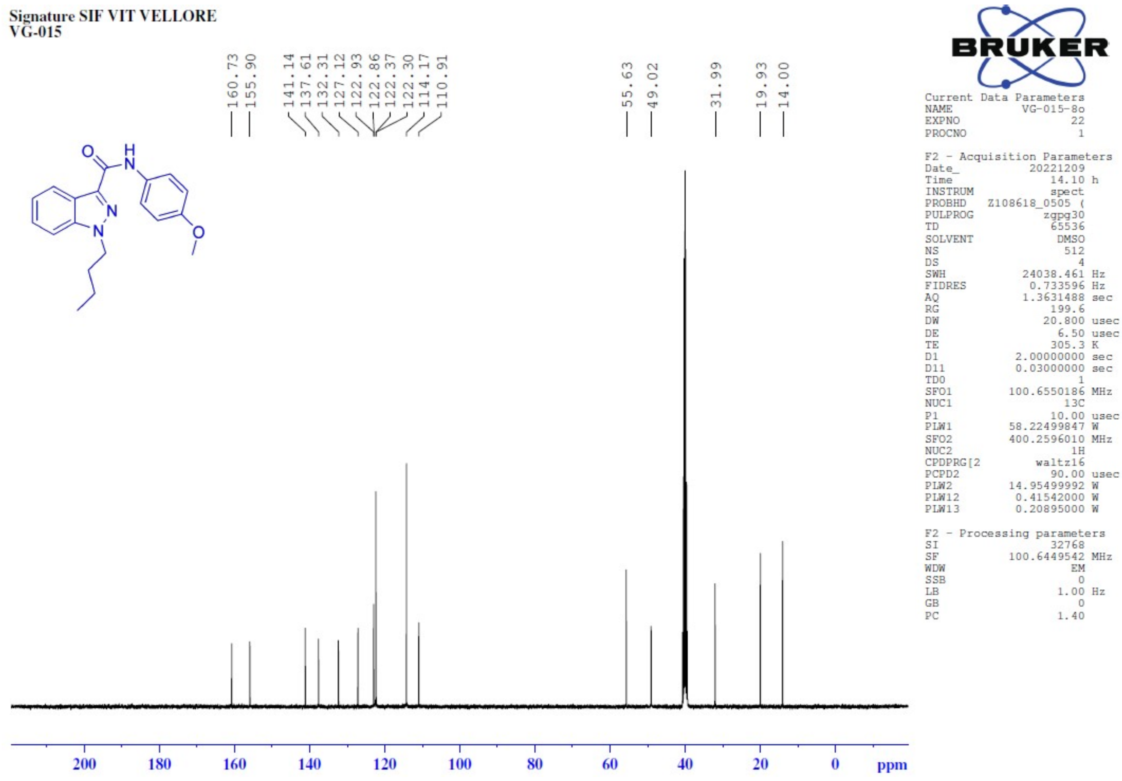

135-DEPT-NMR [400MHz, DMSO-d_6] spectrum of 1-butyl-N-(4-methoxyphenyl)-1H-indazole-3-carboxamide (8o).

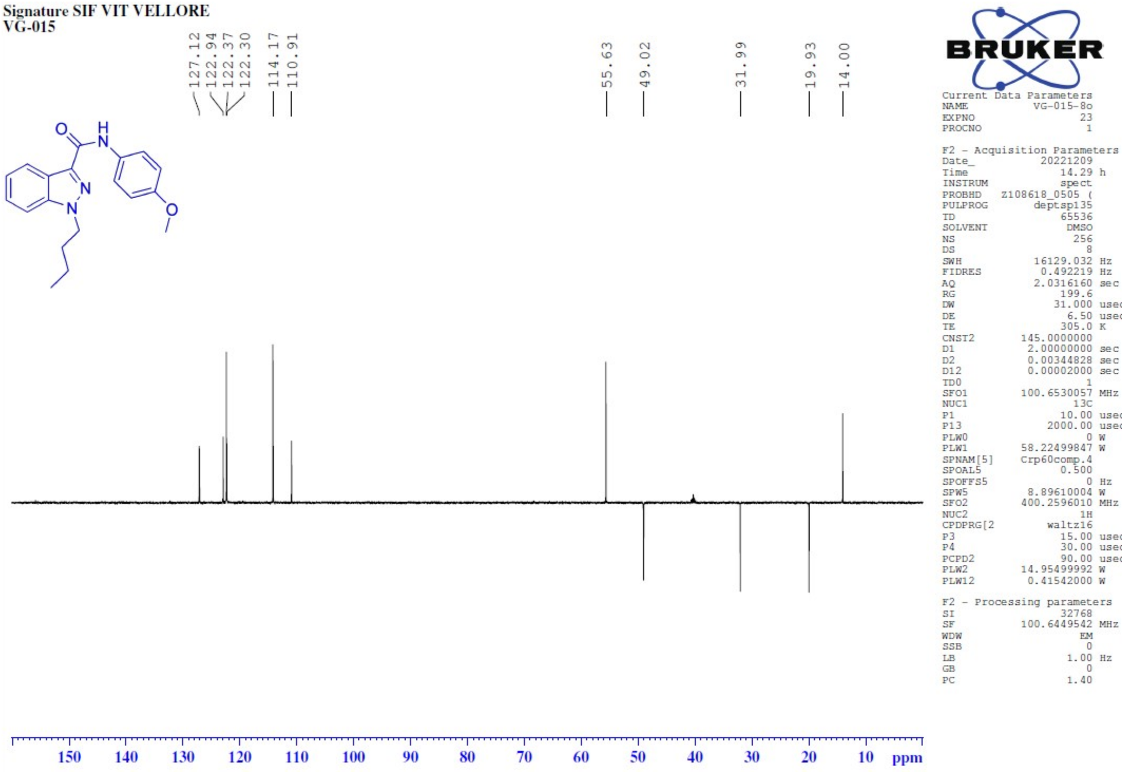

COSY-NMR [400MHz, DMSO-d_6] spectrum of 1-butyl-N-(4-methoxyphenyl)-1H-indazole-3-carboxamide (8o).

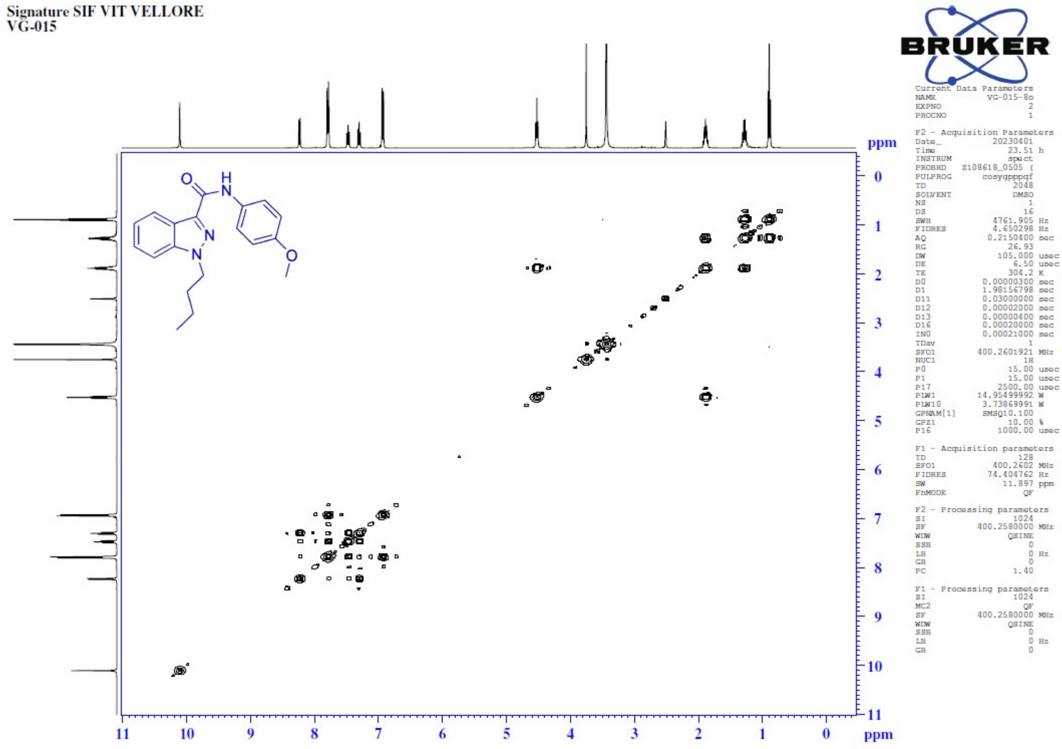

HSQC-NMR [400MHz, DMSO-d_6] spectrum of 1-butyl-N-(4-methoxyphenyl)-1H-indazole-3-carboxamide (8o).

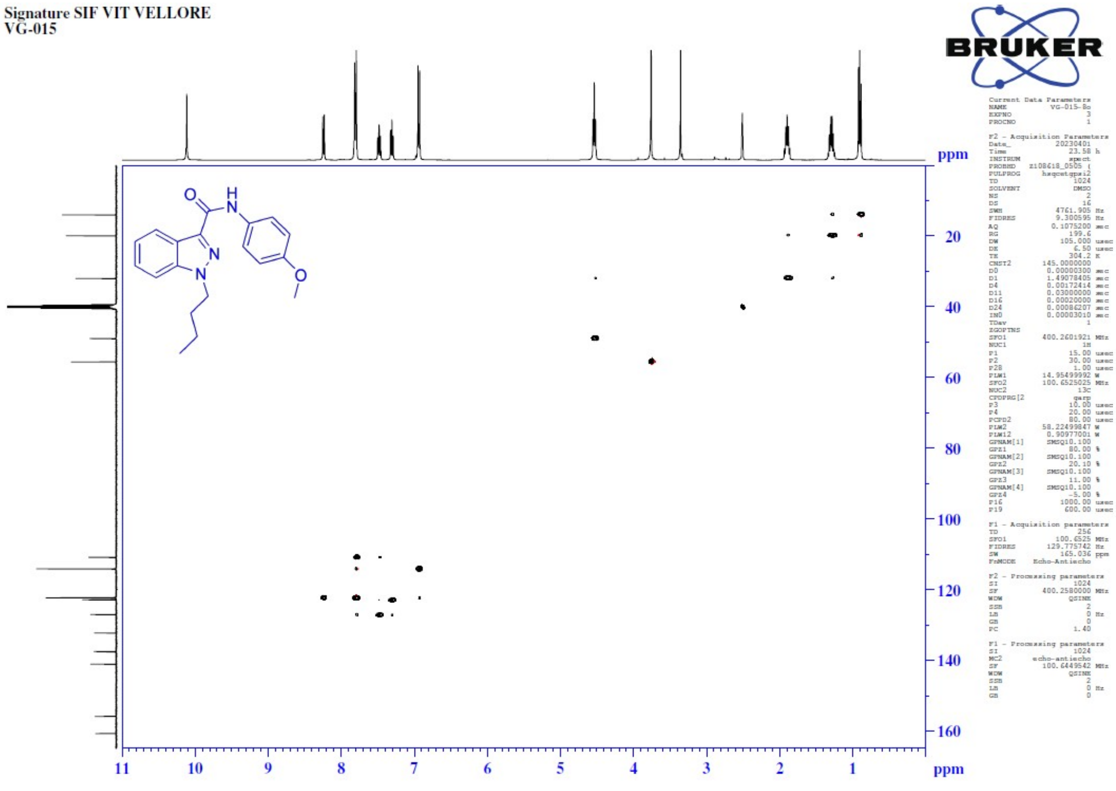

FT-IR spectrum of 1-butyl-N-(4-methoxyphenyl)-1H-indazole-3-carboxamide (8o).

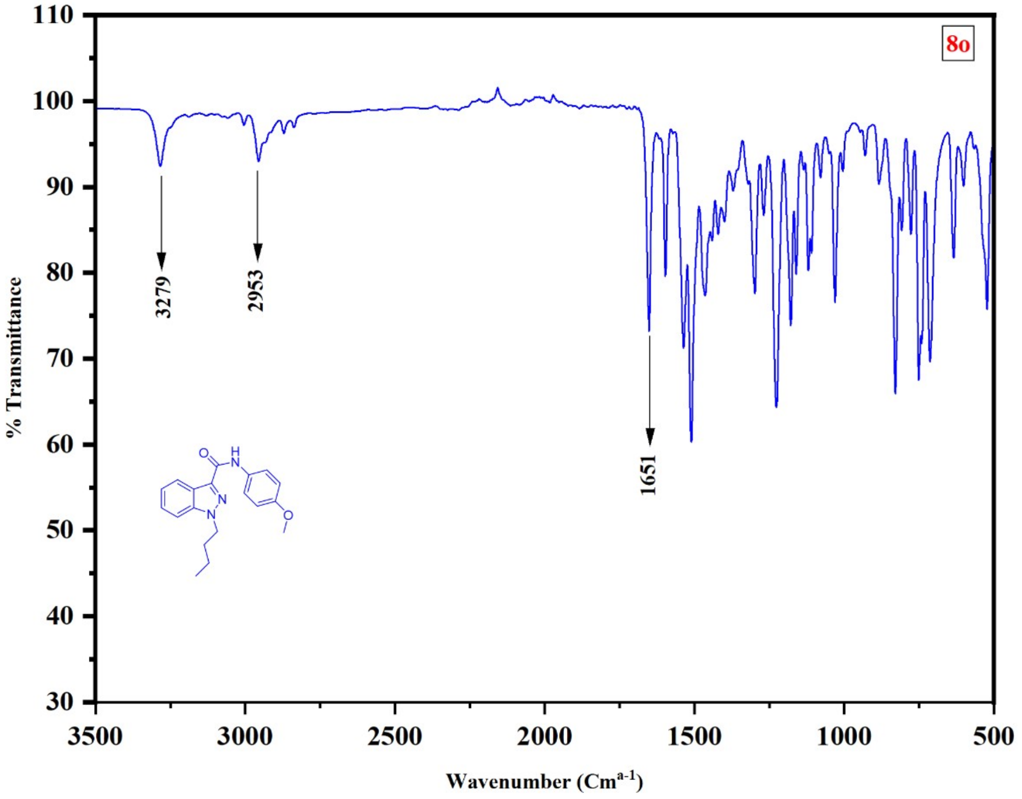

HRMS of 1-butyl-N-(4-methoxyphenyl)-1H-indazole-3-carboxamide (8o).

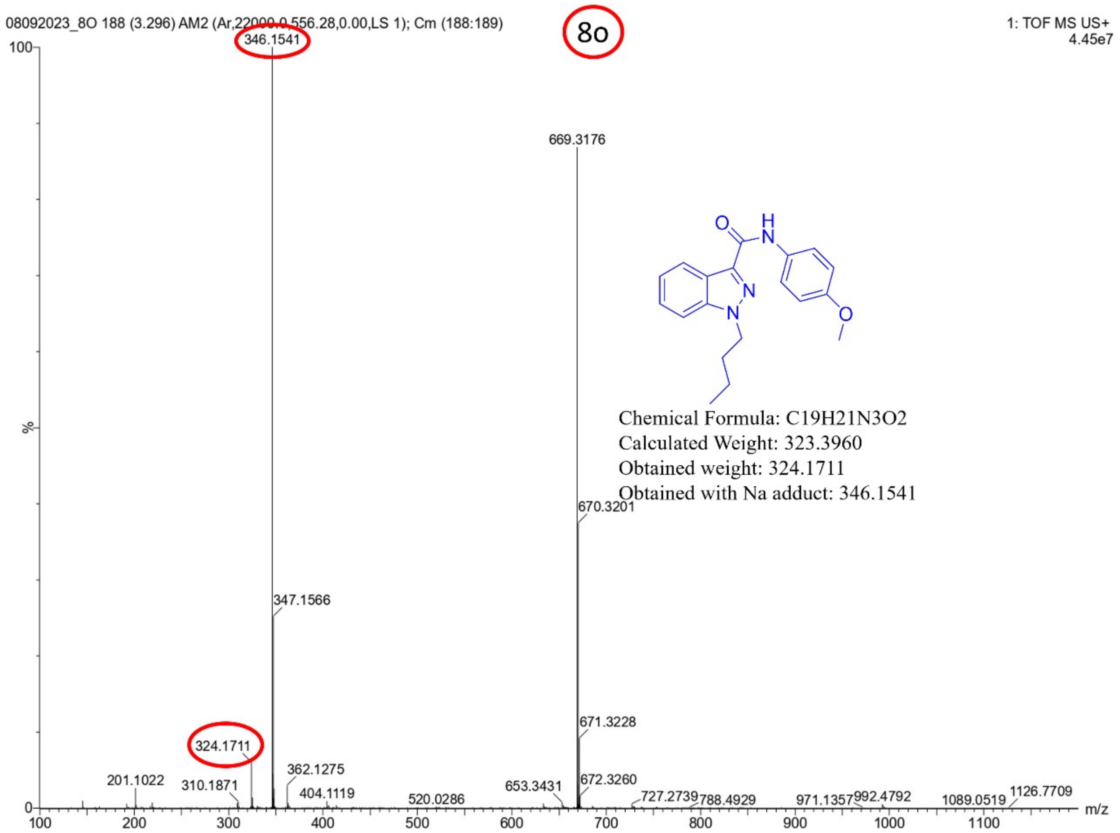

^{1}H-NMR [400MHz, DMSO-d_6] spectrum of 1-butyl-N-(4-fluorophenyl)-1H-indazole-3-carboxamide (8p).

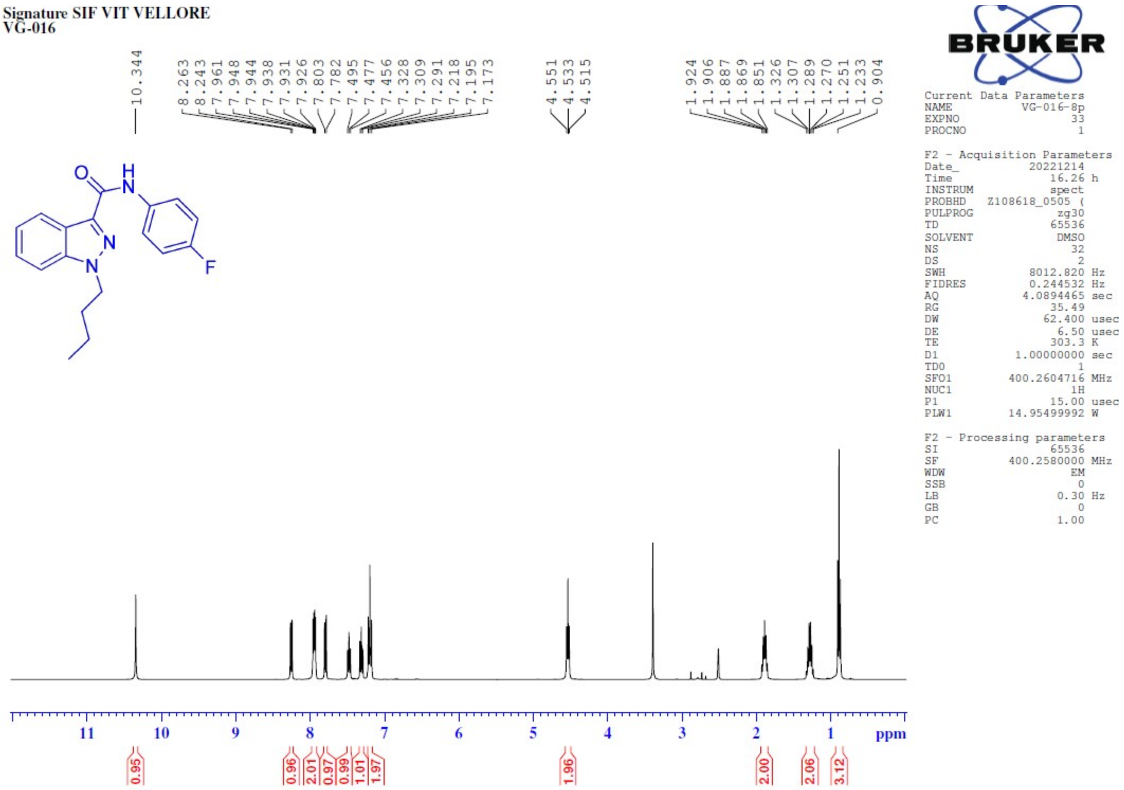

^{13}C-NMR [100MHz, DMSO-d_6] spectrum of 1-butyl-N-(4-fluorophenyl)-1H-indazole-3-carboxamide (8p).

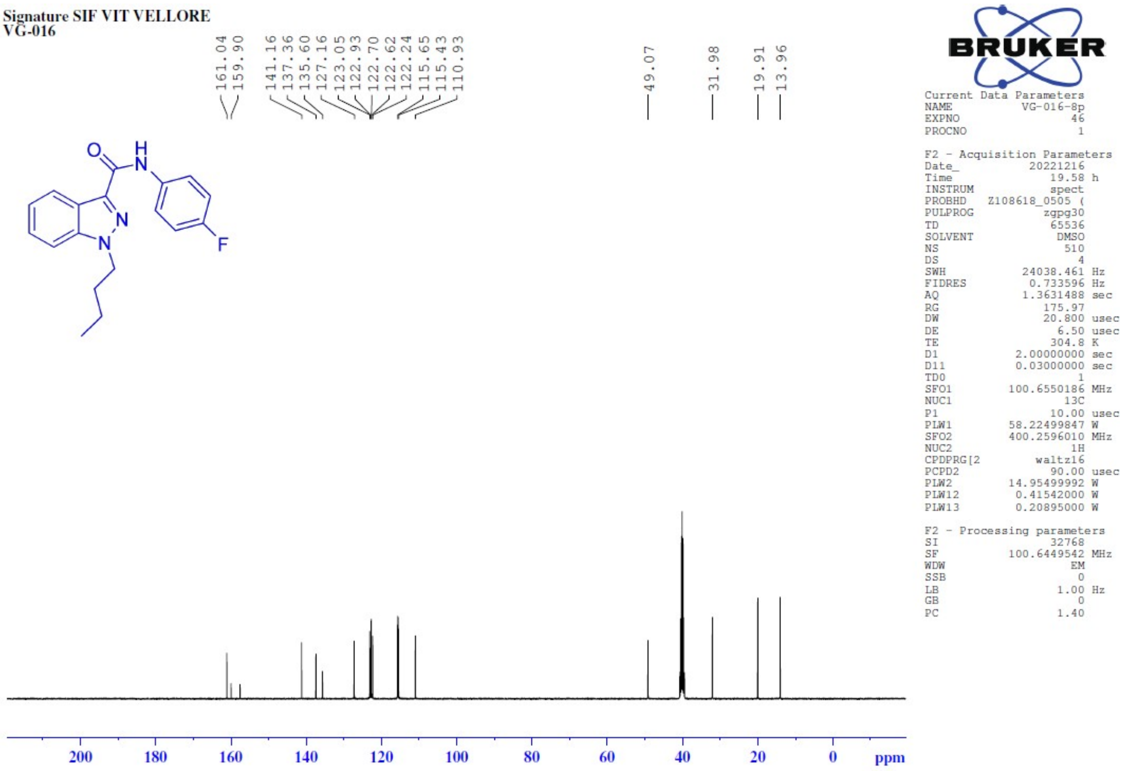

135-DEPT-NMR [100MHz, DMSO-d_6] spectrum of 1-butyl-N-(4-fluorophenyl)-1H-indazole-3-carboxamide (8p).

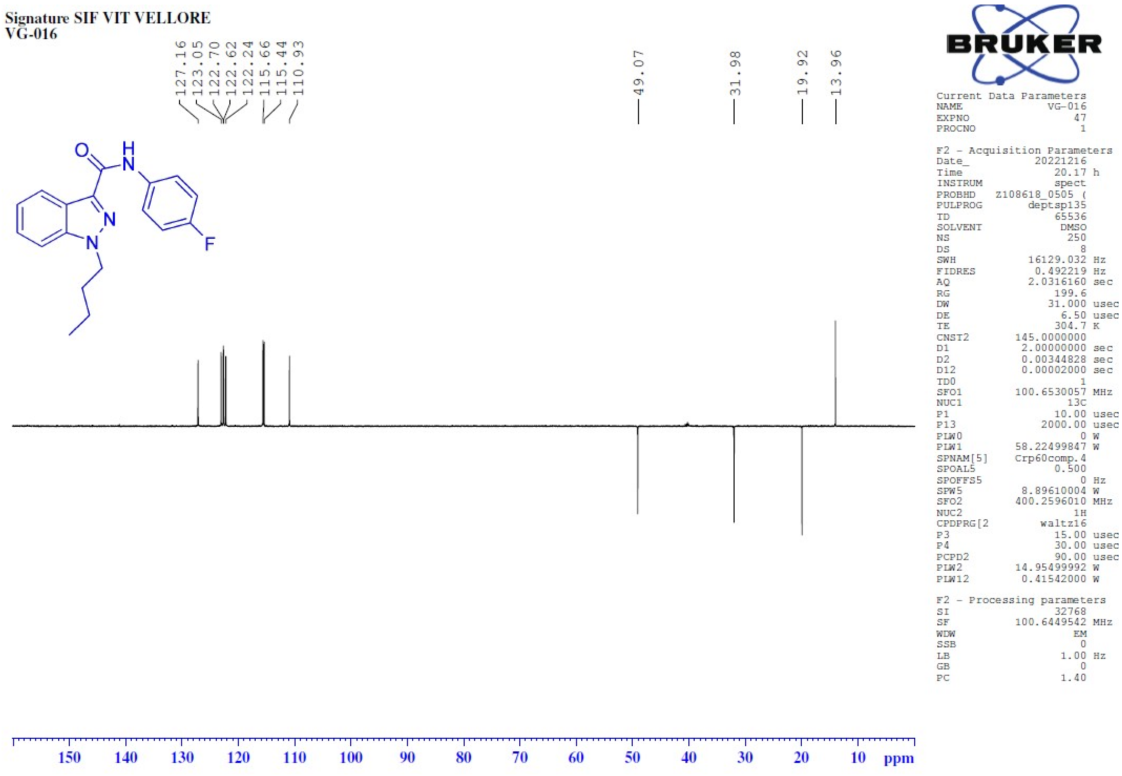

FT-IR spectrum of 1-butyl-N-(4-fluorophenyl)-1H-indazole-3-carboxamide (8p).

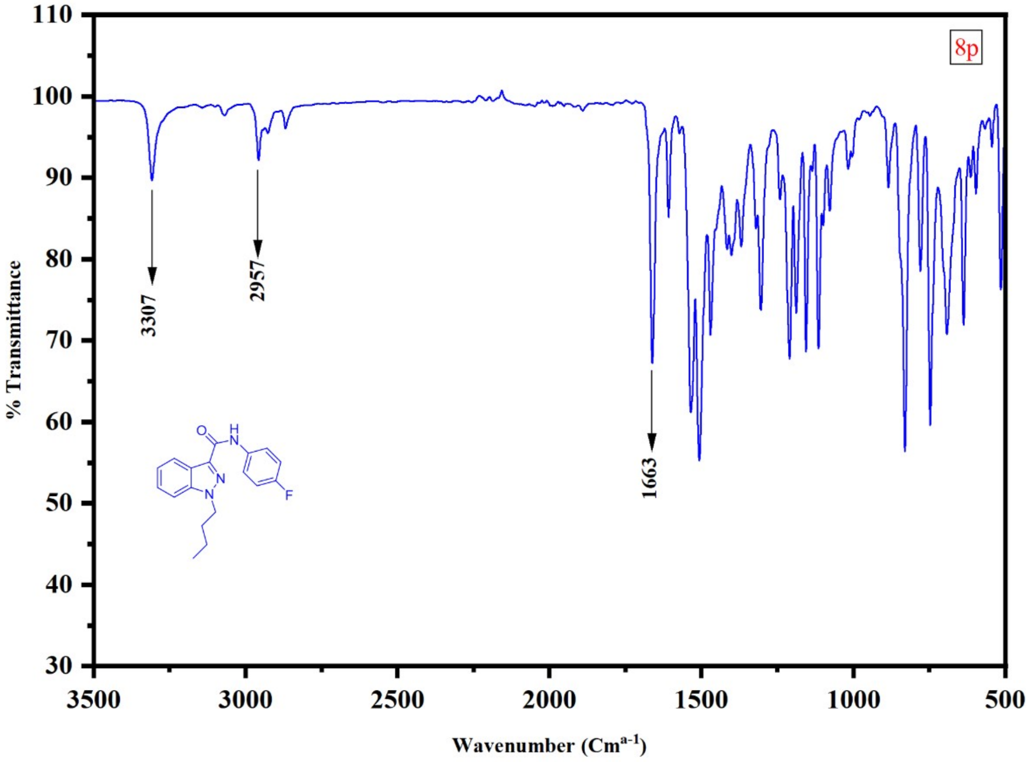

HRMS of 1-butyl-N-(4-fluorophenyl)-1H-indazole-3-carboxamide (8p).

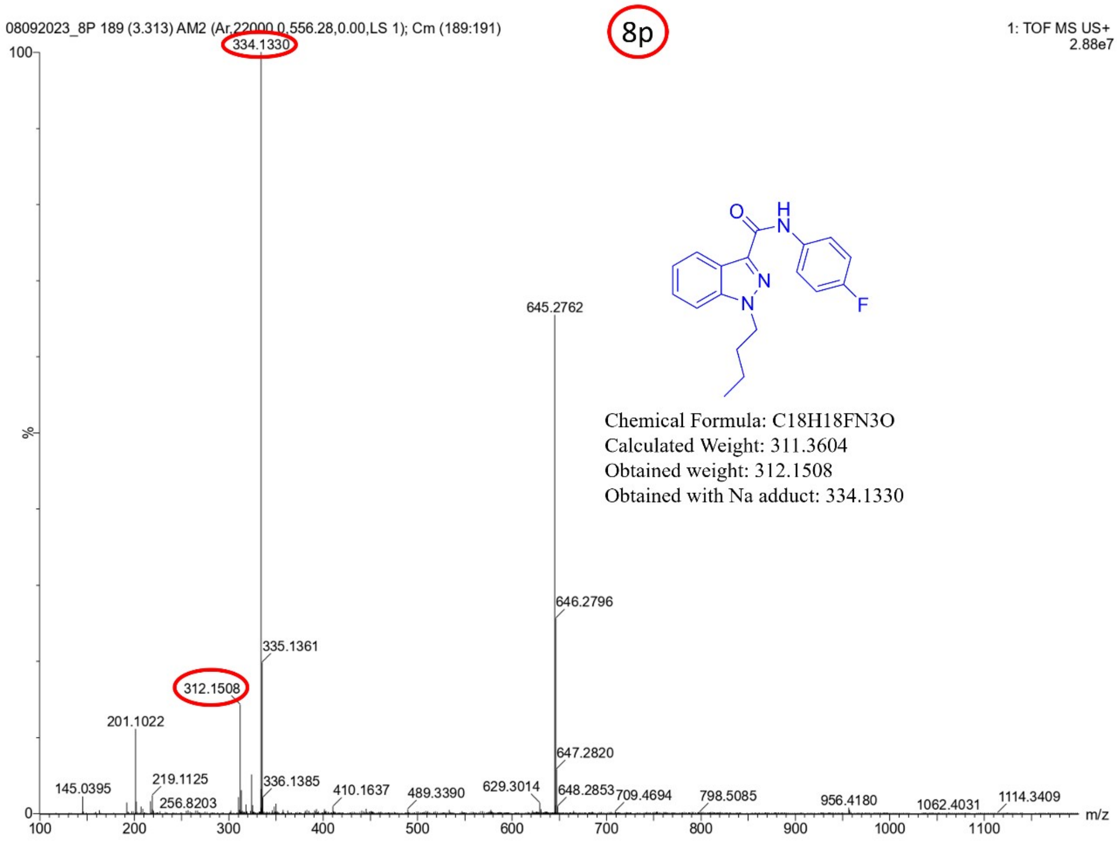

^{1}H-NMR [100MHz, DMSO-d_6] spectrum of 1-butyl-N-(2-methoxyphenyl)-1H-indazole-3-carboxamide (8q).

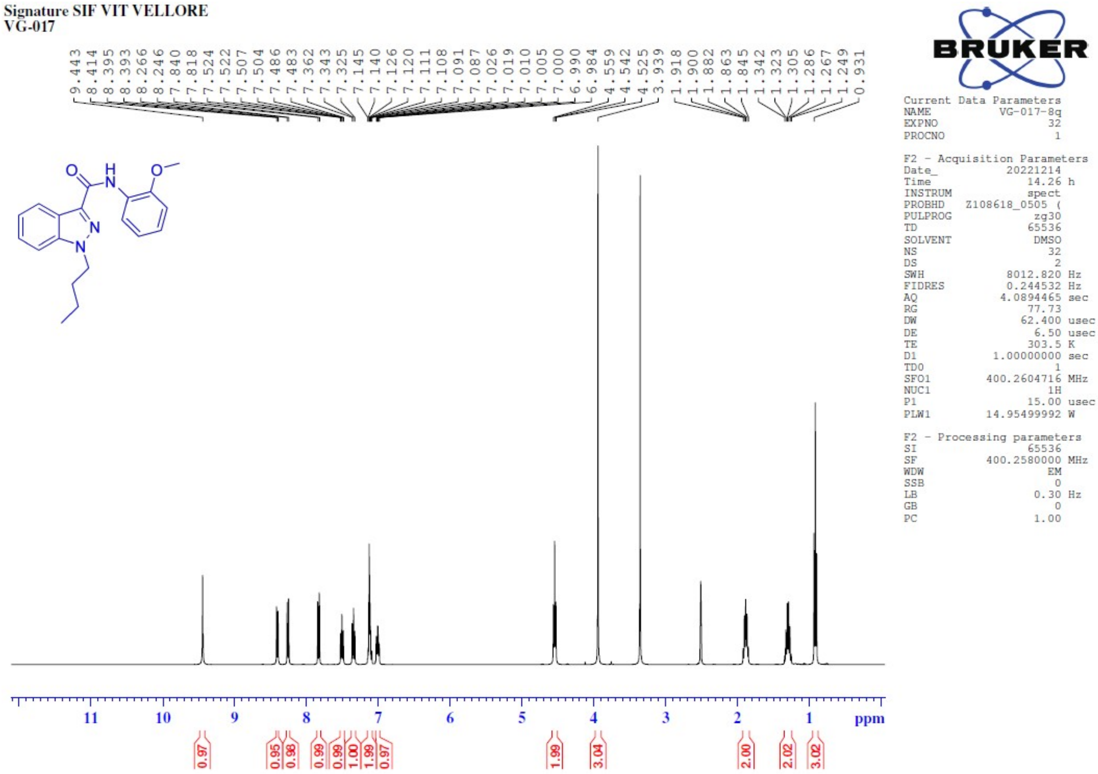

^{1}H-NMR [400MHz, DMSO-d_6] spectrum of 1-butyl-N-(2-methoxyphenyl)-1H-indazole-3-carboxamide (8q).

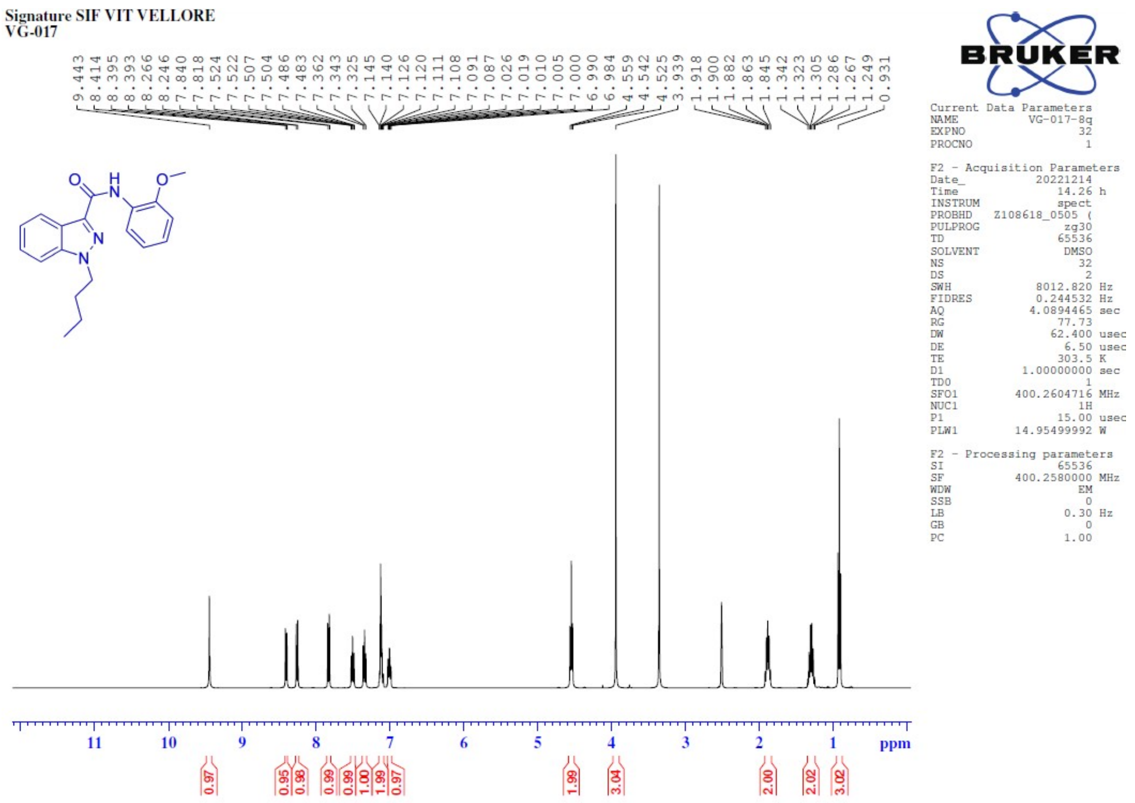

^{13}C-NMR [100MHz, DMSO-d_6] spectrum of 1-butyl-N-(2-methoxyphenyl)-1H-indazole-3-carboxamide (8q).

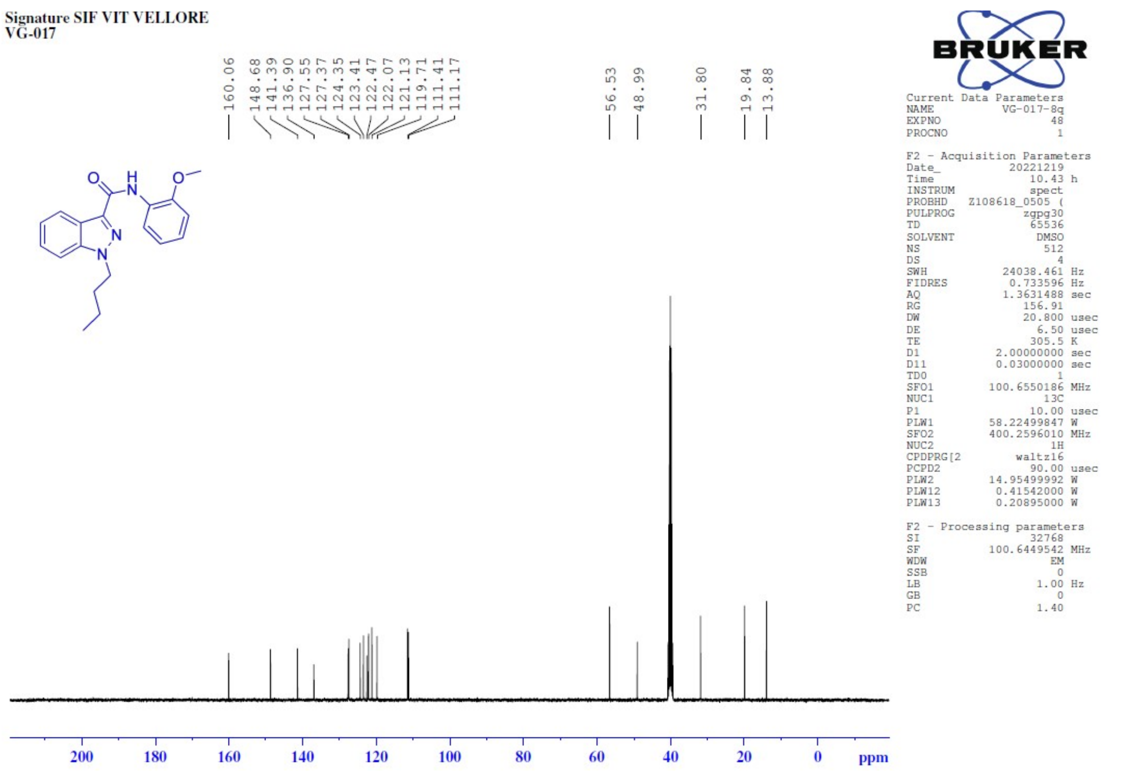

135-DEPT-NMR [100MHz, DMSO-d_6] spectrum of 1-butyl-N-(2-methoxyphenyl)-1H-indazole-3-carboxamide (8q).

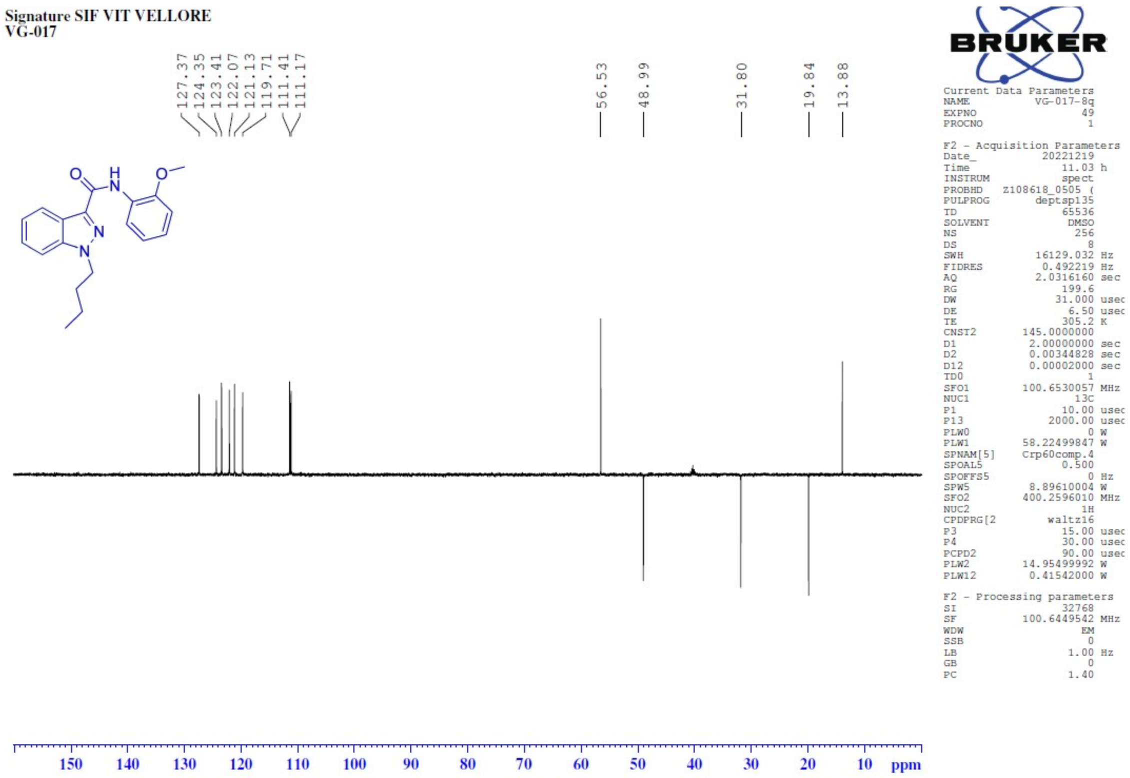

FT-IR spectrum of 1-butyl-N-(2-methoxyphenyl)-1H-indazole-3-carboxamide (8q).

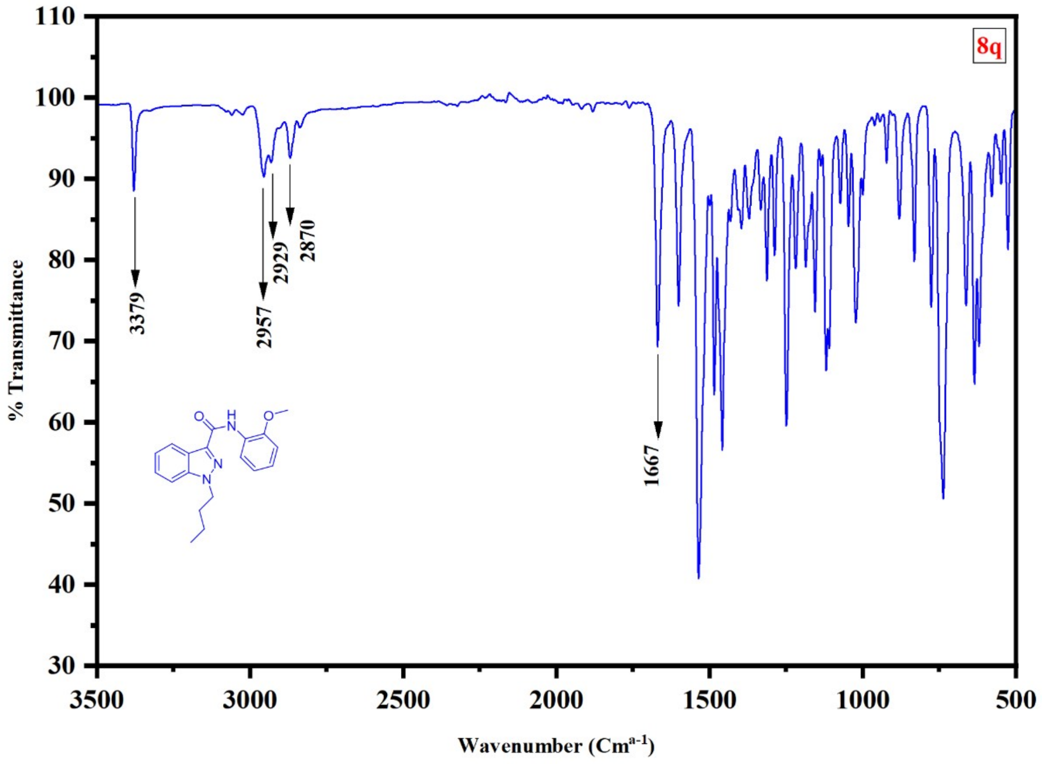

HRMS of 1-butyl-N-(2-methoxyphenyl)-1H-indazole-3-carboxamide (8q).

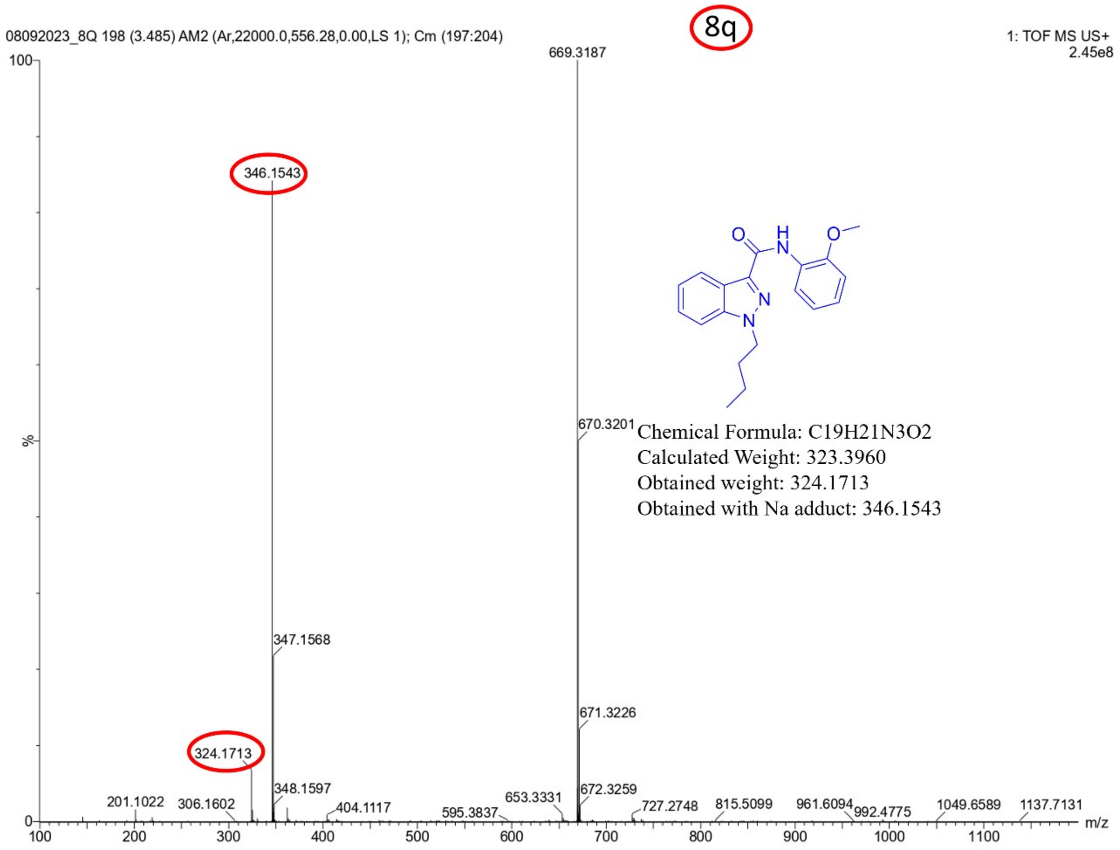

^{1}H-NMR [400MHz, DMSO-d_6] spectrum of 1-butyl-N-(3-hydroxyphenyl)-1H-indazole-3-carboxamide (8r).

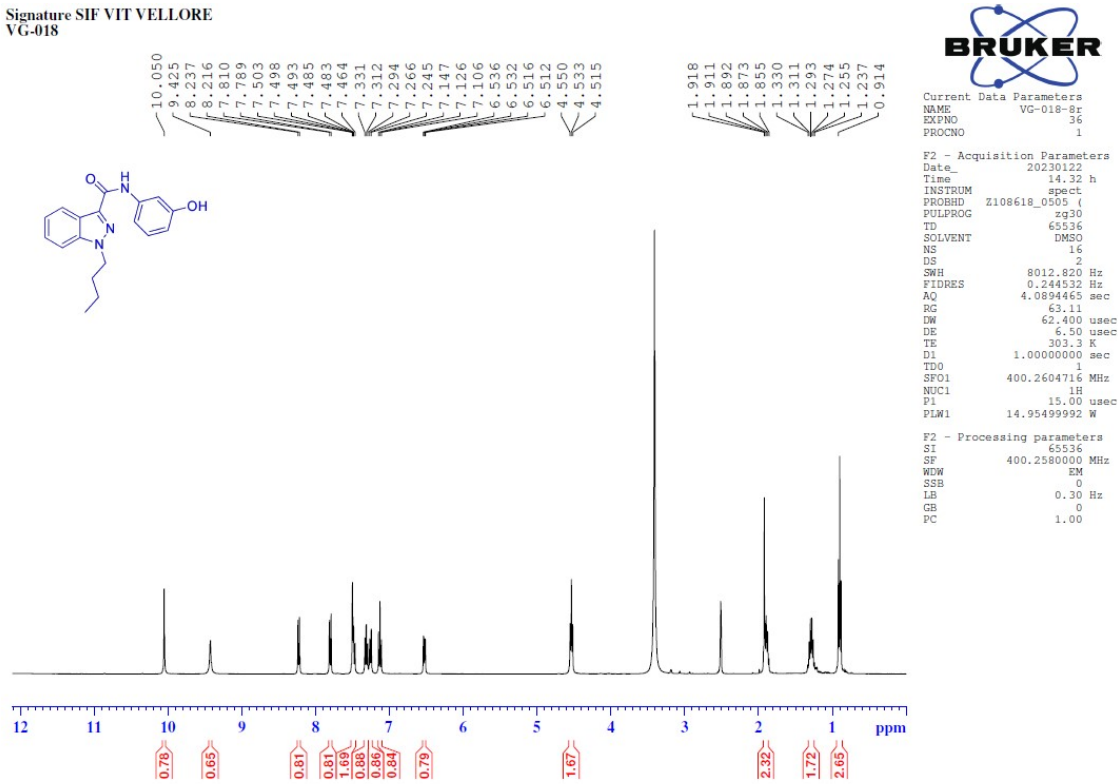

^{13}C-NMR [100MHz, DMSO-d_6] spectrum of 1-butyl-N-(3-hydroxyphenyl)-1H-indazole-3-carboxamide (8r).

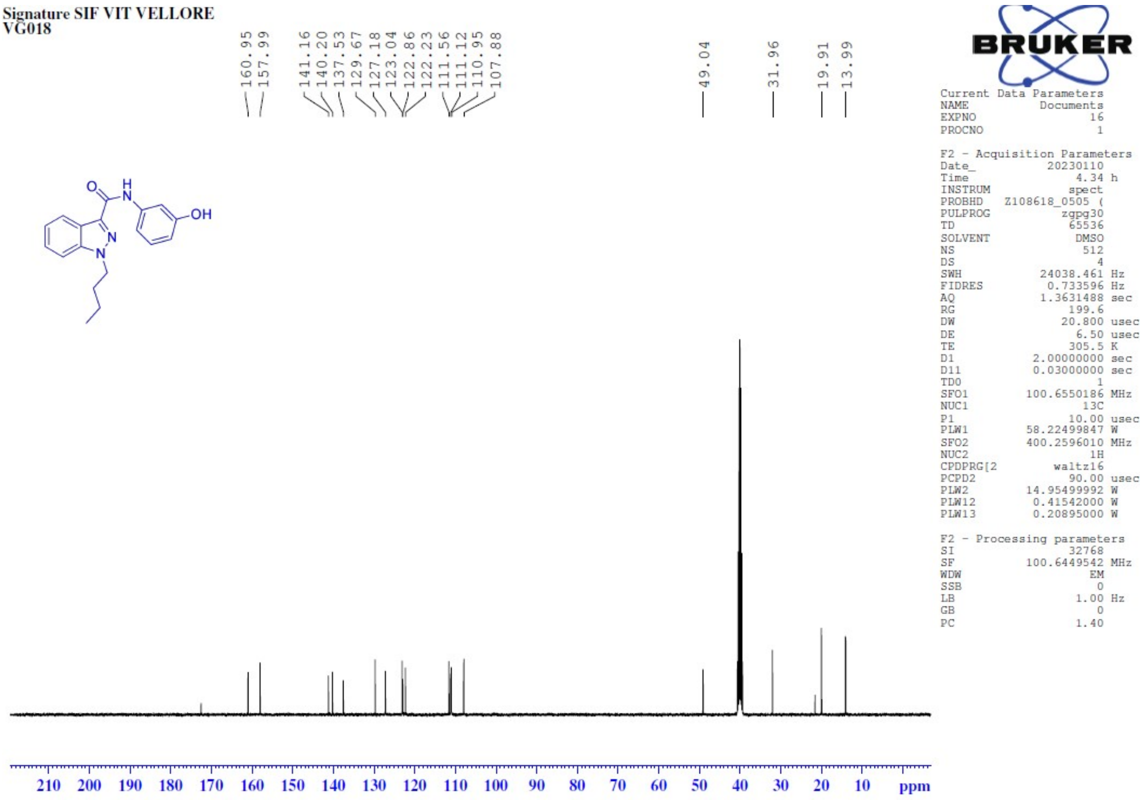

135-DEPT-NMR [100MHz, DMSO-d_6] spectrum of 1-butyl-N-(3-hydroxyphenyl)-1H-indazole-3-carboxamide (8r).

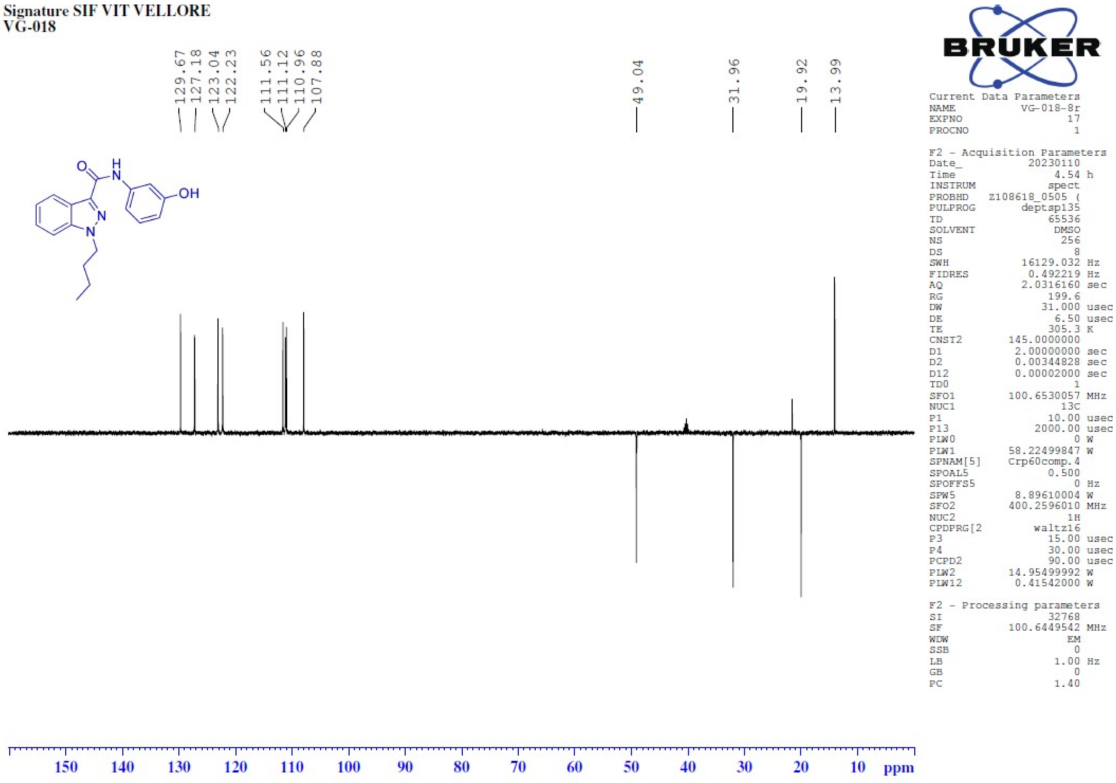

COSY-NMR [400MHz, DMSO-d_6] spectrum of 1-butyl-N-(3-hydroxyphenyl)-1H-indazole-3-carboxamide (8r).

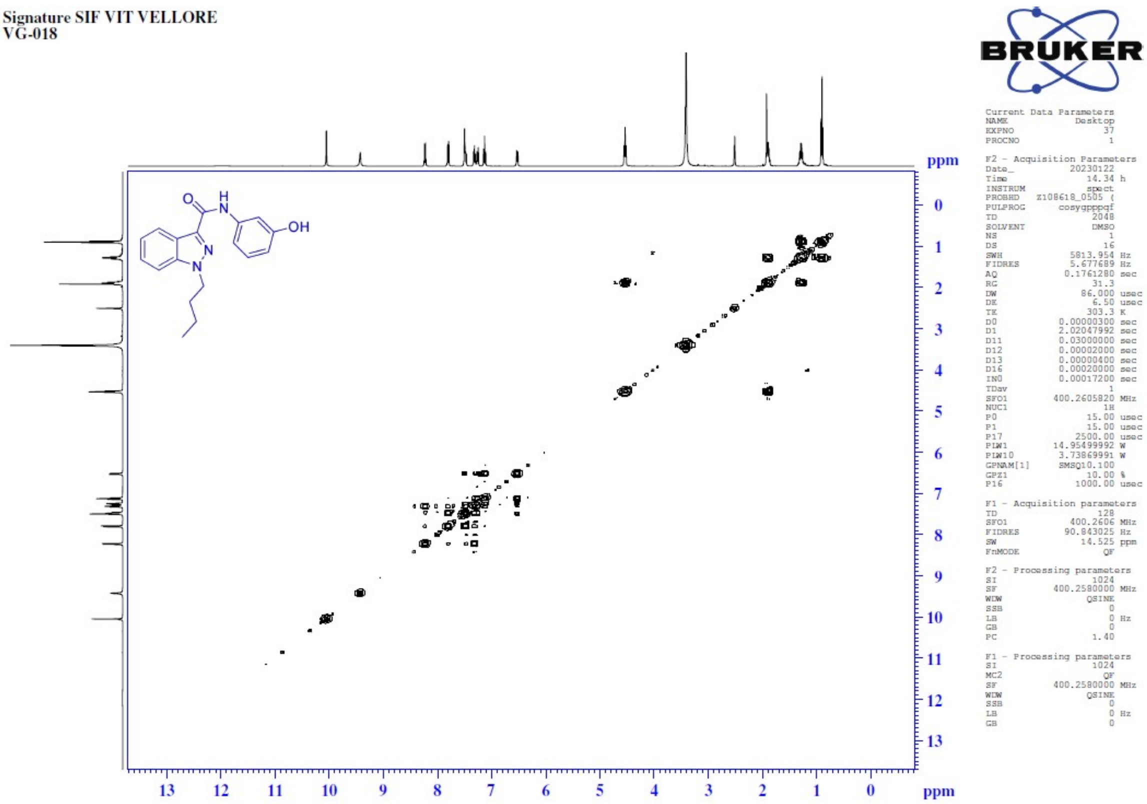

HSQC-NMR [400MHz, DMSO-d_6] spectrum of 1-butyl-N-(3-hydroxyphenyl)-1H-indazole-3-carboxamide (8r).

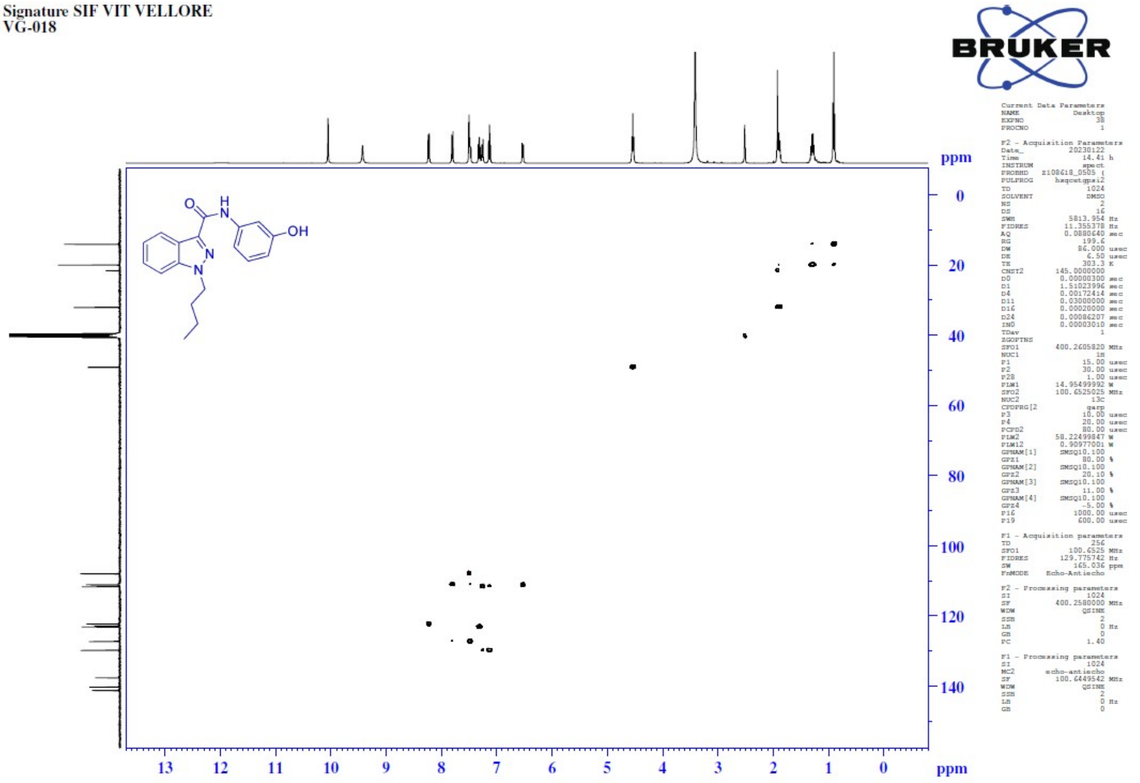

FT-IR spectrum of 1-butyl-N-(3-hydroxyphenyl)-1H-indazole-3-carboxamide (8r).

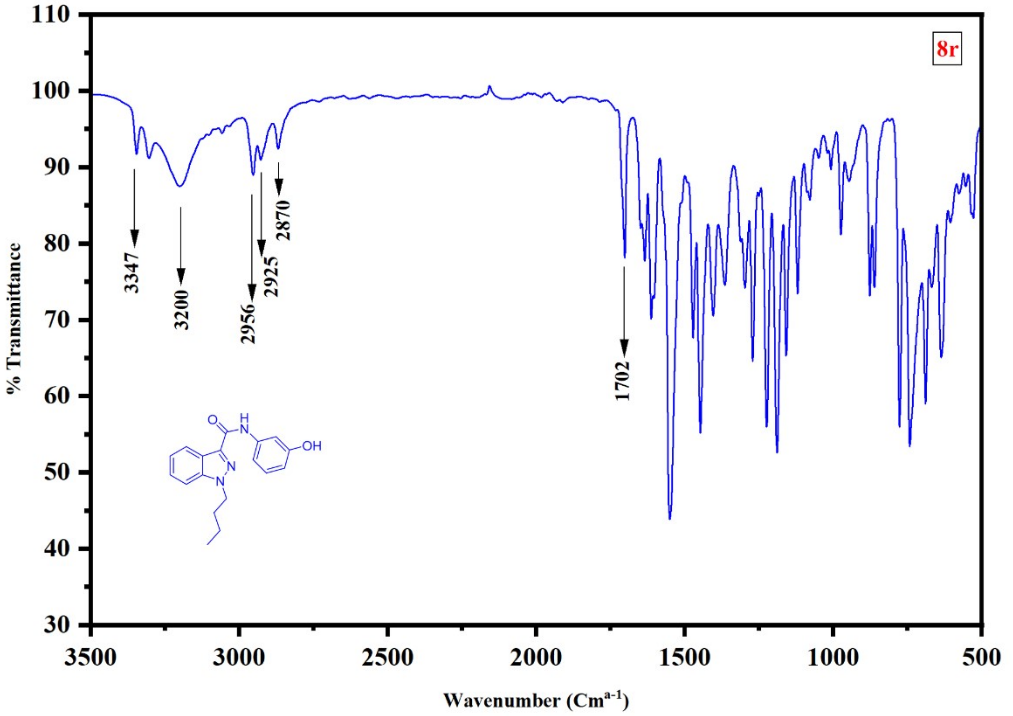

HRMS of 1-butyl-N-(3-hydroxyphenyl)-1H-indazole-3-carboxamide (8r).

^{1}H-NMR [400MHz, DMSO-d_6] spectrum of 1-butyl-N-(4H-1,2,4-triazol-4-yl)-1H-indazole-3-carboxamide (8s).

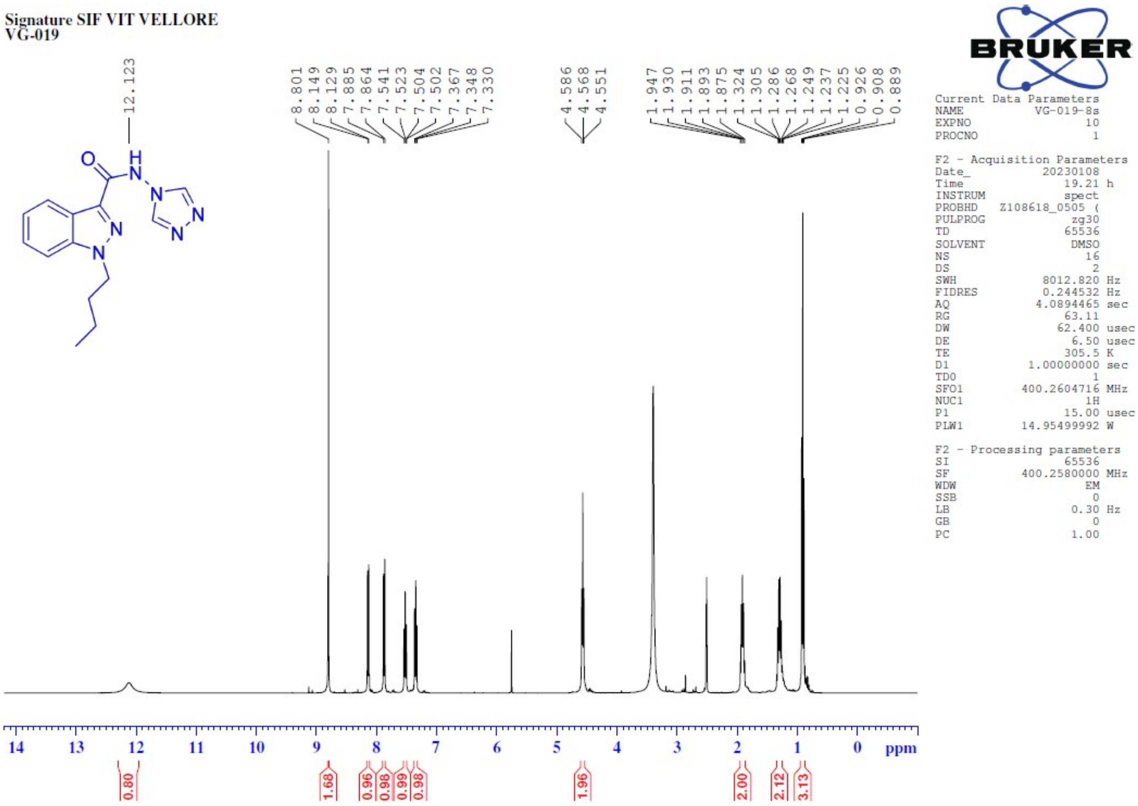

^{13}C-NMR [100MHz, DMSO-d_6] spectrum of 1-butyl-N-(4H-1,2,4-triazol-4-yl)-1H-indazole-3-carboxamide (8s).

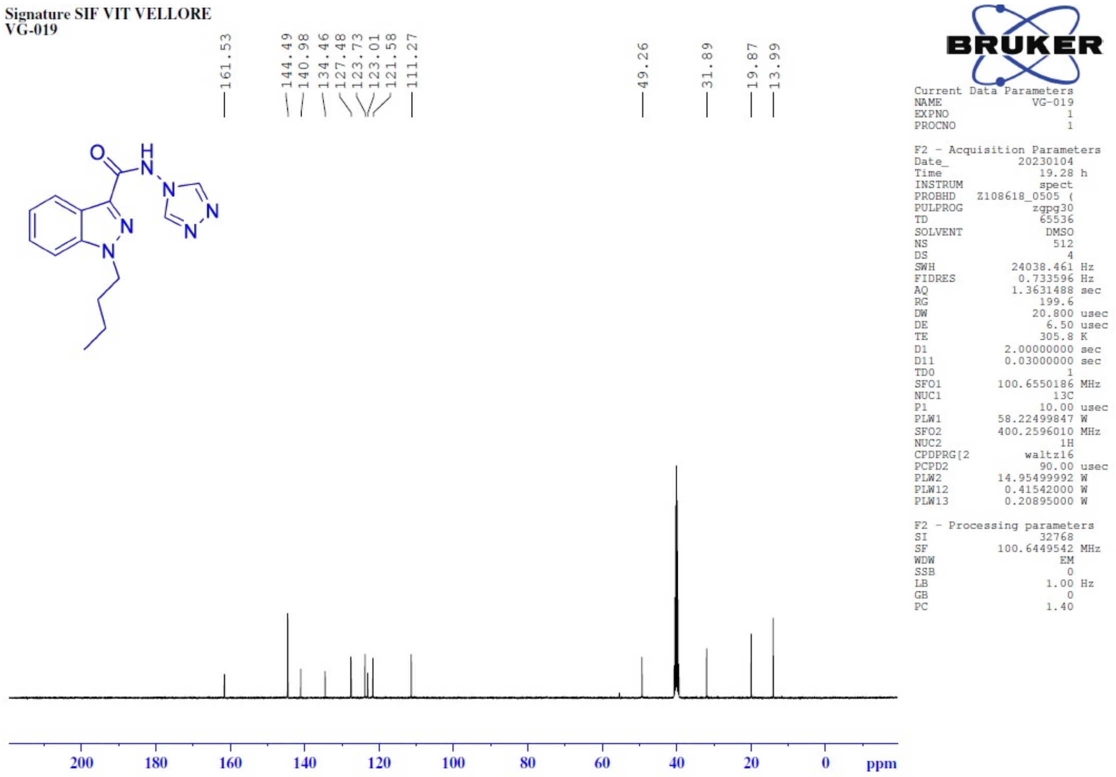

135-DEPT-NMR [100MHz, DMSO-d_6] spectrum of 1-butyl-N-(4H-1,2,4-triazol-4-yl)-1H-indazole-3-carboxamide (8s).

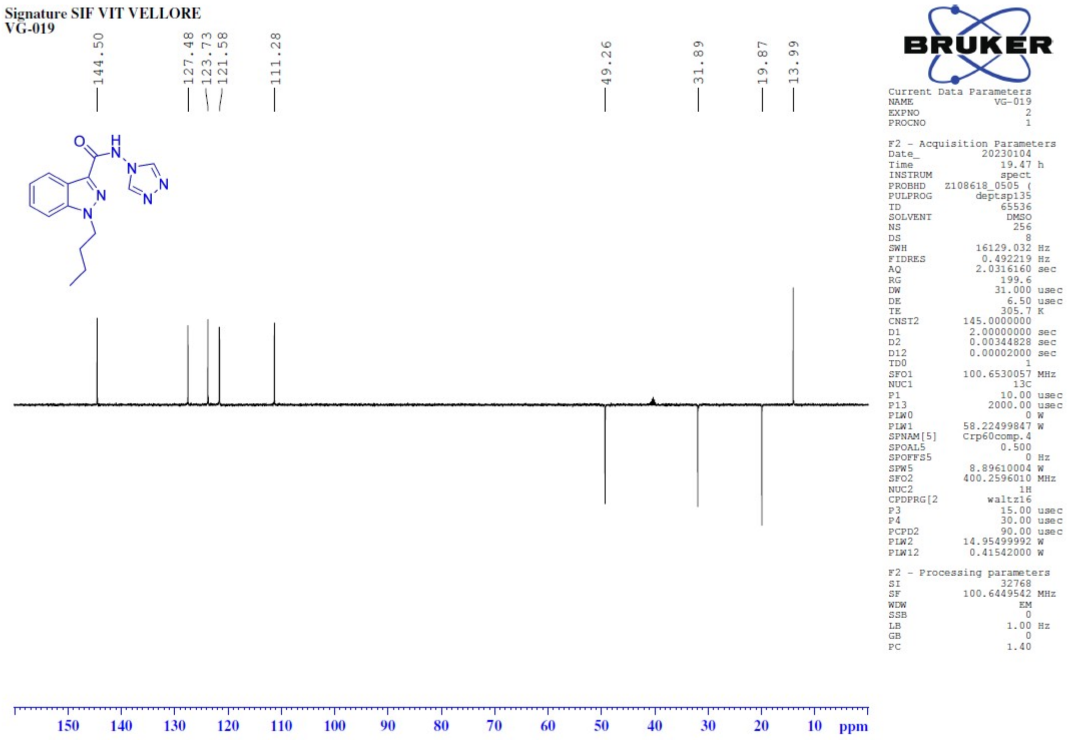

COSY-NMR [400MHz, DMSO-d_6] spectrum of 1-butyl-N-(4H-1,2,4-triazol-4-yl)-1H-indazole-3-carboxamide (8s).

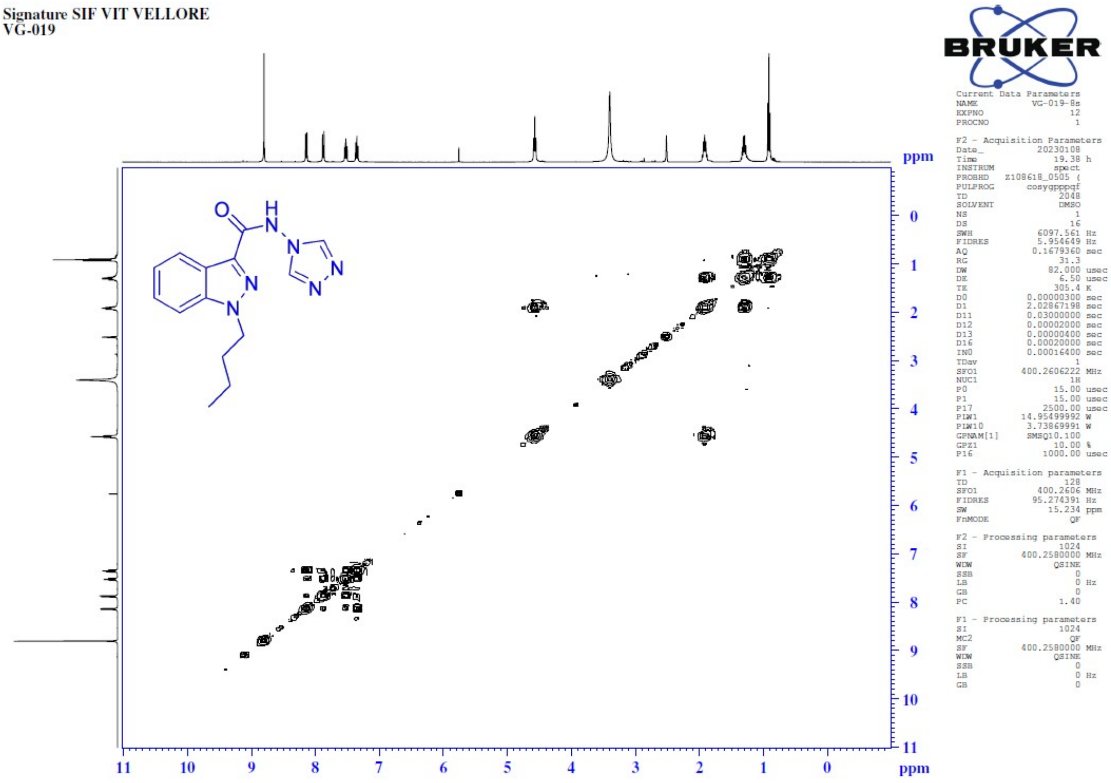

HSQC-NMR [400MHz, DMSO-d_6] spectrum of 1-butyl-N-(4H-1,2,4-triazol-4-yl)-1H-indazole-3-carboxamide (8s).

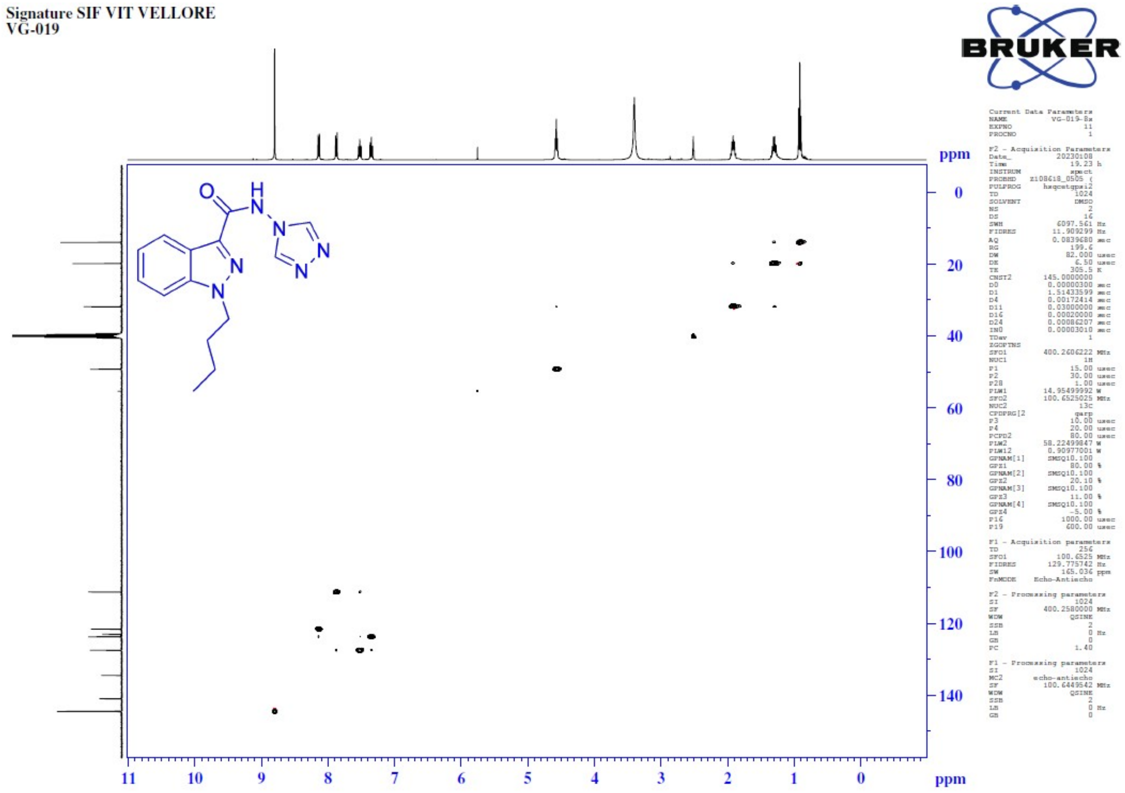

FT-IR spectrum of 1-butyl-N-(4H-1,2,4-triazol-4-yl)-1H-indazole-3-carboxamide (8s).

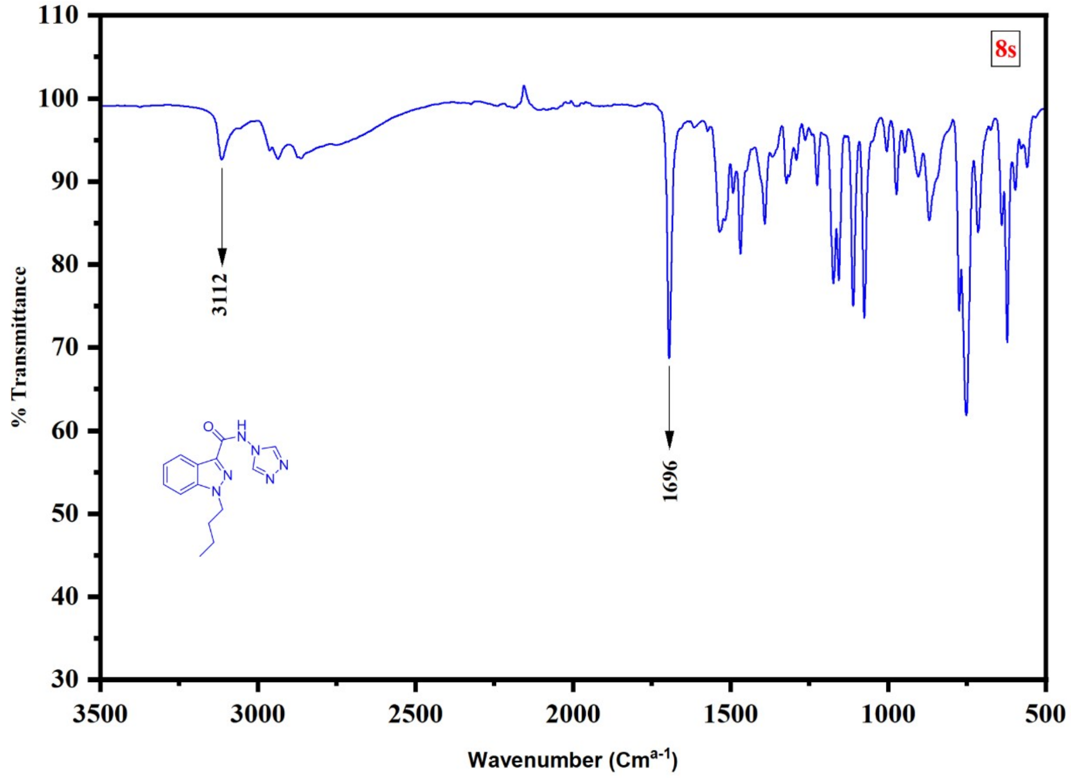

HRMS of 1-butyl-N-(4H-1,2,4-triazol-4-yl)-1H-indazole-3-carboxamide (8s).

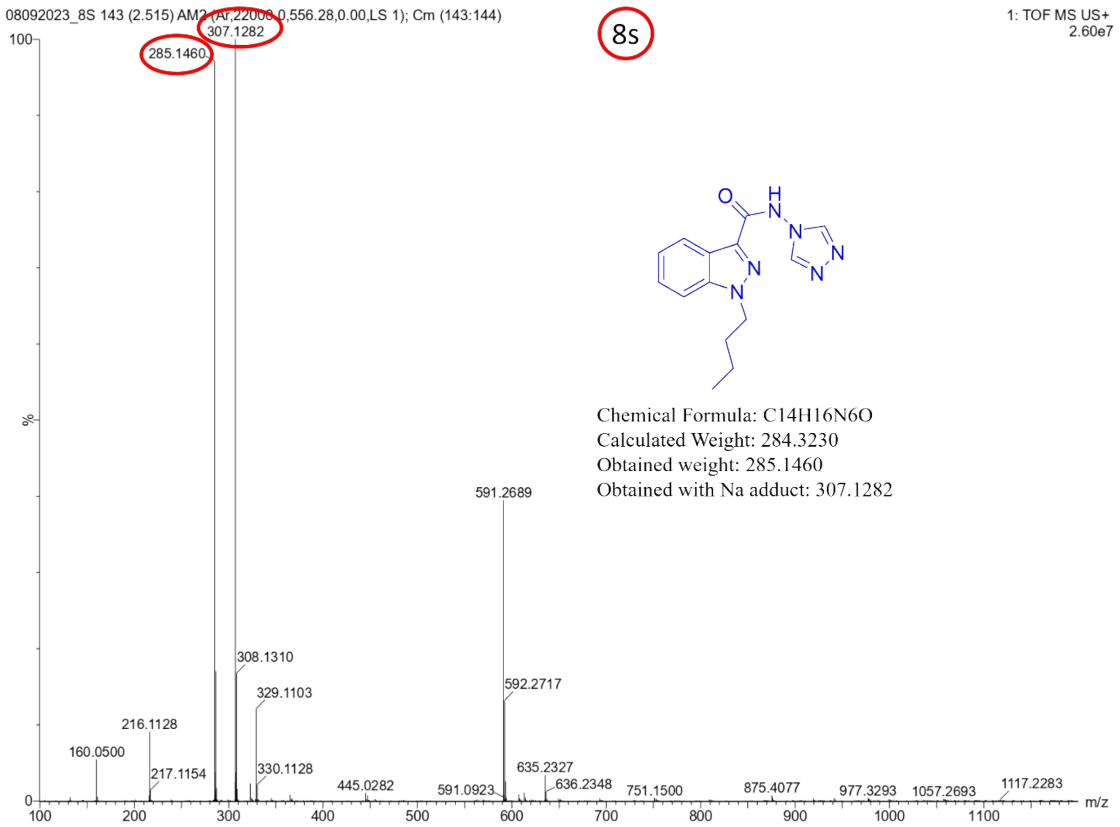

^{1}H-NMR [400MHz, DMSO-d_6] spectrum of N-(2-amino-4-nitrophenyl)-1-butyl-1H-indazole-3-carboxamide (8t).

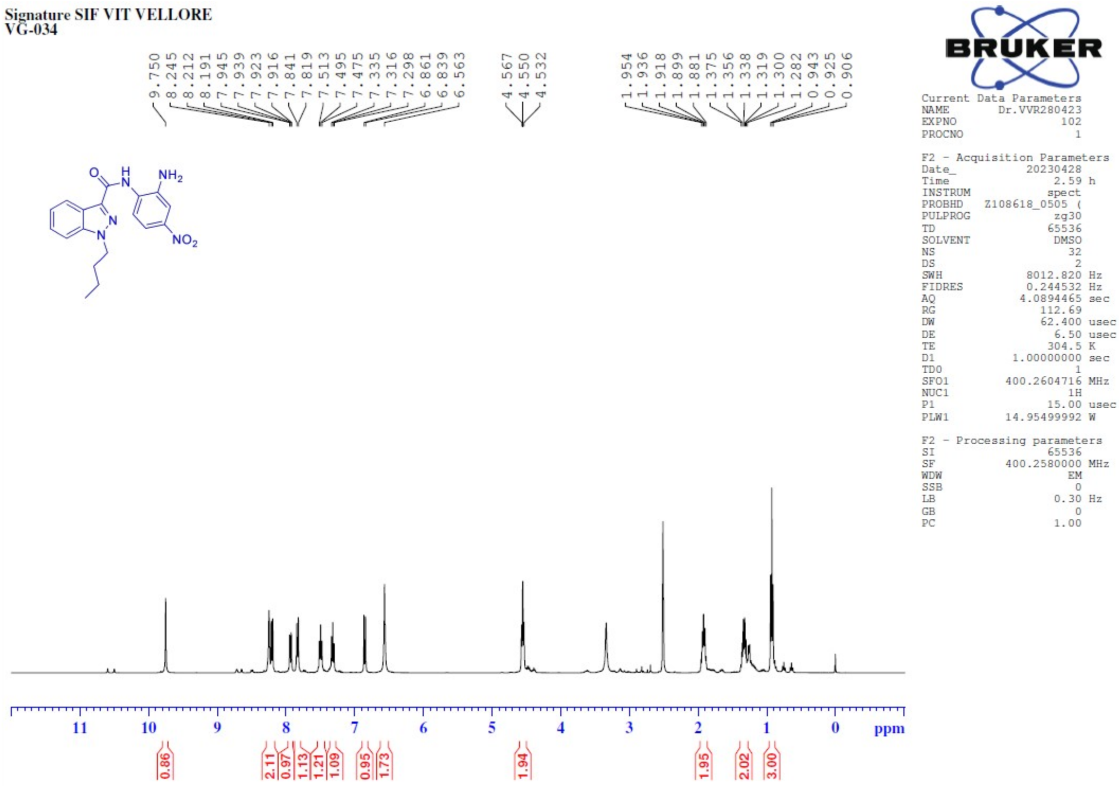

^{13}C-NMR [400MHz, DMSO-d_6] spectrum of N-(2-amino-4-nitrophenyl)-1-butyl-1H-indazole-3-carboxamide (8t).

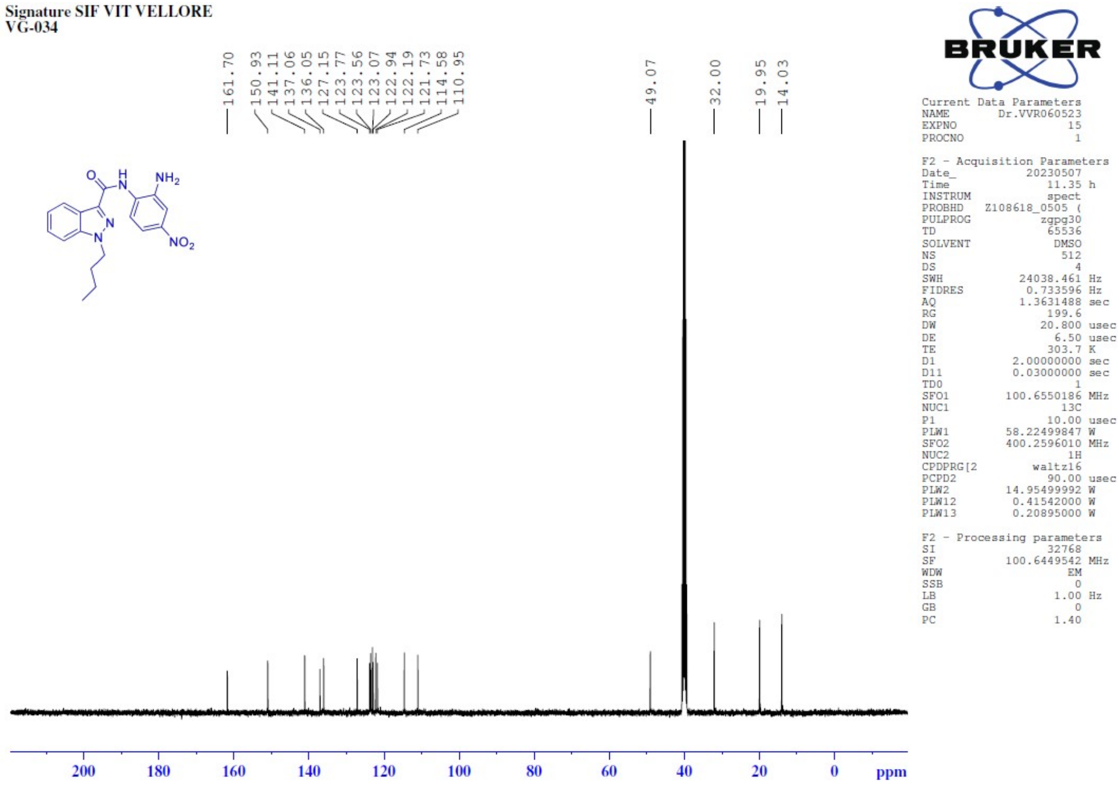

FT-IR spectrum of N-(2-amino-4-nitrophenyl)-1-butyl-1H-indazole-3-carboxamide (8t).

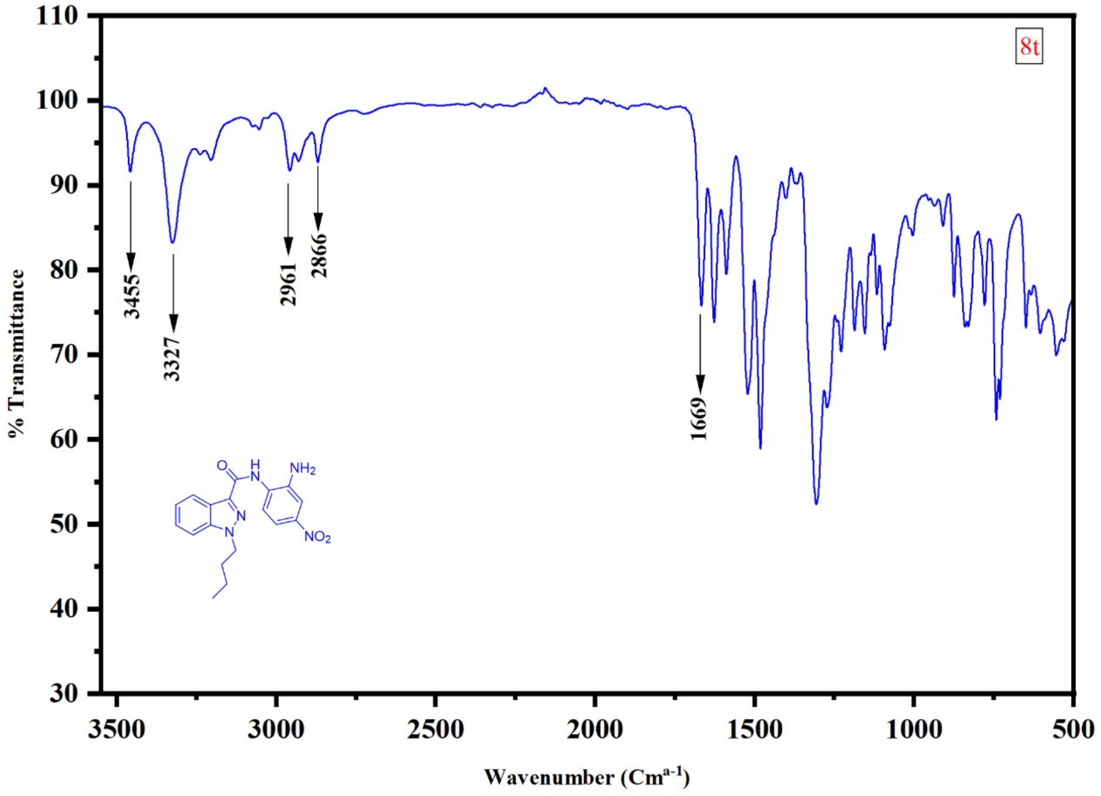

HRMS of N-(2-amino-4-nitrophenyl)-1-butyl-1H-indazole-3-carboxamide (8t).

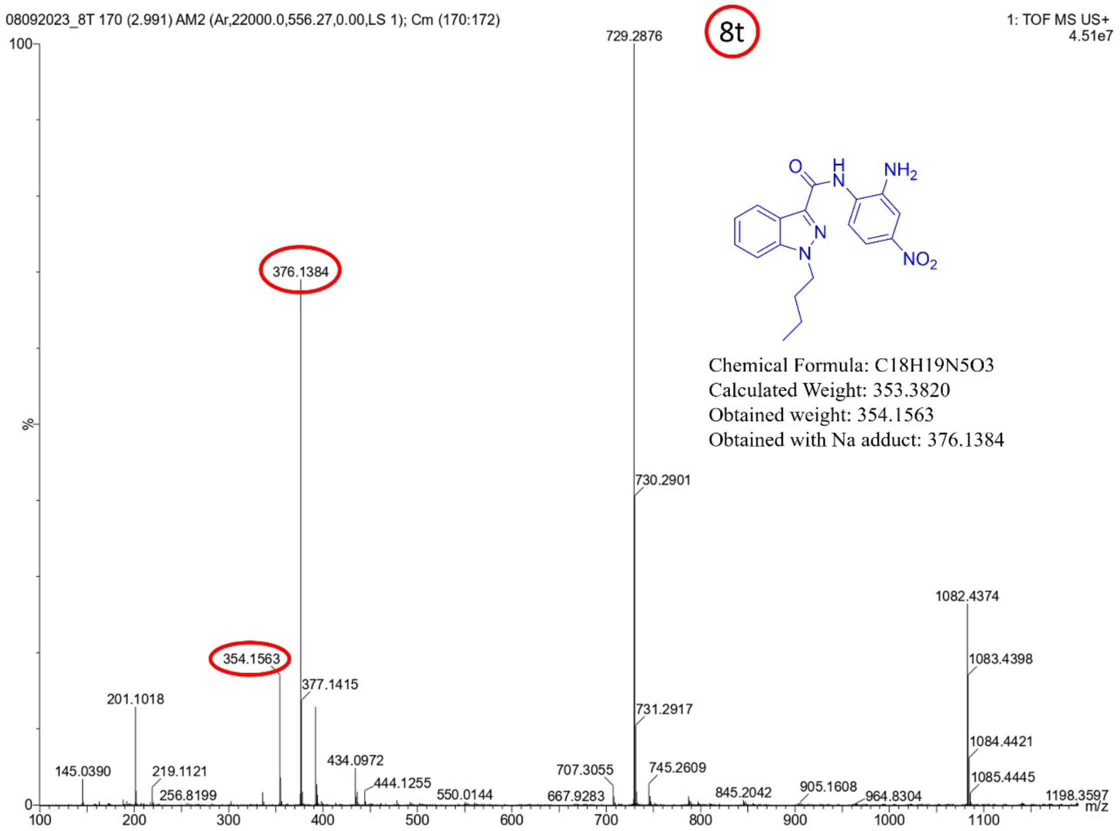

^{1}H-NMR [400MHz, DMSO-d$_6$] spectrum of 1-butyl-N-phenyl-1H-indazole-3-carbohydrazide (8u).

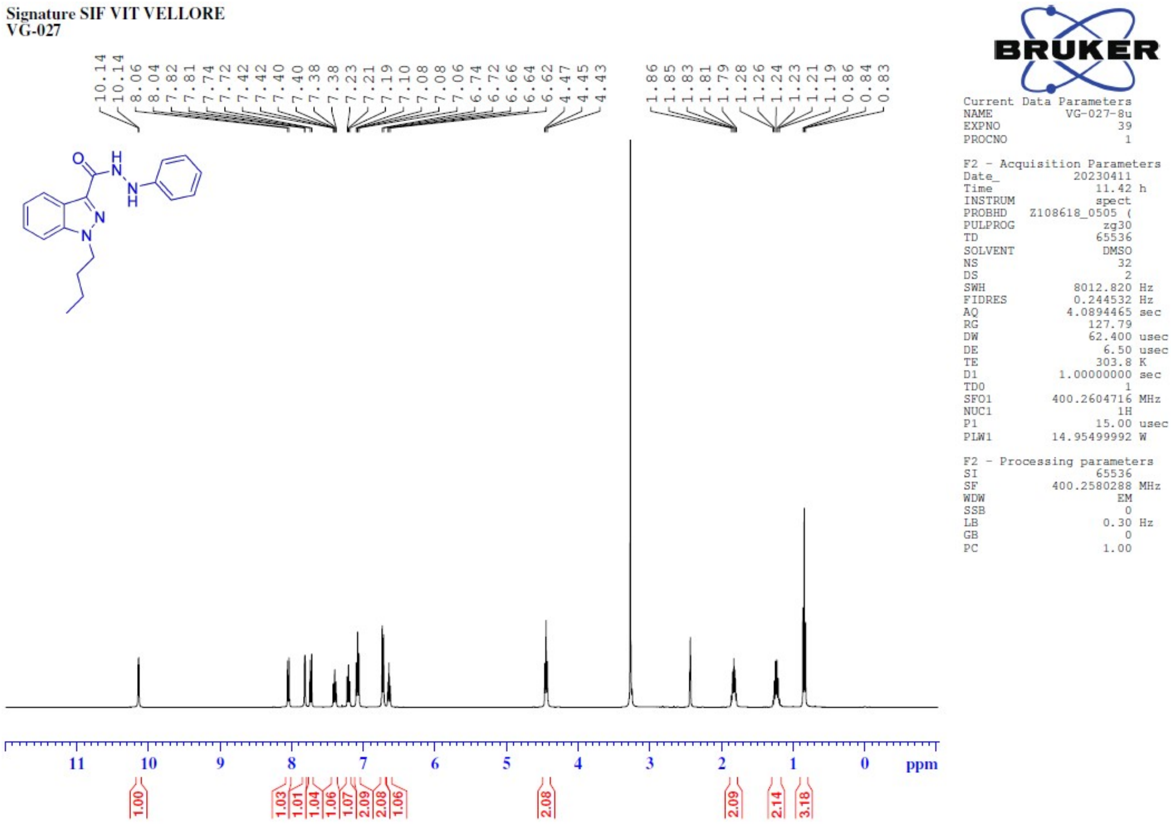

^{13}C-NMR [100MHz, DMSO-d_6] spectrum of 1-butyl-N-phenyl-1H-indazole-3-carbohydrazide (8u).

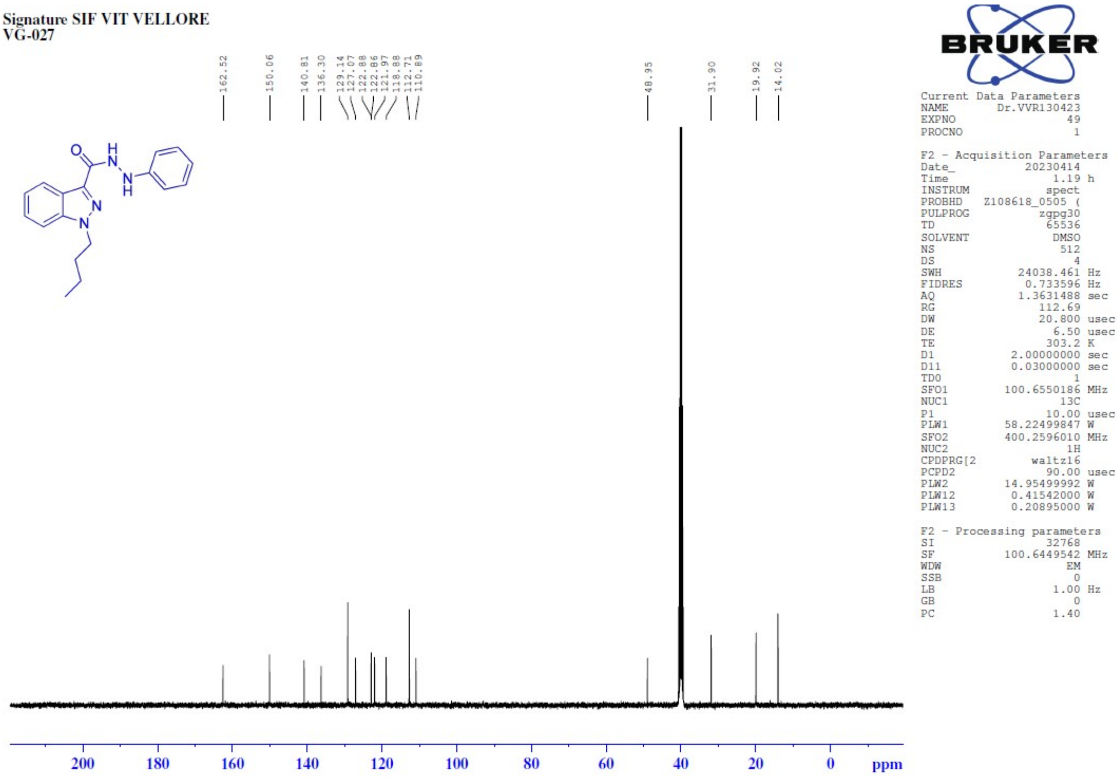

135-DEPT-NMR [100MHz, DMSO-d_6] spectrum of 1-butyl-N-phenyl-1H-indazole-3-carbohydrazide (8u).

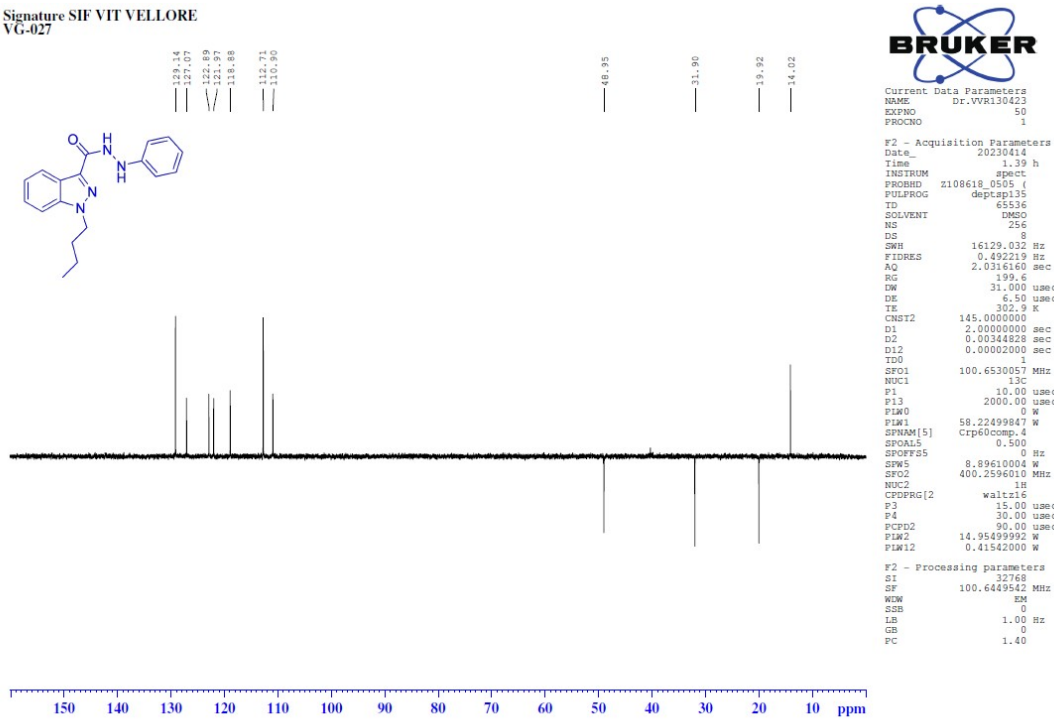

FT-IR spectrum of 1-butyl-N-phenyl-1H-indazole-3-carbohydrazide (8u).

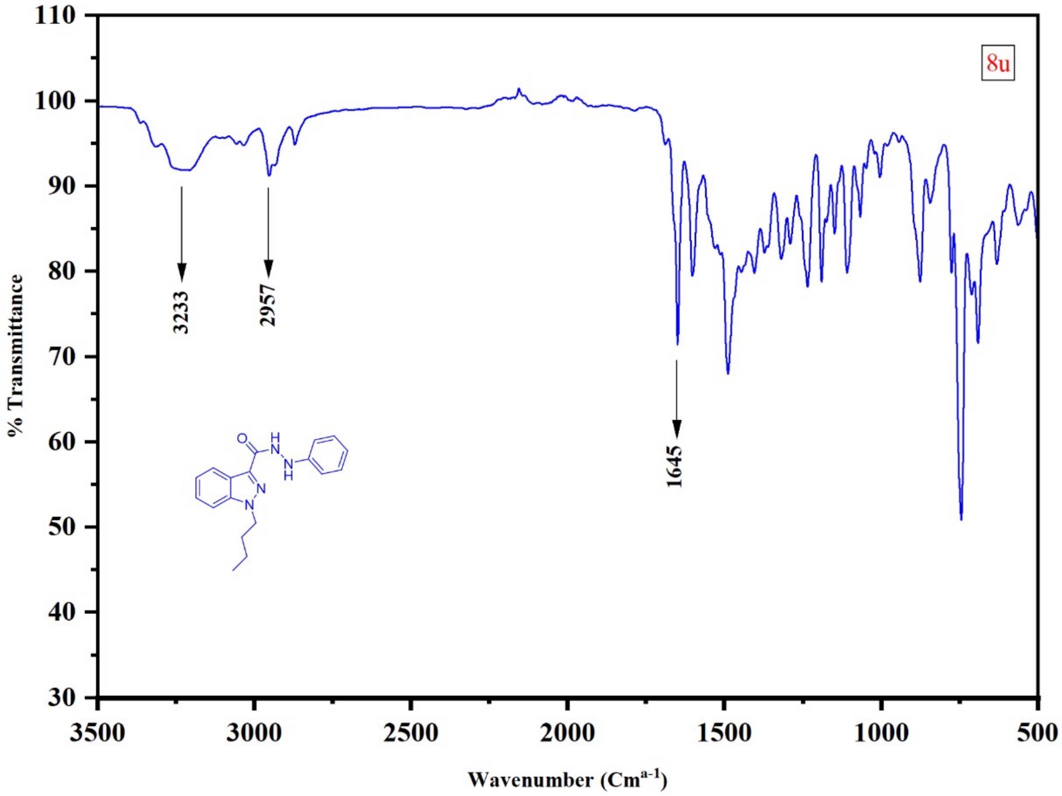

HRMS of 1-butyl-N-phenyl-1H-indazole-3-carbohydrazide (8u).

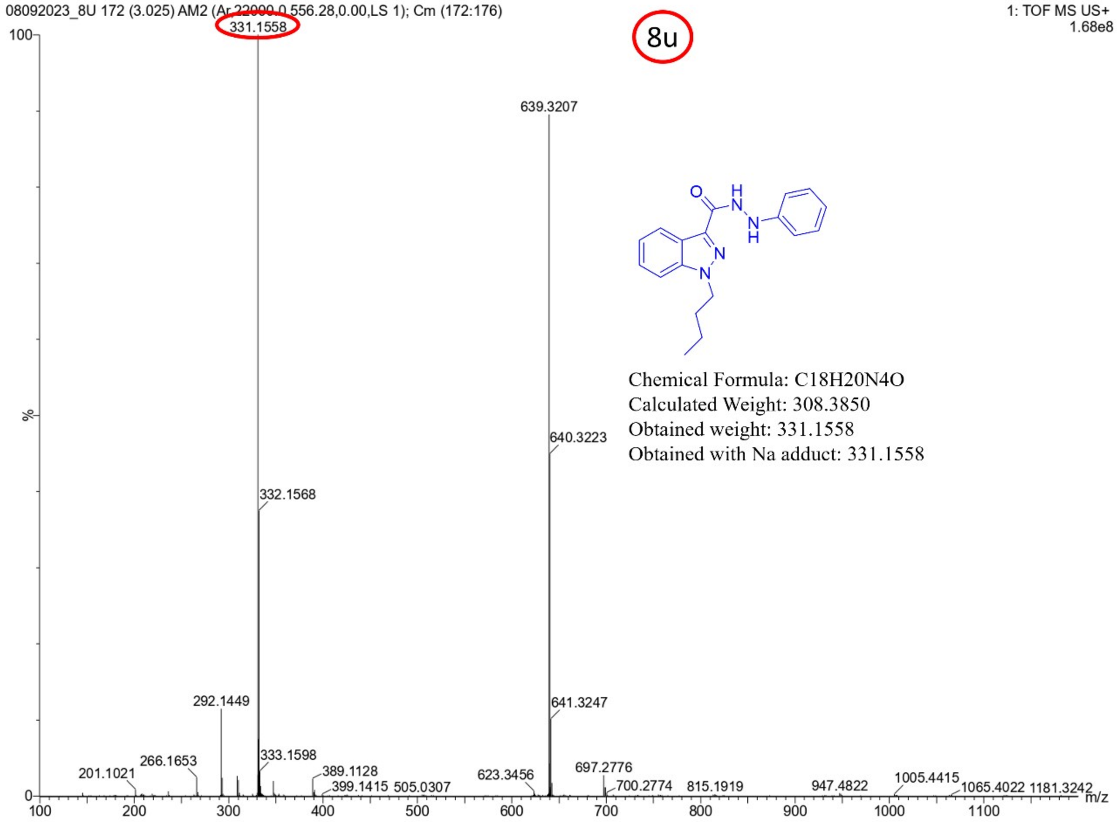

^{1}H-NMR [400MHz, DMSO-d_6] spectrum of 1-butyl-N-(2,4-dinitrophenyl)-1H-indazole-3-carbohydrazide (8v).

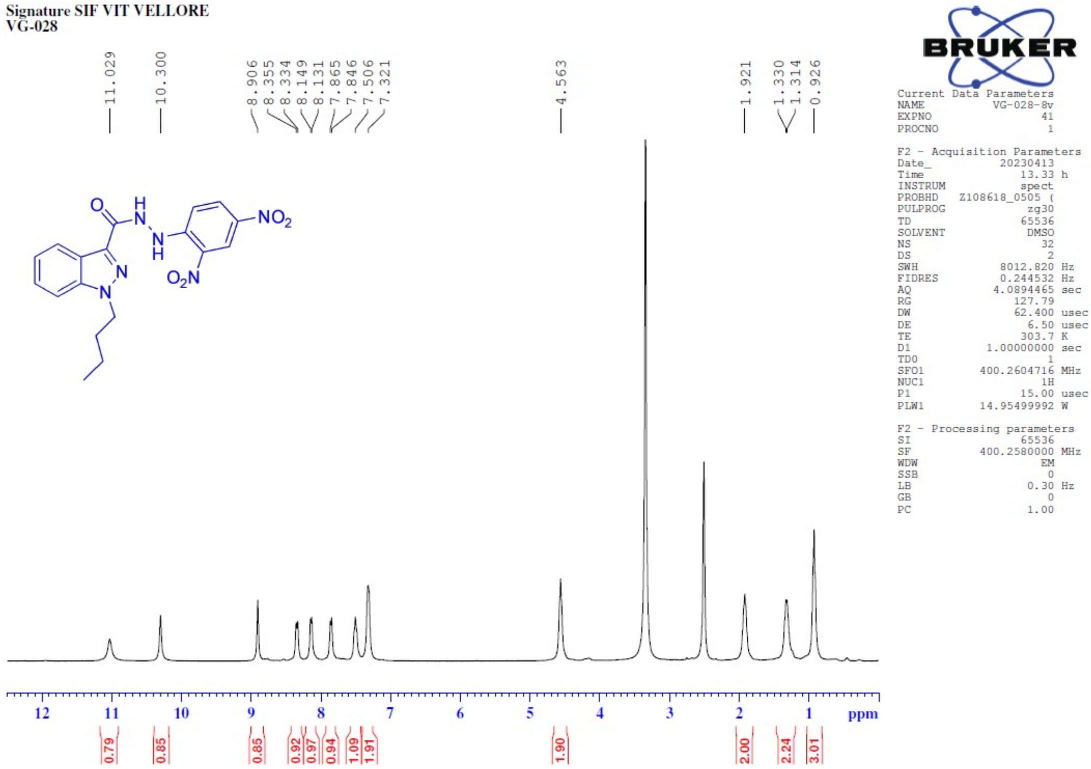

^{13}C-NMR [100MHz, DMSO-d$_6$] spectrum of 1-butyl-N-(2,4-dinitrophenyl)-1H-indazole-3-carbohydrazide (8v).

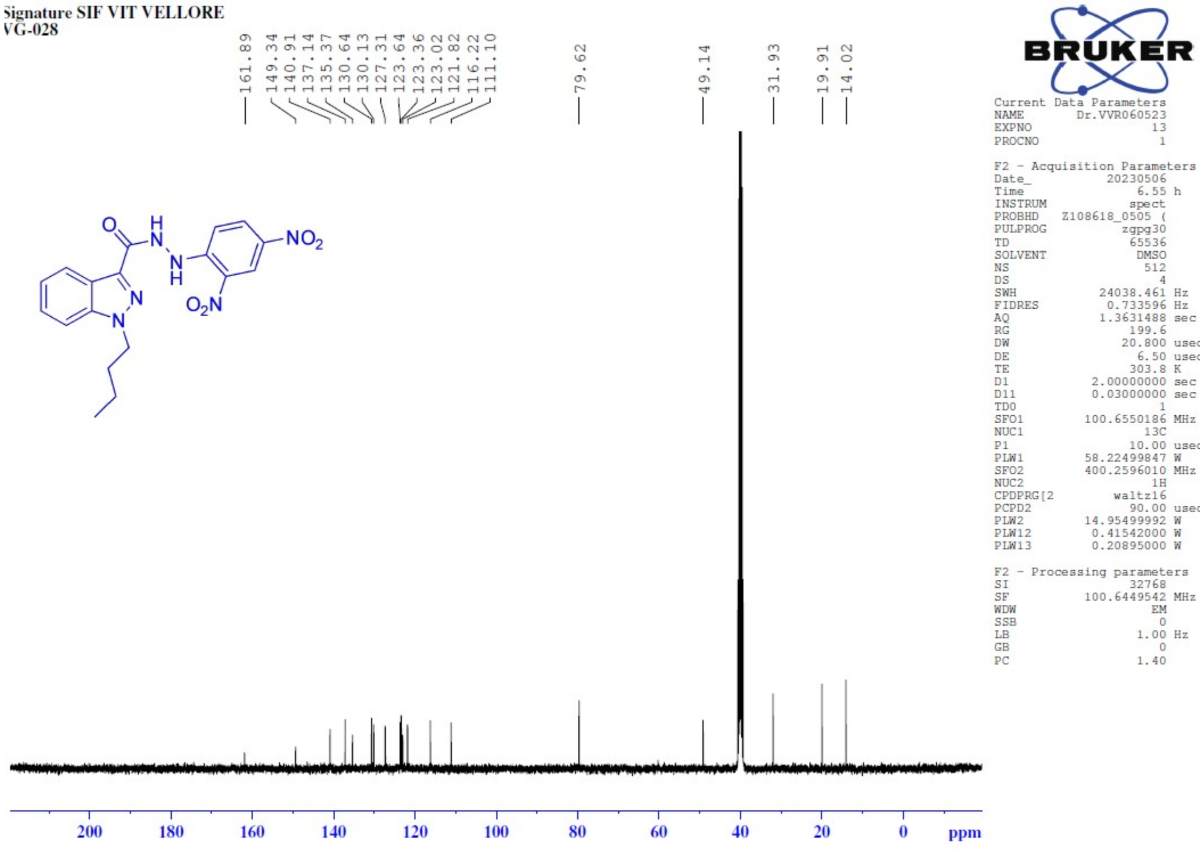

135-DEPT-NMR [100MHz, DMSO-d$_6$] spectrum of 1-butyl-N-(2,4-dinitrophenyl)-1H-indazole-3-carbohydrazide (8v).

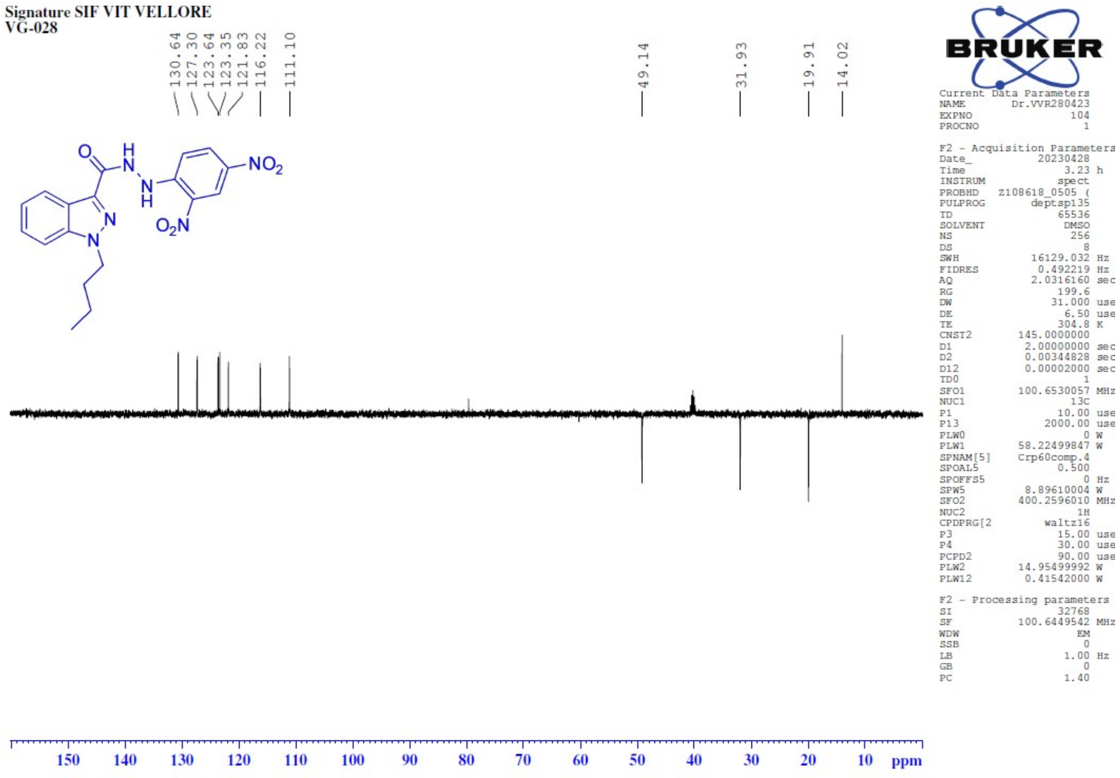

FT-IR spectrum of 1-butyl-N-(2,4-dinitrophenyl)-1H-indazole-3-carbohydrazide (8v).

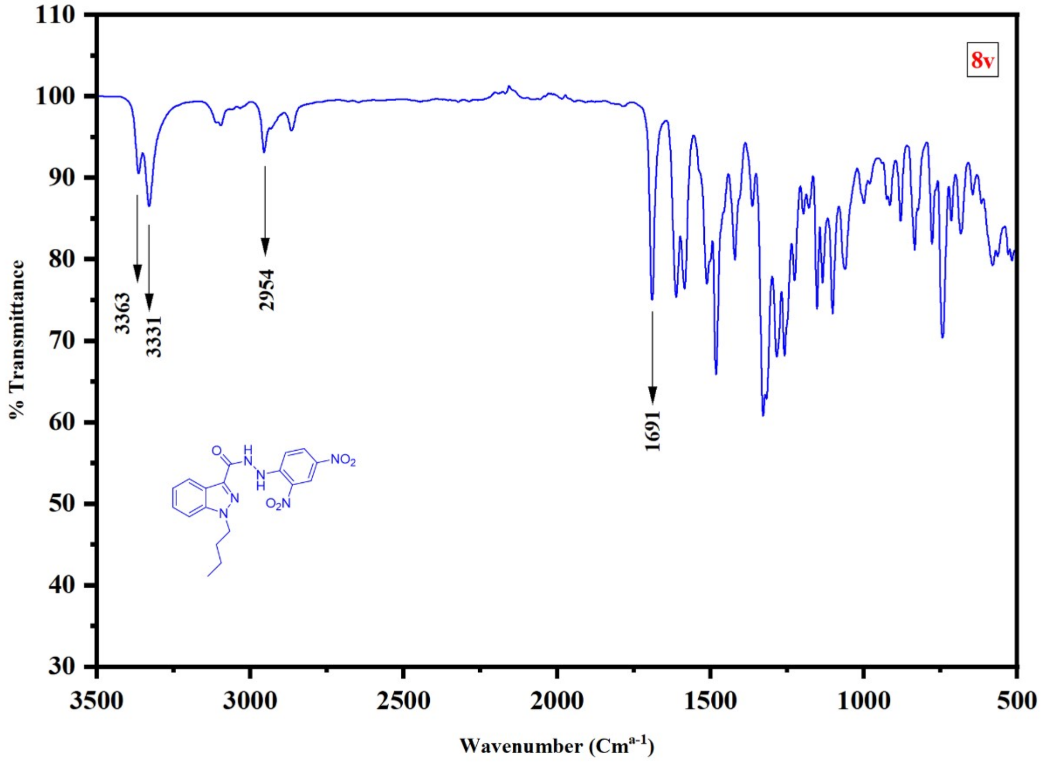

HRMS of 1-butyl-N-(2,4-dinitrophenyl)-1H-indazole-3-carbohydrazide (8v).

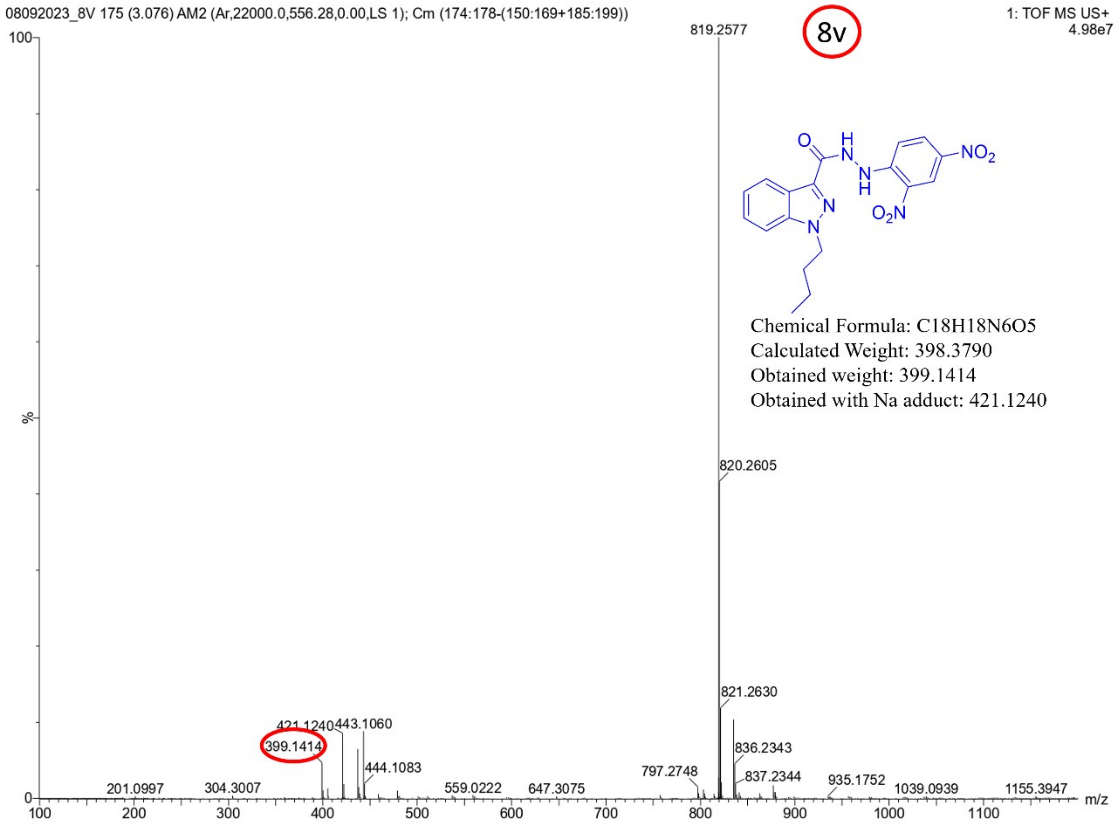

^{1}H-NMR [100MHz, DMSO-d_6] spectrum of 1-butyl-N-(4-cyanophenyl)-1H-indazole-3-carbohydrazide (8W).

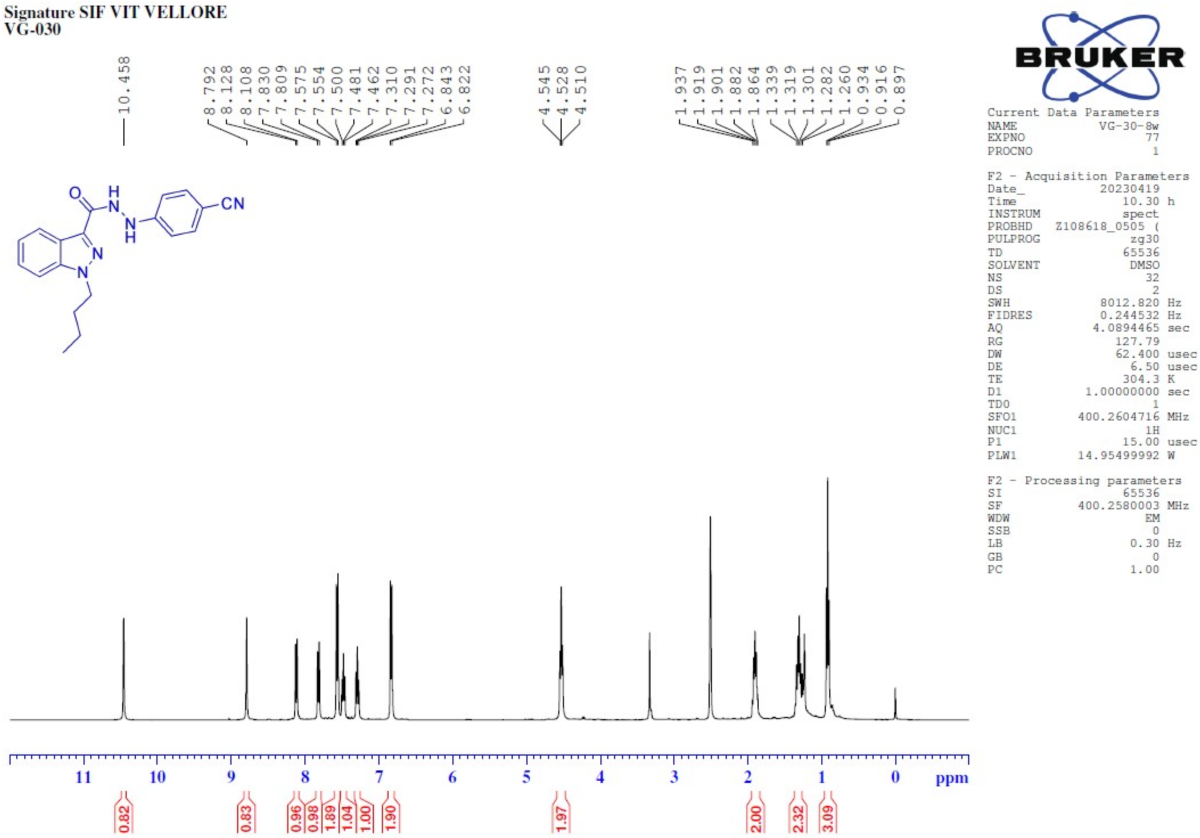

^{13}C-NMR [100MHz, DMSO-d$_6$] spectrum of 1-butyl-N-(4-cyanophenyl)-1H-indazole-3-carbohydrazide (8W).

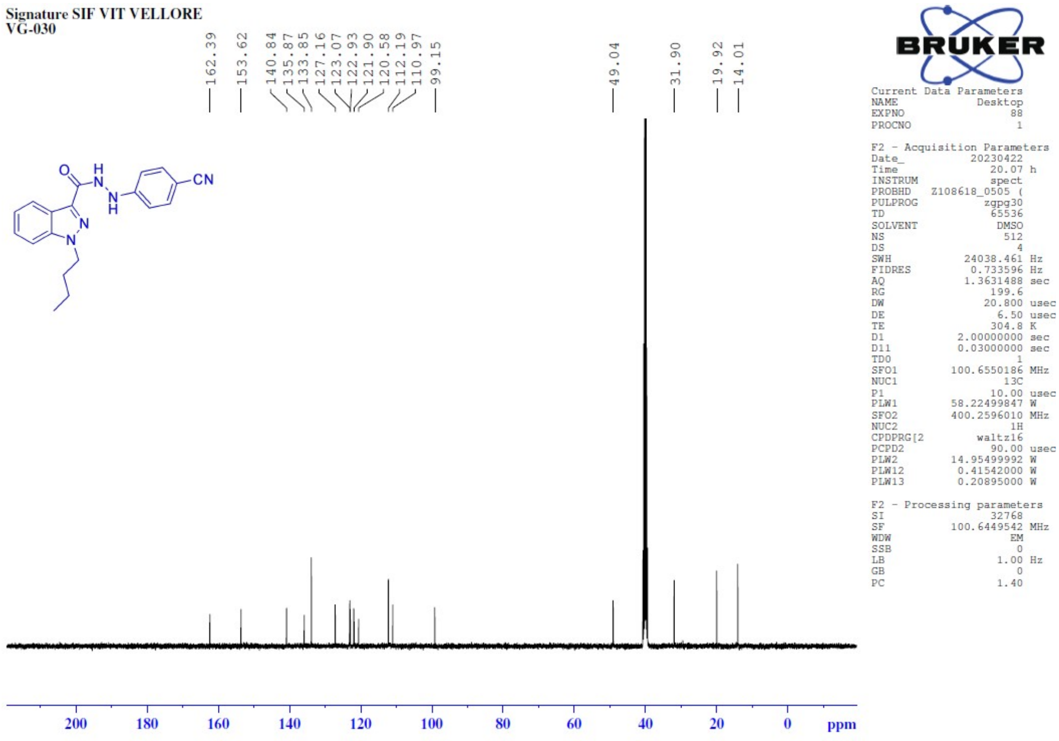

135-DEPT-NMR [100MHz, DMSO-d$_6$] spectrum of 1-butyl-N-(4-cyanophenyl)-1H-indazole-3-carbohydrazide (8W).

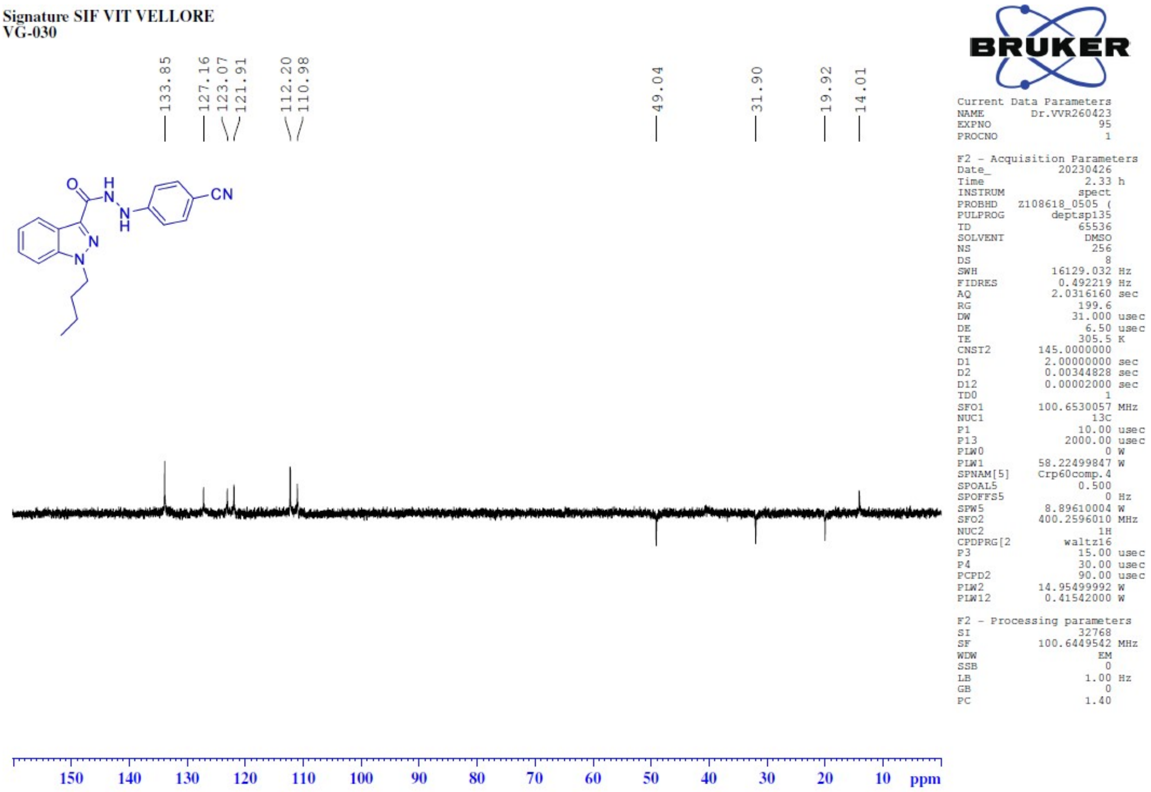

FT-IR spectrum of 1-butyl-N-(4-cyanophenyl)-1H-indazole-3-carbohydrazide (8W).

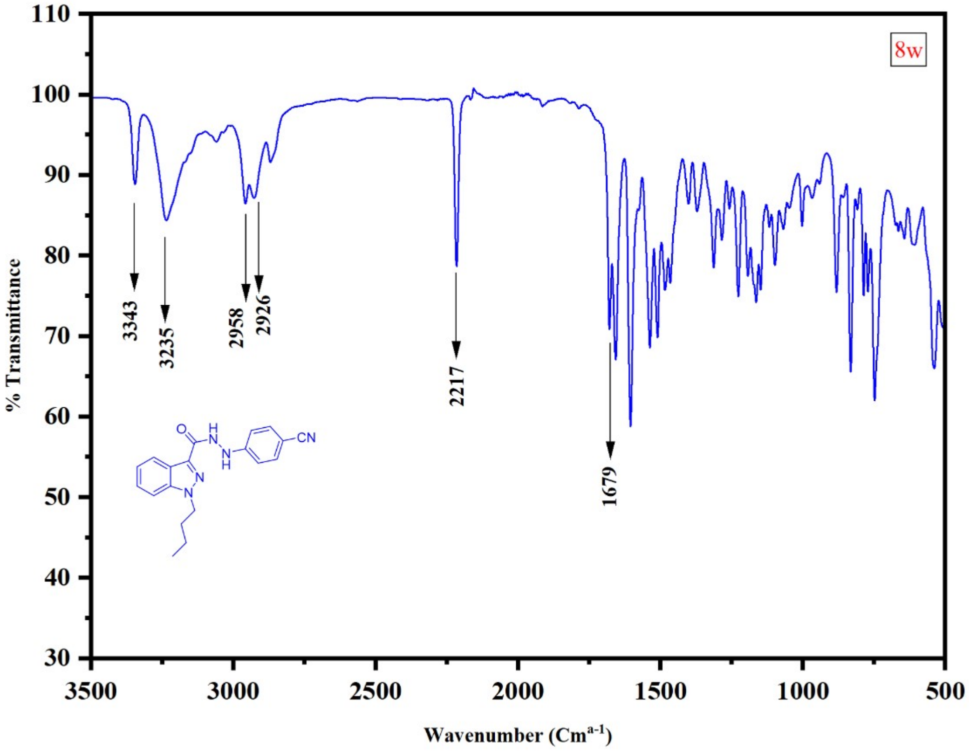

HRMS of 1-butyl-N-(4-cyanophenyl)-1H-indazole-3-carbohydrazide (8W).

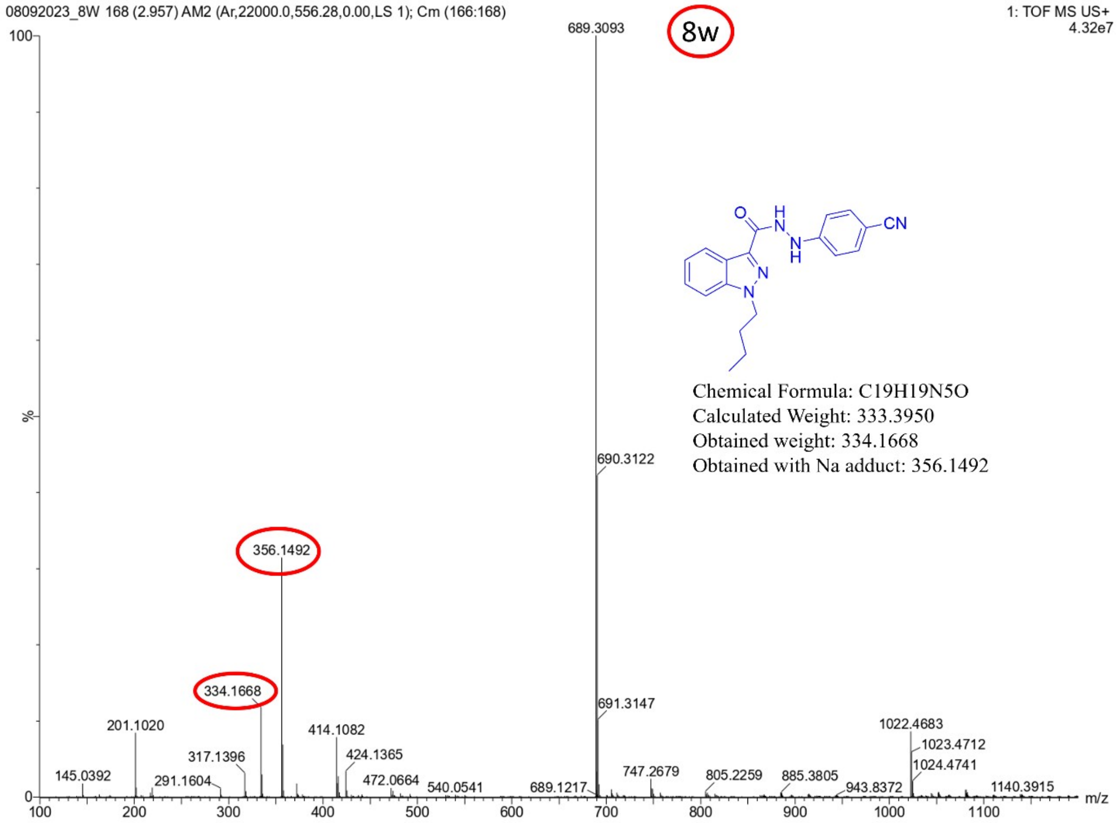

^{1}H-NMR [400MHz, DMSO-d_6] spectrum of 1-butyl-N-(4-hydroxyphenyl)-1H-indazole-3-carbohydrazide (8x).

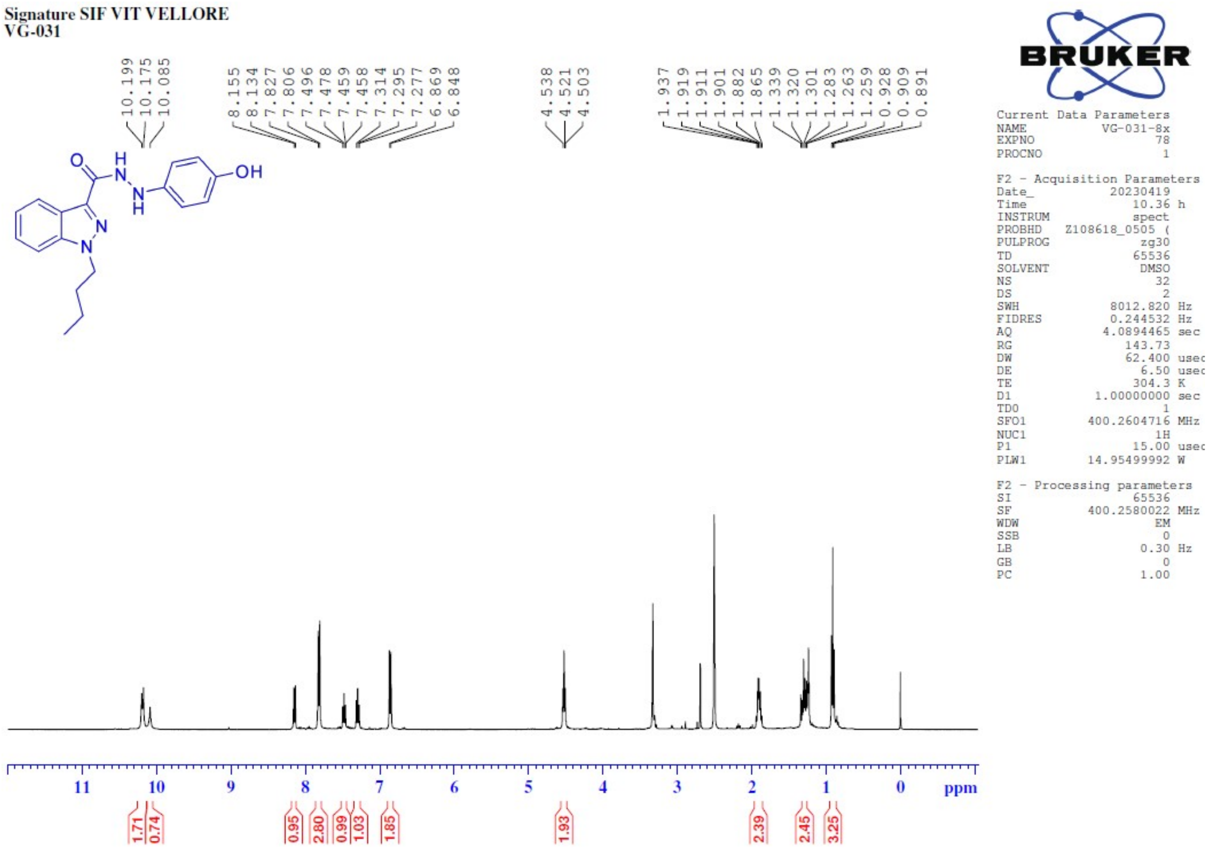

^{13}C-NMR [100MHz, DMSO-d_6] spectrum of 1-butyl-N-(4-hydroxyphenyl)-1H-indazole-3-carbohydrazide (8x).

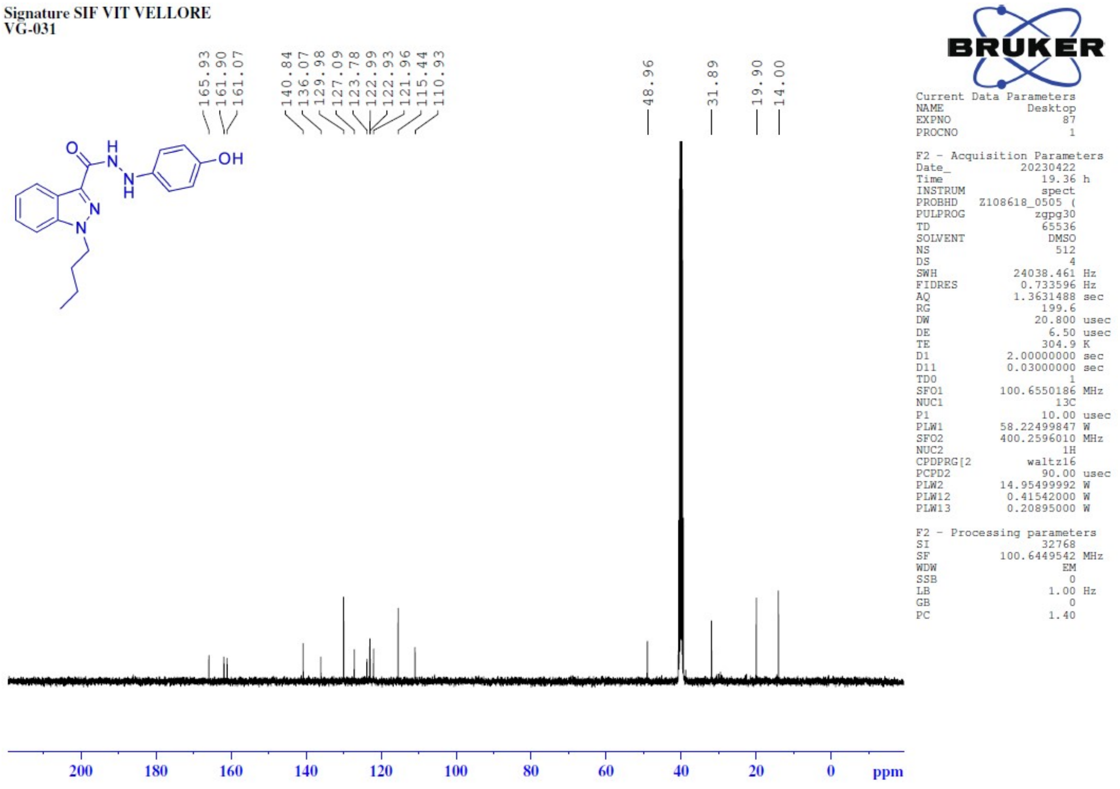

135-DEPT-NMR [100MHz, DMSO-d_6] spectrum of 1-butyl-N-(4-hydroxyphenyl)-1H-indazole-3-carbohydrazide (8x).

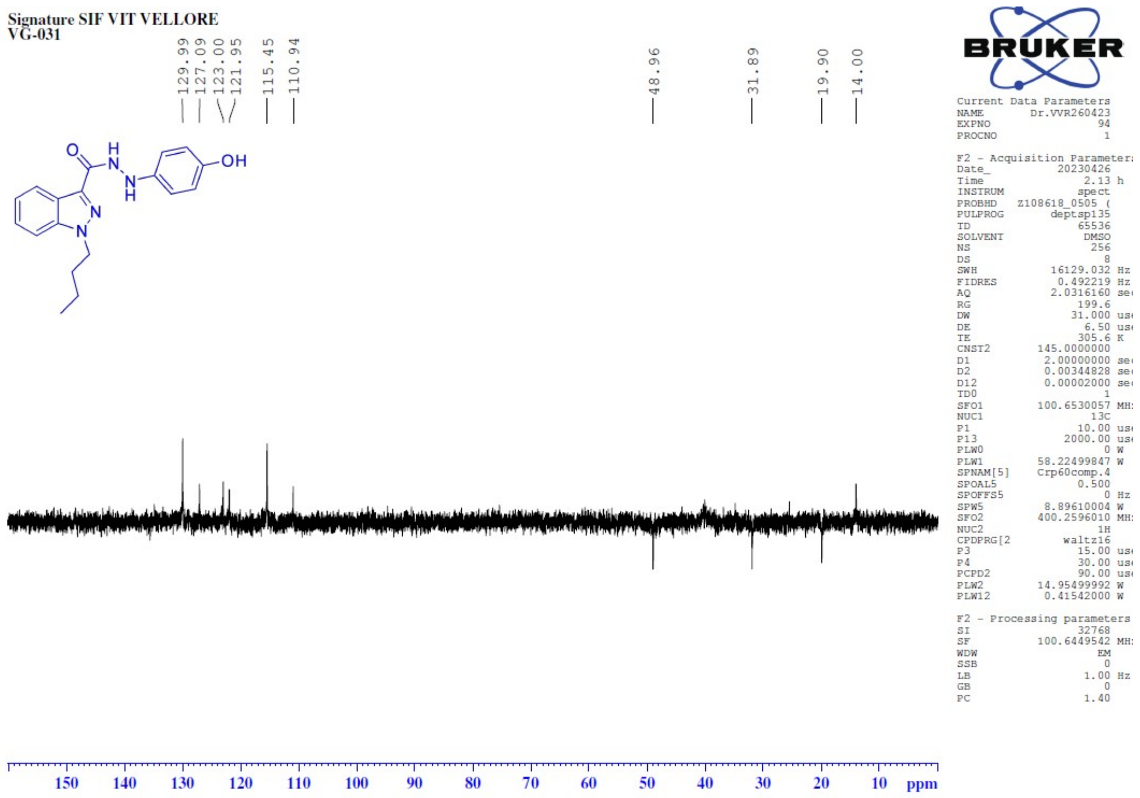

FT-IR spectrum of 1-butyl-N-(4-hydroxyphenyl)-1H-indazole-3-carbohydrazide (8x).

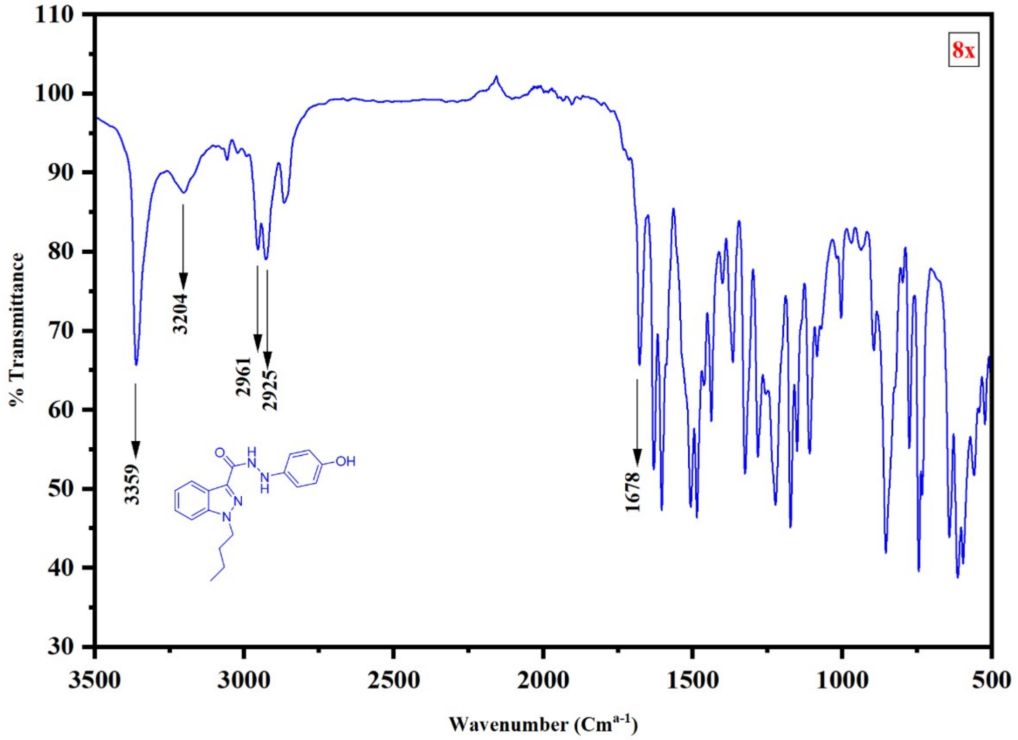

HRMS of 1-butyl-N-(4-hydroxyphenyl)-1H-indazole-3-carbohydrazide (8x).

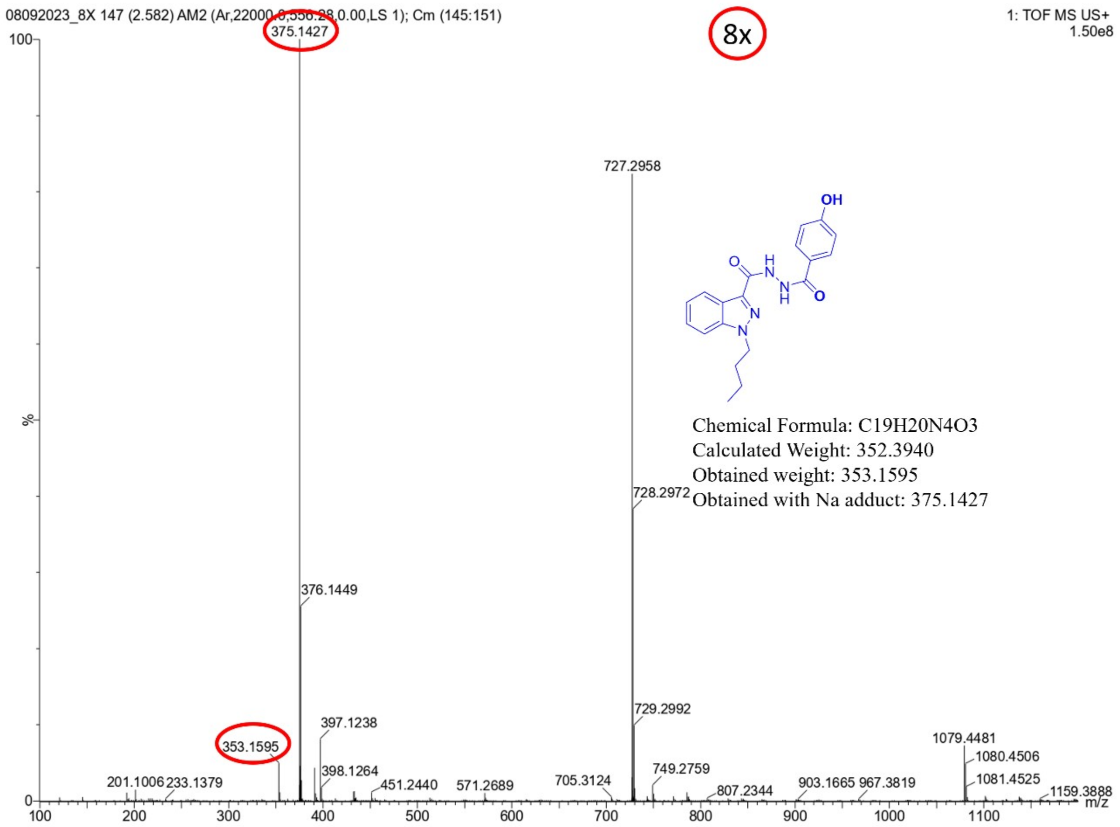

^{1}H-NMR [400MHz, DMSO-d_6] spectrum of N-(4-bromophenyl)1butyl-1H-indazole-3-carbohydrazide (8y).

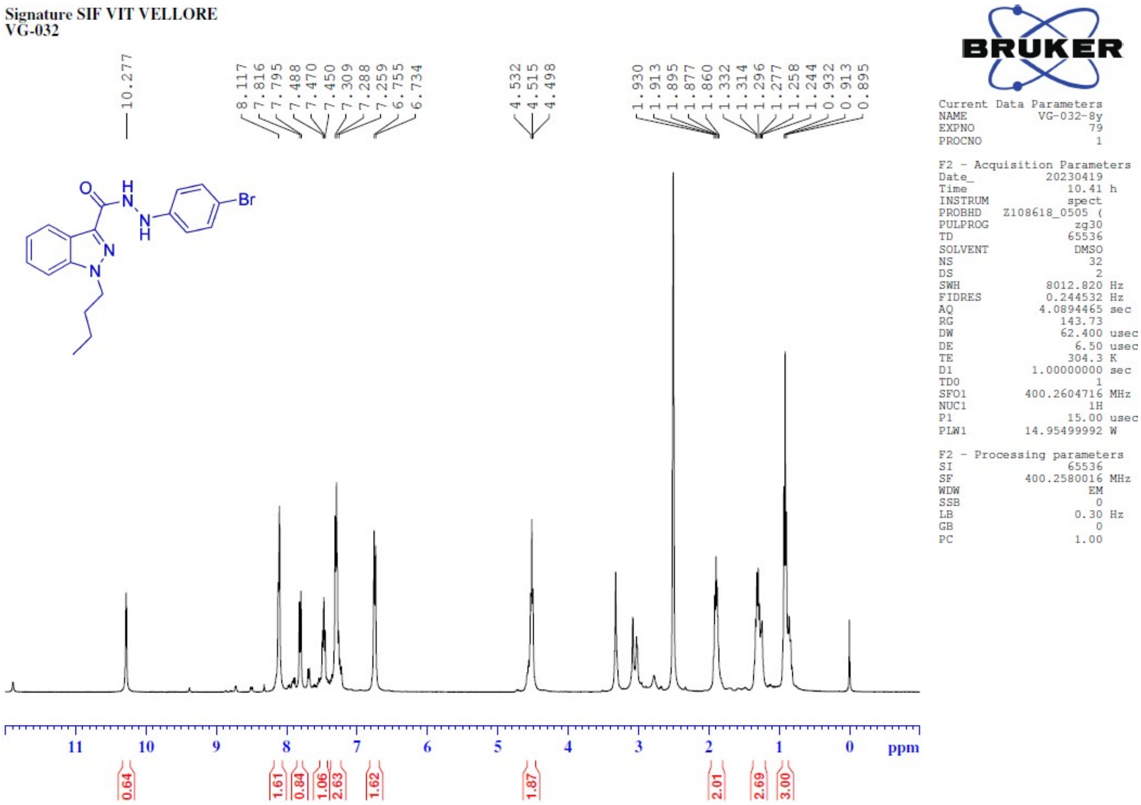

^{13}C-NMR [100MHz, DMSO-d_6] spectrum of N-(4-bromophenyl)1butyl-1H-indazole-3-carbohydrazide (8y).

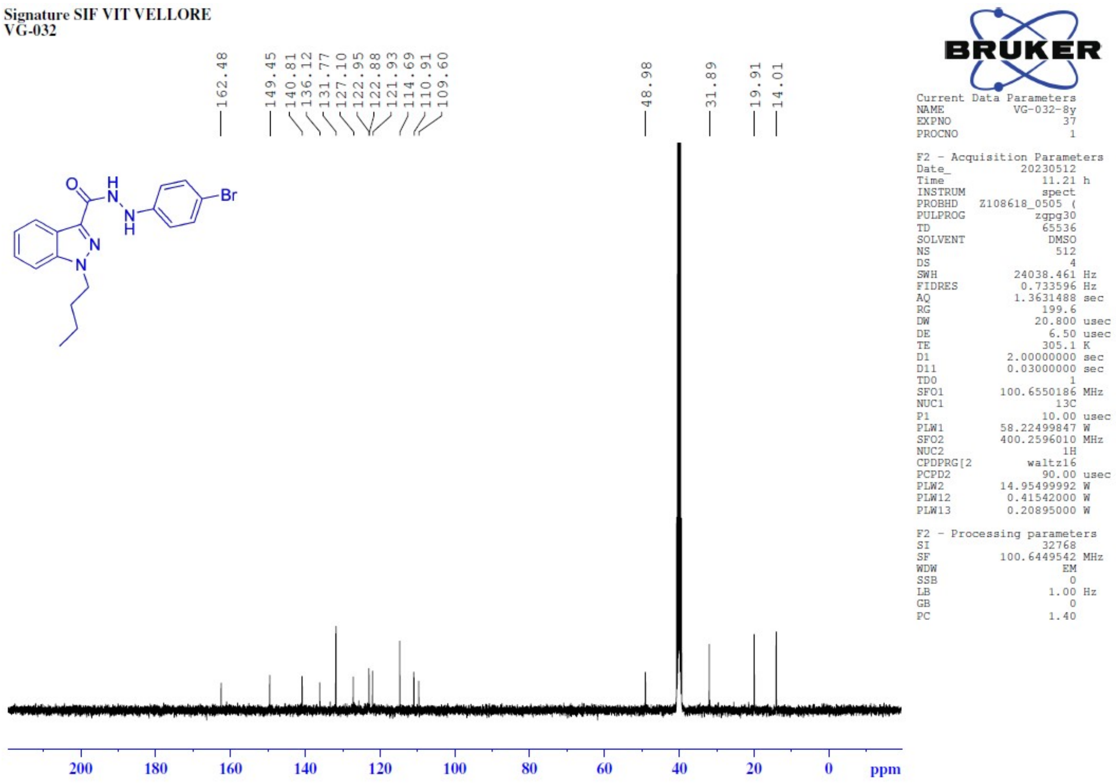

135-DEPT-NMR [100MHz, DMSO-d_6] spectrum of N-(4-bromophenyl)1butyl-1H-indazole-3-carbohydrazide (8y).

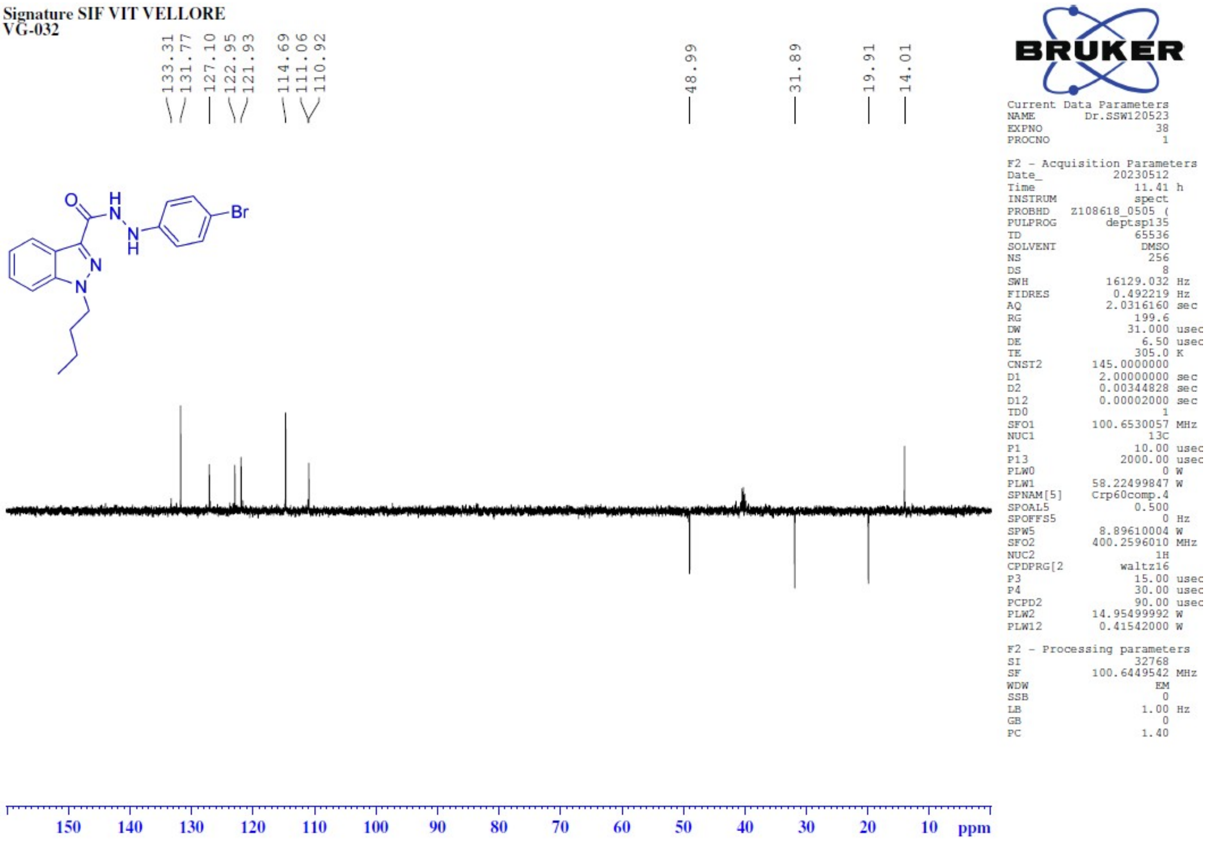

FT-IR spectrum of N-(4-bromophenyl)1butyl-1H-indazole-3-carbohydrazide (8y).

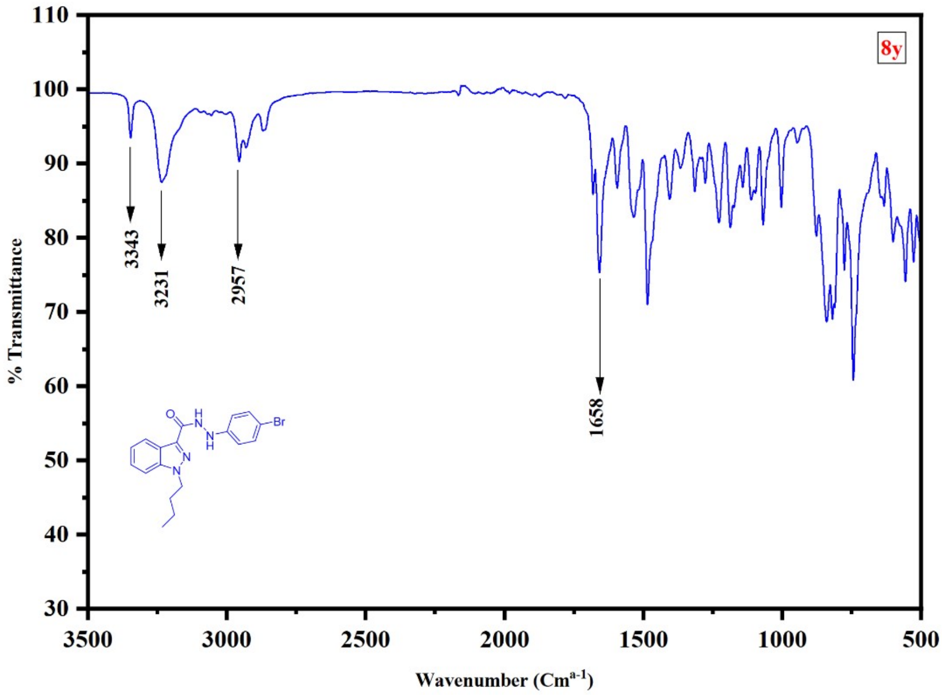

HRMS of N-(4-bromophenyl)1butyl-1H-indazole-3-carbohydrazide (8y).

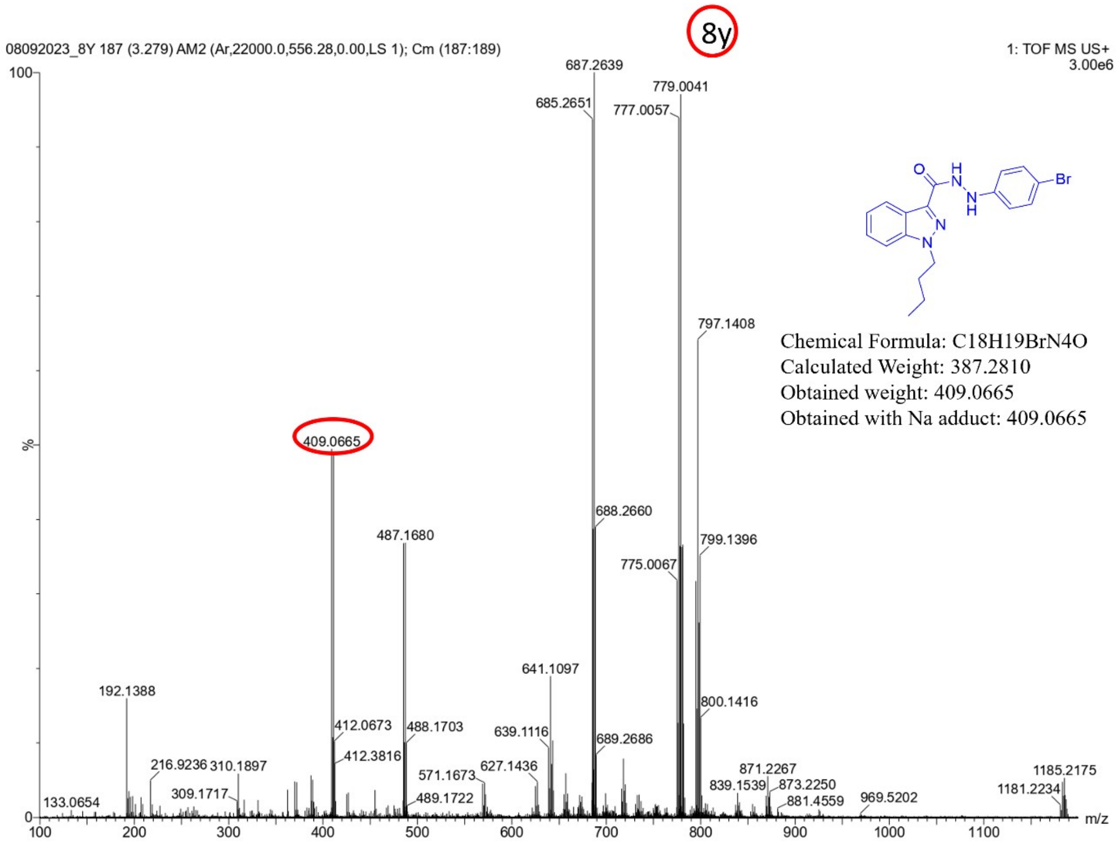

^{1}H-NMR [400MHz, DMSO-d_6] 1-Butyl-1H-indazole-3-carboxylic acid to 1-butyl-N-(4-nitrophenyl)-1H-indazole-3-carboxamide (8z).

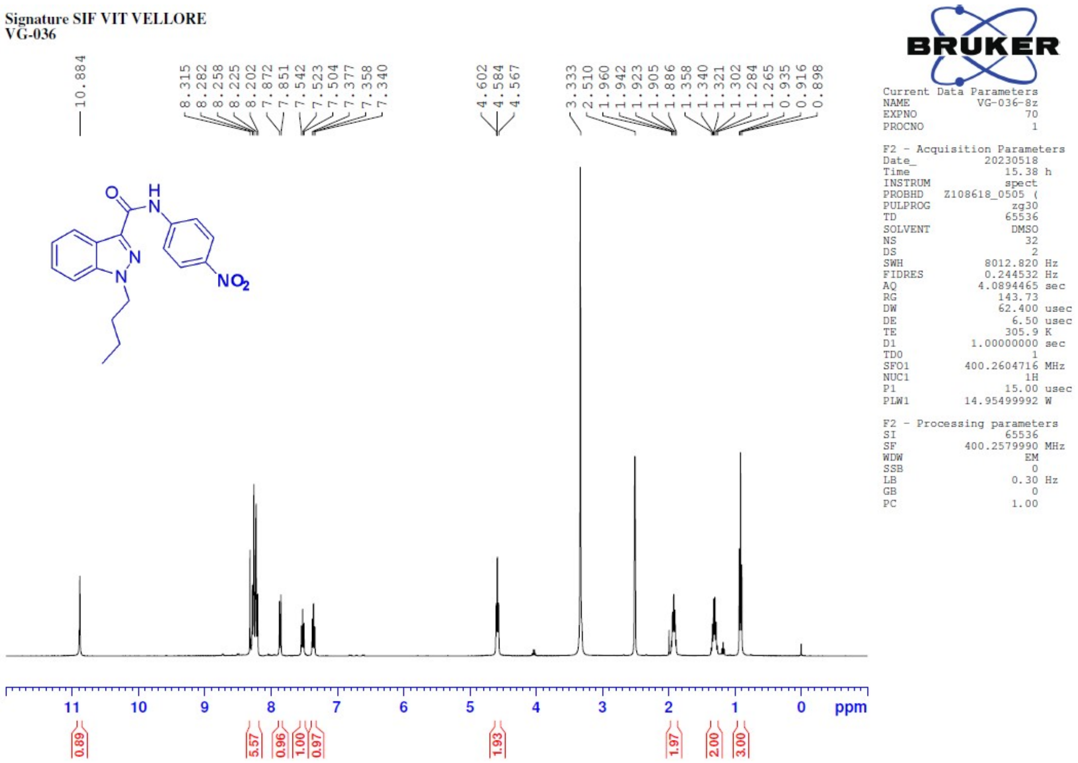

^{13}C-NMR [100MHz, DMSO-d_6] 1-Butyl-1H-indazole-3-carboxylic acid to 1-butyl-N-(4-nitrophenyl)-1H-indazole-3-carboxamide (8z).

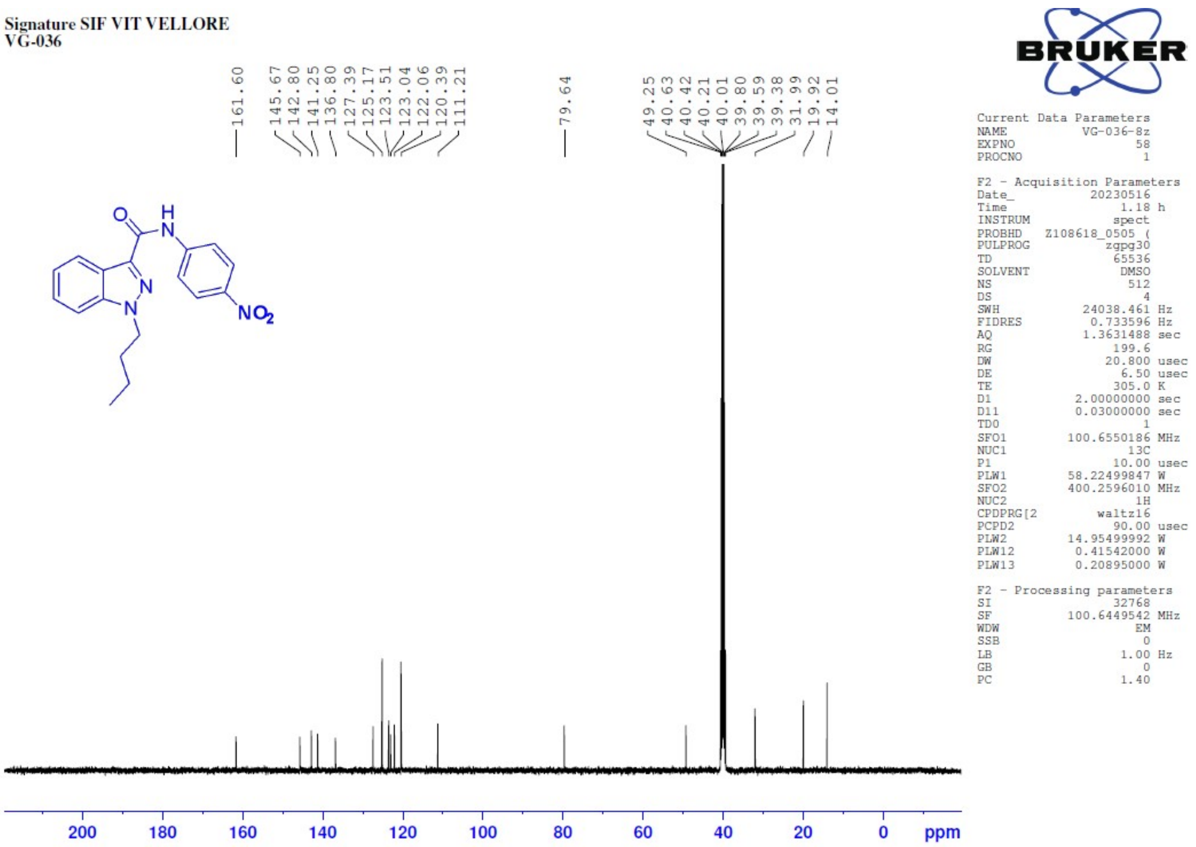

^{135}C-NMR [100MHz, DMSO-d_6] 1-Butyl-1H-indazole-3-carboxylic acid to 1-butyl-N-(4-nitrophenyl)-1H-indazole-3-carboxamide (8z).

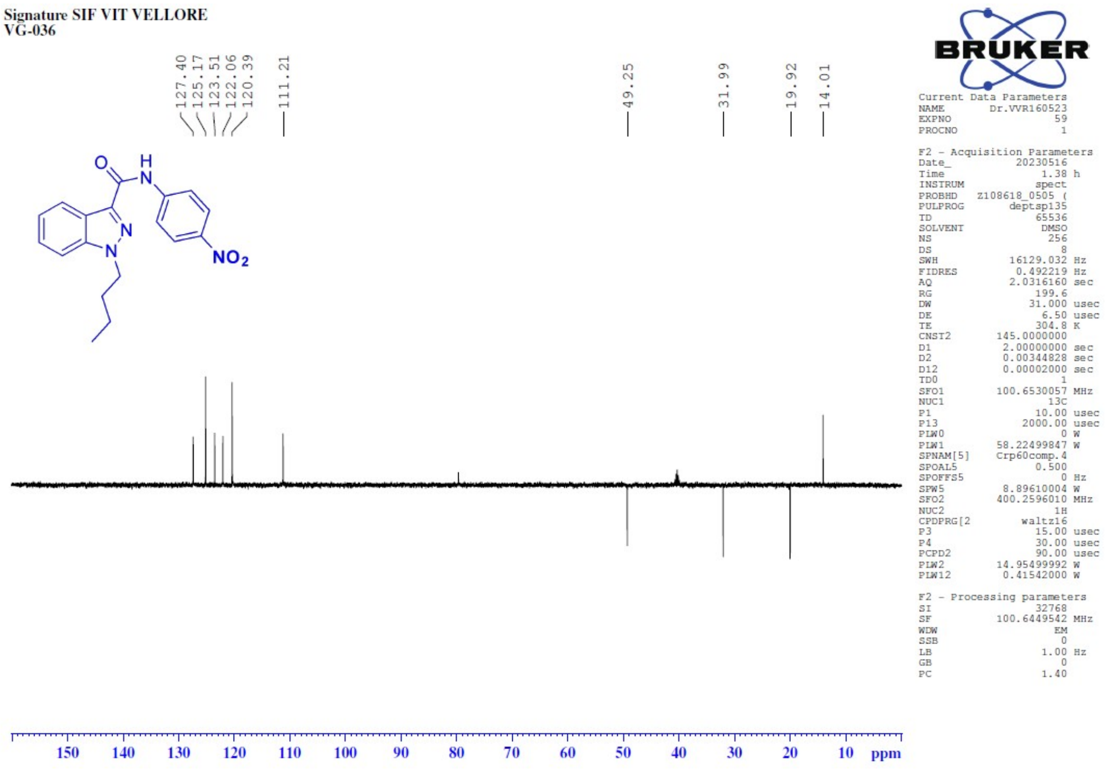

FT-IR spectrum of 1-Butyl-1H-indazole-3-carboxylic acid to 1-butyl-N-(4-nitrophenyl)-1H-indazole-3-carboxamide (8z).

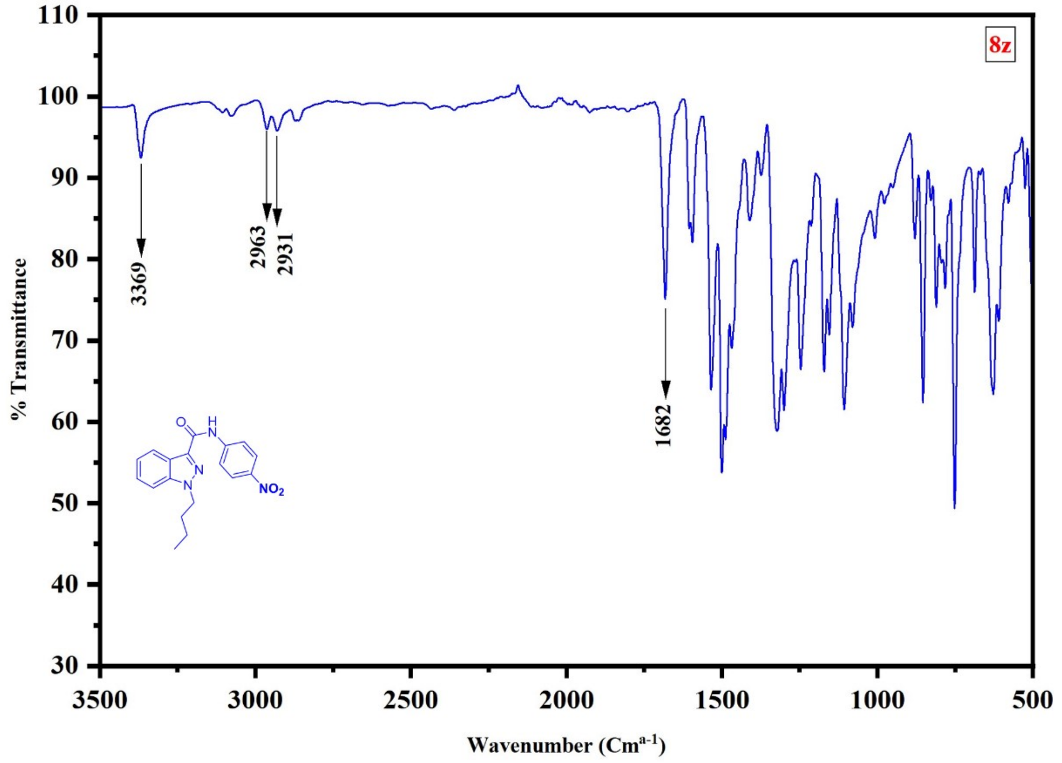

HRMS of 1-butyl-N-(4-nitrophenyl)-1H-indazole-3-carboxamide (8z).

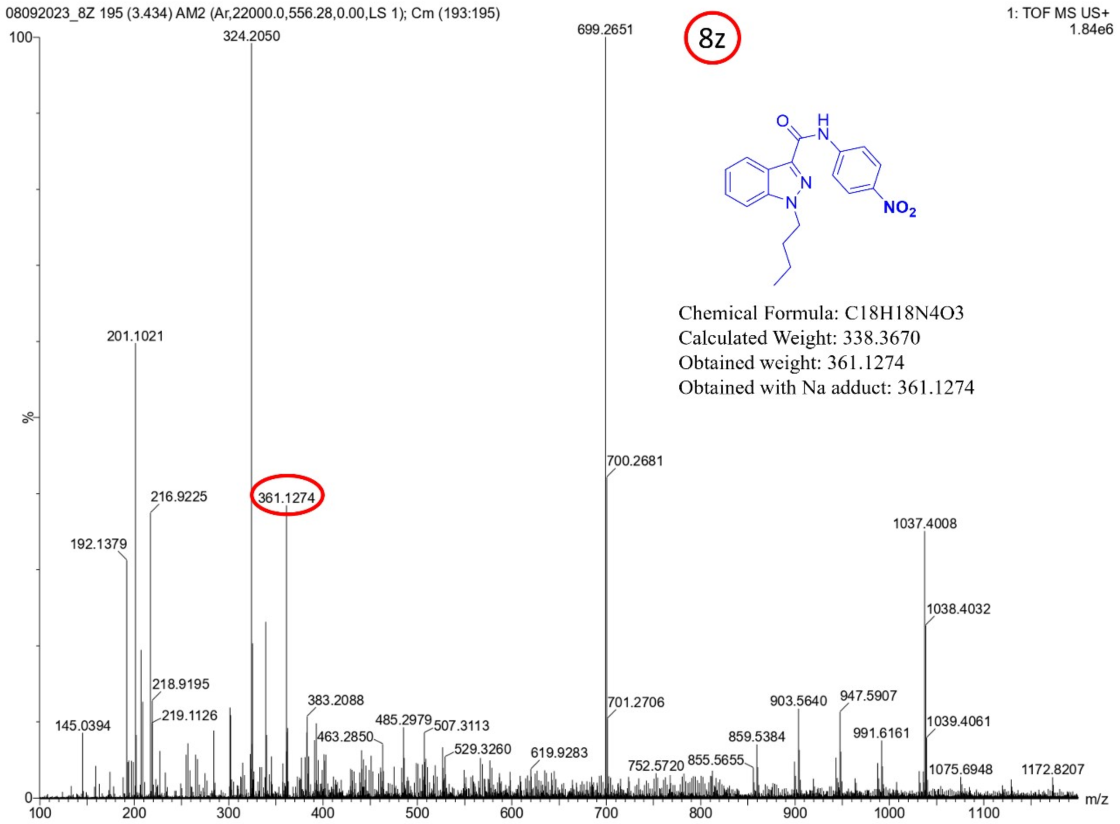

<u>**5.DFT studies indazole derivatives with HOMO and LUMO values**</u>

S. No:	COMPOUND	HOMO	LUMO	ΔE = E_{LUMO}-E_{HOMO}
1	8a	-6.0453	-0.86238	**5.18292**
2	8b	-5.51394	-1.07163	4.44231
3	8c	-5.88654	-0.81999	**5.06655**
4	8d	-6.12522	-2.65032	3.4749
5	8e	-5.7888	-1.17666	4.61214
6	8f	-4.44879	-0.98955	3.45924
7	8g	-5.89761	-1.40211	4.4955
8	8h	-5.65434	-2.31201	3.34233
9	8i	-5.62275	-1.21878	4.40397
10	8j	-5.14566	-1.04112	4.10454

11	8k	-5.23692	-0.92853	4.30839
12	8l	-5.44563	-1.05111	4.39452
13	8m	-5.41485	-1.07244	4.34241
14	8n	-5.67216	-1.00143	4.67073
15	8o	-5.11137	-1.0287	4.08267
16	8p	-5.5039	-1.15128	4.35262
17	8q	-5.21937	-0.92529	4.29408
18	8r	-5.47317	-1.14426	4.32891
19	8s	-6.34878	-1.4283	**4.92048**
20	8t	-5.27715	-2.60523	2.67192
21	8u	-6.8823	-2.45457	4.42773
22	8v	-6.36687	-3.59964	2.76723
23	8w	-5.08923	-1.32435	3.76488
24	8x	-6.67332	-2.42568	4.24764
25	8y	-4.24305	-1.00143	3.24162
26	8z	-6.4638	-2.77776	6.68604

DFT studies indazole derivatives HOMO &LUMO (8a-8z)

8a

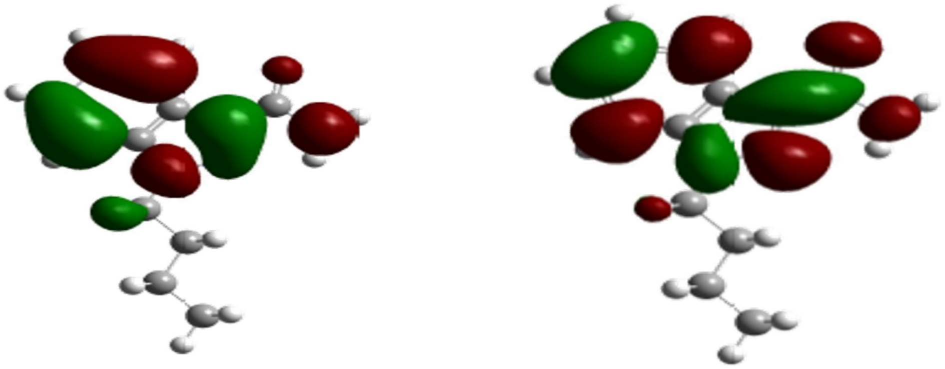

HOMO LUMO

8b

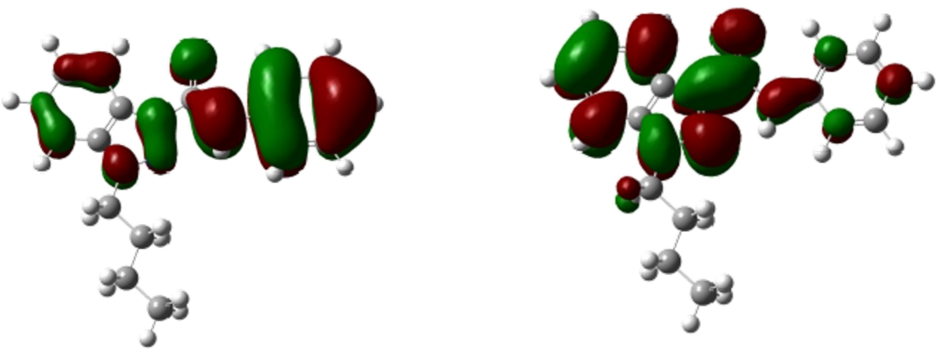

HOMO LUMO

8c

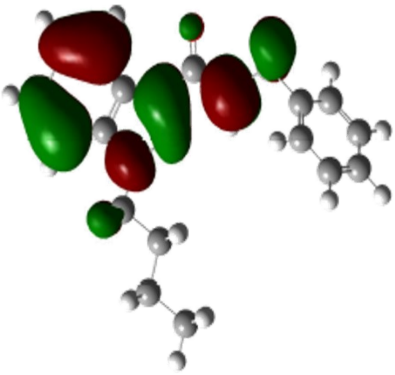

HOMO

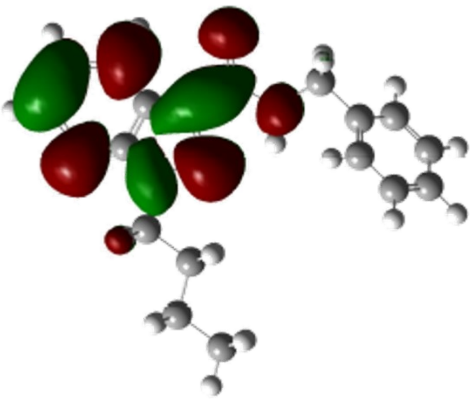

LUMO

8d

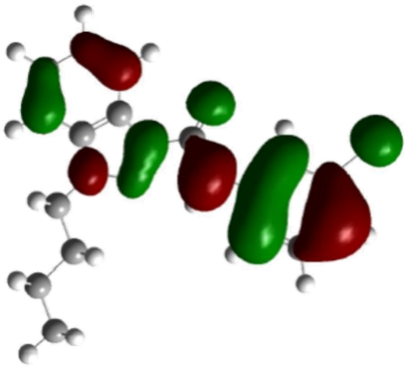

HOMO

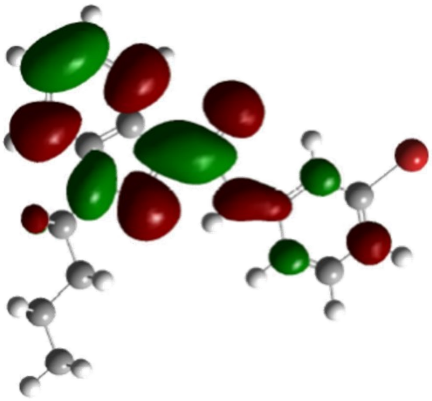

LUMO

8e

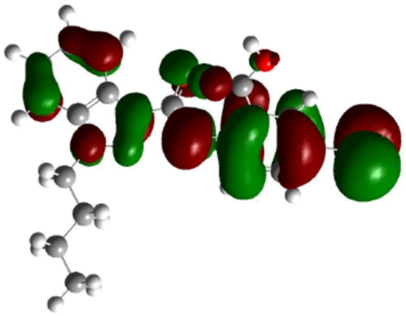

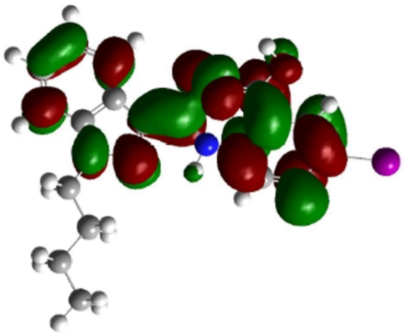

HOMO LUMO

8f

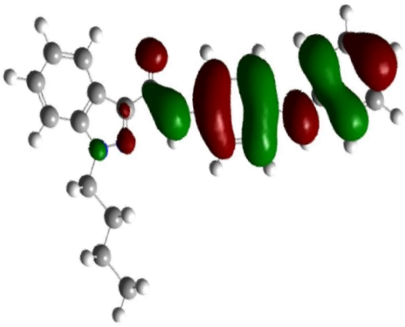

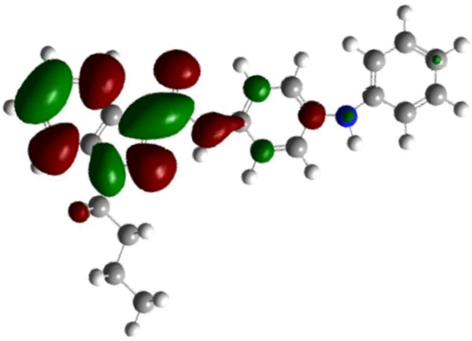

HOMO LUMO

8g

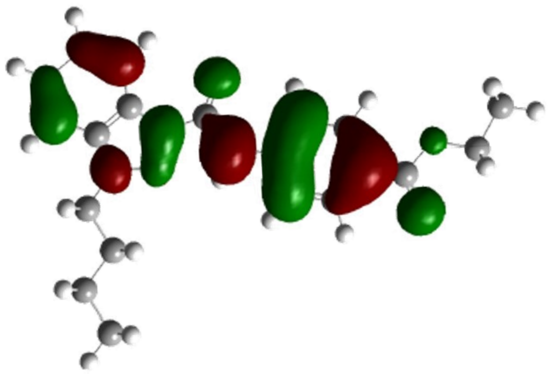

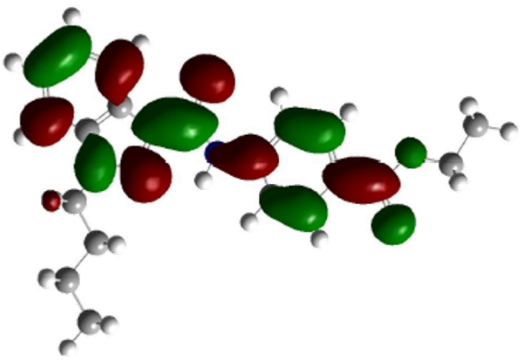

HOMO LUMO

8h

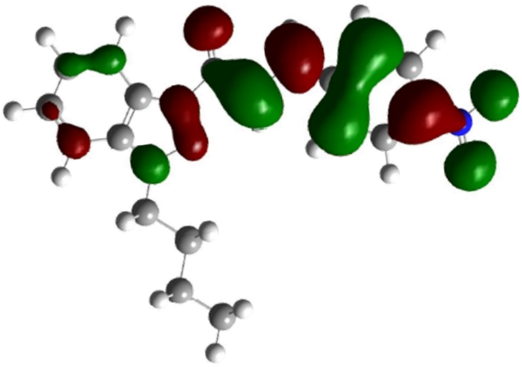

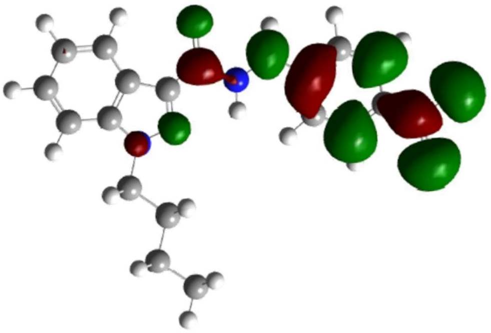

HOMO LUMO

8i

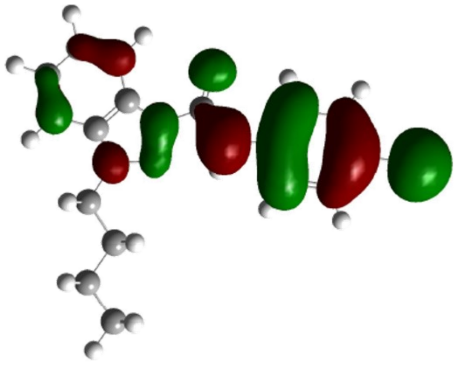

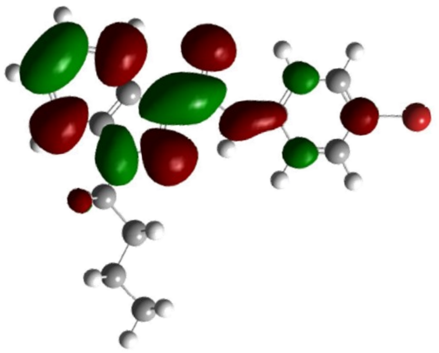

HOMO LUMO

8j

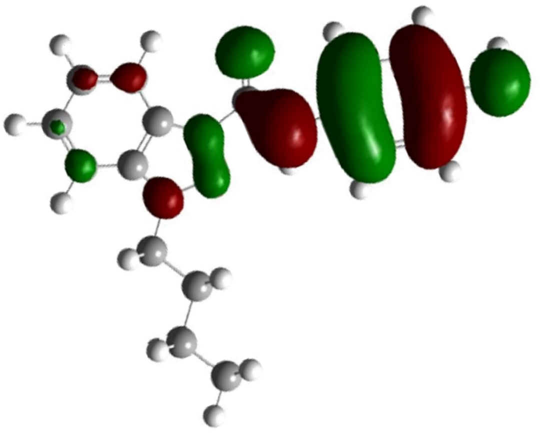

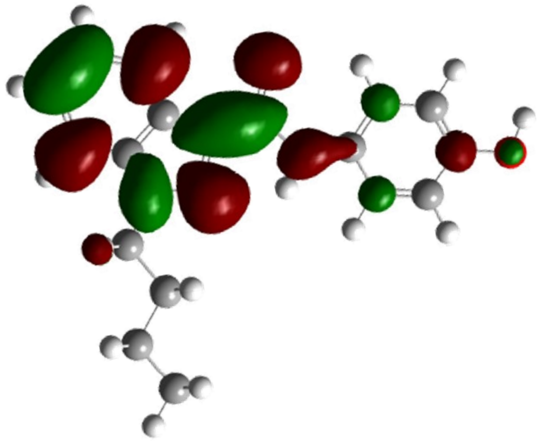

HOMO LUMO

8k

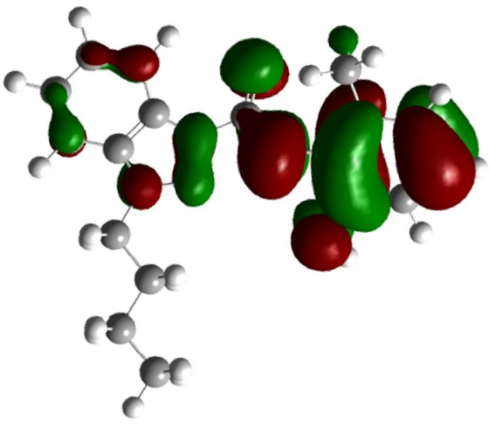

HOMO

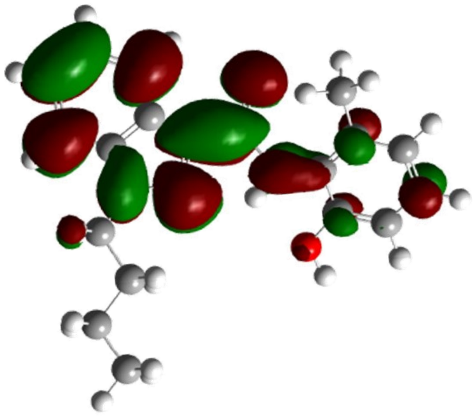

LUMO

8l

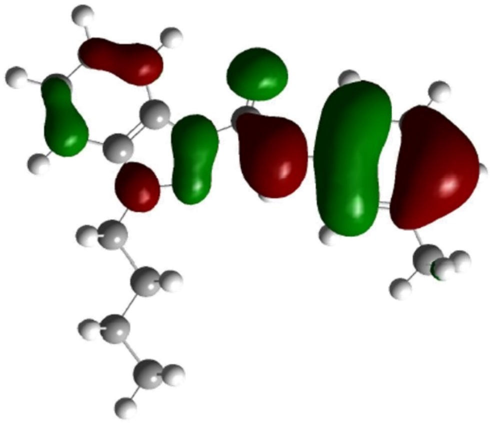

HOMO

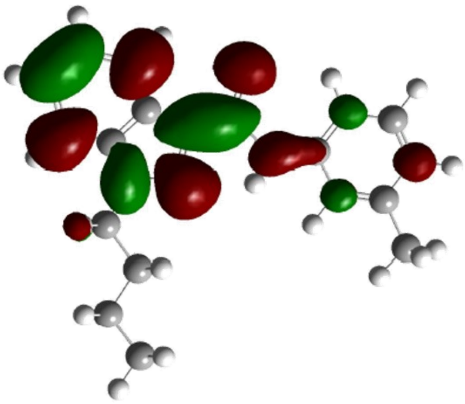

LUMO

8m

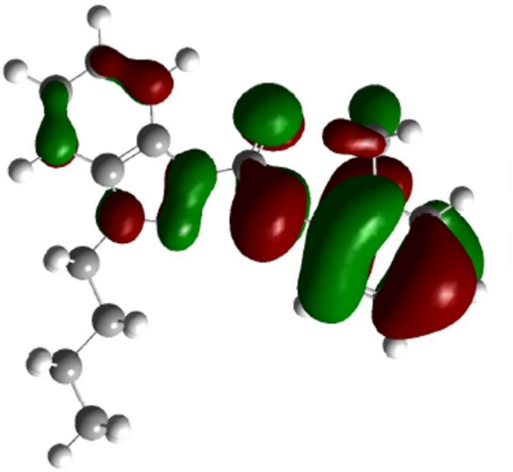

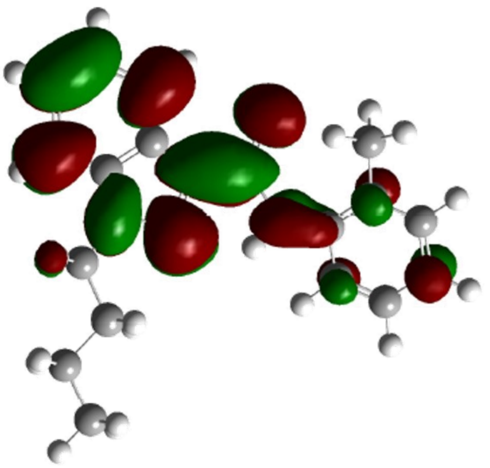

HOMO LUMO

8n

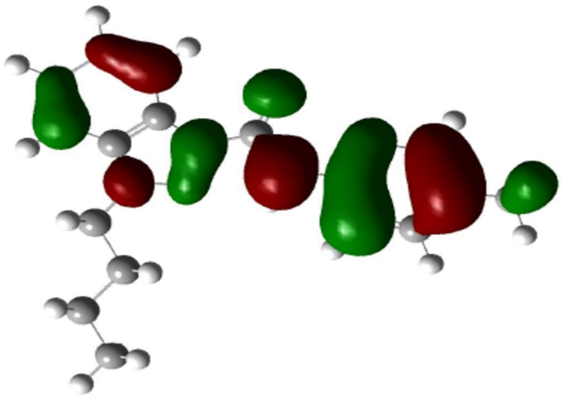

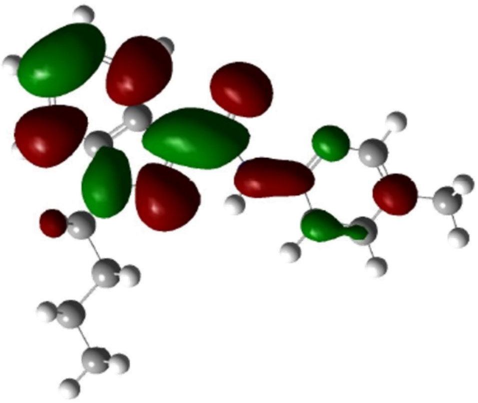

HOMO

8o

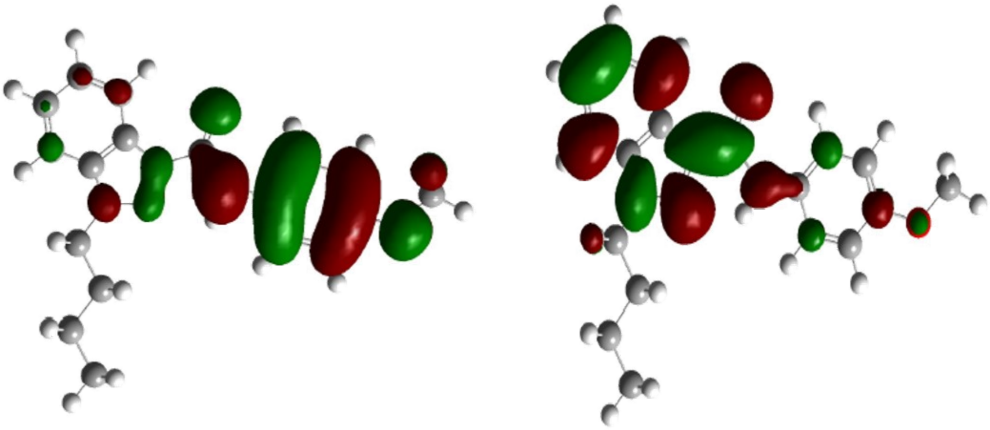

HOMO LUMO

8p

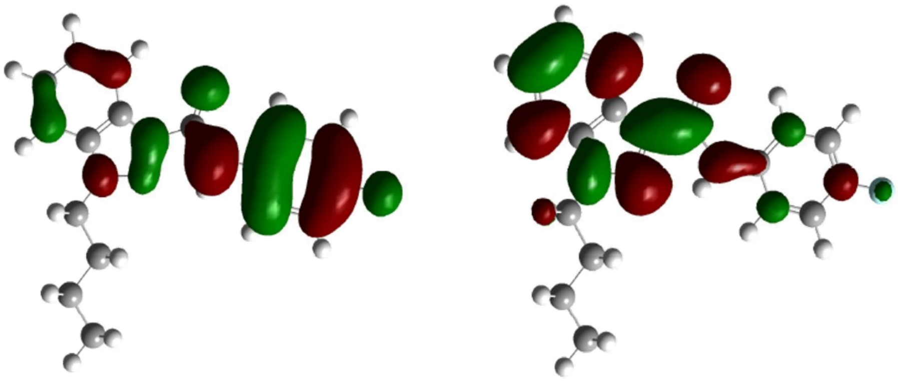

HOMO LUMO

8q

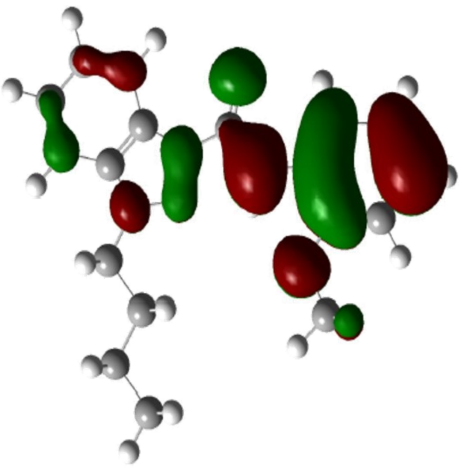

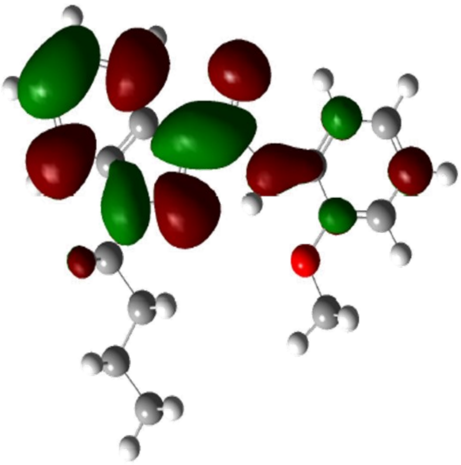

HOMO LUMO

8r

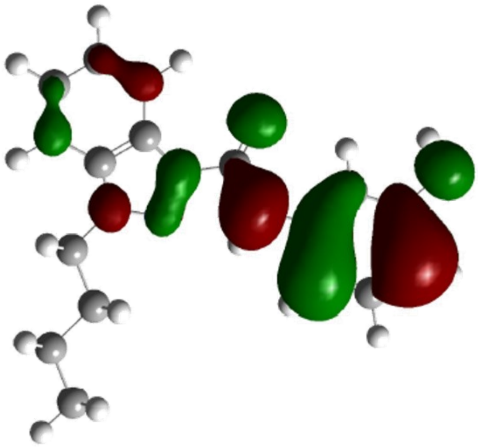

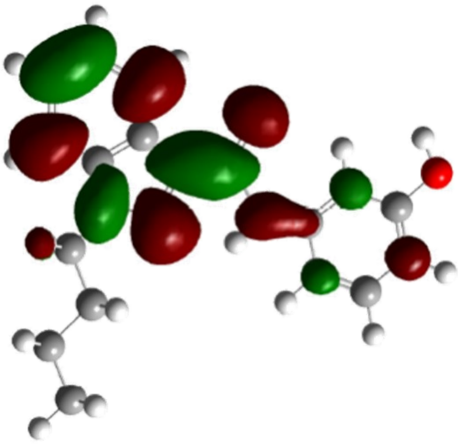

HOMO LUMO

8s

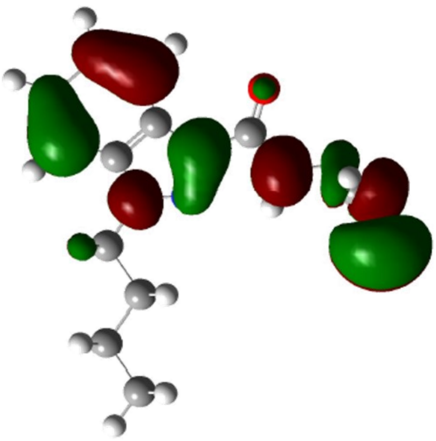

HOMO

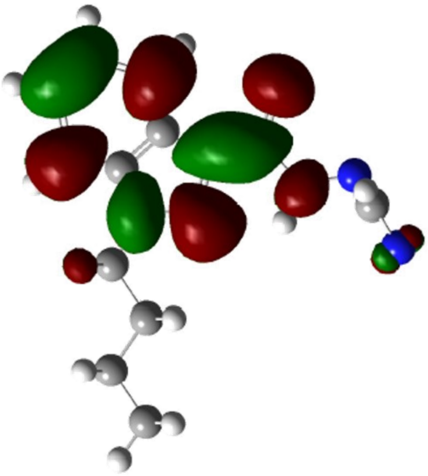

LUMO

8t

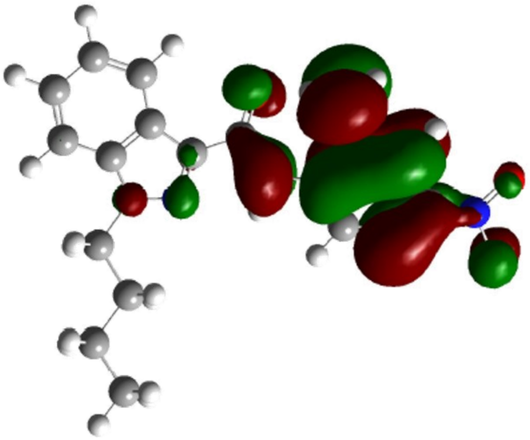

HOMO

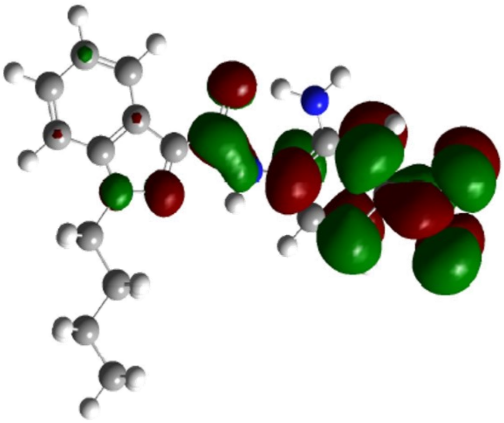

LUMO

8u

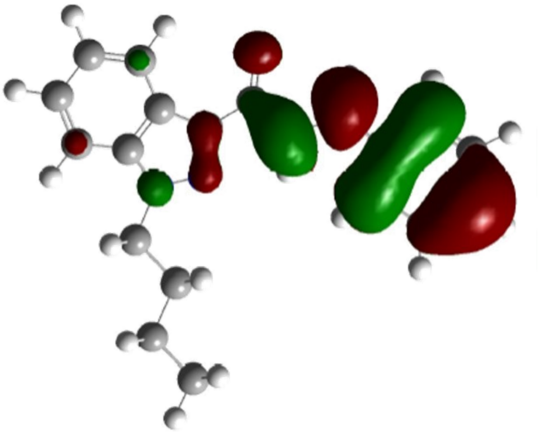

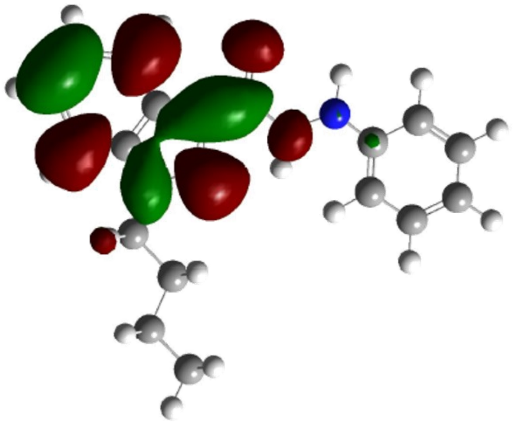

HOMO LUMO

8v

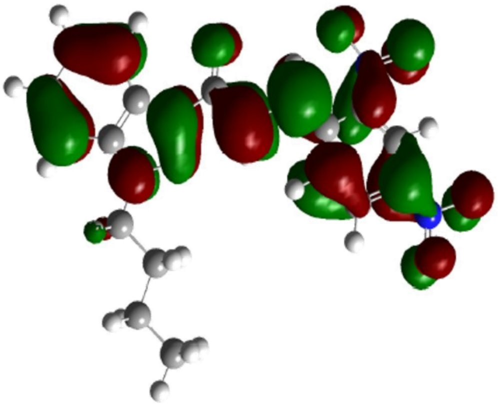

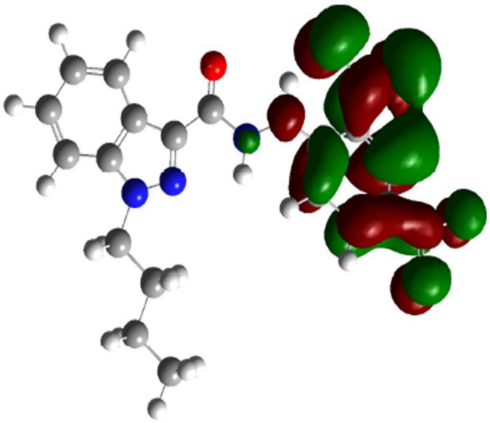

HOMO LUMO

8w

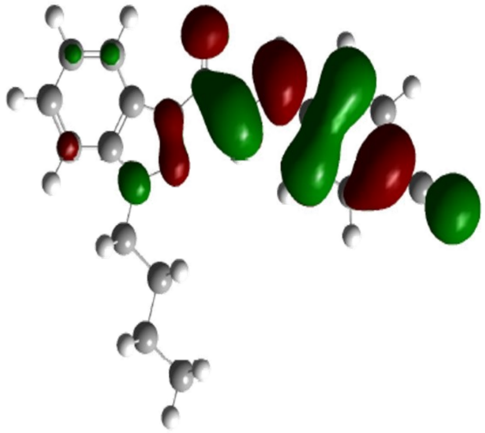

HOMO

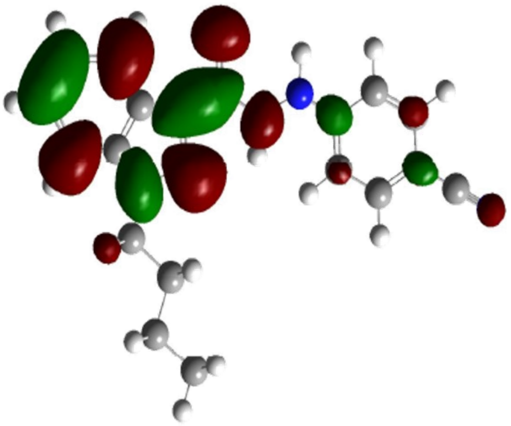

LUMO

8x

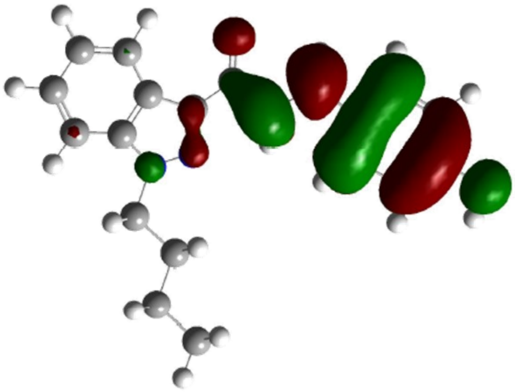

HOMO

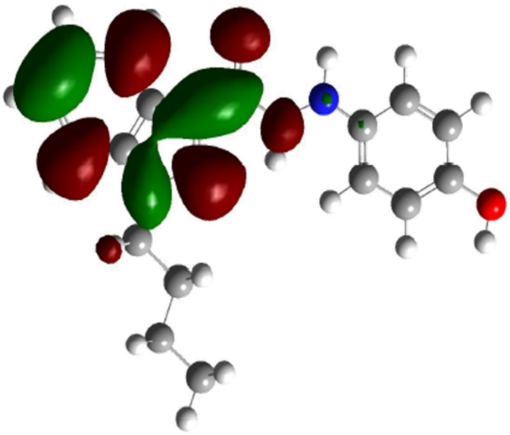

LUMO

8y

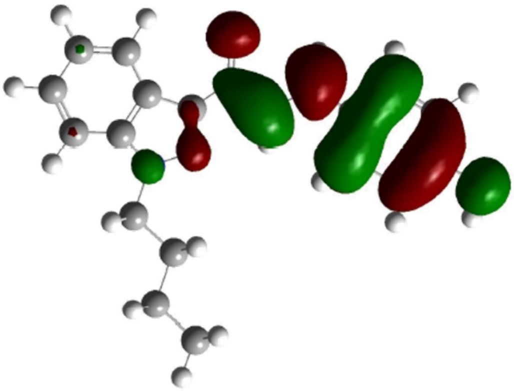

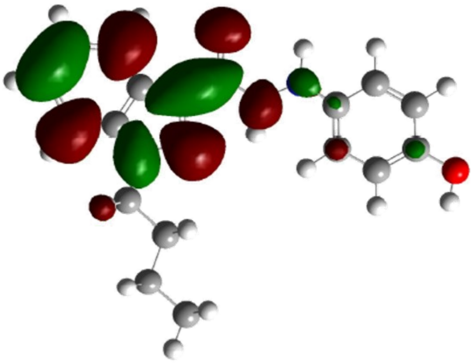

HOMO LUMO

8z

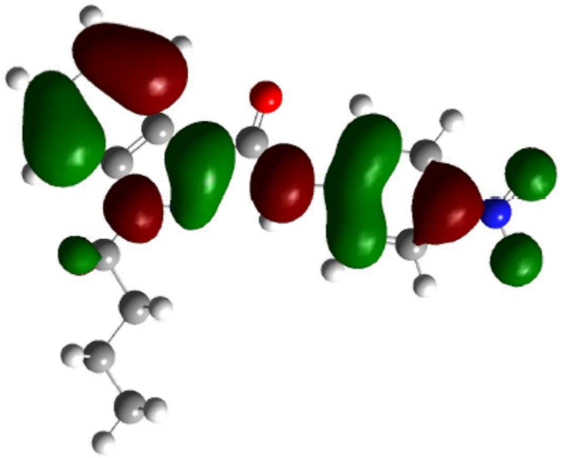

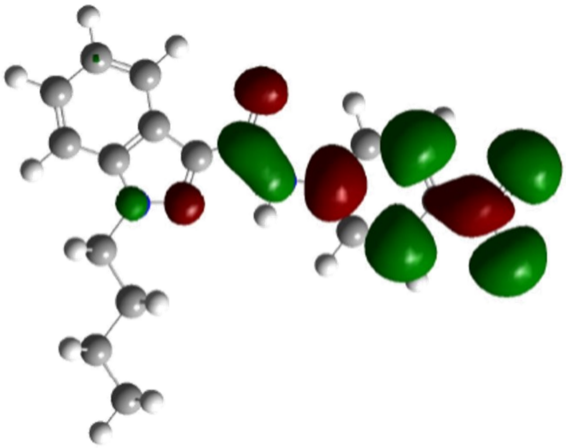

HOMO LUMO

<u>Molecular Electrostatic Potential surface of synthesized indazole derivatives</u>

8a

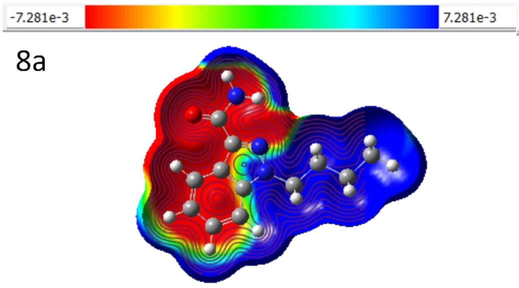

8b

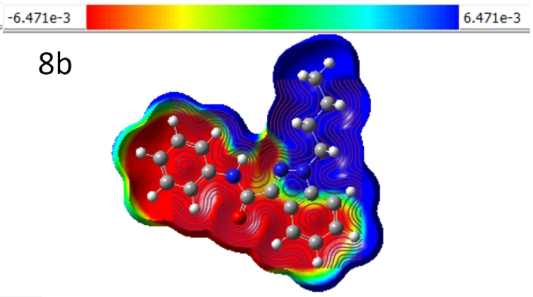

8c

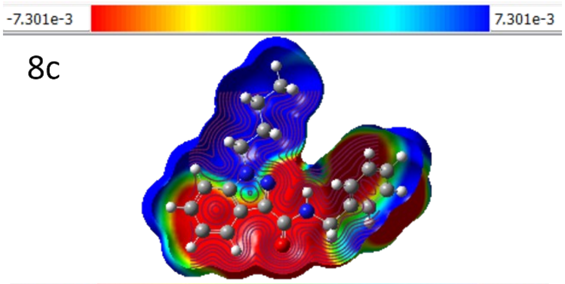

8d

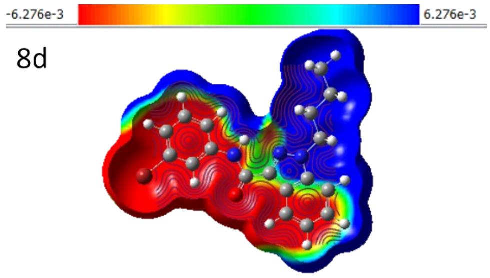

8e

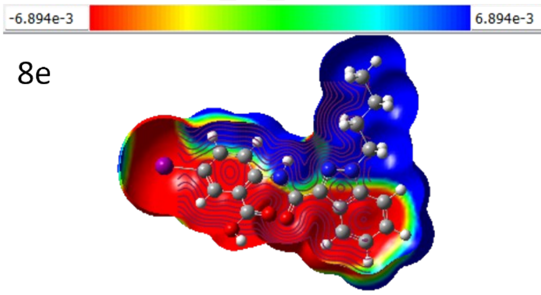

8f

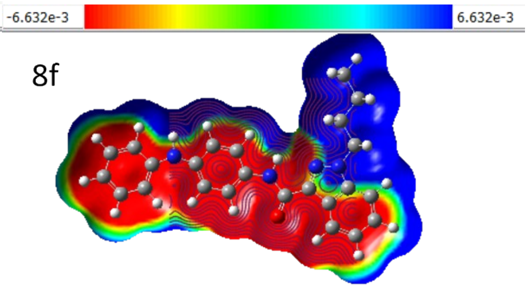

8g

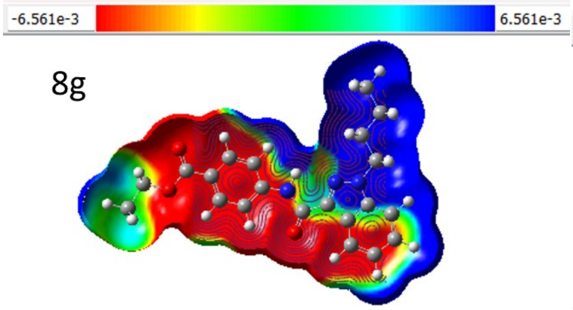

8h

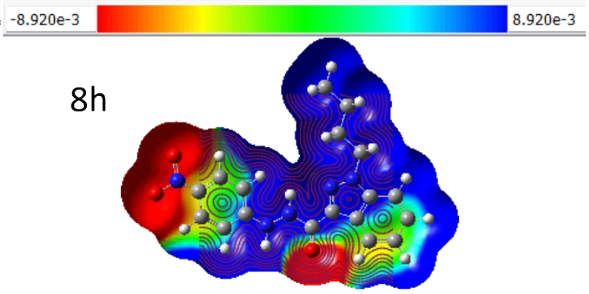

8i

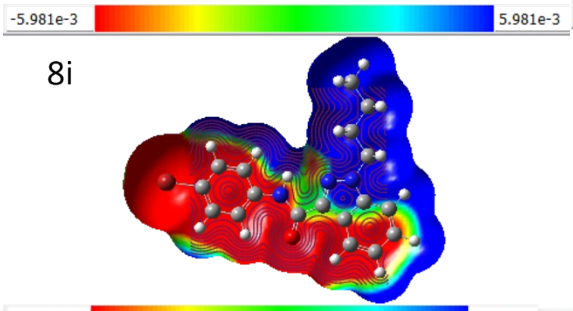

8j

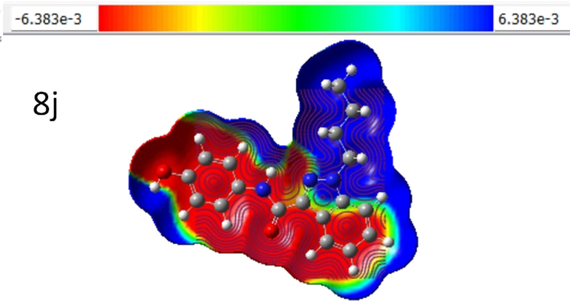

8k

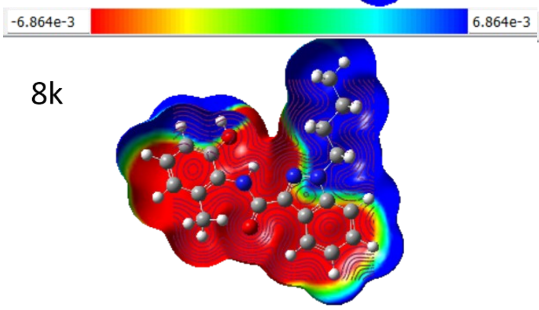

8l

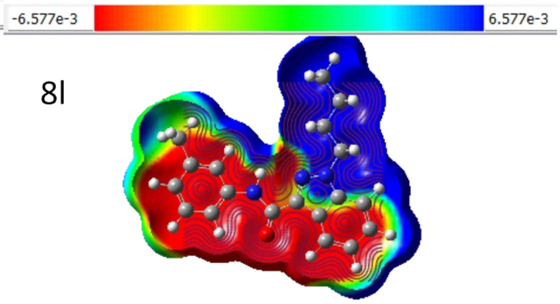

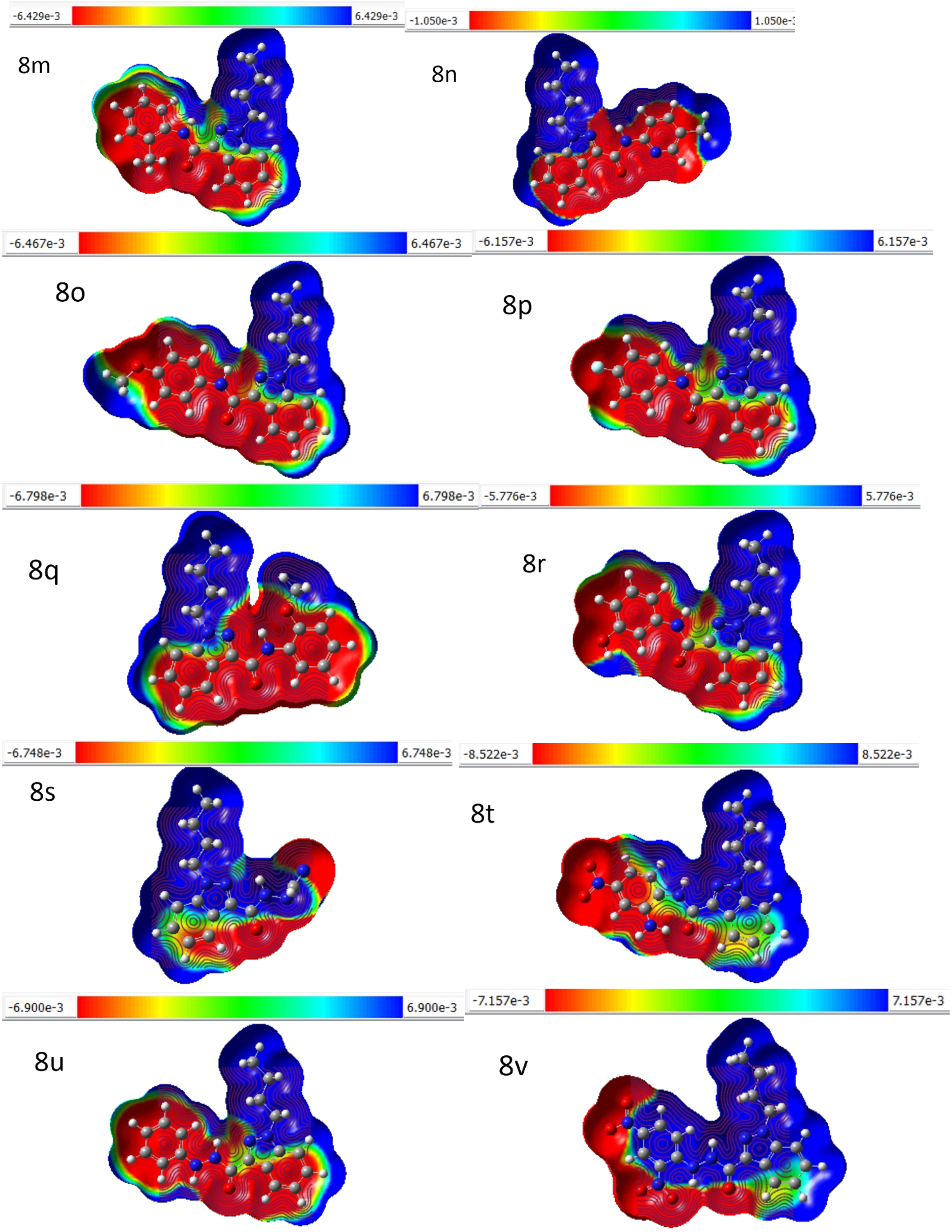
8m
-6.429e-3
6.429e-3
8n
-1.050e-3
1.050e-
8o
-6.467e-3
6.467e-3
8p
-6.157e-3
6.157e-3
8q
-6.798e-3
6.798e-3
8r
-5.776e-3
5.776e-3
8s
-6.748e-3
6.748e-3
8t
-8.522e-3
8.522e-3
8u
-6.900e-3
6.900e-3
8v
-7.157e-3
7.157e-3

8w

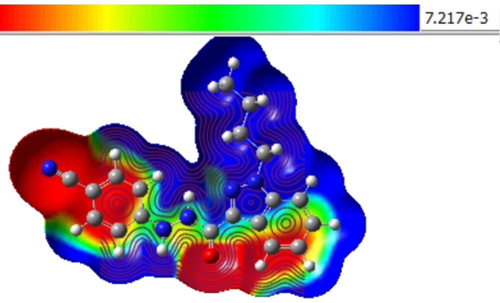

8x

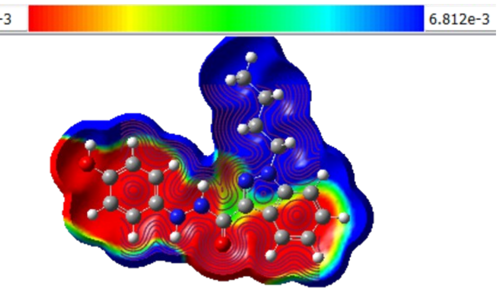

8y

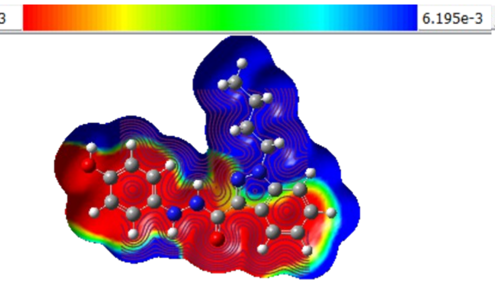

8z

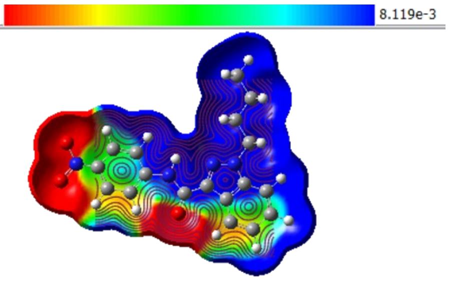

Docking results from (8a-8z) 3-carboxamide indazole derivatives

S.no/Compound	$\Delta G_{binding\ energy}$ (Kcal/mol)	Ki (micromolar) [Temperature = 298.15 k] need to change nanomolar to micro molar	H bond energy (Kcal/mol)
8a	-7.03	7.00	-8.24
8b	-9.38	133.69	-10.84
8c	-10.51	19.74	-12.19
8d	-9.51	106.73	-10.67
8e	-10.23	31.66	-11.48
8f	-10.59	17.28	-12.66
8g	-10.21	32.61	-12.50
8h	-10.56	18.24	-12.48
8i	-10.47	21.07	-11.93
8j	-9.12	207.41	-10.84
8k	-9.94	51.93	-11.70
8l	-9.77	69.21	-11.24
8m	-10.18	34.63	-11.59
8n	-9.55	100.18	-11.00
8o	-9.94	51.34	-11.62
8p	-9.71	76.64	-11.15
8q	-10.22	32.11	-11.92
8r	-9.83	61.84	-11.56
8s	-8.36	729.01	-9.81
8t	-10.72	13.89	-12.63
8u	-10.38	24.47	-12.09
8v	-11.77	2.35	-13.47

8w	-11.64	2.94	-13.33
8x	-10.32	27.09	-12.31
8y	-11.52	3.61	-13.21
8z	-10.81	11.9	-12.40

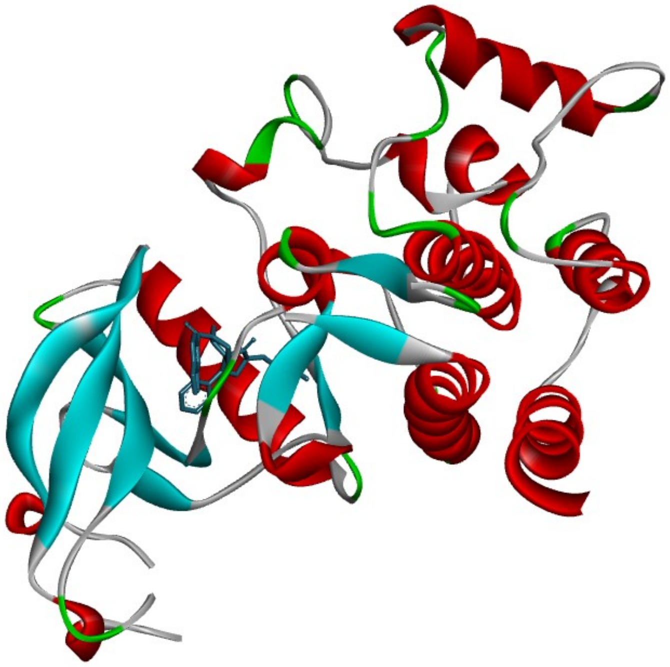

Fig 5. The crystal structure of DDR1, 2-[8-(1H-indazole-5-carbonyl)-4-oxo-1-phenyl-1,3,8-triazaspiro [4.5] decan-3-yl] -N-methyl acetamide (6FEW). 3D graphics were generated using Discovery Studio Visualizer 2021.

The binding pattern of indazole derivatives with PBD-6FEW. A) represents 3D surface representation, B) represents active ligand catalytic centre of the protein target, C) 2D-schematic LigPlot interactions shown for the docked pose of indazole derivatives shown by the spokes

8a

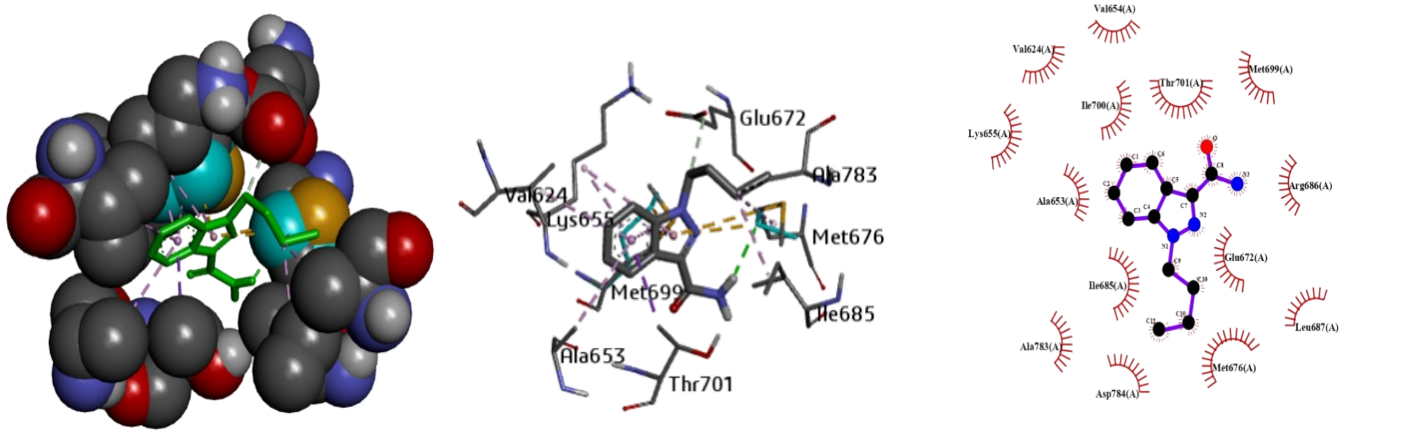

A B C

8b

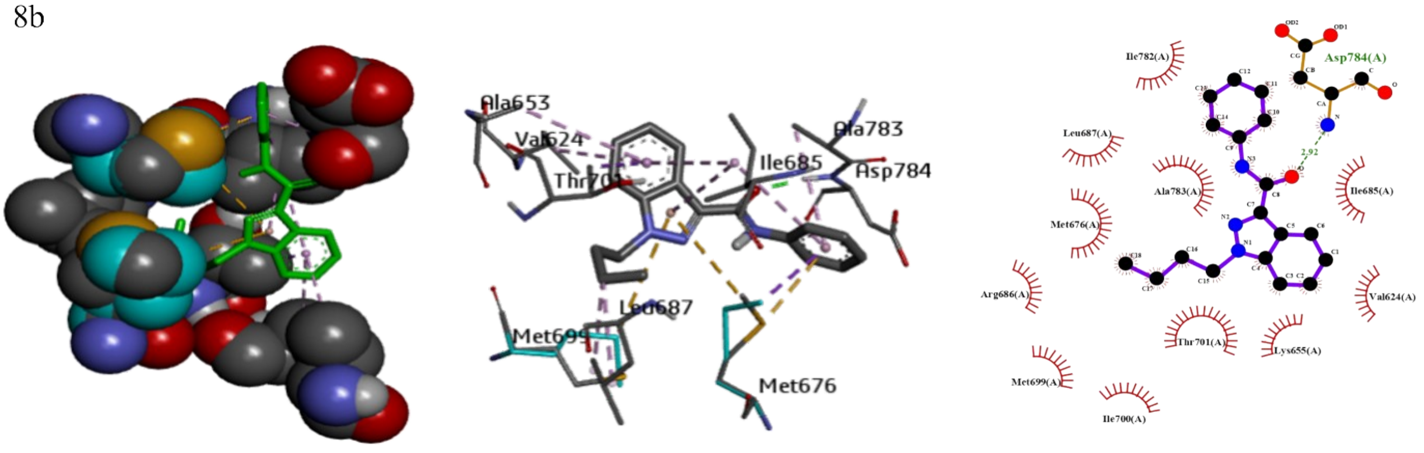

A B C

8c

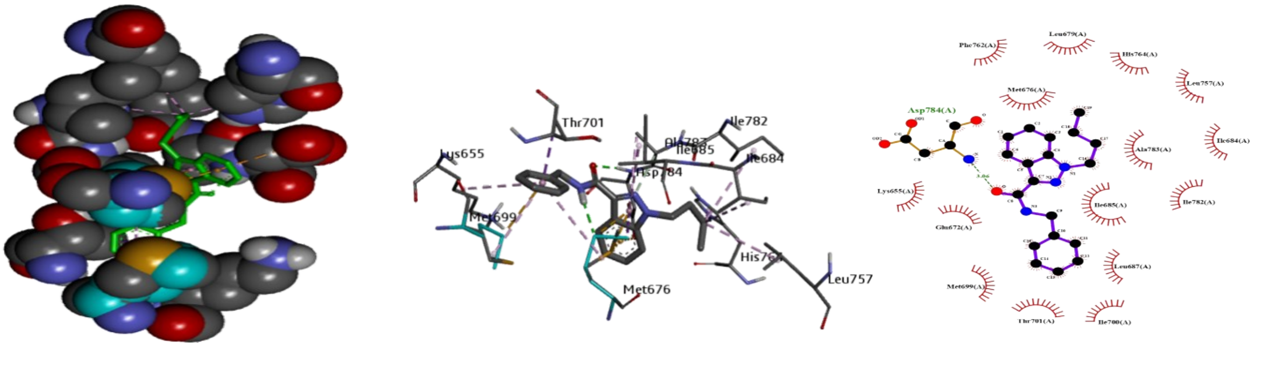

A B C

8d

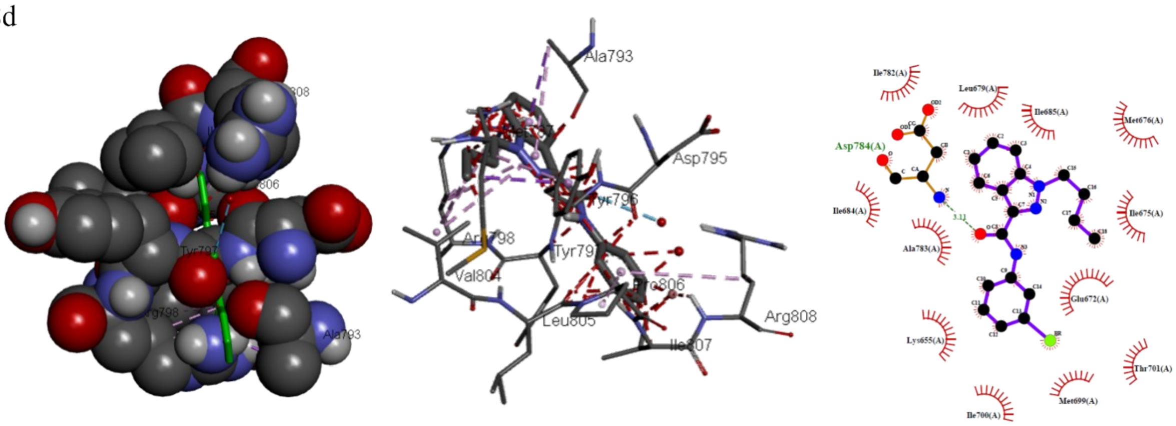

A B C

8e

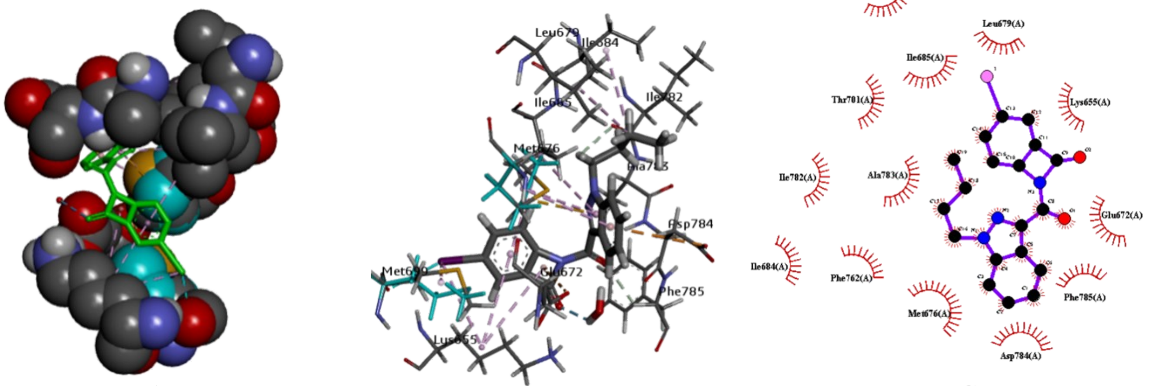

A B C

8f

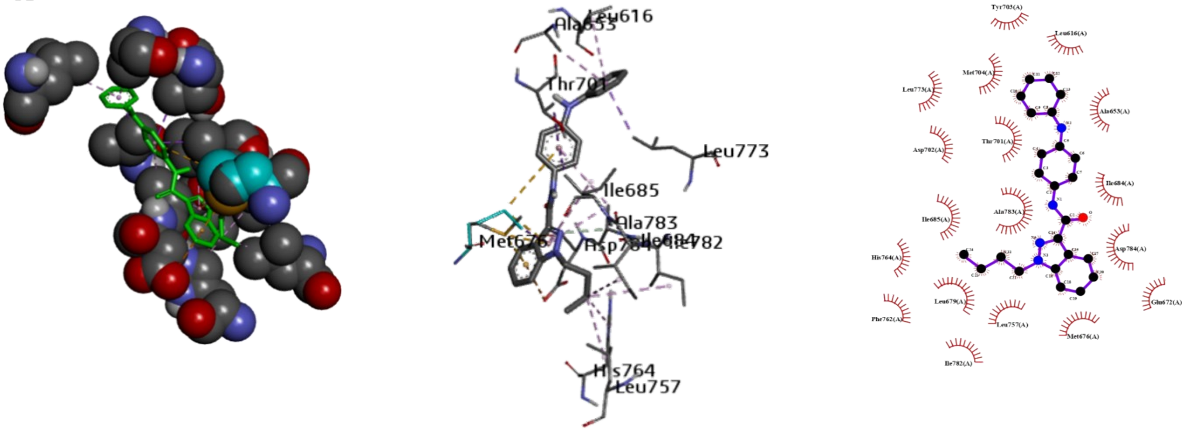

A B C

8g

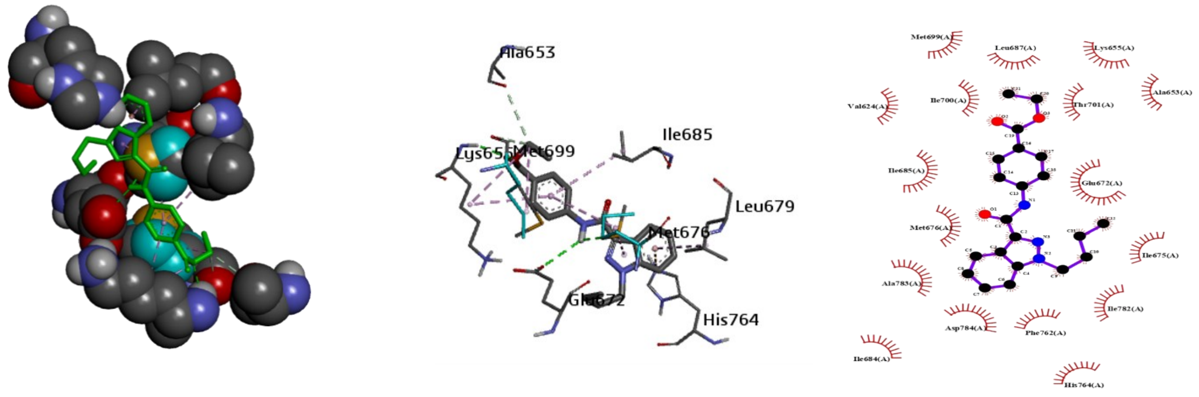

A B C

8h

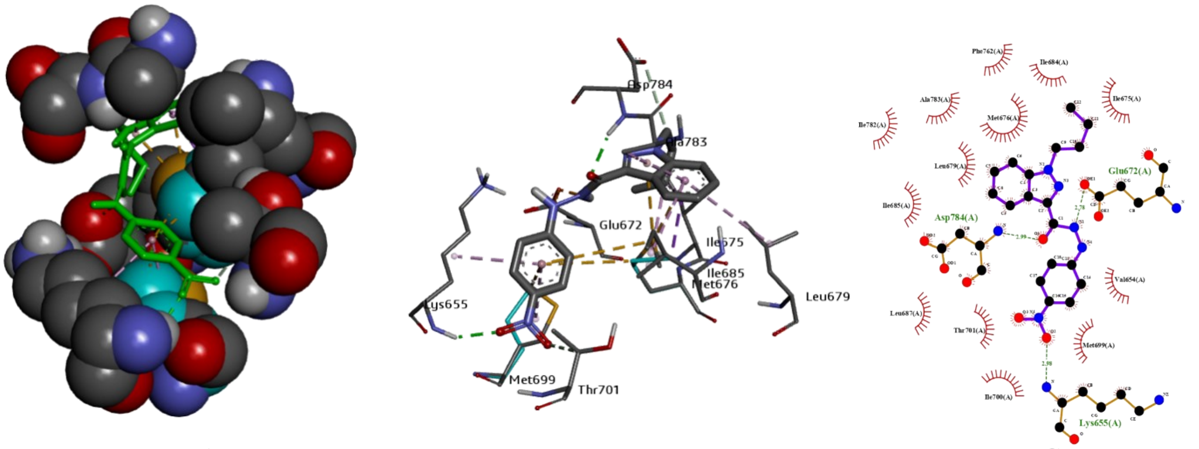

A B C

8i

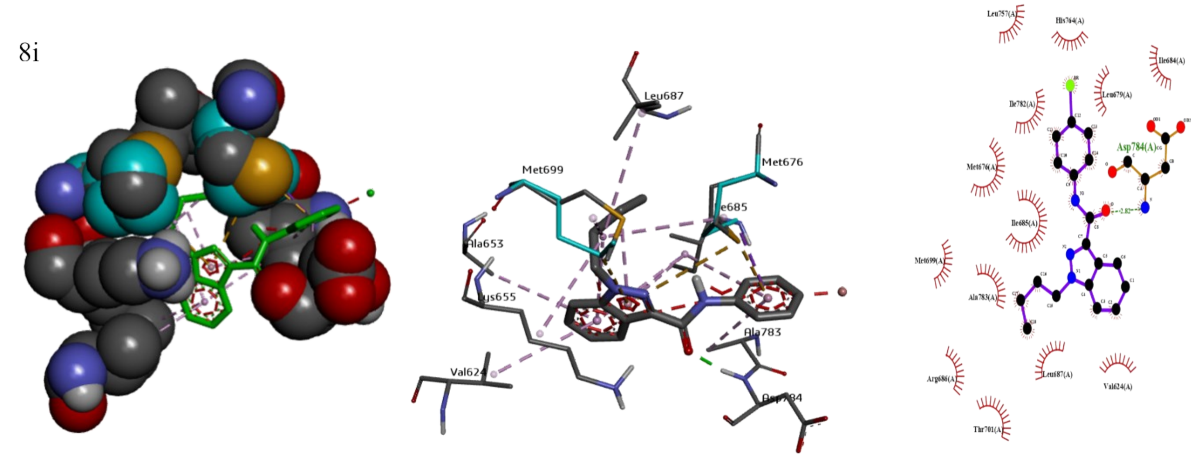

A B C

8j

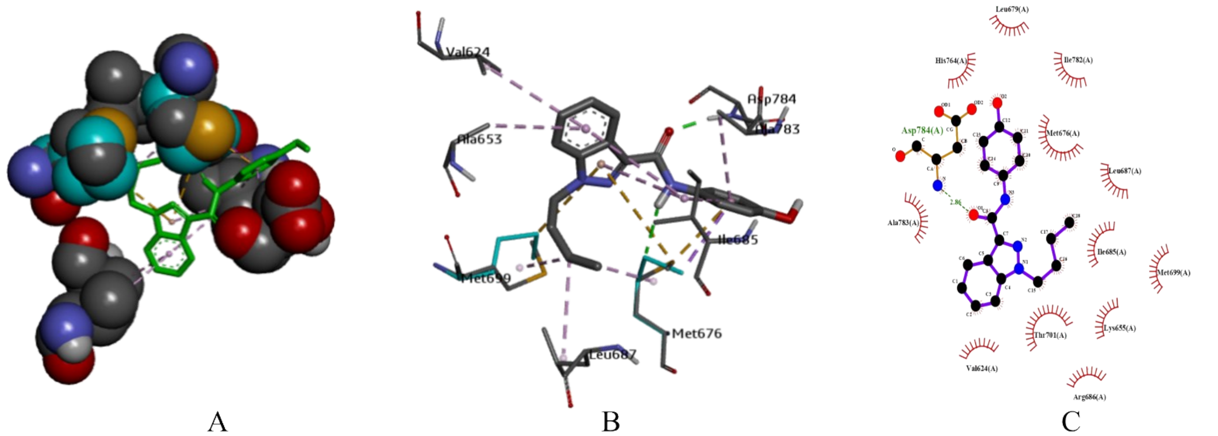

A B C

8k

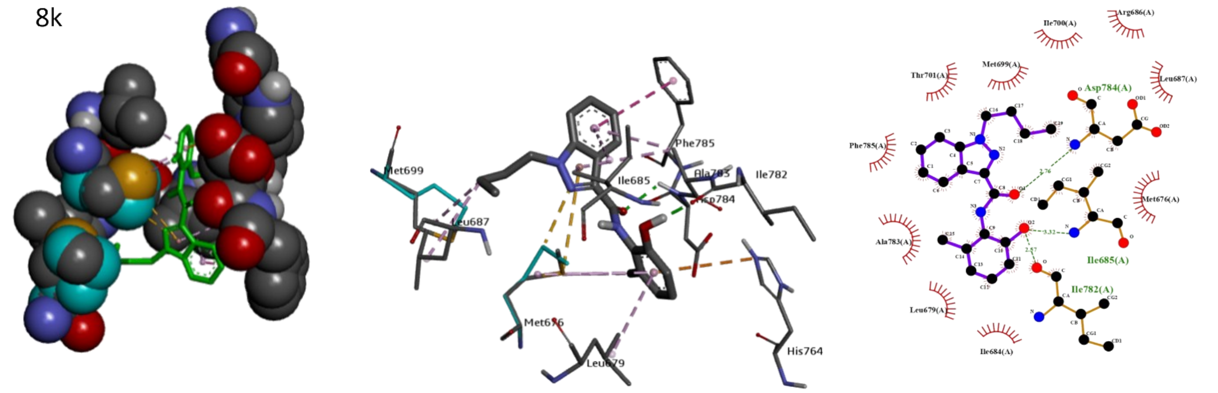

A B C

8l

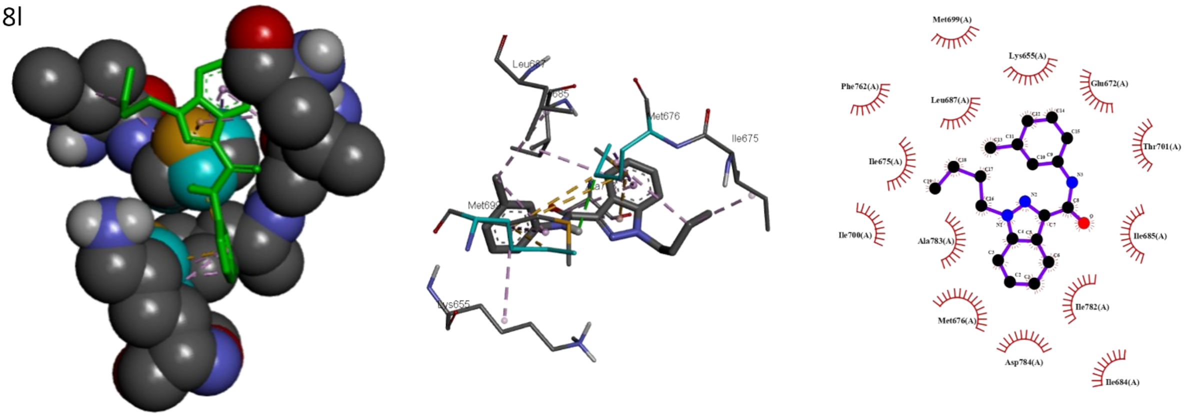

A B C

8m

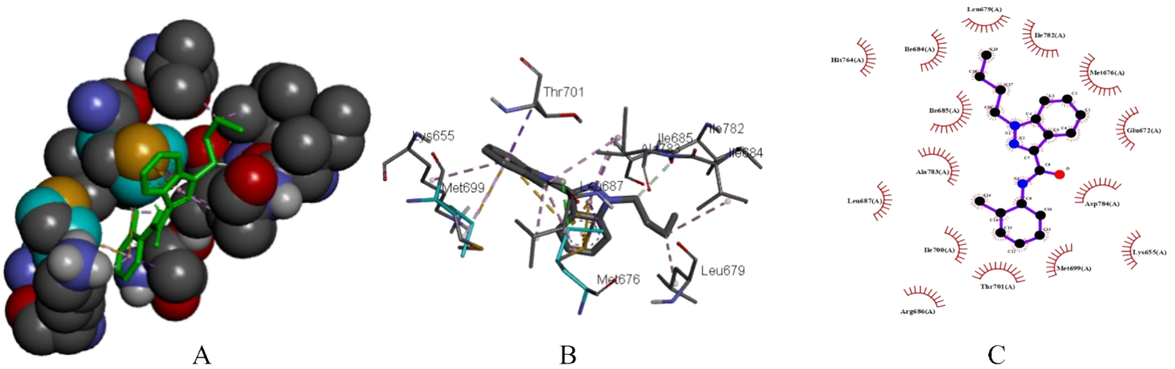

A B C

8n

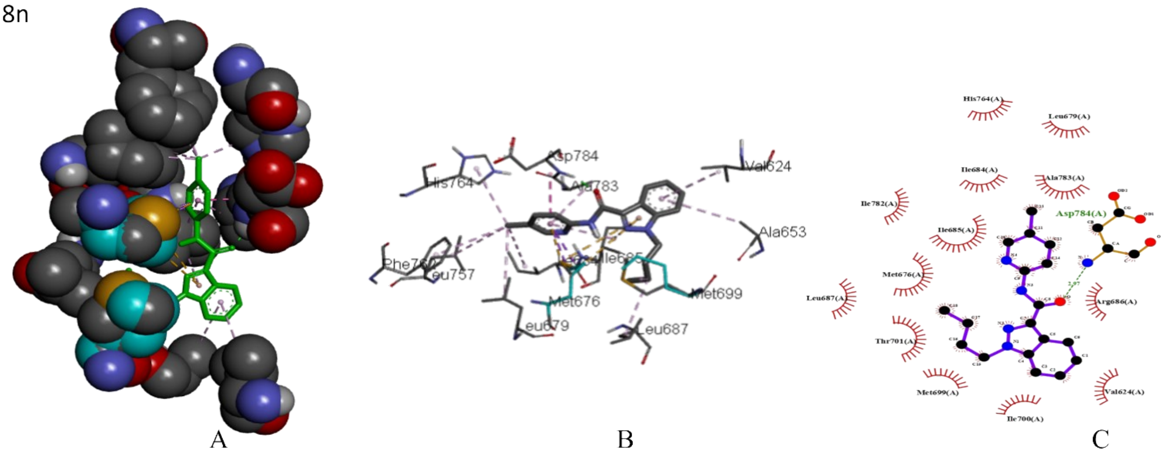

A B C

8o

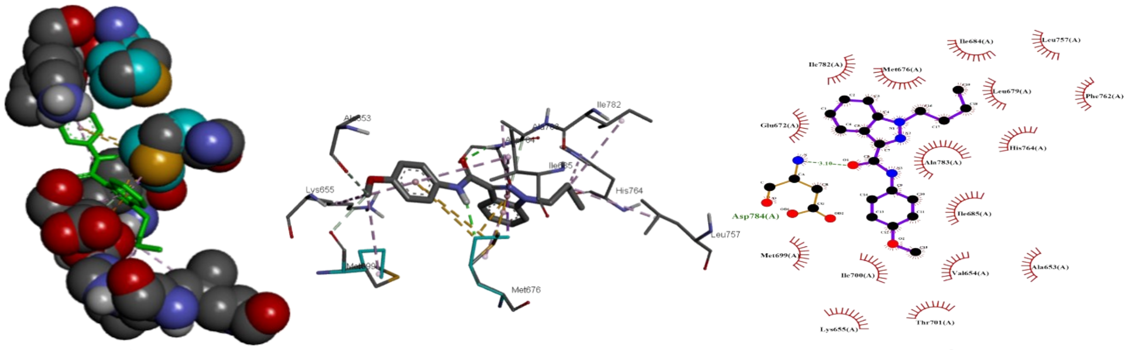

A B C

8p

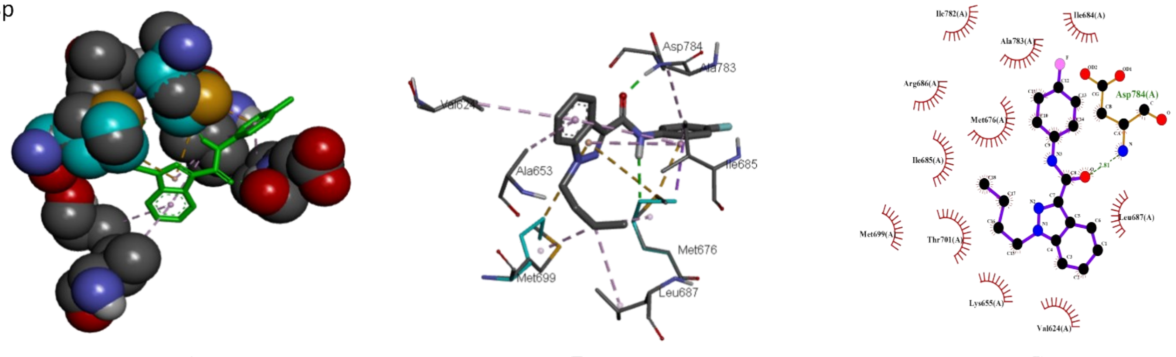

A B C

8q

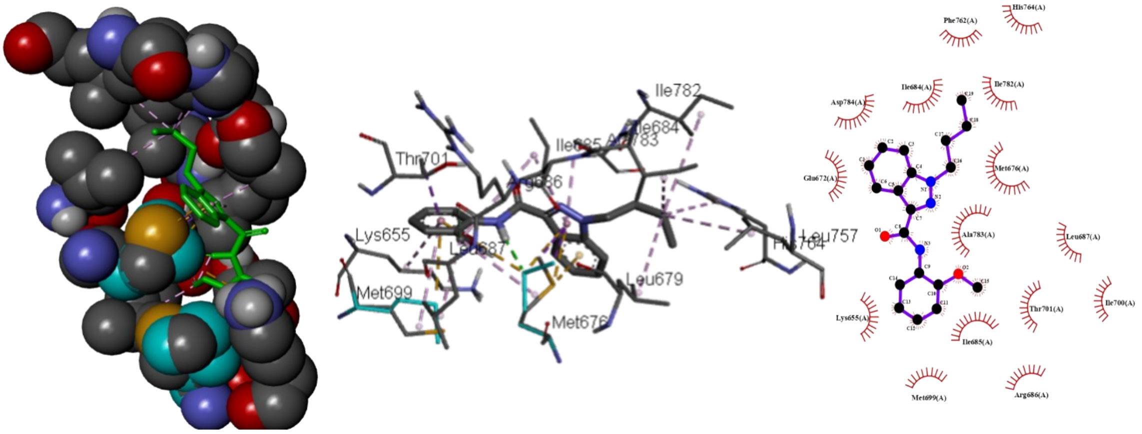

A B C

8r

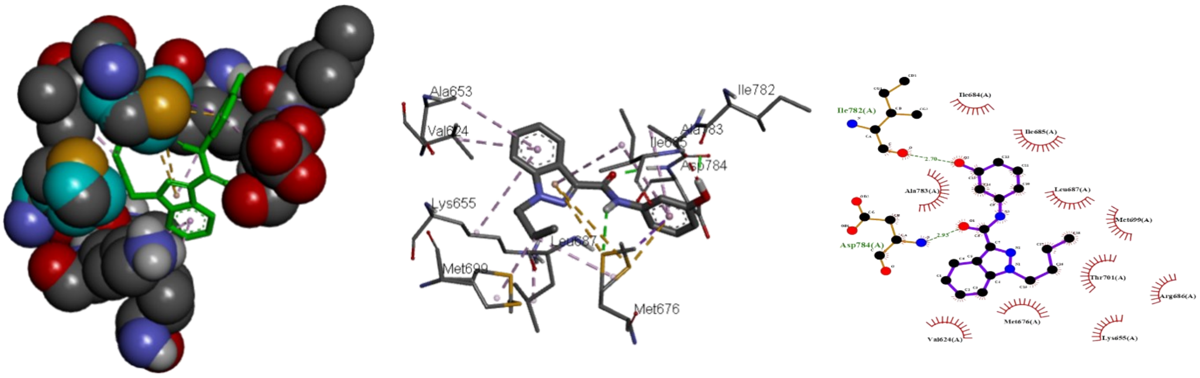

A B C

8s

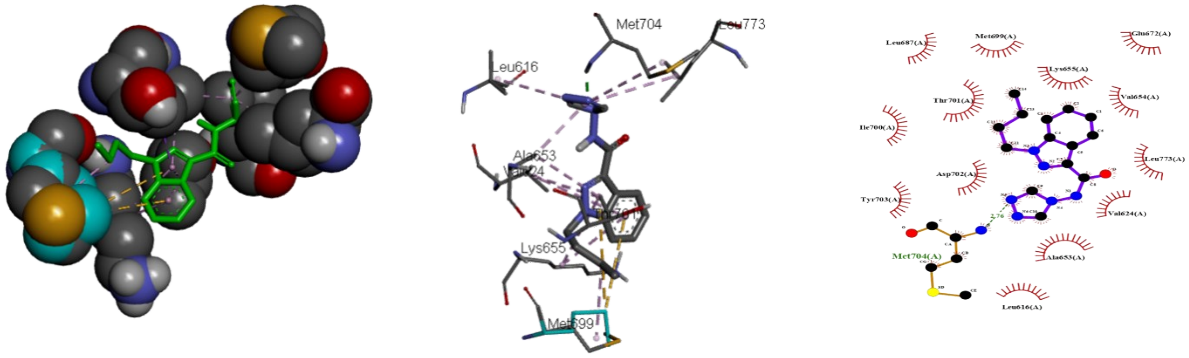

A B C

8t

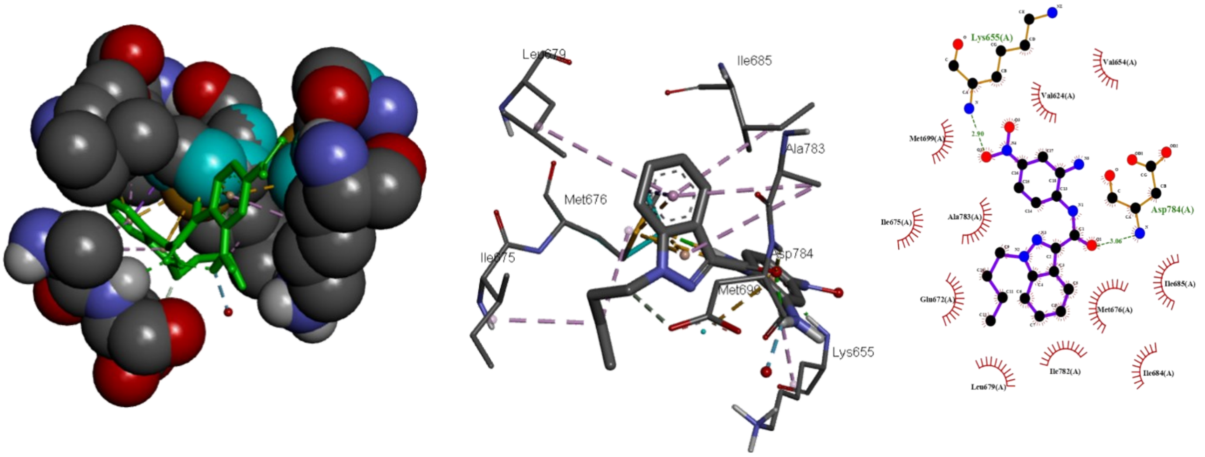

A B C

8u

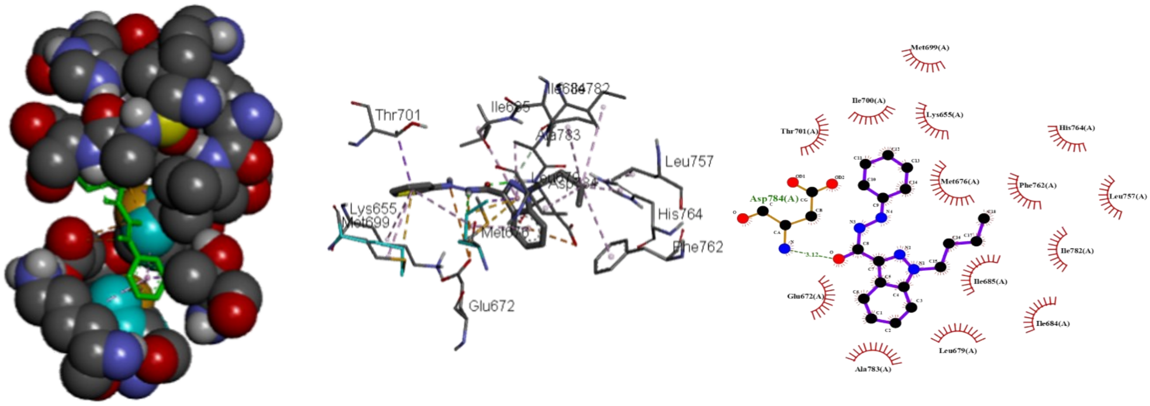

A B C

8v

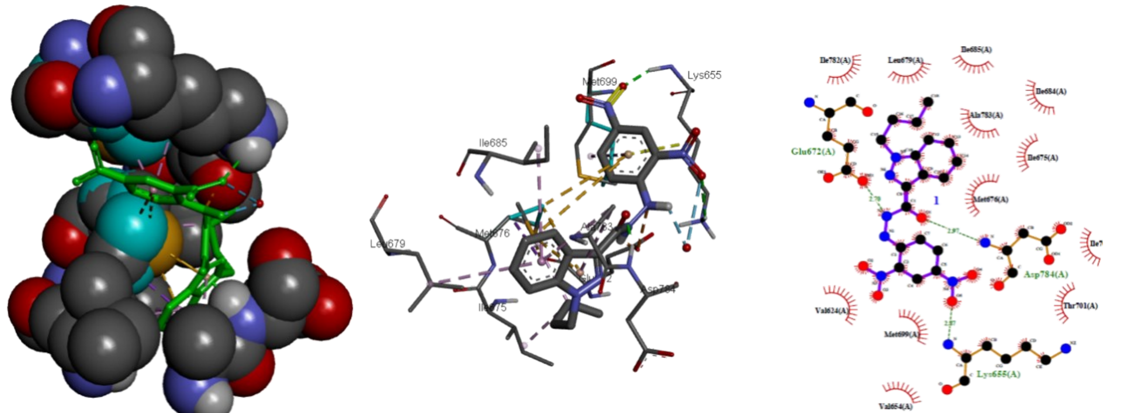

A B C

8w

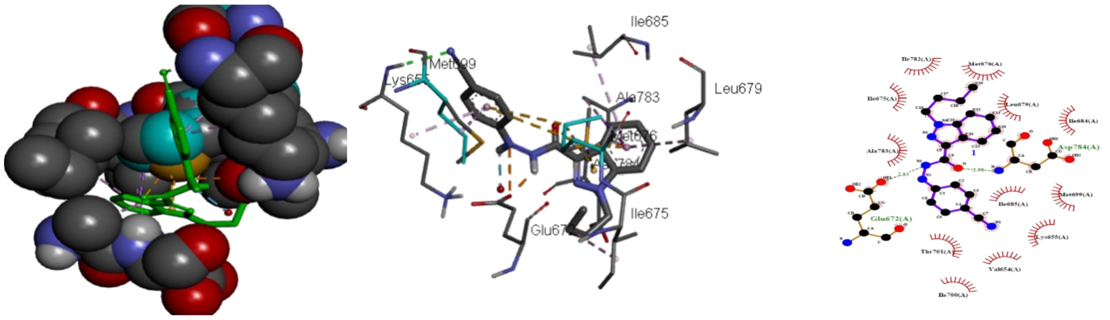

A B C

8x

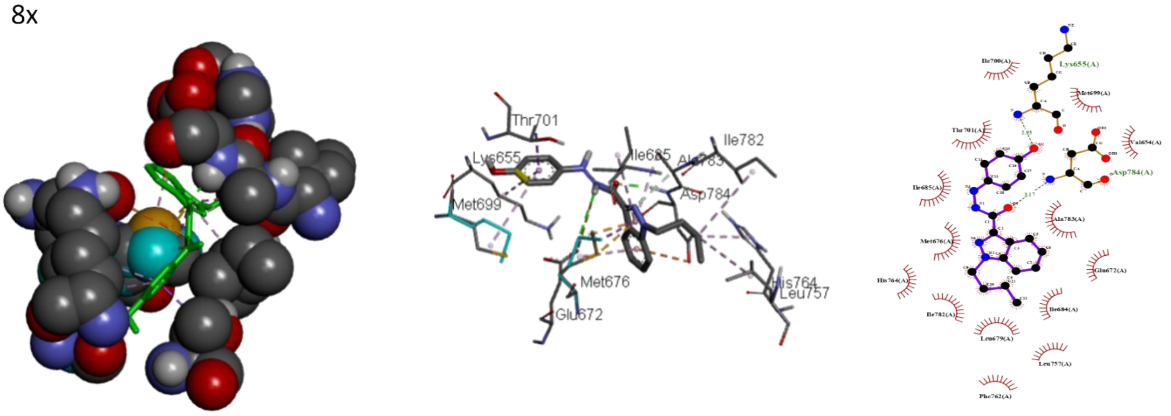

A B C

8y

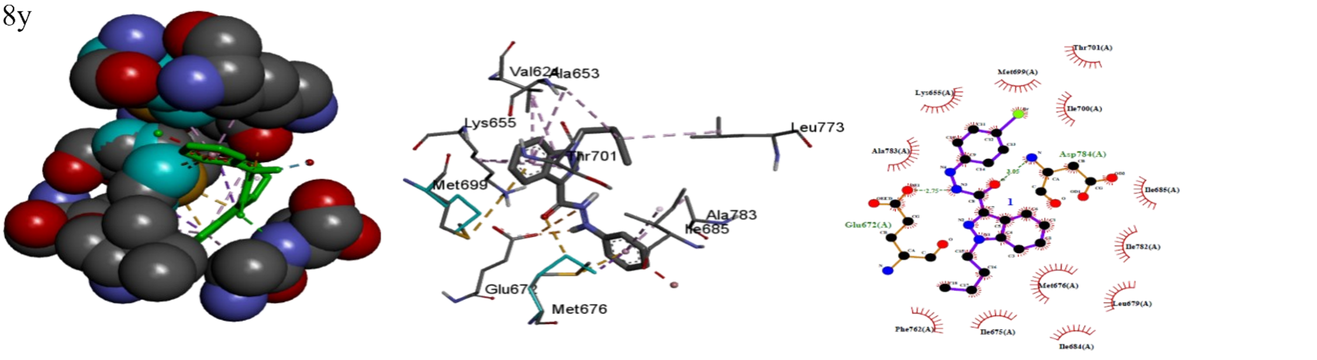

A B C

8z

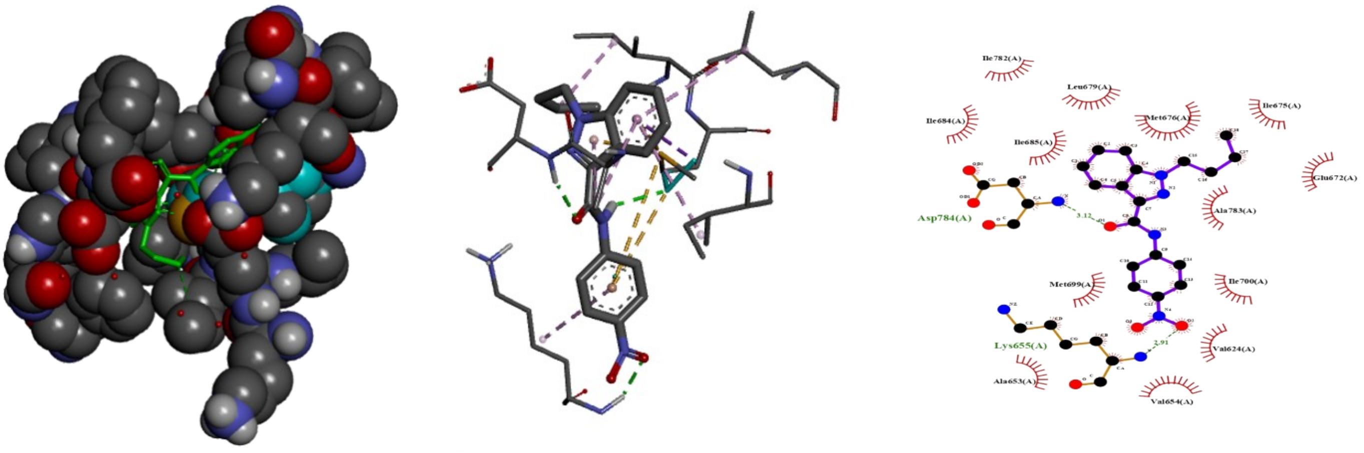

A B C

Reference

1 O. s. Kim, J. H. Jang, H. T. Kim, S. J. Han, G. C. Tsui and J. M. Joo, *Org Lett*, 2017, **19**, 1450-1453.

2 N. Lohitha and V. Vijayakumar, *Polycycl Aromat Compound*, 2022, **42**, 5521-5533.

3 S. Khaidem, S. Sarveswari, R. Gupta and V. Vijayakumar, *Cheminform*, 2012, **2(2)**, 258-266.

4 P. Ghosh, S. Mondal and A. Hajra, *J. Org. Chem*, 2018, **83**, 13618-13623.

5 C. K. Chung, P. G. Bulger, B. Kosjek, K. M. Belyk, N. Rivera, M. E. Scott, G. R. Humphrey, J. Limanto, D. C. Bachert and K. M. Emerson, *Org. Process Res. Dev* 2014, **18**, 215-227.

6 L. Yadav and S. Chaudary, *Org. Biomol. Chem*, 2020, **18**, 5927-5936.

7 Y. Wan, S. He, W. Li and Z. Tang, *Anticancer Agents Med Chem*, 2018, **18**, 1228-1234.

8 O. Pontes, S. Oliveira-Pinto, F. Baltazar and M. Costa, *Drug Discov Today*, 2022, 27, 304–314.

9 W. Wei, Z. Liu, X. Wu, C. Gan, X. Su, H. Liu, H. Que, Q. Zhang, Q. Xue, L. Yue, L. Yu and T. Ye, *RSC Adv*, 2021, **11**, 15675-15687.

10 S. puri and K. Juvale, *J. Mol. Struct*, *1269*, 133727. https://doi.org/10.1016/j.molstruc.2022.133727.

11 A. Dey and A. Hajra, *J. Org. Chem*, 2019, 84, 14904-14910.

12 C. Kucuk, S. Celik, S. Yurdakul, E. Cotelı and B. Erden, *Polyhedron*, https://doi.org/10.1016/j.poly.2023.116469.

13 G. M. Morris, H. Ruth, W. Lindstrom, M. F. Sanner, R. K. Belew, D. S. Goodsell and A. J. Oslon, *J. Comput. Chem*, 2009, **30**, 2785-2791.

14 K. Murugavel, S. Amirthaganesan, R. Rajamohan, S. Bharanidharan and T. Sabapathy Mohan, *J. Mol. Struct*, https://doi.org/10.1016/j.molstruc.2023.135299.

15 V. Hemalatha and V. Vijayakumar, *Inorg Chem Commun*, https://doi.org/10.1016/j.inoche.2022.109894.

16 P. Karuppusamy and S. Sarveswari, *J. Mol. Struct*, https://doi.org/10.1016/j.molstruc.2021.131494.

17 R. Bhaskar and S. Sarveswari, ChemistrySelect, 2020, 5, 4050-4057.

18 S. Sarveswari, A. J. Beneto and A. Siva, *Sens Actuators B Chem*, 2017, 245, 428-434.

19 T. Uelisson da silva, E. Tomaz da silva, K. De Carvalho Pougy, C. Henrique da silva Lima and S. De Paula Machado, *Inorg. Chem Commun*, https://doi.org/10.1016/j.inoche.2021.109120.

20 M. A. Mumit, T. K. Pal, M. A. Alam, M. A. A. A. A. Islam, S. Paul and M. C. Sheikh, *J. Mol. Struct*, https://doi.org/10.1016/j.molstruc.2020.128715.

21 J. K. Ojha, G. Ramesh and B. V. Reddy, *Chemical physics impact*,

https://doi.org/10.1016/j.chphi.2023.100280.
22 A. Jumabaen, U. Holikulov, H. Hushvaktov, N. ISSAOUI and A. Absanov, *J. Mol. Liq*, https://doi.org/10.1016/j.molliq.2023.121552.
23 S. Y. Ghansenyuvy, K. O. Eyong, P. Yemback, L. Mehreen, V. De. P. N. Nziko, M. S. Ali and G. N. Folefoc, *Eur. J. Med. Chem. Rep*, https://doi.org/10.1016/j.ejmcr.2023.100108.
24 M. K. Sharma, S. Parashar, D. Sharma, K. Jakhar, K. Lal, N. U. Pandya and H. Om, *J. Ind. Chem. Society*, https://doi.org/10.1016/j.jics.2023.100973.
25 C. Yuan and X. Hao, *Heliyon*, https://doi.org/10.1016/j.heliyon.2023.e18742.
26 E. Zarenezhad, E. Behmard, I. Sadeghian, S. Sadeghian, A. Ghanbariasad, A. Ghasemian, S. Behrouz, A. Zarenezhad and N. S. Rad, *J. Mol. Struct*, https://doi.org/10.1016/j.molstruc.2023.135378
